# Psychedelics align brain activity with context

**DOI:** 10.1038/s41586-026-10910-z

**Published:** 2026-08-19

**Authors:** Devon Stoliker, Leonardo Novelli, Moein Khajehnejad, Mana Biabani, Matthew D. Greaves, Tamrin Barta, Martin Williams, Sidhant Chopra, Olivier Bazin, Otto Simonsson, Richard Chambers, Frederick S. Barrett, Gustavo Deco, Katrin H. Preller, Robin L. Carhart-Harris, Anil K. Seth, Suresh Sundram, Gary F. Egan, Adeel Razi

**Affiliations:** 1 Turner Institute for Brain and Mental Health, School of Psychological Sciences, Monash University, Melbourne, Victoria Australia; 2 School of Health Sciences, Swinburne University of Technology, Hawthorn, Victoria Australia; 3 Orygen, The University of Melbourne, Melbourne, Victoria Australia; 4 Centre for Youth Mental Health, The University of Melbourne, Melbourne, Victoria Australia; 5 British Association of Mindfulness-based Approaches (BAMBA) Listed Teacher, Oxford, UK; 6 Department of Neurobiology, Care Sciences and Society, Karolinska Institute, Solna, Sweden; 7 Monash Centre for Consciousness and Contemplative Studies, Monash University, Melbourne, Victoria Australia; 8 Department of Psychiatry and Behavioral Sciences, Center for Psychedelic and Consciousness Research, Johns Hopkins University School of Medicine, Baltimore, MD USA; 9 Center for Brain and Cognition, Theoretical and Computational Group, Universitat Pompeu Fabra/ICREA, Barcelona, Spain; 10 Department of Adult Psychiatry and Psychotherapy, Psychiatric University Clinic Zurich and University of Zurich, Zürich, Switzerland; 11 Centre for Psychedelic Research, Department of Brain Sciences, Imperial College London, London, UK; 12 Psychedelics Division, Neuroscape, University of California, San Francisco, San Francisco, CA USA; 13 Sussex Centre for Consciousness Science, Department of Informatics, University of Sussex, Brighton, UK; 14 Program on Brain, Mind, and Consciousness, Canadian Institute for Advanced Research, Toronto, Ontario Canada; 15 Department of Psychiatry, School of Clinical Sciences, Monash University, Clayton, Victoria Australia; 16 Monash Biomedical Imaging, Monash University, Melbourne, Victoria Australia; 17 CIFAR Global Scholars Program, Toronto, Ontario Canada

**Keywords:** Consciousness, Human behaviour, Neural decoding, Dynamical systems, Emergence

## Abstract

Psychedelics can profoundly alter consciousness by reorganizing brain connectivity^1,2^, producing acute experiences that shape lasting psychological change^3,4^. Psychedelic dynamics are commonly described as desynchronized or entropically disordered^5,6^, yet the brain organization underlying self-dissolving and boundary-dissolving experiences that participants often report^7^, and how context shapes that organization^8^, remain unresolved. To address this, we acquired the largest single-site psychedelic neuroimaging dataset to date. Sixty-two adults underwent functional magnetic resonance imaging (fMRI) and electroencephalography (EEG) during rest and naturalistic stimuli (meditation, music and movie), before and on the day of psilocybin administration (fMRI ~ 80 min post-dose; EEG ~ 150 min post-dose). Half ranked the experience among the most meaningful of their lives^7^. Here, using machine learning to represent the brain dynamics of each individual as low-dimensional trajectories, we show that psilocybin reorganizes brain activity into structured, context-sensitive patterns that co-vary with the quality of subjective experience, revealing a latent order missed by time-averaged measures. Networks that ordinarily segregate internal and external processing integrated, producing cohesive context-aligned trajectories in participants reporting the felt experience of being continuous with, rather than separate from, the environment, a state we refer to as embeddedness. The strength of this context alignment scaled with both the depth of self-dissolving and boundary-dissolving experience and the next-day mindset change. Our findings recast apparent disorder as latent organization aligned with context, linking neurobiology to subjective experience and behavioural change.

## Abstract (web summary)

The psychedelic psilocybin reorganizes brain activity into structured, context-aligned patterns that integrate internal and external processing, providing a neural basis for the felt continuity between self and world, elucidating how the psychedelic state translates into psychological change.

## Main

The profound effects of psychedelics reshape subjective responses to internal and external sensations and are frequently reported as among the most meaningful experiences in life^7^. These states can manifest sustained therapeutic benefits, including reductions in depression, anxiety and addiction, alongside an increase in social connectedness and overall well-being^3,4,9–14^.

The brain constructs perception and selfhood by integrating external sensory inputs with internal models of the environment^15^. These interactions between sensory and associative brain regions are enabled by structural and functional connectivity^16^. Psychedelics such as psilocybin disrupt these interactions by acting at the serotonin 5-HT_2A_ receptor, inducing structural and functional plasticity in preclinical models—rapid medial prefrontal cortex spinogenesis persisting for weeks, mechanistically linked to enduring behavioural effects^17,18^—that can reshape macro-level connectivity^19–22^. Studies that examine effective connectivity further suggest that associative network communication becomes reconfigured^23–27^. Reflecting this reorganization, individuals frequently report an intensified sense of immersion, in which the context of space, time and selfhood feels deconstructed and interconnected^28,29^. Although the exact mechanisms linking brain network-level shifts to subjective experience remain unknown, these dynamics have typically been characterized as entropic and desynchronized^5,6,30,31^. A central mediator of these effects is the default mode network (DMN), which supports the integration of information from diverse associative regions spanning spatial, temporal and self-referential contexts^32–37^. Under psilocybin, connectivity patterns that constrain networks relax, permitting novel interactions between sensory and associative regions to emerge^38,39^. This reconfiguration alters connectivity patterns that underlie emotion, cognition and perception^40^. Of note, in healthy adults, psilocybin produces a persistent reduction in anterior hippocampus (aHip)–DMN connectivity, and in rodents, preclinical studies have reported acute DMN hypoconnectivity^6,41^. Although observed in non-clinical samples, this pattern has been proposed as mechanistically relevant to therapeutic effects. However, despite mounting evidence for these shifts in connectivity, how they give rise to meaningful and therapeutically relevant experiences remains unclear.

Psychedelic effects are also context-sensitive, shaped by mindset and setting^8^ and recognized in clinical guidelines^42^. Understanding how context influences therapeutic change, and the underlying brain connectivity that can be harnessed clinically, requires a comprehensive examination of how psychedelics reshape functional integration of the brain.

Existing studies often rely on small sample sizes, participants with psychedelic experience, limited imaging modalities or single-context designs, making it difficult to generalize findings. Structured cognitive tasks imposed during imaging also risk conflating task demands with the psychedelic state itself, rather than capturing how the state unfolds naturalistically. Some recent work has partly addressed these limitations^2,43,44^, but no study to date has combined large-scale sampling, multimodal imaging and diverse naturalistic contextual manipulations within a single acute session and computational framework.

**Extended Data Fig. 1:**
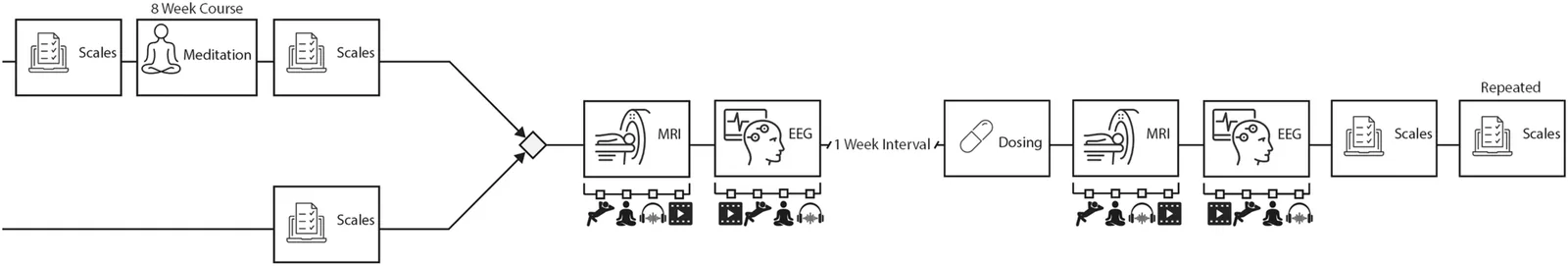
Simplified overview of the study design. Participants were assigned to 8-week mindfulness meditation course or immediately enrolled to multimodal (fMRI and EEG) imaging sessions. An open label ’baseline’ (no-psilocybin) imaging session was conducted approximately one week before imaging under administration of 19 mg psilocybin. MRI and EEG imaging began approximately 80 and 150 minutes post-dose, respectively. Music, mindfulness, personality and psychedelic measures were collected before and longitudinally after psilocybin. Meditators received additional assessments before and after the mindfulness meditation course. Mindfulness meditation training showed no differences in our connectivity and subjective effect analyses, and was subsequently collapsed for group-level analyses. See Methods for details. Illustrations of the meditating person by gentle studio/iStock, the resting person by grafikazpazurem/iStock, the scales by Anton Porkin/iStock, the MRI and EEG by Takoyaki Tech/iStock, the dosing by Anastasia Malachi/iStock, the headphone by mehmetbuma/iStock and the movie by Intellson/iStock by Getty Images.

We acquired the largest single-site acute-phase psychedelic neuroimaging dataset to date, integrating multimodal fMRI and EEG with controlled contextual manipulations across eyes-open and eyes-closed states in a cohort with no lifetime psychedelic experience (*n* = 62). Experimental conditions of rest, meditation, music and movie were systematically varied, and extensive behavioural measures were collected (Extended Data Fig. 1).

Our analyses show that psilocybin redistributes integration, increasing global functional connectivity in associative regions while reducing it in sensory areas, altering the balance of internally and externally directed processing. Across fMRI and EEG, signals recorded during eyes-closed conditions became similar to those of an eyes-open condition. Critically, using machine learning, we uncovered context-aligned organization in brain activity that was missed by conventional analyses. This reorganization was experientially graded, emerging under positively felt self-dissolving and boundary-dissolving experiences, and not during negatively felt states or cognitive impairment.

These results identify a state-dependent neural signature that emerges when network organization becomes flexibly aligned with context, coinciding with the subjective state that we refer to as embeddedness. We find this state to be consequential for subsequent change and contextually modifiable. The finding that the organization of brain activity tracks the transformative quality of subjective experience helps to elucidate how the psychedelic state translates into psychological change.

### Psilocybin redistributes cortical GFC

**Fig. 1:**
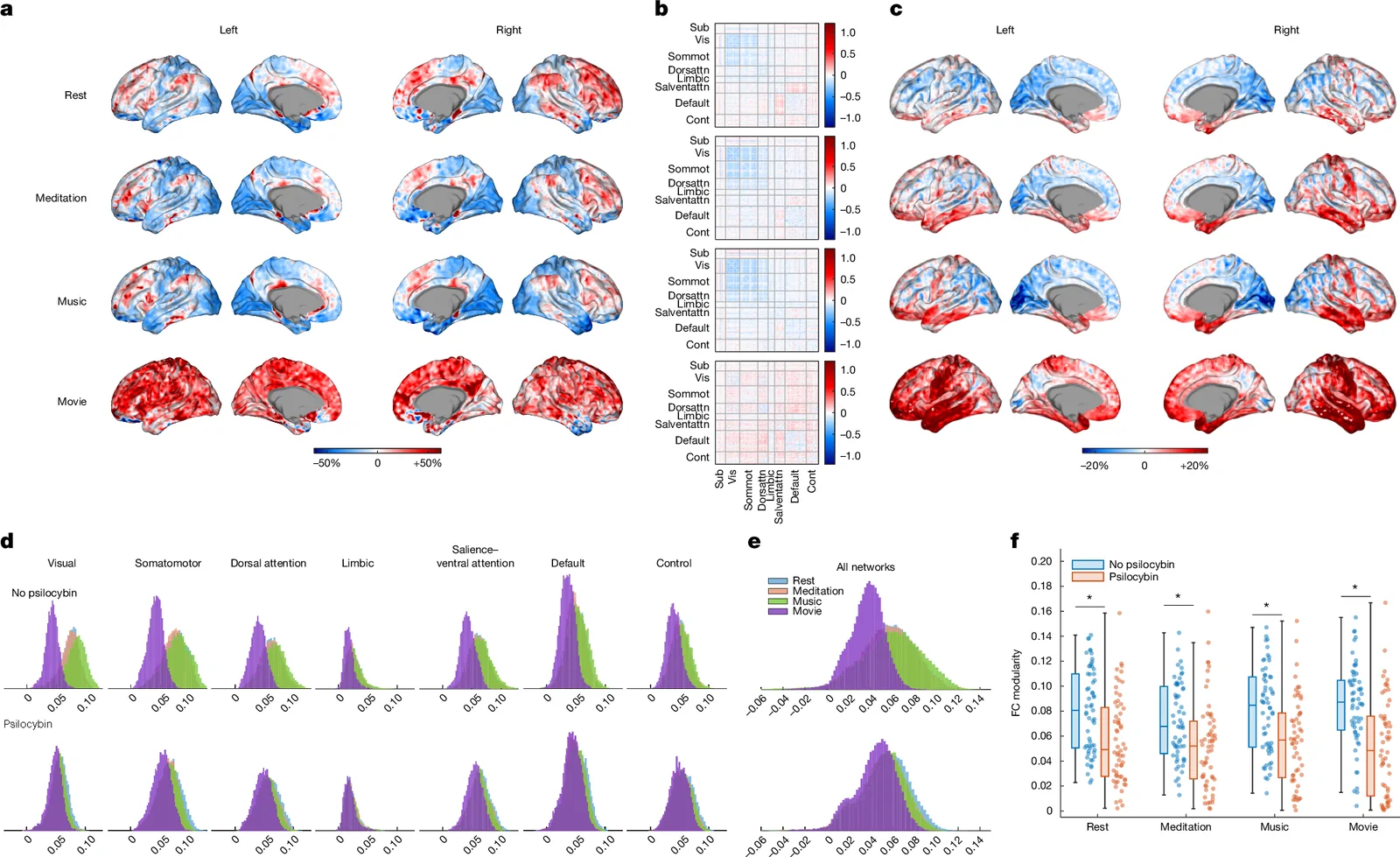
Global redistribution of time-averaged functional connectivity under psilocybin. **a**, GFC percentage change (psilocybin versus no psilocybin) averaged across all participants for each condition (row). The significant clusters after threshold-free cluster enhancement are shown in Extended Data Fig. 2a. **b**, Average functional connectivity difference (psilocybin minus no psilocybin) across participants. Psilocybin reduced functional connectivity within networks during eyes-closed conditions. The network names are: subcortex (sub), visual (vis), somatomotor (sommot), dorsal–ventral attention (dorsventattn), limbic, salience–ventral attention (salventattn), default mode (default) and control (cont). A larger version of these functional connectivity matrices is provided in Extended Data Fig. 4b. **c**, s.d. percentage change (psilocybin minus no psilocybin) computed for each participant and then averaged across participants, for each condition. Only cortical vertices where s.d. changes were not significantly associated with framewise displacement are shown. **d**, Histograms of GFC values for seven resting-state networks (columns) and four conditions (colour-coded). The gap between eyes-closed and eyes-open conditions at baseline (no psilocybin; top row) vastly reduces after psilocybin (bottom row). This reduction was most prominent for the visual, somatomotor and dorsal attention networks, but was larger than −66% in all networks and statistically significant (Extended Data Fig. 4a). **e**, Histograms of GFC values combining all networks. **f**, Psilocybin reduces functional connectivity (FC) modularity across conditions (Cohen’s *d* ≤ −0.6) for all comparisons. Each point corresponds to one participant (rest *n* = 60, meditation *n* = 59, music *n* = 56 and movie *n* = 57), the boxes indicate the median and quartiles, and the whisker length is 1.5 times the interquartile range. The asterisks indicate a significant decrease using the Mann–Whitney *U*-test (right-tailed, all *P* < 10^−3^).

**Extended Data Fig. 2:**
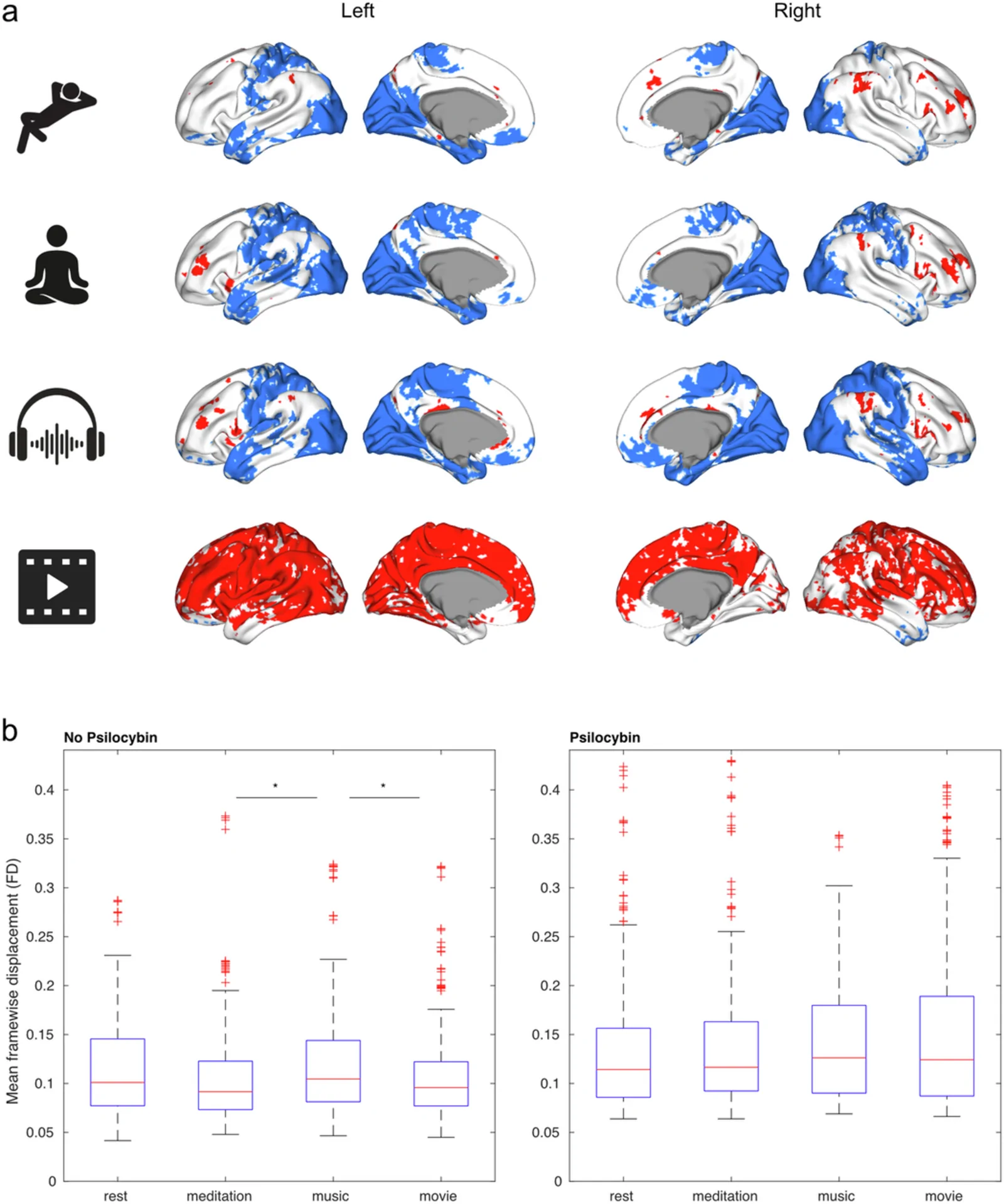
Cluster-level significance of GFC changes and motion-control validation. **a**, Clusters of statistically significant global functional connectivity increase (red) or decrease (blue) at *P* < 0.05 after threshold-free cluster enhancement. **b**, Head motion comparison across conditions within session, quantified via the mean framewise displacement (FD). Left: Baseline session. Mean FD increased modestly between guided meditation and music listening (Mann-Whitney U-test, two-sided, *P* = 0.003) and decreased between music listening and movie watching (*P* = 0.004); both differences, though statistically significant, were small in magnitude. The boxes indicate the median and quartiles, and the whisker length is 1.5 times the interquartile range (sample sizes: rest *n* = 240, meditation *n* = 236, music *n* = 224, movie *n* = 228. Four echoes for each participant). Right: Psilocybin session. No statistically significant differences were observed between conditions (Mann-Whitney U-test, two-sided, all *P* > 0.18). Illustrations in panel a of the meditating person by gentle studio/iStock, the resting person by grafikazpazurem/iStock, the headphone by mehmetbuma/iStock and the movie by Intellson/iStock by Getty Images.

Psilocybin induced distinct shifts in global integration of functional connections across brain regions. This was quantified using global functional connectivity (GFC), a measure of the average correlation of blood oxygen level-dependent (BOLD) signals between each vertex and every other vertex in a high-resolution cortical surface map^45^. During eyes-closed conditions (rest, meditation and music), psilocybin reduced the GFC of sensory regions (Fig. 1a), and these reductions formed statistically significant clusters (Extended Data Fig. 2a). Occipital areas showed the strongest overall reductions (percentage changes up to −39% and Cohen’s *d* = −0.55), with midline ventral parietal decreases prominent during rest, meditation and music. Conversely, GFC increased in associative areas during these conditions (percentage changes up to +72%, Cohen’s *d* = 0.53). This evidence indicates that, during eyes-closed conditions, psilocybin suppressed the integration of sensory regions with the broader cortical network and enhanced the integration of associative regions. By contrast, during the eyes-open movie condition, GFC increased in both sensory and associative areas (Fig. 1a), enhancing global synchrony, independent of head motion (Extended Data Fig. 2b). These eyes-closed shifts in GFC are directionally consistent with previous global connectivity studies under psychedelics during eyes-closed rest, which commonly report increases in associative or frontoparietal systems^46,47^ and, in degree centrality analyses, decreases in sensory or visual areas^48^. However, differences in metrics (such as global correlation and functional connectivity density), spatial domains (volumetric versus surface) and subcortical coverage limit direct comparisons.

**Extended Data Fig. 3:**
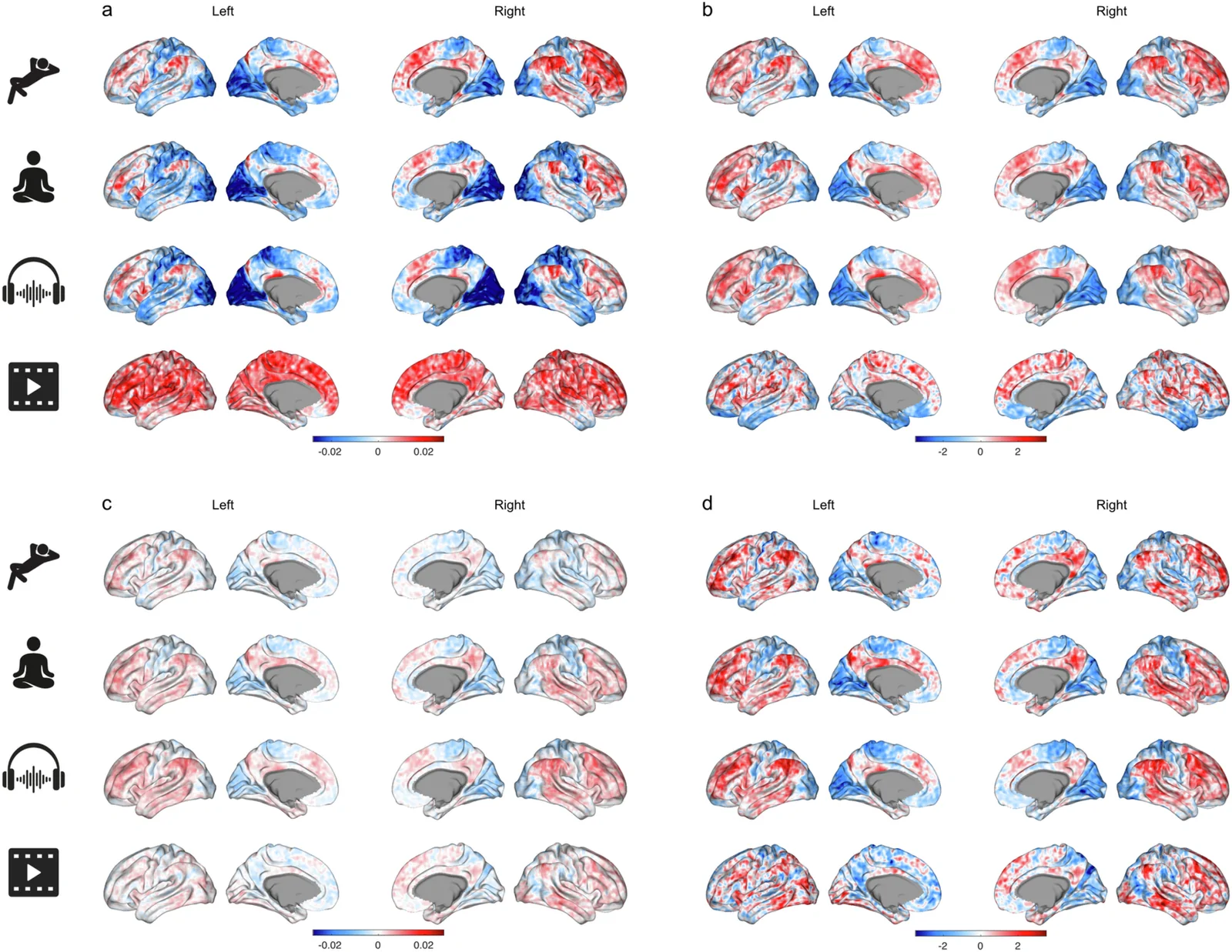
GFC change maps under z-scoring and global signal regression. **a**, Global functional connectivity (GFC) difference between the psilocybin and the no-psilocybin sessions. **b**, The same GFC difference plotted after z-scoring. Note how z-scoring confounds the interpretation by rendering movie-watching similar to the three eyes-closed conditions. This is because positive values below the average became negative after z-scoring. **c**, GFC difference after global signal regression (GSR). Note the smaller magnitude of the differences compared to panel a. In the Methods section, we argue that GSR complicates the interpretation of the GFC, confounding the intended goal of quantifying ‘the extent to which a cortical vertex is positively/negatively correlated with the other vertices, on average’ with ‘the extent to which a vertex is positively/negatively correlated with vertices having large standard deviation, on average’. **d**, GFC difference after both GSR and z-scoring. In addition to the previous confounds, z-scoring also hides the fact that GSR substantially reduces the magnitude of GFC values. Illustrations of the meditating person by gentle studio/iStock, the resting person by grafikazpazurem/iStock, the headphone by mehmetbuma/iStock and the movie by Intellson/iStock by Getty Images.

Group-level findings in eyes-closed conditions were robust to *z*-scoring and global signal regression (Extended Data Fig. 3; see Methods for the mathematical rationale for omitting these steps). Substantial individual variability in GFC changes was observed across participants (Supplementary Fig. 1).

### Context reshapes spatial BOLD variance

Mapping the standard deviation (s.d.) of the BOLD signal within participants across the cortex revealed that signal variability was spatially reorganized and condition dependent under psilocybin.

Compared with baseline (no psilocybin), the s.d. increased during the eyes-open movie condition in the orbitofrontal cortex, occipitotemporal gyri, inferior temporal lobes (particularly anterior regions) and the right hemisphere primary somatosensory cortex (percentage changes up to +19%, Cohen’s *d* = 0.54).

By contrast, s.d. decreased in early visual areas across all eyes-closed conditions, most strongly during music (percentage changes up to −10%, Cohen’s *d* = −0.38). During the eyes-open movie, we also detected spatially constrained s.d. decreases in early visual regions, as well as in the posterior cingulate cortex and precuneus (Fig. 1c), which otherwise showed increased s.d. as part of widespread cortical increases observed during eyes-closed conditions.

Increases in s.d. under psilocybin were partially lateralized to the right hemisphere during eyes-closed rest, meditation and music, and s.d. decreases in sensory regions were observed in the occipital lobe, particularly during music (Fig. 1c). Finally, the maps of s.d. change across the brain in all states were broadly consistent with the maps of GFC change (Fig. 1a), and this redistribution of signal variability is consistent with increased Shannon entropy (see Methods).

### Eyes-closed and eyes-open GFC converge

Complementing the spatial maps of GFC changes, we examined the overall distribution of GFC values across all cortical vertices, independent of their spatial organization. Under psilocybin, the histograms of GFC values in eyes-open and eyes-closed states—which were distinct at baseline (no psilocybin) imaging—largely overlapped (Fig. 1e).

**Extended Data Fig. 4:**
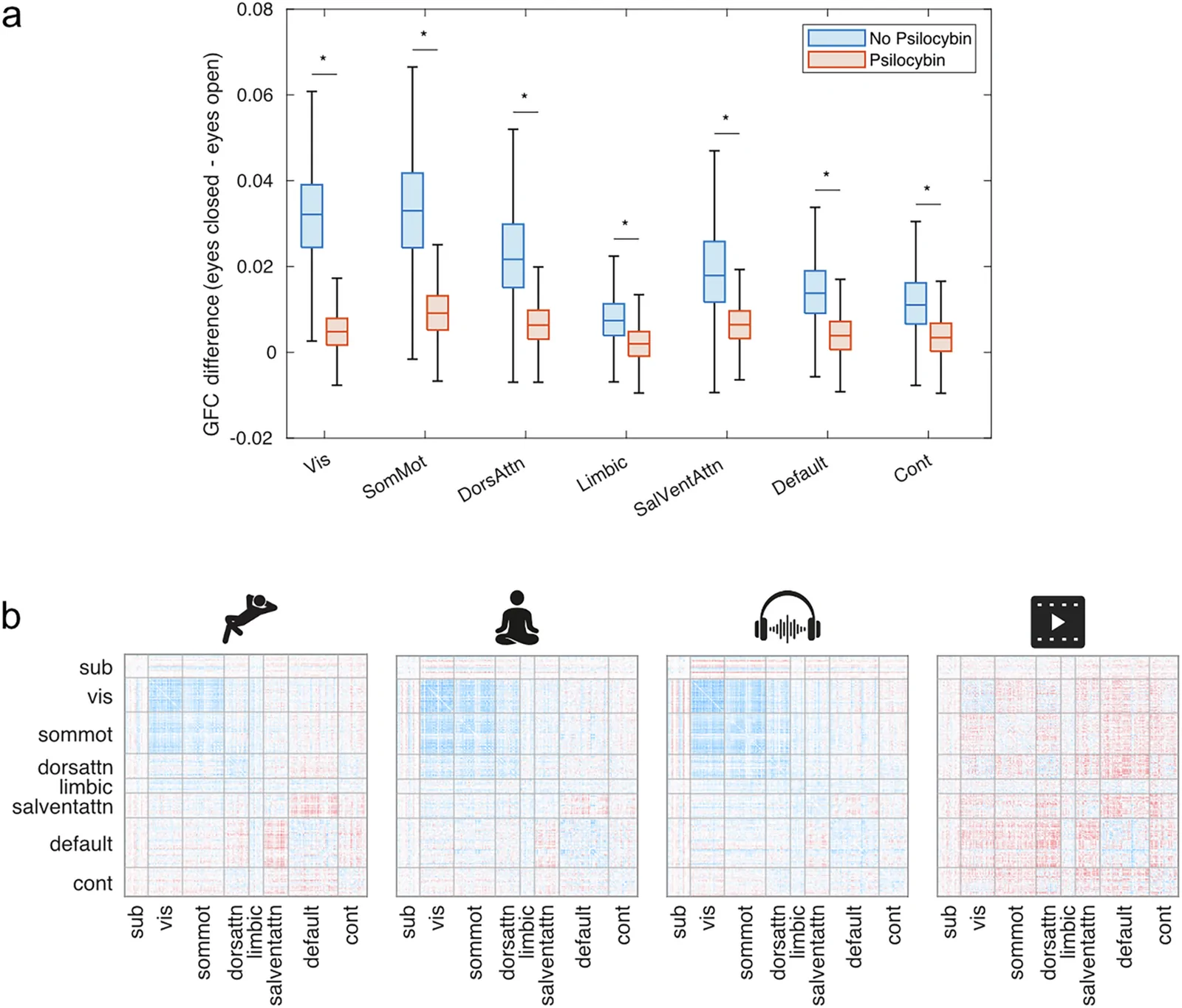
Network-resolved eyes-closed/eyes-open convergence in global functional connectivity and functional connectivity by condition. **a**, The GFC difference between eyes-closed and eyes-open conditions decreased after psilocybin administration. The largest effect was observed in the visual network (−85%, with a Cohen’s *d* effect size of −3.23), but the reduction was larger than −66% and statistically significant in all Schaefer networks (Mann-Whitney U-test, right-tailed, all *P* < 10^−307^). The network names and corresponding sample sizes (number of vertex-level GFC values per network) are: visual (vis, *n* = 28,596), somatomotor (sommot, *n* = 39,570), dorsal-ventral attention (dorsventattn, *n* = 29,697), limbic (*n* = 15,018), salience-ventral attention (salventattn, *n* = 30,174), default mode (*n* = 48,465) and control (cont, *n* = 33,450). The boxes indicate the median and quartiles, and the whisker length is 1.5 times the interquartile range. **b**, Enlarged version of Fig. 1b. Subcortex (sub), visual (vis), somatomotor (sommot), dorsal-ventral attention (dorsventattn), limbic, salience-ventral attention (salventattn), default mode and control (cont). Illustrations in panel **b** of the meditating person by gentle studio/iStock, the resting person by grafikazpazurem/iStock, the headphone by mehmetbuma/iStock and the movie by Intellson/iStock by Getty Images.

This convergence was consistent and statistically significant across sensory, limbic and associative resting-state networks (Fig. 1d and Extended Data Fig. 4a) but particularly pronounced in the visual network, where the gap between eyes-closed and eyes-open GFC values decreased by 85% after psilocybin (Cohen’s *d* = −3.23, *P* < 10^−307^).

### Decreased functional modularity

Functional modularity—the degree to which brain activity is organized into distinct, segregated networks—decreased under psilocybin across all conditions (Fig. 1f; Cohen’s *d* = −0.63, *P* = 6.5 × 10^−5^ for rest; *d* = −0.60, *P* = 1.0 × 10^−4^ for meditation; *d* = −0.70, *P* = 8.8 × 10^−5^ for music; *d* = −0.91, *P* = 2.8 × 10^−6^ for movie; Mann–Whitney *U*-test, right-tailed). Comparable decreases in modularity have also been reported in clinical samples, where lower post-treatment modularity was associated with longer-term symptom improvement following psilocybin therapy^49^. Previous studies have consistently reported disruptions in resting-state network integration and segregation under psychedelics^46,50^, and have primarily analysed DMN connectivity^1,51,52^.

Analysis of our larger dataset revealed that psilocybin-induced reorganization of brain network architecture was primarily attributable to decreased within-network connectivity (Fig. 1b), with significant reductions observed in all networks across eyes-closed conditions (rest, meditation and music), and only in the DMN and dorsal attention network during the eyes-open movie (Supplementary Figs. 2 and 3).

### Context-aligned trajectories emerge

Dimensionality reduction methods have been adopted in neuroscience for uncovering meaningful low-dimensional structures in neural data^53^. In machine learning, an embedding is a learned mapping from high-dimensional data to a low-dimensional representation, expressed as vector coordinates in an embedding space that preserve latent structure^53–55^. Here we used contrastive embeddings for behavioural and neural analysis (CEBRA) to generate low-dimensional embeddings of the preprocessed, frame-by-frame regional BOLD time series and used support vector machine (SVM) classification as a descriptive readout of how well session-specific embeddings distinguished between rest, meditation, music and movie conditions^56^ (see Methods). We then examined the relationship between classification accuracy and subjective experiences assessed via the Mystical Experience Questionnaire (MEQ30)^29,57^, administered at the end of the session.

**Fig. 2:**
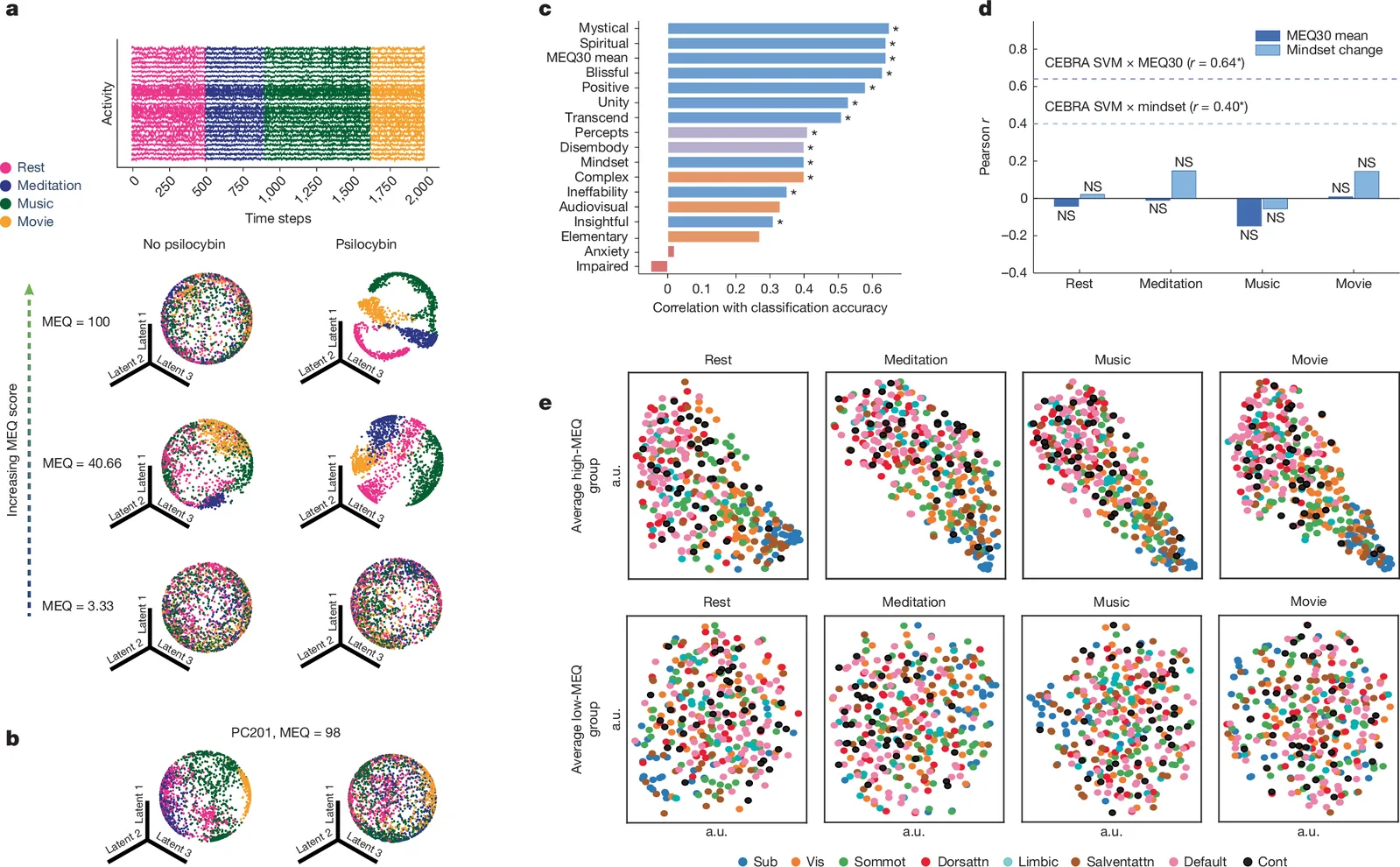
Context-aligned brain trajectories scale with subjective experience under psilocybin. **a**, Using CEBRA, for each participant, we mapped the fMRI time series of all 332 brain parcels at each time point into a three-dimensional space, producing a trajectory spanning the four conditions: rest (pink), meditation (blue), music (green) and movie (yellow). **b**, Network embeddings (CEBRA-derived trajectories) for participant PC201, who reported no subjective effect onset during the MRI session on the day of psilocybin administration (late-onset post-MRI); their embeddings resembled the baseline (no psilocybin) scan. **c**, For the CEBRA trajectory of each participant, a SVM classified the condition label at each time point. Pearson correlation between classification accuracy and acute psilocybin subjective effects (11D-ASC and MEQ30) is shown (left). Positively felt (euphoric) self-dissolving and boundary-dissolving effects (blue), sensory–hallucinogenic effects (orange), negative effects (red) and other (combination) effects (purple) are displayed. The asterisk indicates statistical significance (*P < 0.05; Fig. 4 and Extended Data Fig. 6; Methods). **d**, Pearson correlations between per-participant functional modularity (psilocybin) and two behavioural outcomes (MEQ30 mean, *n* = 54; next-day mindset change, *n* = 53) across the four conditions. Modularity did not predict either outcome (all ∣*r*∣ < 0.15, *P* > 0.28). The dashed lines mark the corresponding CEBRA classification accuracy correlations in the same participants (MEQ30: *r* = 0.64, *P* < 10^−6^; mindset: *r* = 0.40, *P < 0.01). NS, not significant. **e**, A TAVRNN mapped the 332 ROIs into a two-dimensional space. ROIs were projected into a common two-dimensional *t*-distributed stochastic neighbour embedding space and visualized separately for each condition. Average two-dimensional embedding of participants with the highest MEQ scores (80–100; top row), and the lowest MEQ scores (0–20; bottom row) are shown. Each node corresponds to one ROI, colour-coded by brain network. The axes represent abstract latent dimensions in arbitrary units (a.u.) learned by the model and do not correspond to physical coordinates.

Under psilocybin, structured organization emerged in the temporal dynamics of brain activity. Of note, stronger subjective effects were associated with tighter clustering of time point embeddings within the same condition, making them more easily separable from embeddings of other conditions (Fig. 2a). This condition-specific clustering was moderated by the timing of subjective effects, as demonstrated by a participant who reported a late onset of substantial effects but minimal subjective experience during the imaging session (Fig. 2b). Their embeddings resembled baseline (no psilocybin) scans, indicating that the observed neural reorganization covaried with the occurrence and intensity of subjective effects within the imaging window (Fig. 2b).

**Fig. 3:**
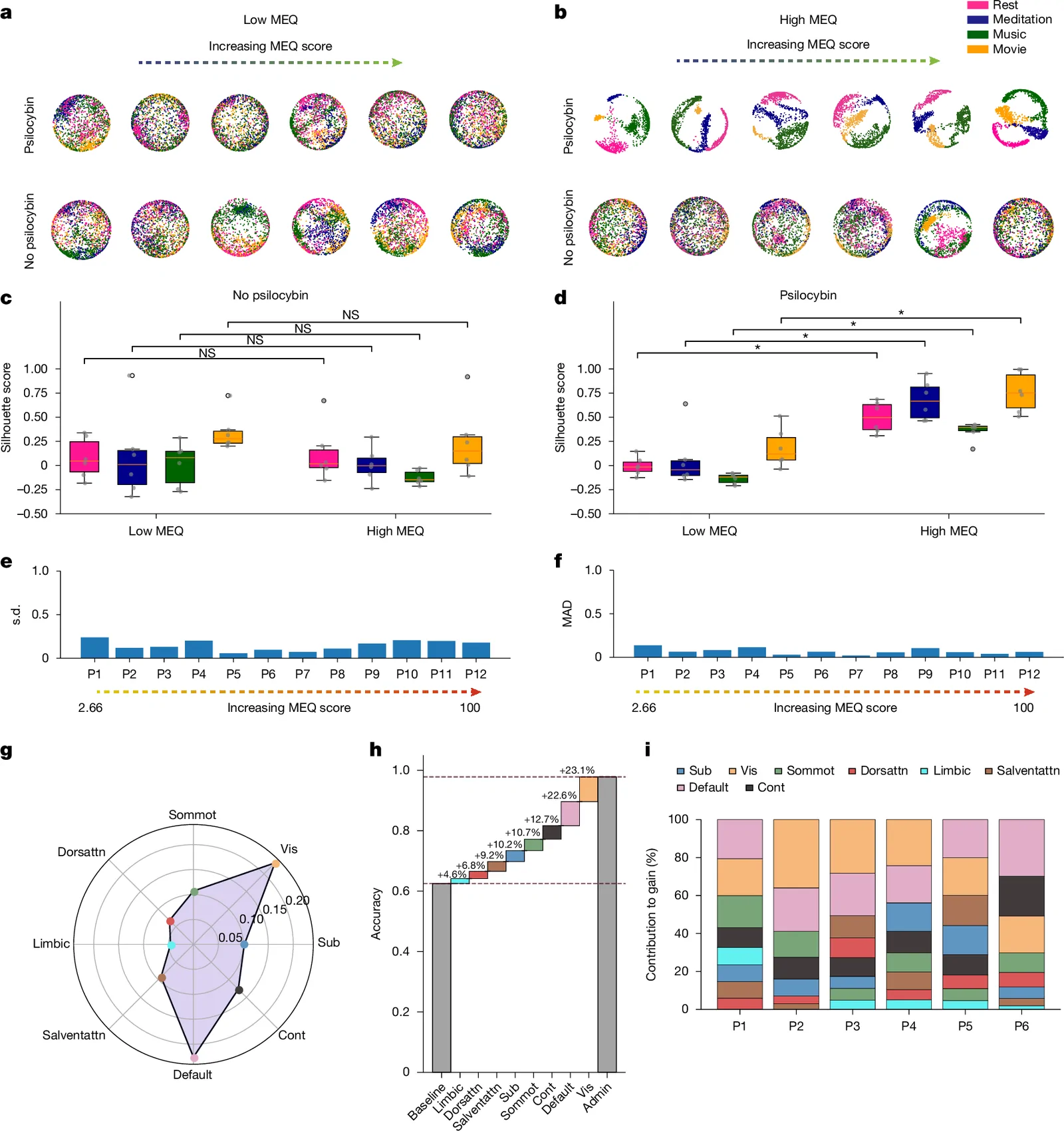
Robustness and anatomical anchoring of context-aligned brain trajectories under psilocybin. **a**,**b**, CEBRA embeddings of *n* = 6 participants with low (**a**) and high (**b**) MEQ scores, ordered by classification accuracy. Psilocybin (top) and no psilocybin (bottom) are shown. **c**,**d**, Silhouette scores quantifying within-condition clustering for the same 12 participants at baseline (no psilocybin; **c**) and under psilocybin (**d**). The boxes indicate the median and quartiles, the whisker length is 1.5 times the interquartile range, and the open circles denote outliers beyond the whiskers (*n* = 6 participants per box). Groups did not differ at baseline (all *P* > 0.19) but differed significantly across all conditions under psilocybin (rest: Δ*s* = 0.51, *P* = 0.0002; meditation: Δ*s* = 0.71, *P* = 0.0023; music: Δ*s* = 0.52, *P* < 0.0001; movie: Δ*s* = 0.63, *P* = 0.0007; Welch’s *t*-test), establishing the gradient as psilocybin-specific. CEBRA training was repeated 10 times per participant. *P < 0.05. **e**,**f**, Run-to-run variability of silhouette scores per participant: mean s.d. (**e**) and median absolute deviation (MAD; **f**) across conditions. Both remain small (s.d. ≈ 0.05–0.25; MAD ≈ 0.03–0.14), confirming clustering stability. P1–12, participant 1–12. **g**–**i**, Network-attributed decomposition from a perturbation analysis (*n* = 6; see Methods): for each participant, the psilocybin time series of one network was replaced with their no-psilocybin dynamics; the drop in classification accuracy was attributed to that network. Radar plot (**g**) of averaged per-network contributions. Waterfall plot (**h**) decomposing the cumulative accuracy gain from baseline (~62%) to psilocybin (~98%); DMN (+22.6%) and visual network (+23.1%) together account for nearly half of the gain, indicating that the gradient end points jointly enable context alignment; dashed lines mark group mean SVM classification accuracy at baseline (lower) and under psilocybin (upper). Per-participant stacked bars (**i**) showing the contribution of each network, demonstrating consistency across individually trained models. See Methods for details.

Further analysis of 12 representative participants, 6 with high MEQ scores and 6 with low MEQ scores, reinforced these findings, revealing a gradient in brain embeddings that scaled with strength of subjective effects (Fig. 3a–d). This gradient reflected a reorganization of neural trajectories under psilocybin that was both context-specific and effect-dependent, suggesting that as subjective effects intensified, context increasingly differentiated neural trajectories into distinct, cohesive patterns.

**Extended Data Fig. 5:**
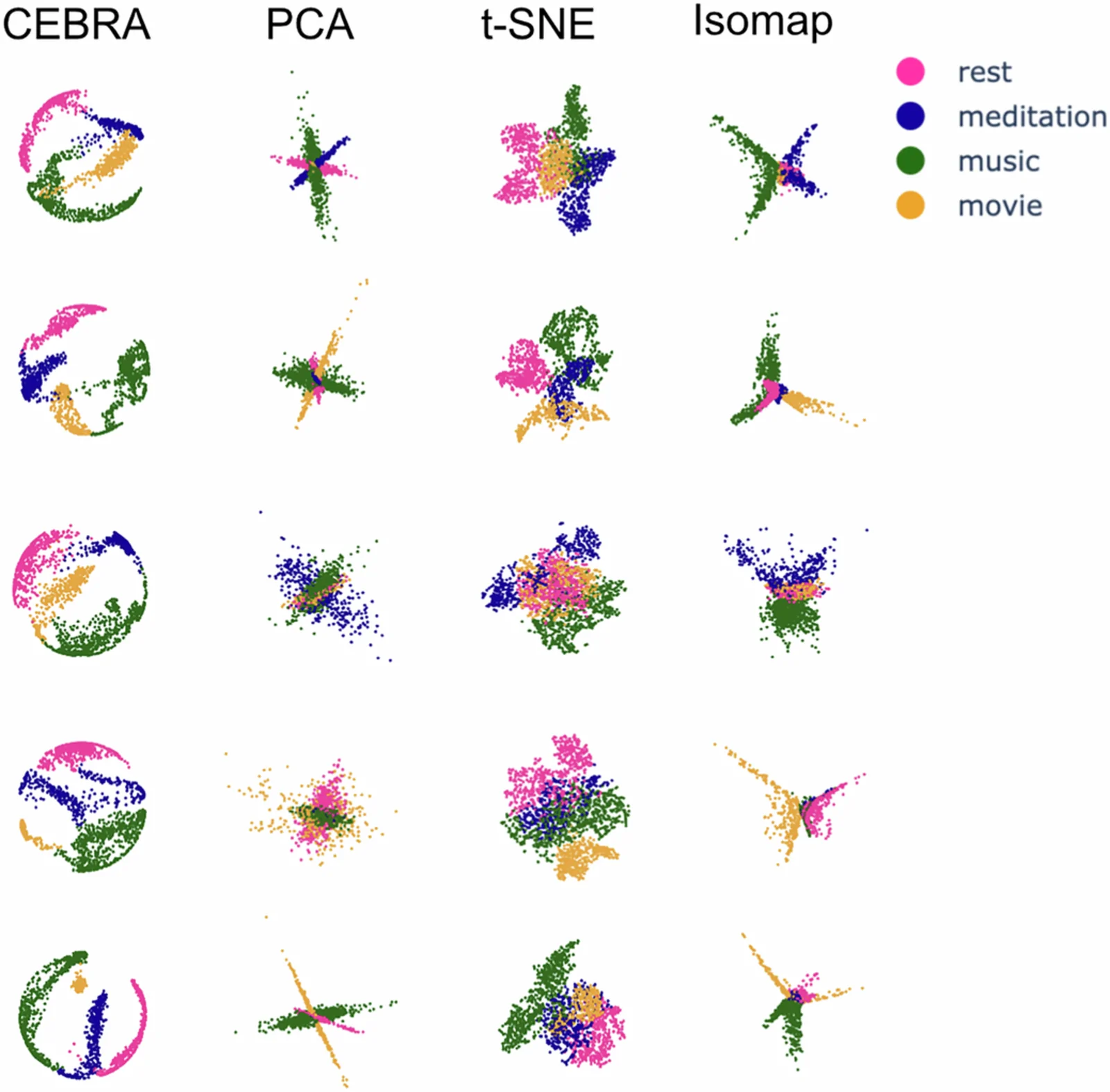
Comparison of CEBRA against PCA, t-SNE and Isomap embeddings. Low-dimensional embeddings of psilocybin fMRI data from high-MEQ participants using four dimensionality reduction methods. Each dot corresponds to a time point representing the activity of our 332 ROIs, colored by condition (rest, meditation, music, movie). Principal component analysis (PCA) shows limited separation due to its linear nature, while t-distributed stochastic neighbour embedding (t-SNE) and Isomap achieve clearer separation but ignore temporal continuity, leading to fragmented embeddings. CEBRA preserves temporal adjacency, producing smooth trajectories that reveal both separation between conditions and natural transitions across brain states. Despite methodological differences, embedding structure shows a generalizable pattern across dimensionality reduction approaches.

To assess the robustness of these effects, we compared CEBRA embeddings with three common dimensionality reduction methods (principal component analysis, *t*-distributed stochastic neighbour embedding and Isomap) in participants with high MEQ. Despite methodological differences, each approach recovered the context-dependent embedding structure (Extended Data Fig. 5), confirming that the observed organization was not specific to CEBRA.

**Extended Data Fig. 6:**
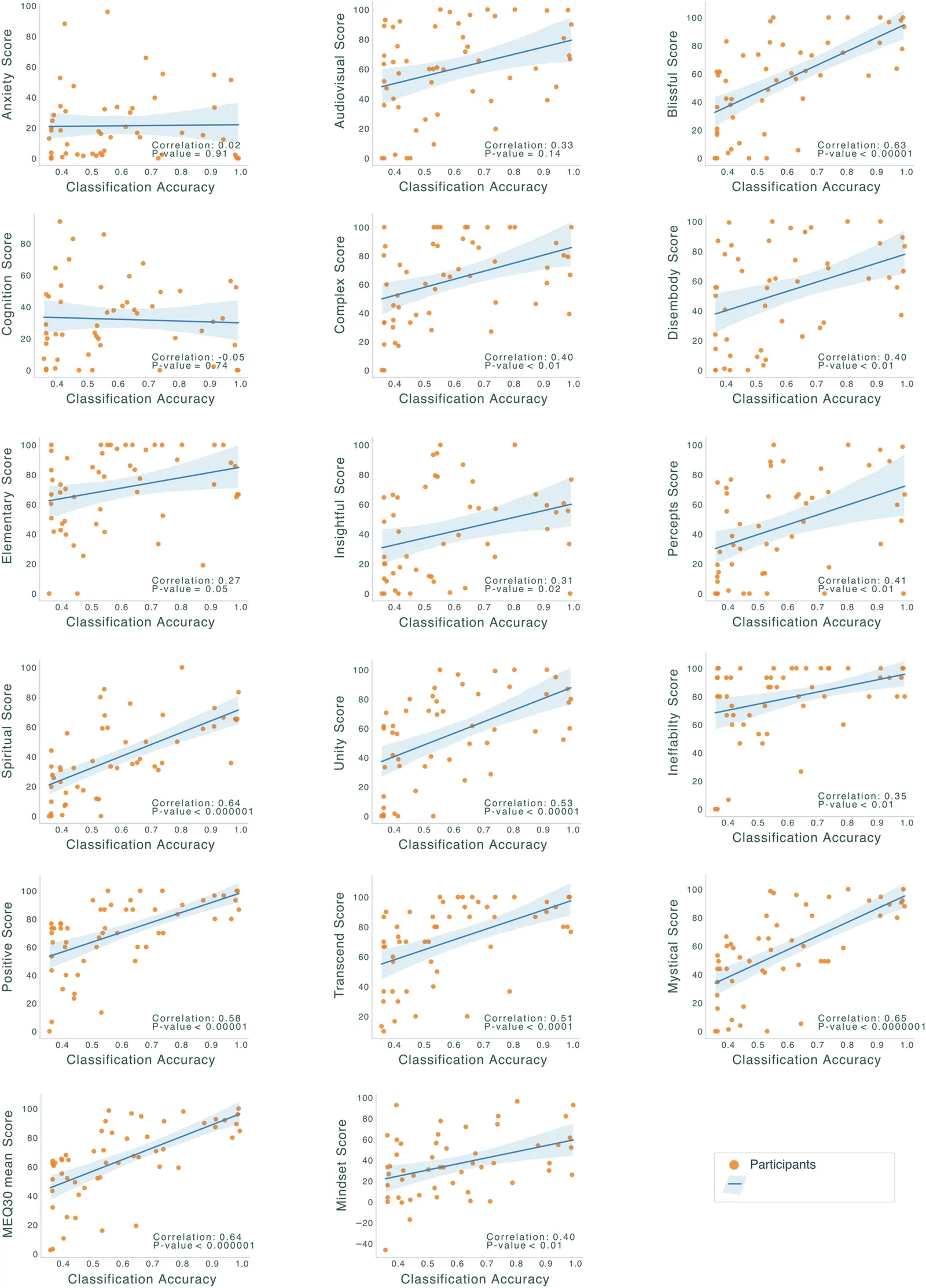
CEBRA-SVM classification accuracy correlates with 11D-ASC, MEQ30 and mindset change. For the CEBRA trajectory of each participant a support vector machine (SVM) was used to classify the correct condition label at each time point (*n* = 54). The figure presents the Pearson correlation between classification accuracy and participants’ 11D-ASC, MEQ30 and next-day mindset change scores, reporting the correlation coefficient and statistical significance level (P-value ≤ 0.05, uncorrected). The blue solid line represents the regression fit, with the shaded region indicating the 95% confidence interval.

**Extended Data Fig. 7:**
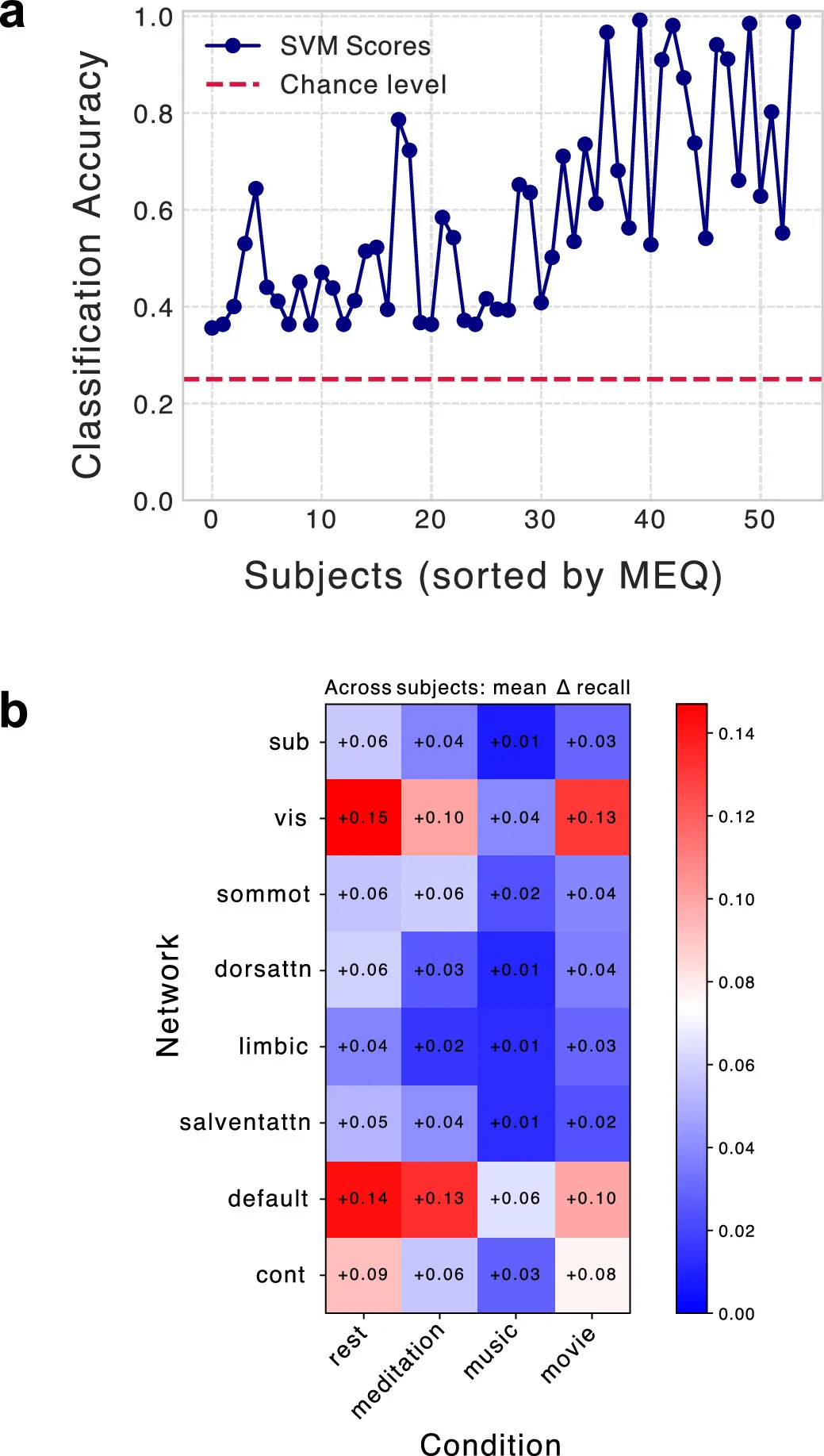
Per-participant classification accuracy and per-condition network attribution. **a**, Classification accuracy from the CEBRA-SVM analysis for each participant, ordered by ascending MEQ score. Red dashed line indicates the chance level (0.25). All participants exceed chance, and accuracy scales with subjective experience intensity (*r* = 0.64, *P* < 10^−6^ for MEQ30 mean; see Fig. 2c). **b**, Per-condition recall decomposition from the network perturbation analysis (Methods): heatmap shows the across-participant mean change in per-condition recall (*Δ* recall = recall_psilocybin_  − recall_substituted_) for each network (rows) and condition (columns). Default mode and visual networks show the largest effects across all four conditions, with graded modulation rather than condition-specific dissociation, indicating that the joint alteration of the systems at both gradient end points supports context alignment uniformly across rest, meditation, music and movie.

To quantify the degree of this organization, we classified the condition label at each time point for each participant with a SVM classifier on the CEBRA-derived trajectories. We examined the correlation between classification accuracy and each of the 11-Dimension Altered States of Consciousness (11D-ASC)^58^ and MEQ30 scores, after psilocybin administration. Classification accuracy was most strongly associated with positively felt self-dissolving and boundary-dissolving effects, followed by weaker correlations with sensory-hallucinogenic effects, and low or negative correlations with anxiety and impaired control and cognition (Fig. 2c and Extended Data Fig. 6). We also found a correlation between classification accuracy and mindset change 1 day after psilocybin (Extended Data Fig. 6, Supplementary Table 1 and Supplementary Fig. 4). Participants with higher MEQ30 mean scores generally exhibited higher classification accuracy, indicating that the context-specific organization of neural dynamics scales with the intensity of subjective experience (Extended Data Fig. 7a). We refer to this structured alignment of neural dynamics with experiential context as context alignment. Of note, these brain–behaviour associations depended on preserving temporal dynamics: per-participant functional modularity—a time-averaged measure of network segregation that, despite robust group-level reductions under psilocybin (Fig. 1f; Cohen’s *d* = −0.60 to −0.91)—was not significantly associated with MEQ30 mean or next-day mindset change in any condition (Fig. 2d).

A network perturbation analysis (see Methods) revealed that this context-aligned organization is globally distributed but depends most strongly on altered dynamics in the DMN and the visual network (approximately 6–8 percentage point accuracy reduction each when their psilocybin-induced dynamics are replaced with baseline; Fig. 3g,h)—the two systems that anchor opposite poles of the principal cortical gradient from internal to external processing^34^. This dependence was uniform across all four conditions rather than condition-specific (Extended Data Fig. 7b), indicating that the joint alteration of the systems at both gradient end points sets the conditions for brain activity to align with context. This convergence held across individually trained models (Fig. 3i), providing anatomical grounding for how psilocybin reorganizes neural trajectories into structured patterns that reflect context. Crucially, if the observed effect merely increased random noise, trajectories would become diffuse and overlapping, degrading classification accuracy. The opposite was observed. Participants reporting stronger effects showed tighter clustering (higher silhouette scores; Fig. 3d) and higher accuracy, indicating that this reorganization was structured rather than random. No structured embedding emerged at baseline or with low MEQ, and clustering was already strong during rest (the first condition acquired), before any sequence could accrue, indicating that the low-dimensional organization is unlikely to be driven by the fixed order of conditions (see Supplementary Information for condition order considerations).

These findings were independently supported using another machine learning-based technique called temporal attention-enhanced variational graph recurrent neural network (TAVRNN)^59^, which captured a lower-dimensional representation of individual network connectivity during each condition. These two-dimensional maps represent abstract latent dimensions learned from brain functional connectivity patterns, not physical brain coordinates, with each point corresponding to one region of interest (ROI) placed closer to others when their connectivity evolves similarly over time. TAVRNN captures temporal changes in network structure by modelling sequential snapshots of brain connectivity, enabling the identification of key connectivity patterns and shifts in communicability (see Methods for TAVRNN details).

**Extended Data Fig. 8:**
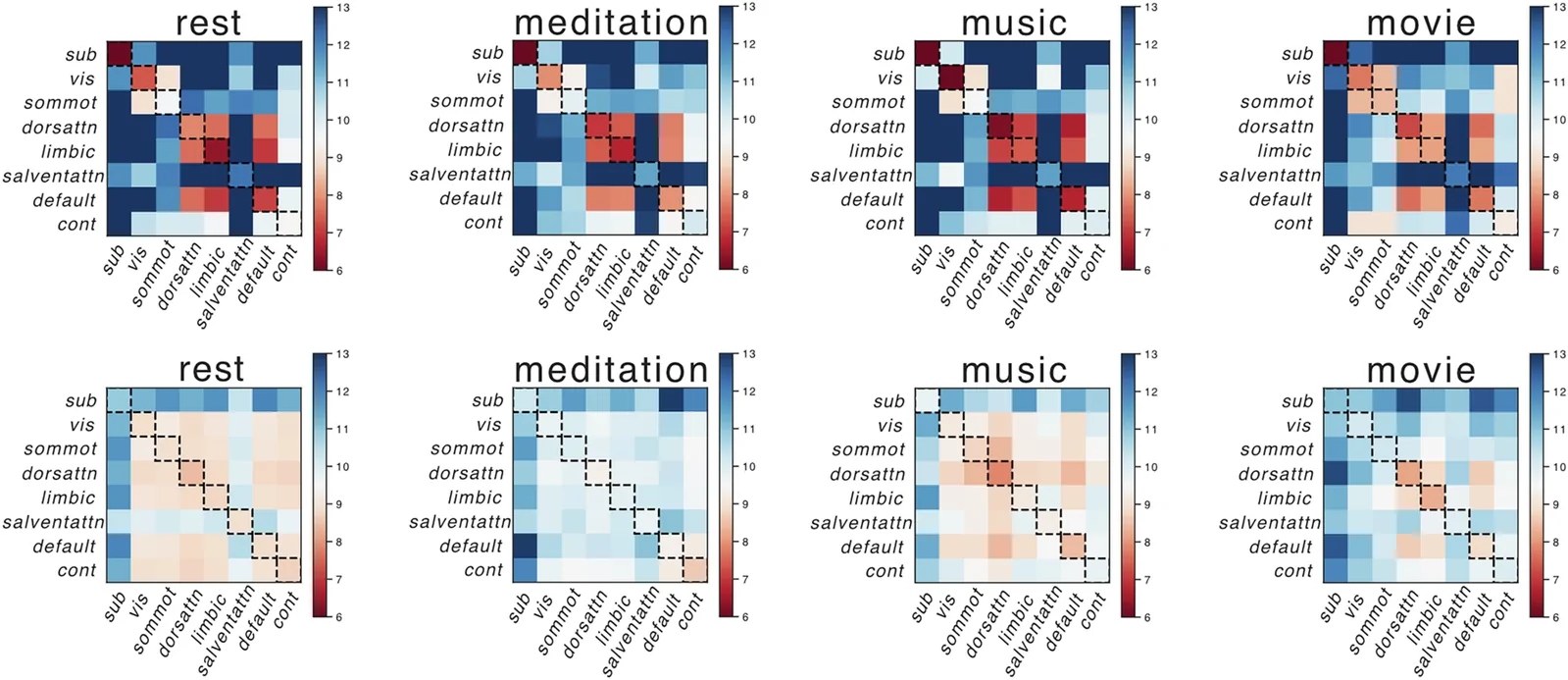
Within-network ROI clustering in TAVRNN embeddings. Heatmaps showing the average Euclidean distance between pairs of ROIs within each brain network in the TAVRNN embedding space across the four cognitive conditions: rest, meditation, music and movie. The top row represents the heatmaps for participants with the top 5 MEQ scores, and the bottom row represents the heatmaps for participants with the lowest 5 MEQ scores. For all conditions, the diagonal of the top row exhibits lower values, indicating more densely clustered embeddings (ROIs positioned closer to each other) within the same network compared to the bottom row, which suggests a greater spread of ROIs in participants with lower MEQ scores. This highlights that network nodes are more closely clustered in the embedding space for participants with high reported subjective effects and this pattern is observed across all conditions, indicating a generalized effect of psilocybin on network compactness.

TAVRNN revealed two distinct patterns under psilocybin. Nodes within individual networks exhibited tighter clustering, reflecting more cohesive within-network dynamics (Extended Data Fig. 8 and Supplementary Information). Simultaneously, embeddings across all networks showed tighter clustering, indicating a shift towards more integrated brain-wide organization and increased global cohesion across contexts. This dual pattern of local within-network cohesion alongside global reorganization scaled with subjective effects, suggesting that the increased within-network and between-network cohesion observed under psilocybin depended on the strength of subjective experience and was broadly preserved across contexts (Fig. 2e).

### Structure of psychedelic phenomenology

**Fig. 4:**
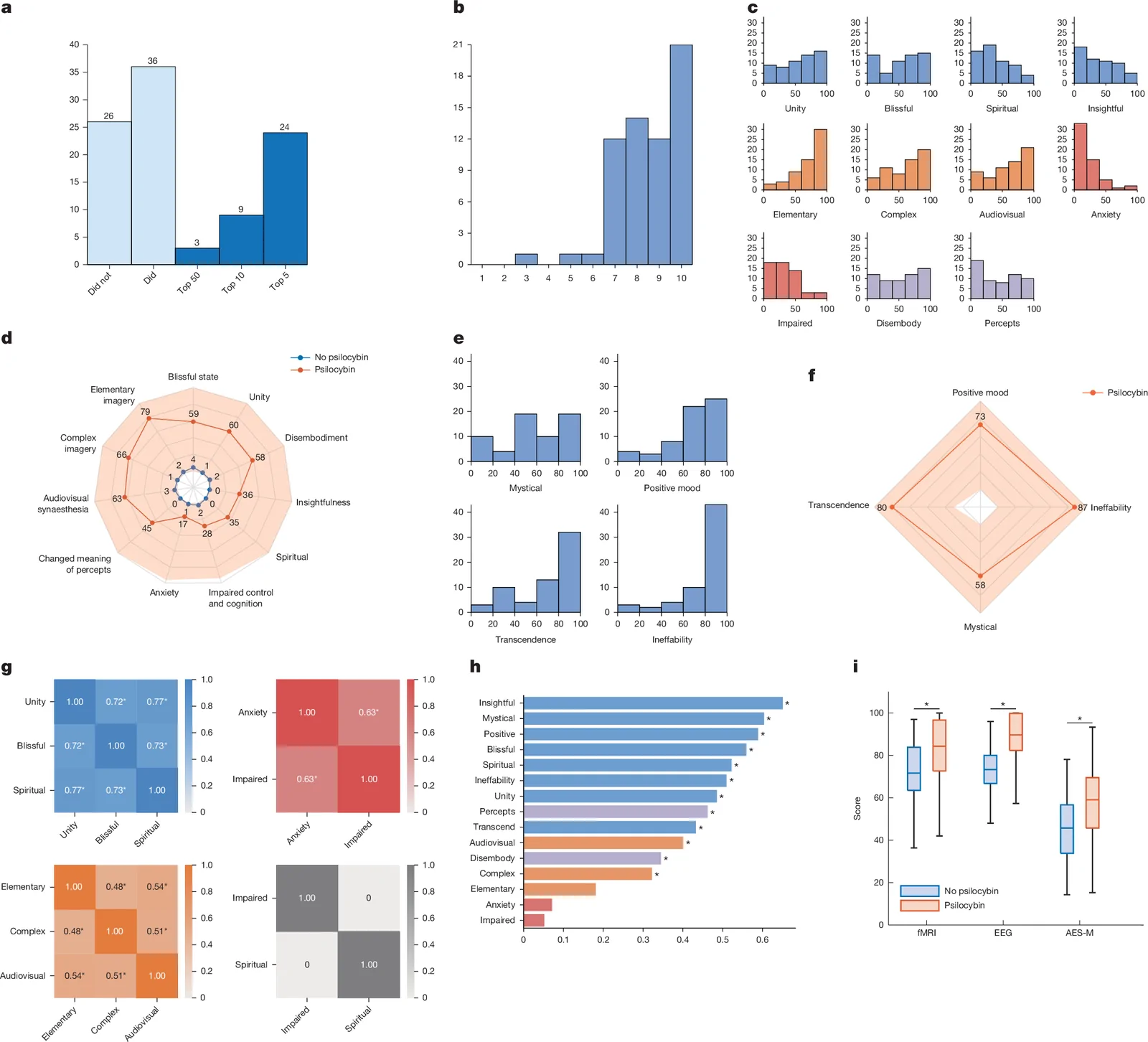
Phenomenological effects and music experience following psilocybin. **a**, Distribution of meaningfulness ratings (see Methods). The first two bars represent participants who did (second) and did not (first) rate the experience as personally or spiritually meaningful; the next three bars indicate the number rating it in the top 50, top 10 and top 5 most meaningful life experiences, respectively. See Methods for expanded range. **b**, Histogram of intensity scores from ‘how intense would you rate the psilocybin experience?’ reported the day after psilocybin. **c**, Histograms of 11D-ASC scores. The colours indicate theoretical distinctions: positively felt associative effects (blue); sensory–hallucinogenic effects (orange); negative effects (anxiety and impaired control and cognition; red); and other (combination) effects (purple). **d**, Radar plot of 11D-ASC: mean scores across participants after psilocybin (solid red) and at baseline (solid blue), with the minimum–maximum range shaded. **e**, Histograms of MEQ30 scores, composed of three positively felt associative subdimensions and one valence-neutral (ineffability) subdimension. **f**, Radar plot of MEQ30 mean scores with the minimum–maximum range shaded. **g**, Associations between 11D-ASC effects show strong correlations among positively felt (blue) and sensory–hallucinogenic (orange) and negative (red) groupings. Grey marks paired subscales from different groupings, shown to indicate the absence of association between them. Impaired control and cognition showed no correlation with spiritual experience. The asterisks denote correlations significant after Bonferroni correction (*P < 0.001) across the 105 unique pairs (*α* = 4.76 × 10^−4^; two-sided Pearson). See Extended Data Fig. 9 for the complete 11D-ASC–MEQ pairwise correlation matrix and respective *n* values. **h**, Correlations between averaged next-day mindset change and acute psilocybin subjective effects (11D-ASC and MEQ30). *P < 0.05, two-sided Pearson, uncorrected; exact *P* values and per-subscale *n* are given in Methods. See Supplementary Information for mindset score dimensions. **i**, Music experience scores (see Methods) during EEG and MRI at baseline and under psilocybin, and AES-M scores comparing aesthetic music experience at baseline and under psilocybin. The asterisks (*P < 0.001) show Mann–Whitney *U*-test, two-tailed (*n* = 55 for all boxplots). The boxes show the median and quartiles, and the whiskers are 1.5 times the interquartile range.

Participants enrolled in the study had no previous psychedelic experience (see Methods). Reports 1 day after psilocybin confirmed that their experiences during imaging were profoundly altered and meaningful. Half of the participants retrospectively ranked it among the most meaningful experiences of their lives (Fig. 4a), and most reported it as substantially intense (at least 9 out of 10; Fig. 4b). Semi-structured reports included accounts that described the experience as ‘one of the most peaceful and profound things I could ever experience’, ‘...meld[ing] with the MRI machine, floor, walls, air’, ‘los[ing] the plot of who I was, where I was, if I was even here, what was happening’ and ‘los[ing] all sense of self... becom[ing] at one with all my surroundings...as part of a bigger network of things’ (see Supplementary Information for tables of qualitative excerpts).

Our large sample enabled a view of within-group subjective reports given a standard 19 mg dose of psilocybin. 11D-ASC and MEQ30 scores illustrate substantial group-level intensity across key scales alongside wide individual variability (Fig. 4c–f). Subscales were grouped to reflect key conceptual distinctions in psychedelic effects (colour-coded in Fig. 4c). Positively felt self-dissolving and boundary-dissolving effects such as bliss and unity received higher ratings across our sample than cognitive insights or spiritual experiences. Negative effects, particularly anxiety, were infrequent or rated as low.

**Extended Data Fig. 9:**
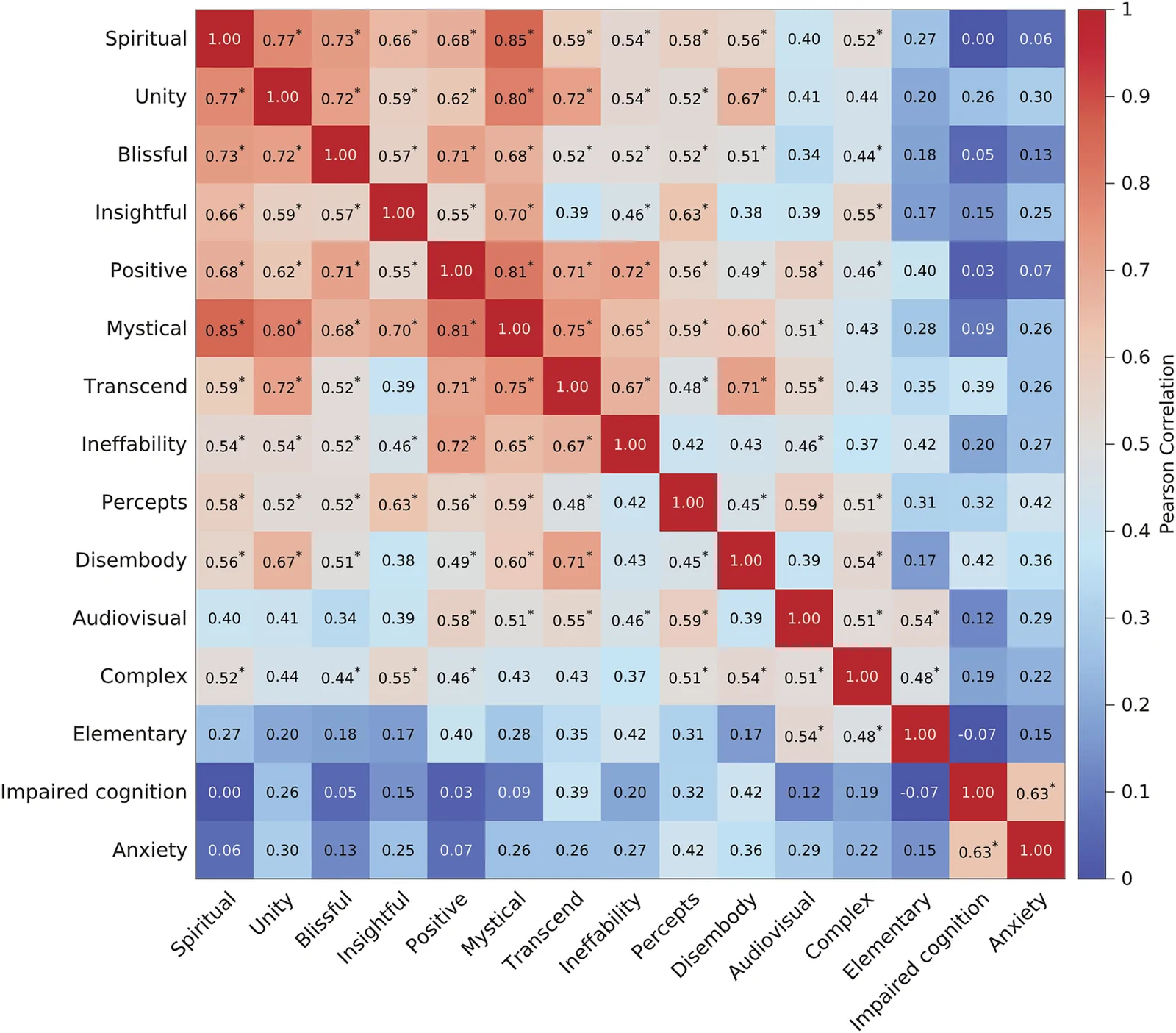
Correlation matrix of subjective-effect subscales. Pairwise Pearson correlations between the four MEQ30 subdimensions (Mystical, Positive, Transcend, Ineffability) and the eleven 11D-ASC subdimensions measured the day of psilocybin administration, computed via pairwise complete-case analysis. Per-subscale sample sizes: Spiritual *n* = 59; Unity *n* = 58; Blissful *n* = 59; Insightful *n* = 56; Positive *n* = 62; Mystical *n* = 62; Transcend *n* = 62; Ineffability *n* = 62; Percepts *n* = 58; Disembody *n* = 57; Audiovisual *n* = 61; Complex *n* = 60; Elementary *n* = 61; Impaired Cognition *n* = 56; Anxiety *n* = 56. Pairwise *n* per cell equals the intersection of the two contributing subscale samples (range 54–62, median 57). Cell text shows the Pearson correlation coefficient. Asterisks denote correlations significant after Bonferroni correction across the 105 unique off-diagonal pairs (*α* = 0.05/105 = 4.76 × 10^−4^; two-sided Pearson); 56 of 105 pairs survived correction. Color bar: Pearson *r*, with red indicating high correlations and blue indicating low or near-zero correlations.

These subscales were analysed post hoc to assess patterns of covariation^60^, identifying groups of subscales that showed high intercorrelations in our sample (Fig. 4g and Extended Data Fig. 9).

The Life Attitudes Profile Revised^61^, used to assess death acceptance and changes in personal meaning, and Nature Relatedness Scale^62^ were measured before and 1 month after psilocybin administration. Results showed group-level improvements in death acceptance (*t*(54) = −3.66, *P* = 0.0006, *d* = 0.49), personal meaning (*t*(54) = −3.77, *P* = 0.0004, *d* = 0.51) and nature relatedness (*t*(55) = −3.37, *P* = 0.0014, *d* = 0.26), alongside individual variability (Supplementary Figs. 5 and 6).

Intensity scores on blissful state and unity matched those of higher-dose studies (300–400 μg kg^−1^)^63^, suggesting that our supportive context (space design, minimized interruptions, immersive music and inward focus) facilitated the depth of the subjective experience.

### Acute experience tracks mindset change

We examined the relationship between the post-psilocybin mindset change score (measured 1 day after administration; see Supplementary Information) for each participant and their subjective experience scores from the 11D-ASC scale and the MEQ30. Mindset change, assessed across dimensions such as connection to self, others and nature, as well as patience, harmony and inner peace, correlated moderately with associative dimensions of the psychedelic experience. Insightfulness showed the strongest correlation (*r* = 0.65, *P* = 9.9 × 10^−8^), followed by mystical, positive, blissful and spiritual sub-dimensions (*r* = 0.52–0.60, all *P* < 10^−4^). Sensory–hallucinogenic effects were less correlated (*r* = 0.18–0.46), with four of five subscales reaching significance (*P* < 0.05). Negative experiences showed minimal associations (*r* < 0.1, not statistically significant; Fig. 4h). These findings suggest that positively felt associative dimensions (self-dissolving and boundary-dissolving effects) are the primary drivers of psychological shifts, with cognitive insights and transcendent states, such as unitive, blissful, mystical and spiritual experiences, contributing more to mindset change than sensory–hallucinogenic or negative effects (anxiety and impaired control and cognition). The observed relationship between subjective experience and psychological shifts provides empirical context for ongoing debates about the necessity of the psychedelic experience for clinical efficacy^64–66^.

### Context directs hippocampus–DMN coupling

**Fig. 5:**
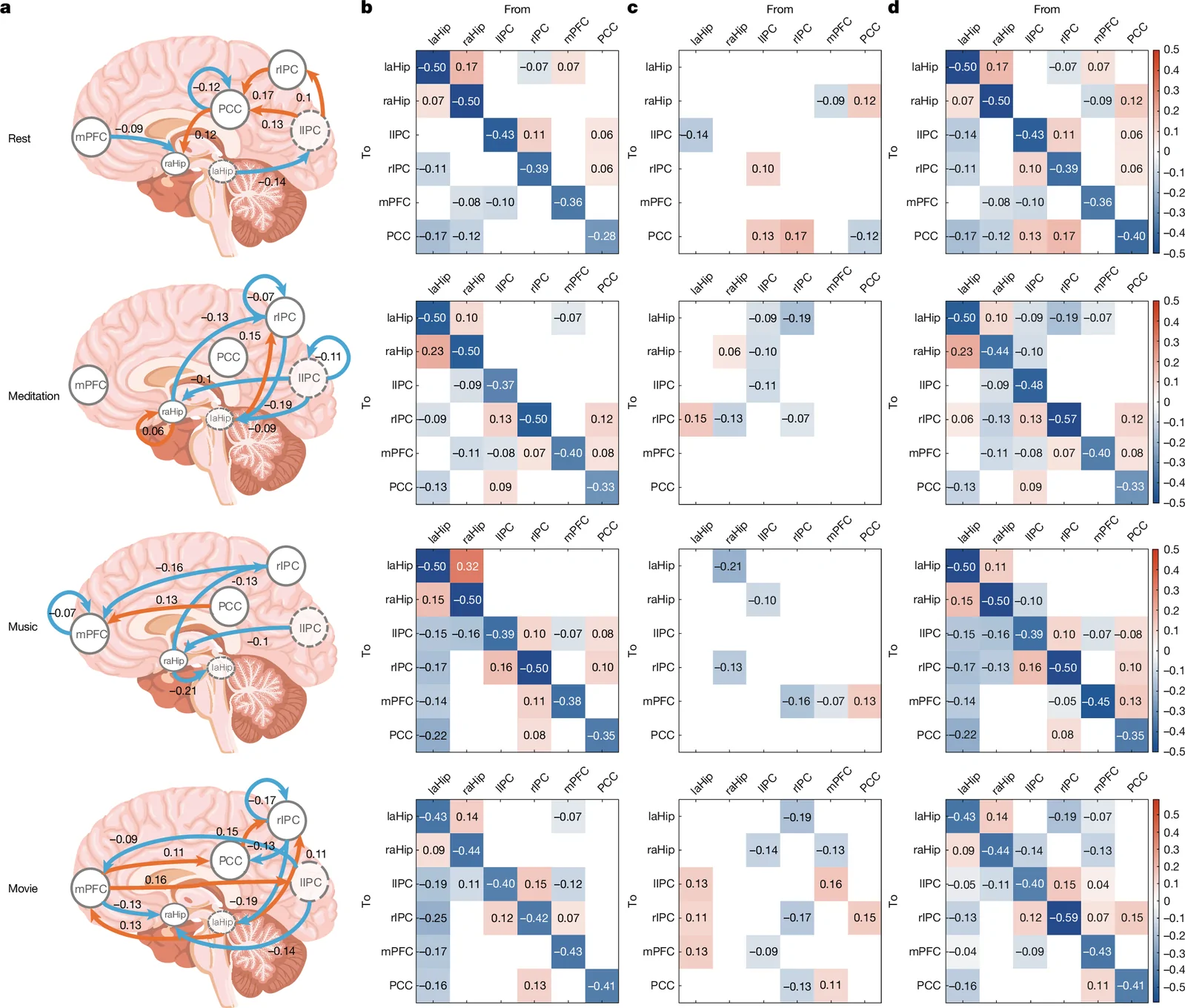
Model of effective connectivity changes from baseline (no psilocybin) to psilocybin. The brain regions included: left aHip (laHip), right aHip (raHip), left inferior parietal cortex (lIPC), right inferior parietal cortex (rIPC), medial prefrontal cortex (mPFC) and posterior cingulate cortex (PCC). Scan sequences (from top to bottom): rest, meditation, music and movie watching. The model was specified as fully connected, allowing all possible causal interactions between regions. **a**, Spatial mapping of ROIs with estimated changes in effective connectivity. Red shades denote increases and blue shades denote decreases (Hz), based on posterior means averaged across participants using Bayesian model averaging. **b**, Mean effective connectivity at baseline. For mean effective connectivity, the warm colours represent excitation and the cool colours represent inhibition. **c**, Matrix of effective connectivity changes from baseline to psilocybin, corresponding to panel **a**. **d**, Mean effective connectivity under psilocybin. Connection strengths (posterior expectations) are reported in Hz. Self-connections are not log-scaled. See Supplementary Information for tables of posterior expectations and credible intervals, and further details. Displayed connections have a posterior probability > 0.99, indicating very strong evidence. Illustration of brain in panel **a** by Kristina Bulgakova/iStock by Getty Images.

Dynamic causal modelling (DCM)^67–69^ estimated effective connectivity between the aHip and core DMN nodes across rest, meditation, music and eyes-open movie, contrasting psilocybin and baseline (no psilocybin) conditions (Fig. 5). Group parametric empirical Bayes contrasts revealed condition-specific changes in effective connectivity. Eyes-closed conditions showed modest changes, whereas the movie condition exhibited the largest number and magnitude of changes. The total strength of between-region parameter changes (sum of absolute changes with posterior probability ≥ 0.99) was: rest ≈ 0.75 Hz, meditation ≈ 0.66 Hz, music ≈ 0.73 Hz and movie ≈ 1.48 Hz (largest). The greater magnitude of effective connectivity changes during movie indicates context-dependent reorganization within the DMN. See Supplementary Tables 2–5 for parameter estimates and credible intervals, and Supplementary Information for condition-level interpretation.

### Meditation training and music context

**Extended Data Fig. 10:**
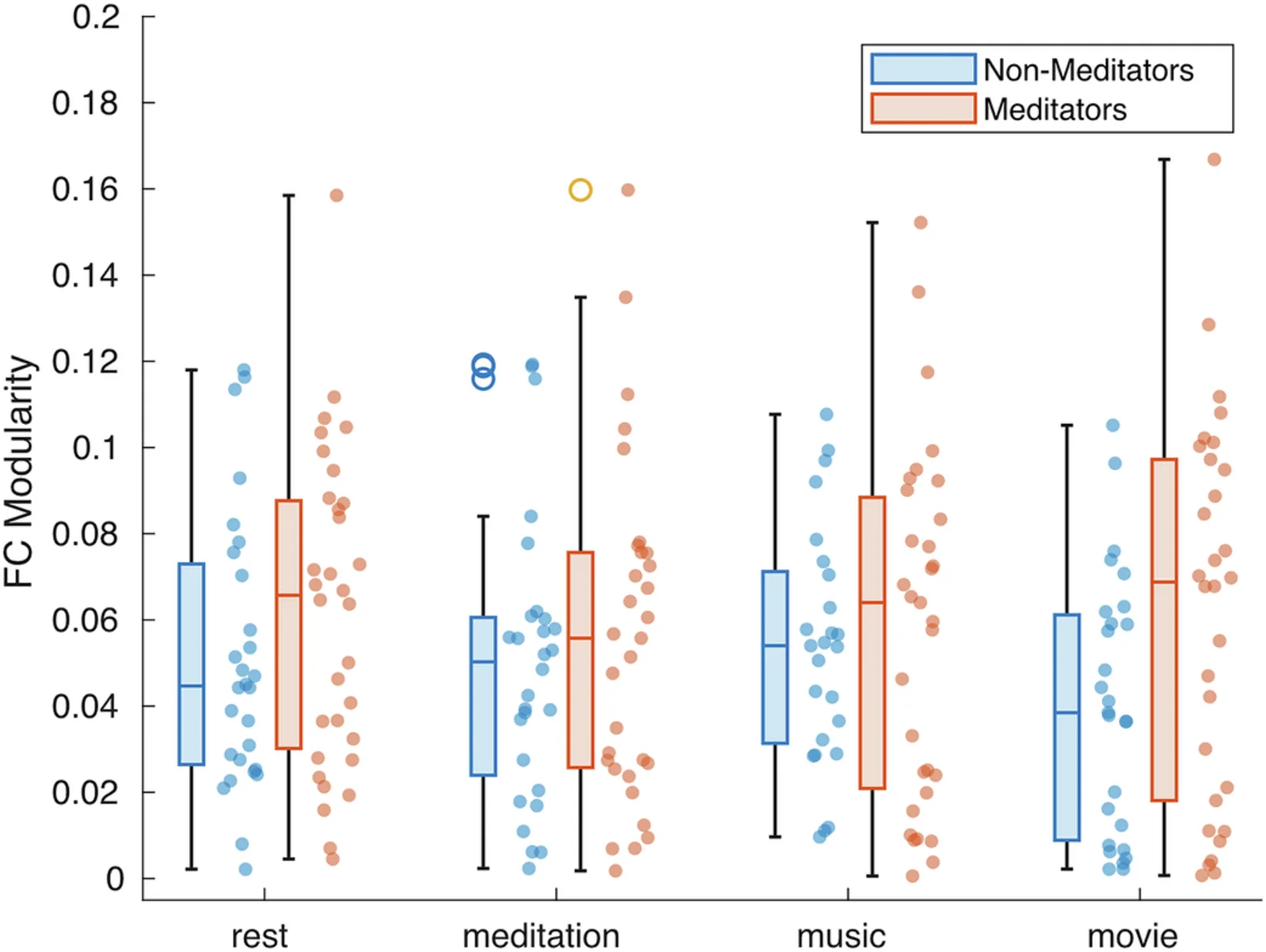
Mindfulness meditation training had no effect on functional modularity under psilocybin. Per-participant functional modularity under psilocybin is shown separately for participants who completed the 8-week mindfulness meditation training (meditators; *n* = 32) and those who did not (non-meditators; *n* = 28), across the four scan conditions (rest, meditation, music, movie). Each dot represents one participant; horizontal lines indicate the group median, and whiskers the interquartile range. No comparison between meditators and non-meditators reached statistical significance (two-sided Mann–Whitney *U* test; rest *P* = 0.289, meditation *P* = 0.590, music *P* = 0.792, movie *P* = 0.054, uncorrected; Bonferroni-corrected across the four condition-wise tests at *α* = 0.0125, none significant). The two groups were pooled for all other analyses reported in the main text.

We assigned half of the participants to an 8-week mindfulness-based cognitive therapy programme (Extended Data Fig. 1; see Methods). No statistically significant differences emerged between meditators and non-meditators in acute psilocybin effects (11D-ASC and MEQ30) or connectivity metrics under psilocybin (for example, functional modularity; Extended Data Fig. 10). Previous reports of meditation–psilocybin synergy have typically involved experienced practitioners in intensive retreat-based practice^70,71^, whereas brief structured training did not produce detectable group-level effects in analyses of our sample.

Music, a contextual element known to shape subjective experiences under psychedelics^72^, had a central role in the study design (see Supplementary Information). Participants rated the ethereal music as significantly more emotionally resonant and engaging under psilocybin during MRI and EEG sequences (fMRI: +16%, Cohen’s *d* = 0.67, *P* = 6.3 × 10^−4^; EEG: +21%, Cohen’s *d* = 1.08, *P* = 3.2 × 10^−7^). This was independently confirmed across the administration day on the Aesthetic Experience Scale–Music (AES-M; +25%, Cohen’s *d* = 0.75, *P* = 2.4 × 10^−4^; Fig. 4i; see Methods for details). Moreover, TAVRNN embeddings showed the greatest global network organization during music (Fig. 2e).

### Context modulates power and complexity

**Fig. 6:**
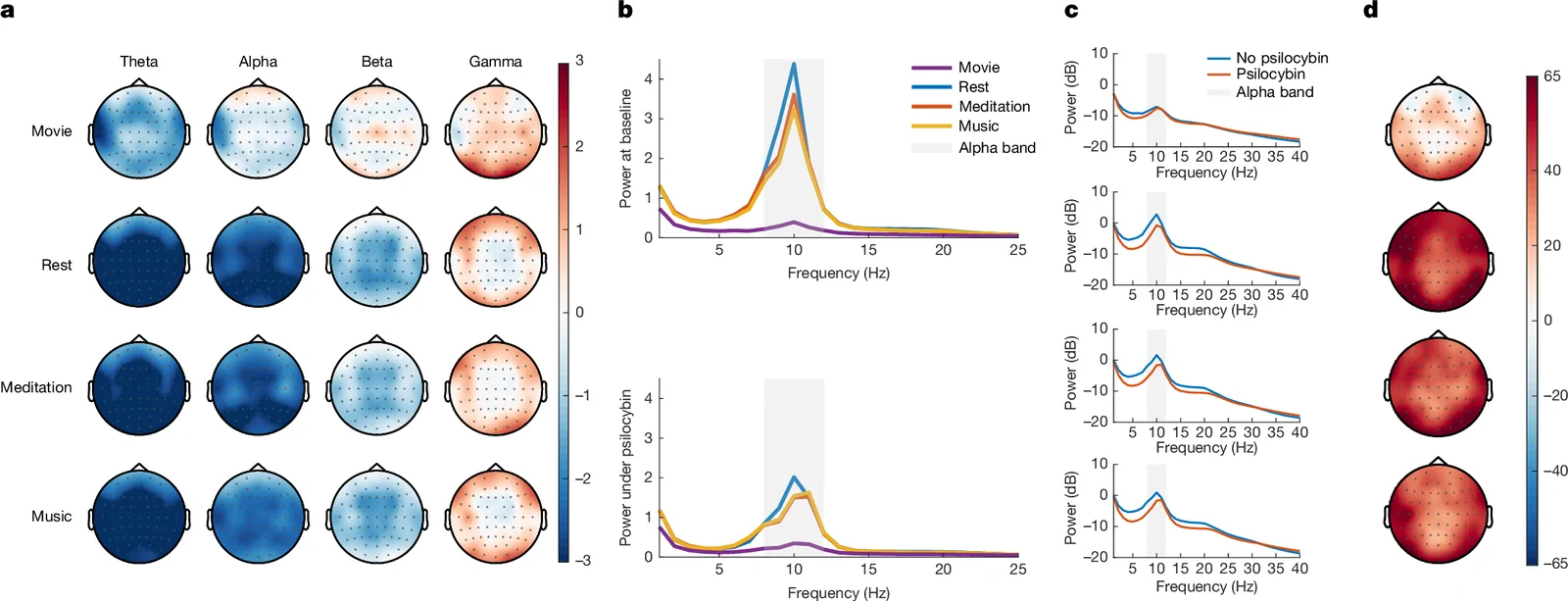
EEG spectral and complexity changes show reduced eyes-open versus eyes-closed separation under psilocybin. **a**, Spatial difference in power (psilocybin minus baseline; dB units) for each frequency band. **b**, Group-averaged power spectra for each condition at baseline (top) and under psilocybin (bottom). Under psilocybin, all eyes-closed conditions (rest, meditation and music) showed broad-band reductions in theta, alpha and beta power (meditation and music overlap; rest modestly differed), whereas the eyes-open movie spectrum was relatively unchanged. **c**, Group-averaged power spectra plotted separately for each condition, to compare psilocybin versus no psilocybin. **d**, Lempel–Ziv complexity (psilocybin minus baseline) showed similar values across eyes-closed conditions and reduced complexity during the eyes-open movie.

Context-sensitive psilocybin-induced brain dynamics observed using MRI were confirmed using 64-channel wet EEG, in both the power spectrum and the signal complexity. Our findings extend preclinical and human observations of desynchronized local brain activity under psilocybin^5,73^ by demonstrating that these alterations are modulated by sensory context. We found that psilocybin expanded the power spectrum, with decreases in theta, alpha and, to a lesser extent, beta power, accompanied by modest gamma increases during eyes-closed conditions, concentrated in frontal regions. These became attenuated during movie, with increases localized to early visual areas, indicating condition-dependent topography (Fig. 6a). This attenuation in power and complexity during movie, relative to eyes-closed conditions, is consistent with reduced network-level disruption during externally directed attention, as previously shown using fMRI functional connectivity^6^. Meditation and music recordings aligned in EEG, showing similar power profiles across frequencies at baseline and under psilocybin, converging despite distinct stimulus properties, whereas rest modestly differed (Fig. 6b). Psilocybin also reduced the difference between eyes-open and eyes-closed alpha power by 48% (Cohen’s *d* = −0.79, Mann–Whitney *U*-test right-tailed *P* < 10^−19^), suggesting an integration of internally and externally focused neural processing (Fig. 6b,c and Supplementary Fig. 7).

Signal diversity, quantified via Lempel–Ziv complexity, was highest in eyes-closed conditions, consistent with recent findings^44^, suggesting that internally generated perceptions are associated with increased signal complexity (Fig. 6d). Alpha-band activity (8–12 Hz), typically associated with suppressing visual input during eyes-closed conditions^74,75^, decreased under psilocybin, particularly in visual regions. This reduced sensory filtering, combined with changes in MRI functional connectivity patterns, suggests diminished distinctions between eyes-open and eyes-closed conditions. This convergence, observed in both fMRI and EEG, is unlikely to reflect lower vigilance during the eyes-closed conditions, given robust baseline eyes-closed alpha and elevated eyes-closed signal complexity under psilocybin (see Supplementary Information for vigilance considerations).

Comparable decreases in alpha power and increases in signal diversity have also been observed under intravenous administration of the serotonergic psychedelic *N*,*N*-dimethyltryptamine^43^, suggesting that these may be conserved markers of serotonergic psychedelic action, despite differences in compound, route of administration, timing and EEG methodology. The convergence of the MRI and EEG findings confirms that this effect cannot be explained solely by vascular changes caused by psychedelics^1^. Together, these results establish context alignment as a property of brain dynamics under psilocybin that is evident across imaging modalities, timescales and analytical approaches.

### Embeddedness as brain–mind continuity

Machine learning embeddings revealed an association between the positively felt experience of self-dissolution and boundary dissolution and the reorganization of brain networks into context-aligned clusters (Fig. 2b,e). The relative activation of these networks regulates the balance between internally (for example, DMN) and externally directed processing, a dynamic that shapes perception and cognition^33,76^. Their increased integration under psilocybin suggests a shift towards more flexible connectivity and altered functional interactions (Fig. 2e).

The correlation between self-dissolving and boundary-dissolving effects and classification accuracy based on machine learning embeddings (Fig. 2c) indicates that these embeddings capture a connectivity state in which internal and external processes become less distinct. This is consistent with the reports of participants of feeling integrally part of a broader physical and psychological relational context. We refer to this state as embeddedness, the phenomenological correlate of context alignment. Unlike connectedness, which implies links between separate entities, embeddedness is the subjective experience of being continuous with, rather than separate from, the environment, which emerges when brain dynamics become aligned with context in proportion to the depth of self-dissolving and boundary-dissolving effects. Psychedelics appear to facilitate this state—a perceptual and cognitive realization of being part of a unified whole—by reorganizing brain network dynamics to increase integration within and between networks (Fig. 2e). The context sensitivity of this reorganization—its dependence on what the participant is doing and experiencing during psilocybin administration—suggests that the underlying synaptic changes may themselves be shaped by ongoing brain activity.

Although embeddedness refers to an experiential state, CEBRA and TAVRNN are machine learning methods that learn embedding spaces from imaging data. Although the experiential construct and the computational representations are conceptually distinct, the context-organized clustering of low-dimensional coordinate vectors within the learned embedding spaces covaried with the intensity of subjective effects (Figs. 2b,e and 3a,b), and classification accuracy covaried specifically with positively felt, self-dissolving and boundary-dissolving effects (for example, spiritual, blissful and unitive; Fig. 2c), with weaker associations for sensory–hallucinogenic effects and none for negative effects (for example, impaired control and cognition, and anxiety).

This pattern supports interpreting embeddedness as a state central to the therapeutic relevance of psychedelics, potentially by addressing distress arising from separation. We used separation to mean the pervasive, felt disconnection between self and world (including self–other boundaries and rigid self-referential attachment). Interpreted this way, embeddedness marks a transient reduction of that separation, an existentially salient state that may help to explain reported reductions in death-related anxiety and existential distress following psychedelic treatment^3,4,77^, and, in our non-clinical cohort, is consistent with self-dissolving and boundary-dissolving experiences (Fig. 4c–g and Extended Data Fig. 9), increased personal meaning and death acceptance, and greater nature-relatedness (see Supplementary Information).

Furthermore, the link between positively felt associative effects and improved mindset (Fig. 4h) was replicated using CEBRA embeddings, where classification accuracy was associated with next-day mindset change (*r* = 0.4, *P* < 0.01; Extended Data Fig. 6). Although this association is probably mediated by subjective experience, it supports the view that brain embeddings capture meaningful experiential features of the psychedelic state that relate to subsequent psychological change. This suggests that brain-derived embeddings may offer a complementary approach for tracking participant-specific correlates of change where self-report is unavailable or unreliable.

The blissfully felt state of boundary-dissolving embeddedness may reflect a general mechanism of psychological adaptation, one that opens a sense of boundlessness and remediates separateness. Under supportive conditions, this can enable personally meaningful acute experiences that translate into persisting psychological benefits^78^. This interpretation is consistent with therapeutic efficacy observed in depression^9^, and with reports from participants of communitas, empathy and nature-relatedness, reflecting an attenuation of self–world boundaries characteristic of embeddedness^12–14,79^. Embeddedness thus offers a construct that may inform clinical approaches to mental health, by characterizing the extent to which the brain and mind of a person are aligned with context under pharmacologically altered experience.

The learned embedding spaces point to a deeper principle. Ordinarily, segregation between internally and externally directed systems buffers brain dynamics from direct environmental coupling, maintaining the separation between internal models and sensory context on which predictive processing depends^80^. Under psilocybin, this boundary dissolves. Rather than maintaining separation from context, brain activity differentiates more coherently across contexts and becomes temporally coherent within each context (Fig. 2a,e).

These results address a key gap in systems neuroscience: how large-scale functional brain embeddings can shift under multiple contextual demands in a pharmacologically altered state. Network perturbation analysis grounds this organization anatomically, identifying the DMN and visual network as its primary contributors (Fig. 3g–i). This anatomical anchoring is at the level of large-scale functional systems, not lower-level neurophysiology, and does not assign unique network generators to individual contexts (Extended Data Fig. 7b). Context alignment is a distributed, temporal property of whole-brain dynamics, and the perturbation analysis identifies which systems’ dynamics are necessary for that organization to emerge, providing a neurobiological account at the grain that whole-brain fMRI can rigorously address, and a concrete entry point for finer-grained mechanistic investigations to follow. These results also position embeddedness as a data-driven construct that bridges measurable organization of brain activity with both acute and enduring effects, providing an alternative to accounts of psychedelic experience that are difficult to operationalize, such as ego dissolution^81^.

Crucially, machine learning embedding techniques show that the prevailing interpretation of psychedelic resting-state network integration–segregation dynamics, commonly summarized by modularity changes, is incomplete. Reductions in within-network functional connectivity, often interpreted as a loss of within-network organization, coexisted with tighter clustering of regional dynamics in the TAVRNN embedding (Fig. 2e), and modularity did not predict the individual differences in subjective experience or mindset change that CEBRA classification accuracy predicted, indicating that experientially meaningful organization resides within the temporal dynamics that time-averaged summaries do not preserve.

By revealing structured organization aligned with context where previous studies found desynchrony^5,6^, these embeddings recast the psychedelic state: apparent disorder in time-averaged measures masks dynamic organization that emerges in proportion to subjective experience. This view is now supported at the circuit level by preclinical evidence that psilocybin selectively weakens corticocortical recurrent pathways while strengthening specific feedforward routes from perceptual and medial regions (the latter a rodent DMN homologue) in an activity-dependent manner^82^.

Although further research is needed to determine whether machine learning embedding signatures generalize across different populations, CEBRA demonstrates how dynamic functional trajectories can uncover structured organization missed by static connectivity approaches, with broad applications in consciousness research and psychiatry.

### Brain activity aligns with context

Our analyses converge on a redistribution of integration under psilocybin, which provides the systems-level basis for how context shapes brain dynamics during the psychedelic state. Time-averaged connectivity (fMRI) and spectral power (EEG) confirmed this redistribution across modalities, whereas temporally resolved trajectory analysis revealed that context increasingly differentiated brain dynamics as subjective effects intensified (Fig. 3a–d), indicating that context alignment is graded by experiential depth rather than imposed by external stimulation alone.

The GFC analysis demonstrated that psilocybin rebalanced sensory and associative connectivity. During eyes-closed conditions, sensory integration decreased, whereas associative integration increased, consistent with a redistribution across the cortical hierarchy.

Our larger sample helps to clarify discrepancies in earlier reports of GFC patterns from serotonergic psychedelic studies, in which spatial patterns varied across datasets and global signal regression^43,83,84^. Further analysis using histograms of GFC values revealed that psilocybin reduced the separation between eyes-open and eyes-closed states, both globally and across individual networks, and was particularly evident in the visual network (Fig. 1d). Similar sensory region degree centrality reductions have been reported under lysergic acid diethylamide (LSD), 3,4-methylenedioxymethamphetamine (MDMA) and d-amphetamine^48^, indicating that sensory global connectivity decreases can occur outside the class of serotonergic psychedelics.

Psilocybin also redistributed BOLD signal variability across sensory and associative regions. During eyes-closed states, variance increased in ventral, temporal and somatosensory areas and decreased in the early visual cortex, consistent with the redistribution of integration between sensory and associative regions observed in our GFC findings. These changes align with previous evidence of increased entropy under psychedelics, measured as magnetoencephalography–EEG signal diversity, fMRI sample entropy and model-derived neuronal firing rate entropy^31,85–88^, demonstrating how psilocybin reshapes spatial signal dynamics in a context-sensitive manner.

The 64-channel wet EEG provided a separate modality that confirmed the pattern of context-sensitive changes that we identified in fMRI. The reduced alpha-band inhibition observed under psilocybin during eyes-closed states suggests a mechanism for the spontaneous production of internally generated visual effects^74^. Lempel–Ziv complexity increased brain-wide during eyes-closed conditions, extending previous evidence of these effects in occipital–parietal regions^85^. Meditation and music show visually similar group-averaged power spectra at baseline and under psilocybin (Fig. 6b); owing to our fixed-order design, we have reported these findings descriptively (see Methods). Similar reductions in signal diversity (Lempel–Ziv complexity) under external stimulation have also been reported under psychedelics^44^.

Therefore, although the spatial organization of connectivity (fMRI surface maps) and brain dynamics (EEG scalp topographies) remains distinct from baseline (no psilocybin), the overall distributions of connectivity strength (GFC values; Fig. 1d,e) and spectral power (EEG power; Fig. 6b) converge under psilocybin, indicating that eyes-closed states shift towards a more externally engaged profile while preserving condition-specific topology. This pattern reflects a redistribution of integration in which associative networks participate more broadly during eyes-closed states while sensory networks become less constrained by baseline organization. The convergence of sensory state boundaries in time-averaged functional connectivity (Fig. 1b,d) is not a loss of structure but a reorganization compatible with the context-aligned organization captured by low-dimensional trajectory analysis (Fig. 2a). As rigid distinctions between eyes-open and eyes-closed states relax, temporally ordered dynamics become more distinctly locked to each context.

DCM analysis also linked these findings to emerging preclinical and human evidence of aHip–DMN neuroplasticity following psilocybin^6^. Using DCM, we demonstrated that eyes-open versus eyes-closed context differentially tunes the effective connectivity of these brain circuits during the acute effects, with the eyes-open movie showing the greatest magnitude of directed change, in line with distinctions in our fMRI and EEG findings.

Machine learning embeddings distinguished patterns of psychedelic brain activity across experimental contexts (rest, meditation, music and movie), with classification accuracy scaling with the intensity of acute subjective experience—particularly positively felt, immersive self-dissolving and boundary-dissolving dimensions (for example, mystical, blissful and unitive). Together, increased integration of cortical–subcortical connectivity—including interactions between systems that ordinarily segregate internal (self-referential) and external (environment-focused) processing—modelled with TAVRNN, and the context separability uncovered by CEBRA, converge to support the construct of embeddedness: a state in which individuals feel fundamentally continuous with their environment, marked by diminished self–world separation. These signatures were detectable at the individual level and associated with next-day mindset improvements, providing a brain-derived marker of large-scale brain reorganization that connects the quality of subjective experience to the change that follows.

Our findings extend prevailing accounts that characterize the psychedelic state as desynchronized or entropically disordered by revealing structured, context-aligned organization in BOLD signal dynamics—a latent order harboured within temporal dynamics that time-averaging obscures—which emerges in proportion to the depth of self-dissolving and boundary-dissolving experience. This organization is distributed across networks but depends on the joint alteration of DMN and visual systems: the gradient end points that ordinarily segregate internal from external processing. When both are altered, that functional boundary dissolves, setting the conditions for brain activity to align with context, an integration that provides a neurobiological rationale for how structured settings can shape outcomes under psychedelics. That the felt boundary between self and world reorganizes when these dynamics shift implies that the boundary is not a fixed datum of conscious experience but a construction actively maintained by neural activity. Embeddedness—the continuity that emerges when this construction relaxes—may thus reveal less about what psychedelics add to consciousness than about what ordinary neural dynamics keep apart.

## Methods

### Ethics and clinical trial registration

The study protocol was approved by the Monash University Human Research Ethics Committee. The trial was registered with the Australian New Zealand Clinical Trials Registry under the registration number ACTRN12621001375842. The research was performed in accordance with all relevant guidelines and regulations, and written informed consent was obtained from all participants.

### Study design

The PsiConnect study was open-label and included two imaging sessions: a baseline (no psilocybin) session and a session following the administration of a 19 mg dose of psilocybin. Both sessions involved MRI and EEG scans, with four conditions repeated in each: resting state, guided meditation, music listening and movie watching. In fMRI, conditions began approximately 80 min post-dose and followed a fixed order (rest → meditation → music → movie). The sequence progressed from low-stimulus to high-stimulus contexts to prioritize safety and experiential coherence for psychedelic-naive participants. The EEG session started after a room transfer and 20–40 min of setup (cap placement and impedance checks), with the movie presented first, followed by the three eyes-closed conditions (movie  → rest → meditation → music). Discussion addressing expectancy, condition order and EEG timing is detailed in Supplementary Information.

In the resting-state condition (8 min MRI, 5 min EEG), participants were instructed to relax and keep their eyes closed while remaining still. During the guided meditation (6 min 30 s MRI, 5 min EEG), participants received meditation guidance via MRI-safe audio, with guiding prompts interspersed between silent periods of practice. For the music listening condition (11 min 24 s MRI, 7 min EEG), a curated playlist was designed to evoke emotional depth and resonance. In the naturalistic movie condition (6 min MRI, 5 min EEG), participants watched a video of moving clouds without audio. EEG blocks were intentionally shorter (total ≈ 22 min) to acquire all four contexts within the acute effects window and to minimize fatigue, motion and impedance drift; see the accompanying PsiConnect data descriptor^89^. These conditions were studied in both the baseline (no psilocybin) and psilocybin sessions and were repeated in both fMRI and EEG, allowing for comprehensive cross-modal and longitudinal analyses of brain activity and connectivity.

The eligible participants were stratified based on age and self-reported gender before allocation to the mindfulness meditation and control groups using a non-randomized, balanced procedure. No blinding was performed at any stage, including data analysis. Half of the participants were assigned to an 8-week mindfulness-based cognitive therapy programme, ‘Finding Peace in a Frantic World’, run by a trained and registered instructor, which involved weekly group meetings and daily independent practice; the other half were assigned to a control group with no intervention. Each participant was scanned at both baseline (no psilocybin) and under psilocybin. Engagement was consistent across the mindfulness-based cognitive therapy group: participants attended at least six of eight sessions and averaged 85 min of independent practice per week. Because no statistically significant differences were observed between meditators and non-meditators in connectivity or subjective effect measures under psilocybin, the two groups were pooled for all analyses reported here. Sample size was constrained by recruitment capacity and available imaging resources. No statistical methods were used to predetermine sample size.

Our decision to administer a standardized dose of 19 mg psilocybin rather than a body weight-adjusted dose was based on previous research showing no clear advantage of weight-adjusted dosing in terms of subjective effect intensity or predictable differences in response across individuals of varying body weight^90^. The dosage was administered as one oral capsule and selected in consultation with multiple collaborators who had previous psychedelic imaging experience. This dose was determined to be tolerable for the majority of healthy adults undergoing imaging procedures, while also sufficient to produce substantial subjective effects.

Several behavioural measures were collected before and during the baseline (no psilocybin) and psilocybin scans (see the section ‘Reported behavioural measures’). The follow-up conducted the day after psilocybin administration included semi-structured, open-ended questions and experience ratings. Further follow-up measures were administered 1 week and 1, 3, 6 and 12 months after psilocybin administration.

### Participants

Sixty-five healthy adults 18–55 years of age (37.7 ± 10.7; 30 female and 35 male) with no psychedelic experience were recruited. Sex was recorded at imaging intake and is reported here; self-reported gender, which was used for stratification between meditators and non-meditators and is concordant with sex for all but one participant, is detailed in the Reporting Summary. Participants were required to have no formal meditation practice and limited previous exposure to meditation. They were first screened via short online survey and then detailed screening was performed by a suitably trained staff member for excluding any psychopathology using the long-form SCID-5 (ref. ^91^). Exclusion criteria included a history of psychiatric disorders or suicidality; a 5-year history of substance and/or alcohol use disorder; first-degree relatives with a diagnosed psychotic disorder; a history of major neurological disorders including stroke or epilepsy; formal meditation practice within the past 6 months or extensive previous exposure to mindfulness meditation; use of contraindicated medications; and any serotonergic psychedelic use within the past 6 months. Participants were also screened for magnetic resonance contraindications and provided informed consent. Although hallucinogen use was assessed via the SCID-5, exclusion was applied to serotonergic psychedelic use specifically. We operationally defined ‘no psychedelic experience’ as no previous serotonergic psychedelic use with subjective effects. During structured screening, three participants disclosed nominal or remote lifetime exposures without subjective effects: two reported ineffective microdoses, and one reported remote exposure over 20 years previously with minimal or no recall. These cases are detailed in Supplementary Table 6. The remaining 62 participants reported no lifetime serotonergic psychedelic use across online, phone and SCID checks. On the day of psilocybin administration, they were assisted by a study doctor, researchers, laboratory staff with relevant training, and volunteers from the community drug harm reduction support organization ‘DanceWize’. Psilocybin was generally well tolerated, although some adverse effects were reported, including reports of transient headaches during the night (*n* = 3). Three participants received follow-up support from a clinical psychologist familiar with psychedelic integration. These follow-up calls were conducted over the phone, and no further support was required. On no occasion was it deemed necessary by the study doctor to administer an anxiolytic.

Of the 65 participants enrolled, 2 were withdrawn before the psilocybin session after meeting exclusion criteria identified during the study period. Their baseline data were not analysed, leaving a baseline sample of 63 per condition for both fMRI and EEG. No baseline fMRI or EEG recordings required further exclusion on quality control. The remaining 63 participants received psilocybin. Of these, one did not complete any post-dose imaging or EEG, and a second partially completed the resting-state fMRI scan (which was retained) but did not complete EEG. This gave post-dose fMRI samples of 62 (rest) and 61 (meditation, music and movie). Two further participants did not complete the post-dose EEG session, giving 59 completed EEG recordings per condition. Quality control applied pre-established thresholds for head motion and signal quality (see the section ‘MRI quality control’). During the psilocybin session, seven participants had one or more fMRI conditions excluded (two rest, two meditation, five music and four movie), resulting in final analysed fMRI samples of: rest = 60, meditation = 59, music = 56 and movie = 57. For machine learning analyses (CEBRA and TAVRNN), participants were included only if all four conditions passed quality control with complete acquisition (expected volume count across the scanning session), giving a balanced sample of *n* = 54. For psilocybin EEG, one recording was excluded due to missing event markers (final *n* = 58 per condition). A full table of per-participant quality control exclusions is included in the Reporting Summary and data descriptor^89^.

### Reported behavioural measures

Reported measures were part of a broader assessment conducted before and longitudinally after psilocybin administration, with a subset integrated into the present analysis. The 11D-ASC scale (42 items)^58,92^ was administered post-EEG at baseline and at the end of the psilocybin session (approximately 320 min after dose) to assess subjective alterations in consciousness. The MEQ30 (ref. ^57^) was also collected at this time to measure mystical-type experiences, along with the Aesthetic Experience Scale–Music (AES-M), used to assess the intensity of emotional and aesthetic responses to music. The AES-M scale is derived from the AES^93^, refined through psychometric validation^94^ and adapted for music-related experiences^95^. We adapted the instructions of this measure for collection on the day of psilocybin by asking participants to respond with reference to their musical experience during the psilocybin session. The order of scale presentation was randomized. A music experience measure^72^ assessed participants’ ratings of liking, openness and resonance with the music. It was administered after both the MRI and the EEG, on both session days. Scores were averaged to produce a single music experience rating.

One day after psilocybin, participants provided self-reported intensity ratings assessing the perceived strength of the experience. To assess the perceived meaningfulness of the experience, participants were asked: ‘would you rate the experience among the most meaningful and spiritually significant experiences of your life?’ If they responded yes, they were then asked: ‘where would you rate the experience among the most meaningful and spiritually significant experiences of your life?’ Response options included top 200 (*n* = 1), top 100 (*n *= 0), top 50 (*n *= 3), top 10 (*n *= 9) and top 5 (*n *= 24)^7^. A novel mindset measure capturing psychological change was also collected. See Supplementary Information for mindset measure details.

The Nature Relatedness Scale-6 (ref. ^62^) and Life Attitudes Profile-Revised^61^—assessing death acceptance, coherence and purpose—were collected before psilocybin and 1 month post-administration to examine changes in ecological connectedness and existential meaning.

Colour groupings (blue, orange, red and purple) shown in Figs. 2c and 4c,e,g,h were assigned post hoc based on within-sample intercorrelations in Extended Data Fig. 9 and theoretical rationale; they are descriptive and are not a validated taxonomy. In Fig. 4g, grey marks paired subscales from different groupings.

For the subscale correlation matrix (Extended Data Fig. 9), Bonferroni correction was applied across the 105 unique off-diagonal pairs (*α* = 4.76 × 10^−4^). Brain–behaviour associations involving individual subscales (Fig. 4h and Extended Data Fig. 6) are reported with uncorrected *P* values and effect sizes, with multiple-comparisons context provided in the respective figure captions. Per-subscale sample sizes for the next-day mindset change correlations (Fig. 4h, pairwise Pearson): *n* = 54 (insightful, anxiety and impaired cognition); *n* = 55 (disembody); *n* = 56 (unity and percepts); *n* = 57 (blissful and spiritual); *n* = 58 (complex); *n* = 59 (audiovisual and elementary); and *n* = 60 (mystical, positive, transcend and ineffability). The two-sided *P* values were: insightful, *P* = 9.9 × 10^−8^; mystical, *P* = 3.3 × 10^−7^; positive, *P* = 7.4 × 10^−7^; blissful, *P* = 6.1 × 10^−6^; spiritual, *P* = 3.1 × 10^−5^; ineffability, *P* = 3.2 × 10^−5^; unity, *P* = 1.5 × 10^−4^; percepts, *P* = 3.4 × 10^−4^; transcend, *P* = 5.6 × 10^−4^; audiovisual, *P* = 1.7 × 10^−3^; disembody, *P* = 9.9 × 10^−3^; complex, *P* = 0.014; elementary, *P* = 0.17; anxiety, *P* = 0.61; and impaired cognition, *P* = 0.71.

### MRI acquisition

Structural and functional MRI data were acquired using a Siemens 3 Tesla Magnetom Skyra scanner at Monash Biomedical Imaging. T1-weighted (T1w) anatomical images were obtained for each participant during two sessions: at baseline (no psilocybin) and on the psilocybin administration day. The images were acquired using a three-dimensional magnetization-prepared rapid gradient-echo sequence with a 32-channel head coil. The acquisition parameters were as follows: repetition time (TR) of 2,300 ms, echo time (TE) of 2.07 ms, 192 slices per slab, 1-mm slice thickness and 1-mm isotropic voxel size. The parallel acquisition technique was GRAPPA. For the structural T1w scan only, the acceleration factor (PE) was 2 at baseline (5:12) and 3 on the psilocybin administration day (3:52) to minimize scan time. A T2w anatomical image was obtained only during the baseline session. The acquisition parameters were: TR of 3,200 ms, TE of 452 ms, 176 slices per slab, 1-mm slice thickness and 1-mm isotropic voxel size, with an acceleration factor of 2.

BOLD fMRI data were collected using a multi-echo, multi-band, echo-planar imaging, T2*w sequence. The acquisition parameters were: TR of 910 ms; multi-echo TE of 12.60 ms, 29.23 ms, 45.86 ms and 62.49 ms; multi-band acceleration factor of 4; field of view of 206 mm; right–left phase encoding direction; and 3.2-mm isotropic voxels. The scan durations were: resting state with eyes closed (8 min, 505 volumes), audio-guided meditation with eyes closed (6 min 30 s, 405 volumes), music listening with eyes closed (11 min 24 s, 728 volumes) and movie watching (6 min, 372 volumes).

The structural and functional MRI images acquired from the Siemens scanner were converted into the Neuroimaging Informatics Technology Initiative format and organized according to the Brain Imaging Data Structure (v1.7.0).

### MRI quality control

Quality control was performed using the MRIQC Brain Imaging Data Structure app^96^, which uses structural MRI and fMRI images to compute several quality metrics. These include the temporal signal-to-noise ratio and the framewise displacement that quantifies head motion^97^. Principal component analysis of these metrics revealed some outliers in the structural and functional images, which were further inspected visually. This process led to the exclusion of six structural T1w images from the psilocybin session, while all the T1w images from the baseline session were retained. For the fMRI data, seven participants had one or more conditions excluded from the analysis (rest = 2, meditation = 2, music = 5 and movie = 4). The list of excluded scans per participant is available in the PsiConnect data descriptor^89^. Most of these outliers corresponded to scans where the head motion was large (mean framewise displacement greater than 0.5, which is often used as an exclusion threshold^98^). The final analysed MRI samples were: rest = 60, meditation = 59, music = 56 and movie = 57.

### MRI preprocessing and cleaning

Anatomical MRI and fMRI data were preprocessed using fMRIprep (v22.0.2). The anatomical MRI preprocessing involved correcting T1w images for intensity non-uniformity, skull-stripping and segmenting brain tissues. The images were then registered, brain surfaces reconstructed and spatial normalization performed using the ICBM 152 nonlinear asymmetrical template version 2009c (MNI152NLin2009cAsym). The fMRI preprocessing involved generating a reference volume and skull-stripped version from the shortest echo of each BOLD run, estimating head-motion parameters and performing slice-time correction. The BOLD reference was co-registered to the T1w reference, confounding time series were calculated and the BOLD time series were resampled into MNI152NLin2009cAsym space.

The preprocessed and optimally combined data produced by fMRIprep was cleaned via a single regression in SPM12 (ref. ^99^) using the general linear model. The regressors were: the white matter and cerebrospinal fluid signals computed by fMRIprep, the framewise displacement and the non-BOLD components identified via multi-echo independent component analysis (ICA) performed using tedana (v0.0.12)^100^. In short, multi-echo ICA identifies non-BOLD components that are independent of the echo time^101^. Very low-frequency components (especially the linear trend) were implicitly filtered out after cleaning^89^, which provides further evidence that multi-echo ICA was able to identify spurious components in the data. Finally, to enable surface-based analyses, the cleaned volumetric data were projected to the FreeSurfer left–right-symmetric cortical surface template with 32,000 vertices for each hemisphere (fsLR32k).

### The s.d. maps

The s.d. was computed for the BOLD time series of each vertex on the cortical surface. The percentage change (psilocybin versus no psilocybin) was computed for each participant and then averaged across participants to obtain the mean s.d. percentage change map shown in Fig. 1c. A surface-based general linear model was used to assess whether changes in BOLD s.d. were attributable to head motion, and in which cortical areas. For each condition and hemisphere, the vertex-wise s.d. percentage change between baseline and psilocybin (Δ*s.d.*_*v*_) was regressed on participant-level framewise displacement (FD) change using the following model: $\Delta s.d{.}_{v}={\beta }_{0}+{\beta }_{1}\Delta FD+{\beta }_{2}\overline{{\rm{F}}{\rm{D}}}+{\varepsilon }_{v}$, where Δ*FD* is the change in mean FD within each participant, $\overline{\,{\rm{FD}}}$ is the mean FD across sessions, *β*_0_, *β*_1_ and *β*_2_ are the linear regression coefficients and *ε* represents the residuals. Inference on *β*_1_ used non-parametric permutation testing with family-wise error correction using FSL PALM. Significant positive associations between s.d. change and FD change were confined to regions near air–tissue interfaces (the orbitofrontal cortex and anterior temporal lobe). The corresponding voxels were masked out in Fig. 1c, so that only s.d. changes that were not significantly associated with head motion remain. These motion-related effects were specific to s.d., and the spatial organization of GFC changes was not significantly associated with framewise displacement.

Although s.d. and Shannon entropy are distinct, there is a monotonic relationship between the two when the data distribution is Gaussian. More generally, these two quantities are tightly related when the data distribution is continuous, unimodal and has finite variance, such as in fMRI data. Variance is bounded above and below by scaled entropy powers, so that as one increases, the other is constrained to increase within bounded limits^102^. Accordingly, under these conditions, greater s.d. corresponds to greater Shannon entropy.

### GFC maps

For each cortical vertex, Pearson’s correlation to all other vertices in the same brain hemisphere was computed, transformed to Fisher *z*-values and averaged. This calculation yielded a GFC map in which each vertex value represents the mean correlation with all other vertices in the same hemisphere^45^. This generated one map for each participant, in each session (baseline and psilocybin) and in each condition (resting state, meditation, music and movie). The histograms of GFC values were used to compare the sessions and conditions, ignoring the spatial distribution across the cortex (Fig. 1e). To appreciate the spatial distribution, the GFC percentage change was computed (psilocybin day minus baseline, then divided by baseline) for each participant and then averaged across participants, yielding the mean GFC percentage change maps shown in Fig. 1a.

Threshold-free cluster enhancement (TFCE)^103^ was used to identify significant clusters in the spatial maps without defining arbitrary thresholds for cluster size. Intuitively, TFCE tests all thresholds and gives higher scores to clusters of vertices that are both large and survive increasingly stringent thresholds. TFCE was computed using the PALM software with the default parameters (see https://github.com/andersonwinkler/PALM/blob/master/palm_defaults.m). The statistical TFCE maps were thresholded at *P* < 0.05 with 1,000 permutations and a gamma approximation for the distribution tail (computing permutations is computationally very costly; in our tests, the approximation produced nearly identical clusters to those obtained using 10,000 permutations but reduced the computation time by one order of magnitude). Once the clusters of increased and decreased GFC were obtained, they were used to mask the GFC maps of the effect of psilocybin (percentage change) to hide the vertices outside these clusters (Extended Data Fig. 2a). Effect sizes are reported as Cohen’s *d*, computed using the Algina–Keselman–Penfield robust estimator, which uses trimmed means and Winsorized variances to reduce sensitivity to outliers and non-normality^104^.

### Note on GSR and *z*-scoring GFC

We recommend not performing global signal regression (GSR) when computing the GFC, although it has been common practice in previous research^43,83,84^. Mathematically, GSR alters the covariance matrix of the data such that the average of each row is zero^105^. As GFC is computed by averaging the rows, it would become zero for each vertex across the cortex. The reason why GFC is not exactly zero in the literature using GSR is that the Pearson correlation matrix is used instead of the covariance matrix. The correlation matrix is a rescaled version of the covariance matrix in which all the entries are divided by the s.d. so that they are between −1 and 1. To achieve this normalization, each entry *σ*_*ij*_ of the covariance matrix is divided by the product of the s.d. of vertices *i* and *j*, that is, by $\sqrt{{\sigma }_{ii}}\sqrt{{\sigma }_{jj}}$. If all the vertices had the same s.d., this normalization would equally rescale all entries and the average of each row of the correlation matrix would still be zero. In practice, the s.d. varies across cortical vertices (Fig. 1c) so that the average of each row of the correlation matrix is not exactly zero, but typically a small value. We argue that this complicates the interpretation of the GFC, confounding the intended goal of quantifying ‘the extent to which a cortical vertex is positively or negatively correlated with the other vertices, on average’ with ‘the extent to which a vertex is positively or negatively correlated with vertices having large s.d., on average’.

Relatedly, plotting *z*-scored GFC values confounds the interpretation in two ways. If GSR is applied, *z*-scoring hides the fact that GSR substantially reduces the magnitude of GFC values. More generally, *z*-scoring is problematic when used to report GFC differences between two conditions. For example, in the main text, we have reported the percentage change between the psilocybin and baseline conditions (Fig. 1a). Positive and negative values indicate an increase or decrease in GFC, respectively. After *z*-scoring the changes, this interpretation is no longer possible: a positive *z*-score only indicates that the change is higher than average, regardless of its original sign (Extended Data Fig. 3). If the average were negative, negative values slightly above the average would have a positive *z*-score.

### Parcel-wise functional connectivity

The denoised, volumetric data were divided into parcels for further functional connectivity analysis. The Schaefer parcellation^106^ was used to partition the cortical voxels into 300 parcels, each belonging to one of the following seven resting-state networks: visual, somatomotor, dorsal attention, limbic, salience–ventral attention, default mode or control. The Melbourne subcortical atlas^107^ was used to divide the subcortical voxels into 32 parcels including subdivisions of the striatum, thalamus, hippocampus, amygdala and globus pallidus. (This atlas is specified in voxel space, hence the use of volumetric rather than surface data for this analysis.) Combining the cortical and subcortical parcellations gave a total of 332 parcels. To extract a representative time series for each parcel, the first principal component of the voxel time series within each parcel was computed.

The parcel-wise functional connectivity matrix was obtained by computing Pearson’s correlation between the time series of each pair of parcels, and the average functional connectivity matrices across participants are shown in Fig. 1b. The within-network functional connectivity was computed by averaging the functional connectivity values within the diagonal blocks of the functional connectivity matrix, each corresponding to the pairwise correlations between parcels belonging to the same functional network. Similarly, the between-network functional connectivity was computed by averaging the functional connectivity matrix entries outside the diagonal blocks, that is, the correlation values between pairs of parcels belonging to different networks (see Supplementary Information). The Mann–Whitney *U*-test was performed in MATLAB using the ‘ranksum’ function and the right-tailed option. That is, the alternative hypothesis is that the median within-network or between-network functional connectivity under psilocybin is lower than the baseline median. To correct for multiple comparisons across eight resting-state networks, the statistical significance was set to *α* = 0.05/8 = 0.00625 (Bonferroni correction).

### Modularity

The functional connectivity modularity was computed using the Brain Connectivity Toolbox^108^ with the ‘negative_asym’ option for asymmetric treatment of negative weights^109^. The Mann–Whitney *U*-test was performed in MATLAB using the ‘ranksum’ function and the right-tailed option. That is, the alternative hypothesis is that the median modularity under psilocybin is lower than the baseline (no psilocybin) median (Fig. 1f). To correct for multiple comparisons across four conditions, the statistical significance was set to *α* = 0.05/4 = 0.0125 (Bonferroni correction).

### Spectral DCM

Spectral DCM is a method used to infer the effective connectivity between brain regions from fMRI time series^68,110,111^. Effective connectivity is defined as the influence one neural system exerts over another^67^, as opposed to functional connectivity, which describes statistical dependencies among BOLD signals^69,112^. DCM uses a forward generative model with neuronal and observation equations. The neuronal model is a linear stochastic differential equation that describes the dynamics of hidden neuronal states. It models how the activity in one brain region is influenced by the activity in other regions, as well as by endogenous fluctuations in neuronal activity. The observation function maps the neuronal activity to the observed BOLD signal by modelling the biophysical processes involved in the haemodynamic response. Spectral DCM estimates the parameters of this model by fitting the generative model to the cross-spectral density of the observed BOLD signals, a second-order statistic that captures the correlations between time series at all time lags^111^. By fitting the model to the cross-spectral density, spectral DCM can estimate the effective connectivity between brain regions, as well as the haemodynamic parameters and the spectrum of the endogenous fluctuations. Because DCM is a Bayesian approach, all model parameters are equipped with prior distributions, which are updated based on the observed data to produce posterior distributions over the parameters. Spectral DCM explicitly models the endogenous fluctuations in neuronal activity, which makes it ideal for applications to the resting state and conditions that do not involve strong experimental inputs or block task designs.

Here we applied spectral DCM to infer the effective connectivity of each participant in each condition. The analysis focused on six regions belonging to the DMN, each modelled as a 6-mm sphere centred on the following coordinates: laHip (*x* = −26, *y* = −16, *z* = −20), raHip (*x *= 28, *y* = −16, *z* = −20), lIPC (x = −44, *y *= −60, *z* = 24), rIPC (*x *= 54, *y* = −62, *z* = 28), mPFC (*x* = 2, *y* = 56, *z* = −4) and PCC (*x *= 2, *y* = −58, *z* = 30). Participant-level fully connected DCMs were combined at the group level with parametric empirical Bayes, which precision-weights individual parameter estimates by their posterior uncertainty. Specifically, parametric empirical Bayes^113^ was used to compute the group-level effective connectivity at baseline and its change under psilocybin (Fig. 5). All DCM analyses were performed using SPM12 (ref. ^99^). See Supplementary Information for further details.

### CEBRA

CEBRA is a self-supervised learning framework that extracts interpretable and consistent low-dimensional embeddings from high-dimensional neural and behavioural datasets^56^. By leveraging contrastive learning and auxiliary variables, such as time or behavioural labels, CEBRA enables analyses of population-level trajectories.

In this study, we used CEBRA-Time, a variant of CEBRA tailored for temporal analyses, to explore latent brain trajectories derived from fMRI data. CEBRA-Time is a contrastive learning-based method designed to extract meaningful latent representations from time-series data, such as fMRI signals. It learns a low-dimensional embedding by maximizing temporal coherence while preserving task-related variability, allowing the transformation of high-dimensional neural data into structured latent trajectories. This enables the discovery of participant-specific brain dynamics in a more interpretable space.

CEBRA is well suited for fMRI data, as it summarizes the activity of all brain regions (ROIs) at each moment into a compact representation of the overall brain state and embeds these points in a lower-dimensional space. Each point in this space corresponds to a single fMRI volume: that is, a whole-brain snapshot across ROIs. The embeddings themselves are learned directly from the fMRI BOLD signals. Training is guided by a contrastive objective: volumes that are similar are encouraged to map closer together in the embedding space, whereas more dissimilar volumes are pushed farther apart. Plotting successive points yields a trajectory that makes shifts between rest, meditation, music and movie watching easier to see and quantify.

Here, for each participant, we concatenated fMRI time series spanning four consecutive conditions: resting state, guided meditation, music listening and movie watching (Fig. 2a). This comprehensive input captured temporal transitions in BOLD activity across conditions. Applying CEBRA-Time transformed each time point of these high-dimensional fMRI time series into a three-dimensional point in latent space, revealing latent brain trajectories unique to each participant (Fig. 2a,b). These trajectories provided insights into how dynamic brain activity evolves over different conditions, aligning with self-reported subjective experiences. We also confirmed that CEBRA embeddings were stable across repeated runs in our dataset (Fig. 3e,f), ensuring robustness of the trajectories presented here.

Thus, although even more conventional machine learning embedding methods confirm the separability of brain states across conditions (Extended Data Fig. 5), CEBRA provides a richer, temporally coherent view of how these states evolve into organized patterns of brain activity, making it uniquely suited to studying continuous experiential processes such as those induced by psychedelics.

### Network perturbation analysis

To identify which functional systems contribute most to the context-aligned trajectory organization revealed by CEBRA, we performed a network perturbation analysis using a baseline substitution approach. For each participant, the psilocybin time series of a given functional network was replaced with the corresponding baseline (no psilocybin) dynamics of that participant, whereas all other networks retained their psilocybin activity. The CEBRA embedding and SVM classification were then re-run on this hybrid time series, and the drop in classification accuracy was measured. This procedure isolates the contribution of the psilocybin-specific modulation of each network to the overall context-aligned organization, while preserving the natural statistical structure of the biological signal of the individual. Only positive contributions were retained when constructing an additive attribution, ensuring that individual network contributions sum to the total psilocybin-induced accuracy gain (see Supplementary Information for mathematical details). The analysis was performed on each individually trained CEBRA model to assess whether the anatomical anchoring was consistent across participants. This attribution quantifies the contribution of each network to the overall multi-context organization; it does not assign unique network generators to individual context contrasts.

To further characterize condition-specific contributions, we computed per-condition recall (the fraction of time points truly belonging to a given condition that were correctly classified) from the confusion matrix of the SVM classifier trained on psilocybin embeddings. We then repeated inference after each network substitution and extracted the change in per-condition recall (diagonal of the row-normalized confusion matrix). The mean change in recall across participants (psilocybin minus substituted) is reported for each network and condition.

### TAVRNN

Although CEBRA captures overall latent brain trajectories, a deeper understanding requires analysing how individual brain regions (ROIs) evolve over time and interact with each other. TAVRNN allows us to track these ROI-specific dynamics and their changing patterns of interaction (that is, functional connectivity), revealing finer details. This captures detailed, context-dependent communication between regions that is undetectable by static analyses.

This perspective inspired our application of TAVRNN^59^. TAVRNN is designed for analysing the temporal dynamics of evolving connectivity networks. It is a deep learning method that models temporal connectivity networks between units of a system by leveraging both structural relationships and past dynamics. Through temporal attention, recurrent neural networks and variational graph techniques, it learns a lower-dimensional latent representation for each unit (ROIs in this work), preserving meaningful temporal patterns while enhancing interpretability. This allowed for tracking the evolution of ROI activities over time, revealing insights into their dynamic interactions. TAVRNN captures both local temporal patterns (how individual ROIs change over time) and global temporal patterns (how the relationships as a whole evolve dynamically). By leveraging latent information from the structure of time-varying functional connectivity networks, TAVRNN enables a robust representation of individual ROI activity and their changing relationships in a low-dimensional space (Fig. 2e). Specifically, a temporal attention mechanism assesses the topological similarity of the network across time steps, incorporating varying time lags to capture complex network dynamics more effectively. This approach is particularly effective in uncovering temporal changes in network structures and their alignment with external behaviours or stimuli.

In our study, we used TAVRNN to analyse functional connectivity matrices derived from fMRI time series under four consecutive conditions: rest, meditation, music and movie. For each condition, we computed the functional connectivity matrices between 332 brain parcels, which served as input nodes to TAVRNN (*n* = 54; the same participants used in the CEBRA analysis). No segmentation or overlapping windows were applied; each functional connectivity was computed using the full time series for that condition to capture stable, condition-specific network states. In this work, each scan under a specific condition (rest, meditation, music or movie) was treated as a temporal snapshot. Therefore, the attention mechanism enables the lower-dimensional representation of one scan to inform the representation of the subsequent scans. The model then generated two-dimensional embeddings for each parcel, representing their temporal evolution across conditions (Fig. 2e).

For further details on biological interpretability of the latent spaces derived from our machine-learning analyses, and conventional and machine learning metric complementarity (Supplementary Figs. 8 and 9), see the Supplementary Information.

### EEG acquisition

EEG data were recorded using a 64-channel BrainAmp MR Plus amplifier (Brain Products) with BrainVision Recorder software (v1.22.0001, Brain Products) and Ag–AgCl electrodes embedded in an actiCAP slim cap according to the standardized 10–10 system^114^. The FCz electrode served as the reference and the FPz electrode served as the ground for online recording. The impedances were maintained below 10 kΩ using an abrasive paste (Nuprep, Weaver and Company) followed by applying a conductive gel (Easycap). EEG signals were sampled at 500 Hz with a bandpass filter of 0.01−1,000 Hz. Participants were comfortably seated in an acoustically absorbent room to minimize environmental noise, and their chin strap was securely fastened to ensure head stability. They were instructed to remain as still as possible, avoid jaw clenching and keep their eyes closed during the recording to minimize artefacts.

The EEG recording session included the same four conditions as the fMRI described above. The naturalistic movie (5 min) was presented first, followed by the three eyes-closed conditions: resting state (5 min), audio-guided meditation (5 min) and music listening (7 min). The raw data were acquired as a single continuous recording over all conditions and then split into four separate files using the event markers for the start and end of each condition. Owing to technical issues, event markers were not recorded for two participants in the baseline session and one participant in the psilocybin session, so these recordings were excluded from pre-processing and analysis. In summary, pre-processed EEGs were analysed for 63 participants in the baseline session and 58 in the psilocybin session.

### EEG pre-processing

The raw EEG signals were pre-processed using the default parameters of the automated RELAX pipeline^115^ implemented in MATLAB using functions from EEGLAB^116^ and FieldTrip^117^ toolboxes. First, the data were bandpass filtered between 0.25 Hz and 80 Hz using a fourth-order Butterworth filter with zero phase, with a notch filter applied between 47 Hz and 53 Hz to reduce line noise. Subsequently, bad channels were removed by applying a multistep process incorporating the ‘findNoisyChannels’ function of the PREP pipeline^118^. This was followed by the initial reduction of the artefacts related to eye movements, muscle activity and drift using a multi-channel Wiener filter^119^, after which the data were re-referenced to the robust average reference^118^. Residual artefacts were then removed by Wavelet-enhanced independent component analysis with the artefactual components identified by the automated ICLabel classifier^120^. Electrodes rejected during the cleaning process were then restored to the data through spherical interpolation. Finally, all pre-processed data were visually inspected for data quality.

### EEG data analysis

The power spectrum was computed for all electrodes across all participants using the multitaper frequency transformation (1−45 Hz) with a 2-s Hanning window and 50% overlap, implemented in FieldTrip^117^. To quantify the effect of psilocybin, the power difference (psilocybin minus baseline) was computed for each channel and then averaged across participants. The results are reported in Fig. 6 for each condition and four power bands: theta (4−7 Hz), alpha (8−12 Hz), beta (13−30 Hz) and gamma (30−80 Hz).

The Lempel–Ziv complexity was calculated in two steps using the Lempel–Ziv 1976 algorithm^121^. First, the time series for each channel was binarized by setting values above the mean to 1 and values below the mean to 0. The resulting binary sequence was then scanned sequentially to identify distinct patterns and build a dictionary of these patterns. The Lempel–Ziv complexity is given by the number of patterns in the constructed dictionary. Regular signals can be represented by a small number of patterns, resulting in low Lempel–Ziv complexity, whereas irregular signals have a greater diversity of patterns, leading to higher Lempel–Ziv complexity.

### Reporting summary

Further information on research design is available in the Nature Portfolio Reporting Summary linked to this article.

## Online content

Any methods, additional references, Nature Portfolio reporting summaries, source data, extended data, supplementary information, acknowledgements, peer review information; details of author contributions and competing interests; and statements of data and code availability are available at https://doi.org/10.1038/s41586-026-10910-z.

## Supplementary information

Supplementary Information Supplementary Text and Data including Supplementary Figs 1–9 and Supplementary Tables 1–6—see Contents for details.

Reporting Summary

Peer Review File

## Data Availability

All data reported are open access and available through OpenNeuro (https://openneuro.org/datasets/ds006110; v1.2.0). Sensitive demographic information will be available upon signing a data access and confidentiality agreement (through the link available in the OpenNeuro data repository).

## Code availability

Open-source code for all data analysis pipelines is available on GitHub (https://github.com/razilab). These pipelines used the following software packages: fMRIprep v22.0.2 (https://fmriprep.org), tedana v0.0.12 (https://tedana.readthedocs.io), MRIQC v22.0.6 (https://mriqc.readthedocs.io), MATLAB R2022a (https://www.mathworks.com), SPM12 (https://www.fil.ion.ucl.ac.uk/spm/software/spm12), Freesurfer v7.2 (https://surfer.nmr.mgh.harvard.edu), FSL v6.0.7 (https://fsl.fmrib.ox.ac.uk/fsl/fslwiki) and FieldTrip 20240916 (https://www.fieldtriptoolbox.org). Lempel–Ziv complexity was computed by adapting open-source code by F. Rosas and P. Mediano (https://information-dynamics.github.io/complexity/information/2019/06/26/lempel-ziv.html). Projection of volumetric fMRI data to the cortical surface was performed by adapting open-source code by Pang et al., available on GitHub (https://github.com/NSBLab/BrainEigenmodes).
