# Supplementary material for: Psychedelics align brain activity with context
Source: Nature. 2026 Aug 19;656(8129):936–47. doi: 10.1038/s41586-026-10910-z (PMC13518247; doi:10.1038/s41586-026-10910-z)
Supplement: Supplementary file 2 [file 41586_2026_10910_MOESM2_ESM.pdf]

Corresponding author(s): Devon Stoliker, Adeel Razi

Last updated by author(s): Jun 11, 2026

Reporting Summary

Nature Portfolio wishes to improve the reproducibility of the work that we publish. This form provides structure for consistency and transparency in reporting. For further information on Nature Portfolio policies, see our Editorial Policies and the Editorial Policy Checklist.

Statistics

For all statistical analyses, confirm that the following items are present in the figure legend, table legend, main text, or Methods section.

n/a	Confirmed	
☐	☒	The exact sample size (*n*) for each experimental group/condition, given as a discrete number and unit of measurement
☐	☒	A statement on whether measurements were taken from distinct samples or whether the same sample was measured repeatedly
☐	☒	The statistical test(s) used AND whether they are one- or two-sided<br>*Only common tests should be described solely by name; describe more complex techniques in the Methods section.*
☐	☒	A description of all covariates tested
☐	☒	A description of any assumptions or corrections, such as tests of normality and adjustment for multiple comparisons
☐	☒	A full description of the statistical parameters including central tendency (e.g. means) or other basic estimates (e.g. regression coefficient) AND variation (e.g. standard deviation) or associated estimates of uncertainty (e.g. confidence intervals)
☐	☒	For null hypothesis testing, the test statistic (e.g. *F*, *t*, *r*) with confidence intervals, effect sizes, degrees of freedom and *P* value noted<br>*Give P values as exact values whenever suitable.*
☐	☒	For Bayesian analysis, information on the choice of priors and Markov chain Monte Carlo settings
☒	☐	For hierarchical and complex designs, identification of the appropriate level for tests and full reporting of outcomes
☐	☒	Estimates of effect sizes (e.g. Cohen's *d*, Pearson's *r*), indicating how they were calculated

Our web collection on statistics for biologists contains articles on many of the points above.

Software and code

Policy information about availability of computer code

Data collection	Stimuli were presented with PsychoPy3 (2021.2.3). Audio files were converted with VLC (3.0.12) and normalised in Audacity (3.1.3) to meet PsychoPy requirements.
Data analysis	Preprocessing and analysis used: fMRIPrep 22.0.2 (including ANTs 2.3.3, FSL 6.0.7, FreeSurfer 7.2.0, AFNI 23.3.06, Nilearn 0.9.1); tedana 0.0.12 for multi-echo denoising; SPM12 (r7771) for GLMs and ME-ICA confound regression; PALM for permutation testing; MATLAB R2022a; Brain Connectivity Toolbox 2022.12.08; FieldTrip 20240916; Python 3.12.2 and additional Python packages as cited. See https://github.com/razilab/psiconnect for custom code collection.

For manuscripts utilizing custom algorithms or software that are central to the research but not yet described in published literature, software must be made available to editors and reviewers. We strongly encourage code deposition in a community repository (e.g. GitHub). See the Nature Portfolio guidelines for submitting code & software for further information.

Data

Policy information about availability of data

All manuscripts must include a data availability statement. This statement should provide the following information, where applicable:

- Accession codes, unique identifiers, or web links for publicly available datasets
- A description of any restrictions on data availability
- For clinical datasets or third party data, please ensure that the statement adheres to our policy

All MRI, EEG and behavioural data supporting this study are publicly available on OpenNeuro under accession ds006110, version 1.2.0 (https://openneuro.org/datasets/ds006110/versions/1.2.0). Data are BIDS-conformant and include raw and derivative releases (e.g., /derivatives/fmriprep-22.0.2, /derivatives/tedana-0.0.12, cleaned EEG in FieldTrip format). Any restricted media files are documented with identifiers.

Analysis code and notebooks used to generate the reported results will be released upon publication at the repositories cited in the manuscript's Code availability section; core preprocessing used published, versioned tools listed above.

Research involving human participants, their data, or biological material

Policy information about studies with human participants or human data. See also policy information about sex, gender (identity/presentation), and sexual orientation and race, ethnicity and racism.

Reporting on sex and gender	Gender identity was self-reported during participant screening using the options: female, male, or non-binary/gender-diverse, and was reported as 30 female; 34 male; 1 non-binary/gender diverse. Separately, self-reported sex was collected as part of imaging screening, and was reported as 30 females and 35 males. No biological verification was conducted. Participants were stratified by age and self-reported gender to ensure balanced representation across experimental groups.<br><br>The manuscript reports biological sex. The recruited sample comprised 65 participants: 30 female, 35 male. Of these 62 participants participated in imaging at both baseline (no psilocybin) and psilocybin sessions: 28 female, 34 male.<br><br>The manuscript findings are not restricted to any one sex or gender and are considered applicable to adults irrespective of gender identity. Analyses were not conducted separately by gender, as this was not an a priori focus. While the sample size would permit such comparisons in future dedicated analyses, this was beyond the scope of the present study, which aimed to examine psilocybin-induced, context-dependent neural changes in adults.
Reporting on race, ethnicity, or other socially relevant groupings	Additional demographic variables (such as race, ethnicity, and education) were self-reported during screening for the broader PsiConnect dataset but were not used in the present analyses. Age and self-reported gender were used to stratify group allocation.
Population characteristics	Healthy adults aged 18–55 years (mean ± SD = 37.7 ± 10.7). N = 65 enrolled (Gender: 30 female; 34 male; 1 non-binary/gender diverse); N = 62 included in both imaging sessions (28 female, 33 male, 1 non-binary/gender-diverse). Key exclusions included current or past psychiatric disorder or suicidality; substance or alcohol use disorder within five years; first-degree relative with a psychotic disorder; major neurological disorder (e.g., stroke, epilepsy); formal meditation practice within six months or extensive prior exposure; contraindicated medications; any hallucinogen use within six months; and MRI contraindications. Informed consent was obtained from all participants prior to participation. Only three participants reported nominal or remote serotonergic psychedelic exposures.
Recruitment	Interested adults completed a brief online screening survey, followed by structured telephone screening and a psychiatric interview (SCID-5-RV) conducted by trained staff to confirm eligibility and safety. Informed consent was obtained in person before participation. Recruitment targeted healthy volunteers without formal meditation practice and without prior psychedelic experience producing subjective effects.<br><br>As a volunteer sample for a psychedelic neuroimaging study, participants may have been more motivated, curious, or comfortable with imaging and experimental (including altered state) procedures than the general population. This self-selection may limit generalisability but does not affect interpretation of within-participant effects, which form the basis of the study's inferences.
Ethics oversight	The protocol was approved by the Monash University Human Research Ethics Committee and the trial was registered with the Australian New Zealand Clinical Trials Registry (ACTRN12621001375842). All participants provided written informed consent.

Note that full information on the approval of the study protocol must also be provided in the manuscript.

Field-specific reporting

Please select the one below that is the best fit for your research. If you are not sure, read the appropriate sections before making your selection.

☒ Life sciences ☐ Behavioural & social sciences ☐ Ecological, evolutionary & environmental sciences

For a reference copy of the document with all sections, see nature.com/documents/nr-reporting-summary-flat.pdf

Life sciences study design

All studies must disclose on these points even when the disclosure is negative.

Sample size	No formal a priori power calculation was performed. A sample of 65 healthy adults was pragmatically selected to exceed the size of typical single-site psychedelic neuroimaging studies. This sample is more than twice the size of many previous studies, providing sufficient power for within-participant analyses across four experimental contexts. The dataset is openly available (OpenNeuro accession ds006110) for reproducibility and reuse for scientific discovery.
Data exclusions	At baseline (no psilocybin), 65 participants completed both MRI and EEG sessions. Two participants were subsequently withdrawn prior to the psilocybin session after meeting exclusion criteria, and their baseline data were not analysed.<br><br>During the psilocybin session, 63 participants were dosed. Of these, one did not complete the post-dose MRI or EEG, and another only partially completed the resting-state condition of the fMRI scan and did not complete the EEG. This partial resting-state fMRI was included in the analysed data. Sixty-one participants completed the full post-dose MRI session across the meditation, music, and movie conditions. Two additional participants did not complete the second EEG session.<br><br>Psilocybin session, MRI completed: 62, 61, 61, 61 (rest, meditation, music, movie)<br><br>Psilocybin session, EEG completed: 59, 59, 59, 59 (rest, meditation, music, movie)<br><br>From these numbers, quality control resulted in further exclusions. For the functional MRI images collected during baseline, there were no further exclusions. For the functional MRI images collected during psilocybin, six participants had one or more conditions excluded due to head motion and one further participant due to other quality control metrics (n=7): 2 rest, 2 meditation, 5 music, and 4 movie scans.<br><br>The final analysed MRI samples during baseline were: rest = 63, meditation = 63, music = 63, and movie = 63.<br><br>The final analysed MRI samples during psilocybin were: rest = 60, meditation = 59, music = 56, and movie = 57.<br><br>For machine-learning analyses, only scans with identical numbers of volumes per condition across participants were included.<br><br>The final analysed MRI samples for machine-learning analyses for baseline and psilocybin were: rest = 54, meditation = 54, music = 54, and movie = 54.<br><br>Technical issues accounted for EEG exclusions. Starting from 65 EEG recordings collected at baseline, two were excluded due to missing event markers. The final analysed baseline EEG data were: rest = 63, meditation = 63, music = 63, and movie = 63.<br><br>Starting from rest = 59, meditation = 59, music = 59, and movie = 59 EEG recordings collected during the psilocybin session, event markers were not recorded for one participant. The final analysed EEG data during psilocybin were: rest = 58, meditation = 58, music = 58, and movie = 58.<br><br>All exclusion criteria were pre-established or represent standard QC thresholds, and a table of recommended exclusions accompanies the data descriptor (doi.org/10.1038/s41597-026-07312-1).
Replication	Although no formal replication analysis was undertaken, we assessed the robustness and stability of the findings using the same dataset under varying analysis choices. For the fMRI BOLD signal variability analysis, applying a more stringent head-motion threshold (FD < 0.25 mm; ~six-fold greater data censoring) preserved the original spatial pattern of effects, indicating that results are not motion-driven and are robust to preprocessing variation.<br><br>For the machine-learning embeddings (CEBRA), repeated training (ten runs per participant) showed minimal run-to-run variability in silhouette scores relative to condition effects, confirming stable embeddings. Consistent context-related changes were also observed across modalities (fMRI and EEG), further supporting the stability of the results across measurement approaches.<br><br>While this work does not include formal reproducibility (same data, same analysis, independent researchers) or replicability (same analysis, independent dataset) assessments, besides rigorous robustness checks, all raw and pre-processed data are openly available (OpenNeuro ds006110) to enable such efforts by the community.
Randomization	Participants were allocated using a non-randomised, stratified-by-balance procedure. To meet scheduling and class-capacity requirements for the meditation training, individuals were placed in the meditation arm when class seats were available; otherwise, they entered the control arm. Allocation was therefore determined pragmatically rather than by random sequence.<br><br>Age and self-reported gender were monitored throughout recruitment to maintain balanced distributions across meditation intervention and no meditation intervention arms. This manual stratification ensured comparable demographic composition. Pending exclusions, all participants completed both baseline and psilocybin imaging sessions. The main analyses relied on within-participant design, further mitigating potential between-group allocation bias.<br><br>Methodological details on this allocation procedure are available in the data descriptor (doi.org/10.1038/s41597-026-07312-1) and forthcoming protocol documentation.
Blinding	The study used an open-label format to prioritise participant safety, comfort and compliance. Pre-session familiarisation with the setting and staff was used to prepare participants who would undergo first-time psychedelic experience in an MRI and EEG environment. This approach also reflected evidence that moderate- to high-dose psilocybin produces distinct perceptual and physiological changes, albeit with individual variability in intensity, and the current lack of a suitable active placebo capable of maintaining credible blinding under these conditions.

To minimise expectancy-related influences, all instructions and preparatory procedures were standardised across participants, and the main neuroimaging analyses relied on within-participant contrasts between baseline and psilocybin sessions.

Reporting for specific materials, systems and methods

We require information from authors about some types of materials, experimental systems and methods used in many studies. Here, indicate whether each material, system or method listed is relevant to your study. If you are not sure if a list item applies to your research, read the appropriate section before selecting a response.

Materials & experimental systems

n/a	Involved in the study
☒	☐ Antibodies
☒	☐ Eukaryotic cell lines
☒	☐ Palaeontology and archaeology
☒	☐ Animals and other organisms
☐	☒ Clinical data
☒	☐ Dual use research of concern
☒	☐ Plants

Methods

n/a	Involved in the study
☒	☐ ChIP-seq
☒	☐ Flow cytometry
☐	☒ MRI-based neuroimaging

Clinical data

Policy information about clinical studies

All manuscripts should comply with the ICMJE guidelines for publication of clinical research and a completed CONSORT checklist must be included with all submissions.

Clinical trial registration

The study was registered with the Australian New Zealand Clinical Trials Registry (ACTRN12621001375842).

Study protocol

All key protocol and methodological details are described in the current manuscript and the associated data descriptor (doi.org/10.1038/s41597-026-07312-1).

Data collection

Data were collected at the Turner Institute for Brain and Mental Health, Monash University (Melbourne, Australia) between September 2021 and December 2023. Participants underwent baseline and psilocybin imaging sessions, each including multi-echo fMRI and 64-channel EEG, under four conditions (eyes-closed rest, meditation, music, and eyes-open movie viewing). Clinical and behavioural data were acquired using standardised questionnaires before, during, and after dosing. All imaging and behavioural data were stored in BIDS format and are available at OpenNeuro (ds006110).

Outcomes

The primary outcomes were pre-defined as changes in large-scale brain connectivity and activity patterns between baseline and psilocybin sessions, assessed using multi-echo fMRI and EEG across multiple contexts (eyes-closed rest, meditation, music, andeyes-open movie). fMRI outcomes were quantified from pre-processed time-series data using voxel- and network-level analyses of global functional connectivity, BOLD signal variance, functional modularity, and effective connectivity estimated with spectral Dynamic Causal Modelling (DCM). EEG outcomes were derived from cleaned data to estimate spectral power, peak-alpha frequency, and state-level dynamics.

Additional machine-learning analyses characterised the organisation of brain activity across conditions using Contrastive EmBedding Representation Analysis (CEBRA) and Temporal-Aware Variational Recurrent Neural Networks (TAVRNN), which generated low-dimensional embeddings and modelled context-dependent neural organisation.

Secondary outcomes were subjective experiential measures obtained immediately post-session, including the Mystical Experience Questionnaire (MEQ30) and the Altered States of Consciousness Scale (ASC). These were used to relate variability in neural reorganisation to the qualitative features of the psychedelic experience.

All outcome definitions, acquisition parameters, and analytic pipelines are described in the manuscript Methods.

Plants

Seed stocks

Report on the source of all seed stocks or other plant material used. If applicable, state the seed stock centre and catalogue number. If plant specimens were collected from the field, describe the collection location, date and sampling procedures.

Novel plant genotypes

Describe the methods by which all novel plant genotypes were produced. This includes those generated by transgenic approaches, gene editing, chemical/radiation-based mutagenesis and hybridization. For transgenic lines, describe the transformation method, the number of independent lines analyzed and the generation upon which experiments were performed. For gene-edited lines, describe the editor used, the endogenous sequence targeted for editing, the targeting guide RNA sequence (if applicable) and how the editor was applied.

Authentication

Describe any authentication procedures for each seed stock used or novel genotype generated. Describe any experiments used to assess the effect of a mutation and, where applicable, how potential secondary effects (e.g. second site T-DNA insertions, mosiacism, off-target gene editing) were examined.

Magnetic resonance imaging

Experimental design

Design type	Within-participant block design across four naturalistic contexts (rest, meditation, music, movie) with minimal instruction and no overt responses.
Design specifications	Two sessions per participant (baseline [no psilocybin] and with psilocybin).<br>Run durations/volumes: rest = 8:00 (505 vols), meditation = 6:30 (405 vols), music = 11:24 (728 vols), movie = 6:00 (372 vols).
Behavioral performance measures	No behavioral responses were recorded during imaging. Compliance was monitored via eye-monitoring to ensure compliance with eyes-closed (rest, meditation, music) and eyes-open (movie viewing) imaging.

Acquisition

Imaging type(s)	Functional, structural, diffusion MRI.
Field strength	3 Testla (Siemens MAGNETOM Skyra).
Sequence & imaging parameters	Functional BOLD (multi-echo EPI): TR 910 ms; TEs 12.60/29.23/45.86/62.49 ms; flip 56°; multiband 4; 40 axial slices; 3.2 mm isotropic; matrix 64×64; FoV 206 mm; phase-encode right-to-left; interleaved. Reverse-phase EPI (~10 vols) acquired for susceptibility distortion correction.<br>Structural T1-weighted (MPRAGE): TR 2300 ms; TE 2.07 ms; TI 900 ms; flip 9°; 1.0 mm isotropic; 192 slices; FoV 256 mm.<br>Diffusion EPI: TR 5900 ms; TE 171 ms; 2.5 mm isotropic; 56 slices; FoV 212 mm (read), 100% phase; GRAPPA PE = 2; both phase-encode directions acquired.
Area of acquisition	Whole brain.

Diffusion MRI ☒ Used ☐ Not used

Parameters: 71 directions; b = 0 and 3000 s/mm²; single-shell (one non-zero shell); no cardiac gating.

Preprocessing

Preprocessing software	fMRIPrep 22.0.2, using ANTs 2.3.3 (antsRegistration/antsApplyTransforms), FSL 6.0.5.1 (mcflirt/FAST), FreeSurfer 7.2.0 (bbregister, recon-all), AFNI 3dTshift, and Nilearn 0.9.1 within fMRIPrep.
Normalization	Volume-based nonlinear spatial normalisation to MNI152NLin2009cAsym with ANTs (antsRegistration); resampling performed in a single interpolation step with Lanczos when composing transforms.
Normalization template	ICBM 152 Nonlinear Asymmetrical 2009c (TemplateFlow ID: MNI152NLin2009cAsym).
Noise and artifact removal	Multi-echo combination (voxelwise T2* map + optimal echo-weighting), followed by ME-ICA–based denoising; nuisance regressors (FD, DVARS, global/WM/CSF signals; non-BOLD components identified via ME-ICA) are provided for downstream modelling. No global signal regression and no high-pass filtering were applied in the "cleaned" derivatives.
Volume censoring	No scrubbing/censoring performed in preprocessing. Motion metrics (FD, DVARS) are supplied.

Statistical modeling & inference

Model type and settings	Whole-brain: parcel-wise functional connectivity (332-parcel cortical + subcortical atlas); within-/between-network FC; modularity analyses; non-parametric permutation testing; and ROI/effective-connectivity analyses using spectral DCM.
Effect(s) tested	Drug (psilocybin vs baseline), stimulus context (rest, meditation, music, movie), and drug×context interactions on FC; effective-connectivity differences were assessed with DCM (model inversion and group comparison using parametric empirical Bayes, PEB).

Specify type of analysis: ☐ Whole brain ☐ ROI-based ☒ Both

Anatomical location(s): DCM ROIs (6-mm spheres; MNI): L aHPC (−26, −16, −20); R aHPC (28, −16, −20); L IPC (−44, −60, 24); R IPC (54, −62, 28); mPFC (2, 56, −4); PCC (2, −58, 30).

Statistic type for inference<br>(See Eklund et al. 2016)	Whole-brain parcel analyses: permutation-based tests (Mann–Whitney U on parcel metrics where applicable).<br>ROI-DCM: Bayesian using Parametric Empirical Bayes.
Correction	Whole-brain: permutation-based TFCE (PALM) with family-wise error control. Parcel-wise tests: Bonferroni across parcels/networks (analysis-level control).<br>ROI-DCM: inference via PEB posterior-probability thresholds.

Models & analysis

n/a	Involved in the study
☐	☒ Functional and/or effective connectivity
☐	☒ Graph analysis
☐	☒ Multivariate modeling or predictive analysis

Functional and/or effective connectivity

Functional connectivity analysis parcellated denoised fMRI time series into 332 regions (Schaefer cortical 300 + Melbourne subcortical atlas, 32). Representative parcel time series were the first principal component. Parcel-wise FC was computed with Pearson's correlation (Fisher-z for statistics), and within-/between-network FC were derived by averaging the relevant matrix blocks. Group comparisons used right-tailed Mann–Whitney U on parcel metrics. Effective connectivity was analysed with spectral DCM and group-level PEB.

Graph analysis

Modularity was computed on parcel-wise functional connectivity networks using the Brain Connectivity Toolbox. Reported at the group level.

Multivariate modeling and predictive analysis

Low-dimensional embeddings of parcel fMRI time series used CEBRA and trained an SVM to classify condition at each time point. Performance was assessed by accuracy and related to subjective experience via Pearson correlations with 11D-ASC, MEQ30, and Mindset scores.
