# Supplementary material for: Psychedelics align brain activity with context
Source: Nature. 2026 Aug 19;656(8129):936–47. doi: 10.1038/s41586-026-10910-z (PMC13518247; doi:10.1038/s41586-026-10910-z)
Supplement: Supplementary file 3 [file 41586_2026_10910_MOESM3_ESM.pdf]

nature portfolio

Peer Review File

Psychedelics Align Brain Activity with Context

Corresponding Author: Professor Adeel Razi

Any redactions in this file are there to maintain patient confidentiality, the confidentiality of unpublished data, or to remove third-party material.

This file contains all reviewer reports in order by version, followed by all author rebuttals in order by version.

Attachments originally included by the reviewers as part of their assessment can be found at the end of this file.

Version 1:

Reviewer comments:

Referee #1

(Remarks to the Author)
Recap and key results
This manuscript reports the largest single-site neuroimaging study of psilocybin to date, integrating functional MRI, high-density EEG, and rich behavioral phenotyping in a cohort of roughly 60 participants. By manipulating the sensory-cognitive context—eyes-closed rest, guided meditation, music listening, and naturalistic movie viewing—the authors demonstrate that psilocybin changes large-scale brain networks in a context-dependent manner. Under eyes-closed conditions the drug suppresses global functional connectivity in primary sensory cortices while amplifying connectivity in dlPFC and some other areas; during eyes-open movie viewing there appears to be widespread increase in global connectivity (or more precisely, there is a widespread decrease in GFC during movie viewing that is no-longer seen during movie viewing on psilocybin). Histograms show that the usual separation between eyes-open and eyes-closed connectivity distributions is reduced by 85 %, which is interpreted as a convergence of internal and external brain states . Machine-learning approaches—contrastive embedding for brain activity (CEBRA) and a temporal-attention variational graph RNN (TAVRNN)—reveal low-dimensional latent trajectories that correlated with subjective experience. These fMRI findings are corroborated by EEG, which shows context and frequency-specific desynchronisation (θ/α/β power decreases with a γ increase). I find it particularly fascinating and important that drug-baseline differences seem to be much smaller in the movie watching condition compared to the others.
The work stands out for its unusually large sample size in psychedelic research and for its multimodal design—coupling fMRI, EEG, and behavioral measures—to allow cross-validation of neural signatures across measurement scales. By explicitly manipulating sensory and cognitive context, the authors address long-standing questions about “set and setting” in a controlled experimental framework.

Major concerns / criticisms

• Opacity of CEBRA and TAVRNN analyses.
o The central claim of novelty of the results is “Using CEBRA-derived machine learning embeddings, we reveal clear order in the patterns that occur when individuals undergo deeply meaningful self- and boundary-dissolving experiences.” However, the manuscript gives only cursory method descriptions or justification and does not adequate explain what the results (‘low-dimensional latent trajectories that correlated with subjective experience’) mean in the language of neuroscience or how we should understand them. Readers unfamiliar with these complex algorithms will struggle to follow why they were chosen, how they outperform simpler approaches, and what concrete biological inferences should be drawn from their latent spaces. More intuitive schematics and a step-wise rationale (input, loss, validation, interpretation) may help make this accessible for a general neuroscience audience.
o CEBRA classification accuracy was correlated with subjective ratings. However I am having trouble understanding how to interpret CEBRA classification accuracy.
o I take issue with claiming that your metric of CEBRA classification accuracy predicts next-day Mindset change (r=0.4). You should that components of the 11D-ASC predict next day mindset with r=0.6. And that CEBRA classification accuracy predicts components of the 11D-ASC. So I would guess that any relationship between CEBRA classification and next day mindset is simply mediated by both being correlated with 11D-ASC scores.

o I cant figure out what is being shown in Fig. 3d. The plots do not appear to have axis labels.

• Order effects, lack of counterbalancing of conditions. It appears that the four conditions were always in the same order - eyes-closed rest, guided meditation, music listening, then movie. Thus, condition differences are confounded by time/PK/fatigue differences.
• When was the fMRI started? I can not find it stated anywhere how long after drug ingestion the MRI began.
• Dynamic-causal-model - It is not clear to me how this fits with the other observations made in the paper. There is already a lot of content, perhaps this would be better of in a separate manuscript to allow space for other interesting results in this manuscript to shine (for example I would love to see more interpretation of the collapse of GFC differences between rest vs movie watching).
• Motion confounds. There does not appear to be any attempt to report or account for differences in head motion between baseline and psilocybin condition. This is almost certainly the reason why there are large increases in SD in the temporal pole and orbital frontal cortex on psilocybin (Fig 2c) as these are parts of the brain next to sinuses where there is classically the largest motion-correlated confounds. The methods state “7 subjects have one or more condition excluded from the analysis…most corresponded to frame-wise displacement greater than 0.5” This is a fairly loose motion threshold, particularly if there is no censoring of high motion frames prior to analysis.
• Embeddings and “embeddedness.” It is not clear if the authors intend to suggest a link between the ML concept of low-dimensional embeddings and the psychological construct of embeddedness. In the best case, the repeated use of the two terms is confusing.
• Lack of a placebo-controlled or double-blind design. The open-label approach heightens expectancy and demand characteristics, which could inflate subjective scores and neural changes.

Minor concerns / suggestions

• Figure 6c is labeled as “Lempel-Ziv\ complexity difference (psilocybin minus baseline (no-psilocybin))” but I think it may be power spectra (the y axis is power).
• Clarify demographic information (average age, gender ratio, exclusion criteria) in the main text.
• Fig. 4g Caption – “Mutual exclusivity” implies a negative correlation, when in fact no significant correlation was found.
• Text/legends in multiple figures is so tiny that it is impossible to read (e.g. network labels Fig. 2b, all labels in Fig. 6). It appears to be 2.5 point font.
• Consider adding citation for Goodwin et al., 2025 “The role of the psychedelic experience…” at the end of first paragraph on page 8.
• Please define any abbreviations or nonstandard labels in the figure captions (e.g “salventattn”).
• Regarding difference in GFC between eyes open/closed, would cite Wong et al., 2013 Neuroimage.
• Fig 5 – The text references panels a-d however there are no panel labels in the figure.
• Correct minor typos “anti-depresssant” on page 13
• Replace “available upon request” with a DOI-linked repository in the data-availability statement.

Referee #2

(Remarks to the Author)
Summary: This is an ambitious open-label study in which the effects of psilocybin were investigated in a comparatively large sample of healthy volunteers (n = 60), using four distinct experimental conditions: resting state, meditation, listening to music, and watching a video. The study employed both fMRI and EEG to characterize psilocybin's effects on brain function. The paper presents a complex set of analyses. First, the authors demonstrate changes in GFC, showing that psilocybin reduces differences between eyes-closed and eyes-open conditions. Using machine learning approaches, they further show that psilocybin induces a restructuring of brain dynamics in a context-sensitive manner—i.e., aligning neural activity more closely with the sensory and psychological environment.
This work is important in that it offers a shift in framing: from the view that psychedelics merely desynchronize brain activity (e.g., via increased entropy), to a more nuanced interpretation emphasizing alignment of neural dynamics with contextual and experiential factors. This reframing holds potential implications for our understanding of the therapeutic mechanisms of psychedelics.

The authors may wish to address the following points, which would improve clarity, enhance interpretability, and increase the manuscript’s overall readability. The points are listed in no particular order of importance.

Major points:

1. The authors should clarify that the EEG session followed the fMRI session, including in the abstract. Additionally, it remains unclear how much time elapsed between drug administration and the start of the fMRI and EEG recordings, respectively. This information could be added to Figure 1 and described in the Methods. Notably, the timing of the EEG session is not discussed anywhere, which is important given that delayed effects might be observed.

2. In the Main section (page 2), the authors write that “recent human research suggests that reductions in DMN and anterior hippocampus connectivity may contribute to the therapeutic effects of psilocybin,” but then cite Reinwald (rodent study) and Siegel (healthy participants). These references do not provide direct evidence for the stated claim. Rephrasing would be advisable.

3. Regarding psilocybin's contribution to therapeutic effects, the observed reductions in modularity are not discussed in relation to prior work, particularly the study by Daws et al. (10.1038/s41591-022-01744-z), which linked decreased modularity with symptom improvements in patients with depression.

4. Participant demographics: Mean age and sex distribution should be reported alongside the age range.

5. The rationale for excluding participants who used hallucinogens within the past 6 months is unclear if the sample was already restricted to psychedelic-naïve individuals. Clarification would be helpful.

6. In the Participants section (pg 12), the phrase "individual experience" appears, but the section primarily lists adverse effects. It may be clearer to rephrase this to something like: "Psilocybin was generally well tolerated, although some adverse effects were reported, including…". This would align better with the tone typically used in clinical trial reporting.
7. Extended Data Fig. 2 shows mean FD for the music and movie conditions only. Why are rest and meditation not included? Understanding whether head motion differed across all conditions is essential for interpreting functional connectivity results.

8. The order of conditions differs between fMRI and EEG sessions—ending with movie watching for fMRI and beginning with it for EEG. This difference warrants clarification and possibly justification.

9. In the Integration section (pg 3), it would improve clarity to specify in brackets which conditions correspond to "eyes-closed states." The finding that occipital areas show the strongest GFC decreases is difficult to interpret without knowing the specific condition(s). Structuring the description of results across eyes-closed conditions (rest, meditation, music) and providing concrete examples for each condition, as is done for EEG, would enhance readability.

10. When discussing GFC findings, the authors may wish to mention other studies using similar metrics such as global correlation (10.1111/acps.12818; 10.1016/j.euroneuro.2021.06.001), degree centrality (10.1038/s41380-024-02734-y, with and without GSR), or functional connectivity density (10.1016/j.cub.2016.02.010). Contextualizing findings is important, especially as the literature is limited.

11. The manuscript highlights reduced within-network connectivity in the DMN and dorsal attention network (pg 4), but visually, the most pronounced effect appears in the VIS (Figure 2b). This discrepancy should be addressed.

12. The lack of differences between the meditation and control group is intriguing, particularly in light of previous work reporting differences (10.1016/j.neuroimage.2019.04.009, 10.1038/s41598-024-55726-x). Even a brief discussion of potential synergistic or non-additive effects would be valuable.

13. How do the current EEG results (power spectrum and LZ complexity) compare to those reported by Timmermann et al. (10.1073/pnas.2218949120) for DMT under resting conditions? A short comparison would help situate the findings.

14. The following sentence (pg 10) seems ambiguous and may overreach given the data presented:
This pattern supports interpreting embeddedness as a state central to psychedelics' therapeutic relevance by addressing distress arising from separation, which may underlie the therapeutic efficacy of psychedelics in reducing existential distress (Griffiths et al., 2016; Ross et al., 2016).
It is unclear what is meant by separation (from what?). The interpretations should be revised.

15. In the Context shaped connectivity section (pg 11), the authors interpret the findings as reflecting reduced sensory and increased associative integration. It would be useful to comment on the specificity of these patterns, especially in light of recent work suggesting that similar effects may not be unique to psychedelics (e.g., 10.1038/s41380-024-02734-y).

16. It is notable that the movie condition increased GFC yet showed reduced EEG power effects. This raised the question of "grounding," as discussed in Siegel et al. (10.1038/s41586-024-07624-5) although this does not align neatly with the fMRI results. The authors might wish to comment on this divergence.

17. The authors should consider speculating on how psychedelic-induced therapeutic effects might be interpreted in light of this new framework. So far, psychedelics have been thought to desynchronize brain activity, temporarily loosening canonical networks and allowing for novel configurations to emerge. This paper challenges that view by demonstrating that psilocybin reorganizes neural dynamics in a context-sensitive manner—suggesting a flexible reorientation toward both external stimuli and internal states. This raises the intriguing possibility that therapeutic effects may stem not merely from a dismantling of existing patterns, but from the brain's enhanced ability to dynamically align with salient contextual or experiential inputs. Such flexible realignment could be particularly relevant in the context of psychedelic-assisted psychotherapy.

Minor points:

1. On pg 13 under Reported measures, remove "(n=)" from the sentence:
"If they responded yes (n=), they were then asked…"

2. On pg 13 in the MRI acquisition section: Why was the GRAPPA acceleration factor changed from 2 (baseline) to 3 (psilocybin session)? Please clarify.

3. For Extended Data Fig. 1: the legend should include the symbols used to denote conditions and L/R for hemispheres.

4. On pg 16 in the section Tavrrn "FC" is introduced but the abbreviated form has already been used several times before this section – e.g., Parcel-wise FC.

5. Presumably a typo on pg 6: "¿9/10" – please correct.

Referee #3

(Remarks to the Author)
I co-reviewed this manuscript with one of the reviewers who provided the listed reports.

Referee #4

(Remarks to the Author)
Psychedelics modulate perception and self-awareness by disrupting the brain's integration of internal predictive models with external sensory information, a process thought to be primarily mediated through agonism of the serotonin 5-HT2A receptor. This disruption is claimed to induce functional plasticity and reconfiguration of brain connectivity, particularly within and between associative and sensory networks. These changes (under certain controlled conditions) are often experienced as deeply meaningful and therapeutically beneficial. Consequently, emerging evidence indicates clinically meaningful reductions in symptoms associated with depression, anxiety, and substance use disorders. Leading theoretical frameworks suggest that psychedelics exert their effects by relaxing hierarchical constraints within the heteromodal and highly integrative default mode network (DMN). This disinhibition is thought to facilitate the emergence of novel patterns of functional connectivity, which may underlie the immersive and boundary-dissolving nature of the psychedelic experience.

Psychedelic studies often suffer from small sample sizes due to regulatory and logistical constraints. A dataset with 62 participants presented here, especially with no prior psychedelic use, provides statistical power and generalisability that are uncommon in the field. The authors also incorporated a range of contextual conditions—including rest, meditation, music listening, and movie viewing—which provides valuable opportunities to investigate how different environmental and cognitive contexts modulate the psychedelic experience. Combining fMRI and EEG data allows the authors to present results from slow, large-scale network changes (seen in fMRI) with fast oscillatory dynamics (seen in EEG), which is useful for understanding altered states of consciousness. So overall a unique dataset with huge potential that will surely materialise when the data will be made "fully open access through an OpenNeuro repository upon publication". For this reason alone, the manuscript deserves publication.

Beyond this, the authors also present evidence that under psilocybin, brain dynamics shift toward greater integration of anterior-associative processes, especially during positively felt states. This reorganisation emerges according to the authors, as a context-sensitive, psychologically adaptive neural signature, offering insight into how psychedelics may facilitate therapeutic transformation through altered brain network architecture.

The manuscript is well composed, with a solid narrative and a commendable integration of complex methodologies. However, the interpretation of the findings occasionally drifts into overly ambitious territory, where the prose tends to outpace the empirical findings. While the concept of "embeddedness" is intriguing and the contextual sensitivity of the psychedelic state is well-argued, some of the conclusions are reaching beyond what the data can firmly support. I will try to present some examples of this in my detailed comments below.

The statistical robustness of the results presented in this study is strong, reflecting both methodological rigor and the advantages of a large, well-controlled sample. Across modalities (fMRI, EEG, ML embeddings), the authors report significant effects with solid effect sizes. The use of Bayesian model averaging, non-parametric tests (e.g., Mann-Whitney U), and machine learning classification accuracy further strengthens the inferential validity. Importantly, the authors also address potential confounds (e.g., head motion, global signal regression) and demonstrate replicability across conditions and individuals, enhancing confidence in the generalisability of their findings.

Specific comments:

1. Were the multiple fMRI echos combined into one set of images? How did the authors ensure that the components identified and removed were indeed noise, rather than neuronal signals of interest? To my knowledge, Kundu et al. recommend the use of ICA for denoising; however, within this experimental framework, there is a risk that meaningful signal —such as a potential subdivision of the default mode network (or any other network) following psilocybin administration— could be misclassified as noise, particularly if one subnetwork appears smaller or less spatially extensive. Was any quality control performed to mitigate this risk, such as visual inspection of removed components or analysis of their spectral characteristics?

2. Did the authors use any temporal filtering for the fMRI data? High-pass filtering is commonly used in task-fMRI to remove low frequency scanner drifts for example. Did the denoising process used remove particularly high or particularly low

frequencies?

3. Page 2: “Psychedelics such as psilocybin disrupt these inter- actions by acting as an agonist at the serotonin 5-HT2A receptor, inducing structural and functional plasticity.” Please include some details on structural plasticity.

4. Page 2: “…research suggests that reductions in DMN and anterior hippocampus connectivity may contribute to the therapeutic effects of psilocybin (Siegel et al., 2024; Rein- wald et al., 2023). Are the authors suggesting this to be an overarching mechanism or particular to a specific disorder?

5. Page 3: Section on “Spatially and context-dependent increase of BOLD signal variance”. Why do they authors compute standard deviation but really discuss entropy? I am not sure that the references in this paragraph associate variability with entropy “Increased BOLD signal variability under psychedelics has been commonly associated with increased entropy (Carhart-Harris et al., 2014; Herzog et al., 2023; Viol et al., 2019)”.

6. Page 6: “These findings suggest embeddings are indicative of the profound transformative qualities of the psychedelic experience unique to serotonergic psychedelics.” I find this statement challenging to reconcile with the presented evidence. There wasn’t any profoundness statistically tested here and neither were non-serotonergic psychedelics. Furthermore, that particular paragraph repeats the idea that classification accuracy tracks subjective experience multiple times, without adding new information.

7. Page 9. The Precuneus is considered part of the DMN and it’s highly relevant in this study (e.g. Lyu D,…Parvizi J Neuron 2023) why omit it from the DCM model? Also not clear whether the authors modelled many to many interactions in their model or a predetermined set of hypothesis driven interactions.

8. Why was a 3D latent space chosen for CEBRA embeddings? Was this empirically validated (e.g., via explained variance or classification performance)?

9. Were multiple runs of CEBRA performed to assess the stability of embeddings?

10. In the part of the manuscript the authors explain TAVRNN they state: “In this work, each scan under a specific condition (rest, meditation, music, or movie) is treated as a temporal snapshot. Therefore, the attention mechanism enables the lower-dimensional representation of one scan to inform the representation of the subsequent scans.” Was there any segmentation of the timeseries for TAVRNN input e.g. overlapping windows, or condition-averaged matrices? I am not sure how the 2D embeddings of individual brain regions were interpreted? clustering to relate them to subjective or behavioural data?

11. Am I mistaken in thinking that the TAVRNN framework is best implemented with EEG data because of its superior temporal resolution?

12. Page 12: “Our decision to administer a standardised dose of 19 mg psilocybin, rather than a body-weight-adjusted dose was based on previous research showing limited benefits of body-weight-adjusted dosing (Garcia-Romeu et al., 2021). " What does limited benefits mean? Was it in terms of efficacy, safety, variability, or practicality?

13. Fig. Extended Data 1 shows increases and decreases but not clear under what conditions.

Version 2:

Reviewer comments:

Referee #1

(Remarks to the Author)
I think the finding that psilocybin greatly reduces the usual separation between eyes-open and eyes-closed fMRI and EEG measures is quite interesting.

Overall, I find myself unsatisfied with the other main finding - a correlation between A) run separability by an SVM and B) intensity of subjective experience. I'm not sure if that correlation constitutes a substantive advance, much less “a new framework for understanding how psychedelics shape neurobiology.” Perhaps further and better analysis could be done to understand what ARE the differences between conditions (rest, meditation, music, movie) that cause them to separate with CEBRA.

I understand that stronger and more meaningful psychedelic experiences correlate with a larger difference between runs (and perhaps condition, although I’m not sure this is clearly shown). But what is that difference?

As far as I can tell from Fig 1, average FC difference look pretty similar across rest/meditation/music. The only manipulation that seems to have appreciable differences is eyes closed vs open (i.e. movie watching). Although that seems to be primarily because it is different at baseline, which becomes less prominent on psilocybin. But I think this is the opposite of the main claim the authors are making that "CEBRA classification accuracy is interpretable as a marker of differentiation between brain states across contexts."

Even if we accepted that psychedelics increase differences in FC between contexts, I'm not sure this would be interpretable as a neurobiologically meaningful insightful (without some idea of what of where those differences are). I believe the CEBRA models were trained separately for each participant so in fact there is no way to ascertain meaningful insights about whether psilocybin has any context/condition-specific effects (as the authors seem to claim) much less what those effects might be. They have simply shown that participants who had high MEQ scores showed more 'change' in BOLD signals across four scans during their psilocybin visit. This is not a novel insight and it does not justify the claims about 'embeddedness.'

The authors state in R2R "we rely on our conventional analyses (e.g. GFC and DCM), which are more directly tied to underlying physiology." But I am not aware of any evidence tying GFC to any physiological process.

MINOR:
1.1 – were SVM classification scores higher overall during psilocybin than no drug?

1.4 - TAVRNN – "Simultaneously, embeddings across all networks showed tighter clustering" where is this shown?

1.8 It is nice to see that the authors reran processing with more stringent criteria, however the appropriate test of whether increases in SD in the temporal pole and orbital frontal cortex is associated with motion would be the correlation between framewise displacement and SD in those areas (as has been published before and is generally attributed to the interaction of susceptibility artifact with motion), not weather the spatial pattern disappears with more stringent exclusion. A model in which FD is used as a covariate is preferred.

I'm unclear as to whether Extended Data 2 shows FD during psilocybin or baseline. Please show both if possible.

Referee #2

(Remarks to the Author)
The authors have adequately addressed all issues raised by the reviewer.

Referee #3

(Remarks to the Author)
I co-reviewed this manuscript with one of the reviewers who provided the listed reports.

Referee #4

(Remarks to the Author)
The authors did an excellent job addressing most of my questions thoroughly and thoughtfully. Their explanations were clear and well-supported. However, there is one point that would benefit from further clarification to avoid ambiguity.

In their response to my question about temporal filtering of the fMRI data (4.2) the authors state that "This regression step naturally eliminates very low-frequency artefacts without requiring an explicit high-pass filter." And "ICA effectively suppresses non-neuronal low-frequency fluctuations while preserving the broad spectral range of BOLD signal. For clarity, we reproduce the relevant figure here (See Fig. R9)". However, plot R9 is not a frequency plot. It is a plot of global signal change over a 500-volume acquisition. Can the authors please provide a frequency plot to support their argument?

Additionally, in response to my question about the precuneus (4.5), the authors note that "the PCC and precuneus are anatomically adjacent and partially overlapping." This statement does not appear in the main text, so it is not a major concern, however, for the sake of precision, it is worth clarifying that, in terms of cytoarchitecture, the PCC and precuneus are distinct regions.

Version 3:

Reviewer comments:

Referee #1

(Remarks to the Author)
The added analyses (particularly the network-attributed decomposition) strengthen the manuscript and improve the interpretability of the CEBRA findings. At the same time, I still think the central result remains difficult to interpret neurobiologically. As currently presented, a highly complex set of time series data from multiple runs is entered into a

machine-learning pipeline, which yields a separability metric that then correlates with MEQ scores. This is interesting and may indeed capture meaningful structure in the data, but it does not yet make clear which specific temporal, spatial, or network-level features of the BOLD signal are driving the effect, nor what neurobiological process should be inferred from it. For that reason, I would encourage the authors to soften some of the stronger interpretive language. In particular, I do not think the present data clearly establish that psychedelics “align brain activity with context” (title) or that the identified patterns “reflect the quality of subjective experience” (abstract) in a mechanistic sense. A more cautious framing would better match the evidence provided.

Two related concerns illustrate the limitations to interpretation. First, as noted in the prior reviews, the interpretation of the CEBRA/SVM findings as context-specific reorganization still seems difficult to disentangle from time-on-drug effects. In the fMRI session, conditions were acquired in a fixed order (rest → meditation → music → movie) beginning approximately 80 minutes post-dose, and the model was trained on concatenated time series from these consecutive runs. As a result, condition is confounded with time within session, and with changing drug level or effect near peak. The authors show greater clustering/separability under psilocybin, which is intriguing, but I do not think they have ruled out the possibility that this effect reflects temporal nonstationarity in drug effects rather than condition-specific reorganization per se. If additional analyses cannot address this directly, this should be acknowledged explicitly as a limitation.

Second, the reduction in separation between eyes-closed and eyes-open states may be vulnerable to a vigilance-state confound. The key comparison contrasts three eyes-closed conditions (rest, meditation, music) with an eyes-open movie condition, but I do not find a description of direct vigilance or sleep monitoring during the fMRI scans. Given that drowsiness and sleep occurs during >40% of eyes-closed resting scans, it seems important to clarify whether the authors can exclude the possibility that sleepiness or sleep contributed to the observed convergence. If this cannot be ruled out, it should be acknowledged as a limitation in the manuscript.

Overall, I think the manuscript contains an interesting and valuable dataset, and the additional analyses have improved it. My suggestion is not necessarily that the authors must fully resolve each of these issues analytically, but rather that they should adopt a somewhat more cautious interpretation and acknowledge limitations.

Open Access This Peer Review File is licensed under a Creative Commons Attribution 4.0 International License, which permits use, sharing, adaptation, distribution and reproduction in any medium or format, as long as you give appropriate credit to the original author(s) and the source, provide a link to the Creative Commons license, and indicate if changes were made.
In cases where reviewers are anonymous, credit should be given to 'Anonymous Referee' and the source.
The images or other third party material in this Peer Review File are included in the article’s Creative Commons license, unless indicated otherwise in a credit line to the material. If material is not included in the article’s Creative Commons license and your intended use is not permitted by statutory regulation or exceeds the permitted use, you will need to obtain permission directly from the copyright holder.
To view a copy of this license, visit https://creativecommons.org/licenses/by/4.0/

Psychedelics Align Brain Activity with Context

Response to Reviewers for MS-ID: 2025-04-08374

Dear Reviewers,

We are grateful for your thoughtful and comprehensive reviews. In response, we conducted several additional analyses to test the robustness of our results against the core concerns raised: order effects, motion confounds, and analytical choices. We find that our conclusions remain well supported.

In brief: (i) our findings are robust to head motion, as shown using a framewise displacement (FD) threshold of 0.25 (approximately 2× more stringent censoring; ∼6× more data excluded; Fig. R5) and persist across leading ML methods (Fig. R1) and retraining iterations (Fig. R10); (ii) we more clearly articulate the structured embeddings as vector points in learned embedding spaces and show that they emerge in the first scan—before order is introduced—and only under strong self- and boundary-dissolving subjective effects (Fig. R4a,b). They are absent at baseline and in low MEQ participants and exhibit no monotonic trends across condition order (Fig. R4c,d; Fig. R8; response 1.5); (iii) We demonstrate that individual pharmacokinetic variability distributes peak subjective effects across participants, while placebo controls are ineffective and macrodose subjective and neural effects are distinctly tied to psilocybin presence (response 1.10); (iv) We have clarified the analytic framework (Fig. R2) and made targeted revisions to ensure the interpretations are commensurate with the data, while addressing all reviewer comments. These changes substantively strengthen the central finding that psilocybin dynamically reorganises brain activity according to contextual and experiential factors.

Referee 1

This manuscript reports the largest single-site neuroimaging study of psilocybin to date, integrating functional MRI, high-density EEG, and rich behavioral phenotyping in a cohort of roughly 60 participants. By manipulating the sensory-cognitive context—eyes-closed rest, guided meditation, music listening, and naturalistic movie viewing—the authors demonstrate that psilocybin changes large-scale brain networks in a context-dependent manner. Under eyes-closed conditions the drug suppresses global functional connectivity in primary sensory cortices while amplifying connectivity in dlPFC and some other areas; during eyes-open movie viewing there appears to be widespread increase in global connectivity (or more precisely, there is a widespread decrease in GFC during movie viewing that is no-longer seen during movie viewing on psilocybin). Histograms show that the usual separation between eyes-open and eyes-closed connectivity distributions is reduced by 85 percent, which is interpreted as a convergence of internal and external brain states . Machine-learning approaches—contrastive embedding for brain activity (CEBRA) and a temporal-attention variational graph RNN (TAVRNN)—reveal low-dimensional latent trajectories that correlated with subjective experience. These fMRI findings are corroborated by EEG, which shows context and frequency-specific desynchronisation ($\theta/\alpha/\beta$ power decreases with a γ increase). I find it particularly fascinating and important that drug-baseline differences seem to be much smaller in the movie watching condition compared to the others. The work stands out for its unusually large sample size in psychedelic research and for its multimodal design—coupling fMRI, EEG, and behavioral measures—to allow cross-validation of neural signatures across measurement scales. By explicitly manipulating sensory and cognitive context, the authors address long-standing questions about "set and setting" in a controlled experimental framework.

Authors' Response:

We are thankful to the reviewer for summarising our main findings and for the very positive appraisal of our work.

Major concerns / criticisms

1.1. *The central claim of novelty of the results is "Using CEBRA-derived machine learning embeddings, we reveal clear order in the patterns that occur when individuals undergo deeply meaningful self- and boundary-dissolving experiences." However, the manuscript gives only cursory method descriptions or justification and does not adequate explain what the results ('low-dimensional latent trajectories that correlated with subjective experience') mean in the language of neuroscience or how we should understand them. Readers unfamiliar with these complex algorithms will struggle to follow:* ***a)*** *Why they were chosen.* ***b)*** *How they outperform simpler approaches.* ***c)*** *What concrete biological inferences should be drawn from their latent spaces.* ***d)*** *More intuitive schematics and a step-wise rationale (input, loss, validation, interpretation) may help make this accessible for a general neuroscience audience.*

Authors' Response:

We thank the reviewer for this helpful and important feedback. We have now substantially revised the manuscript to clarify the rationale, accessibility, and interpretability of our machine learning methods. Below we respond to each point in turn:

a) Why they were chosen. To uncover structure in complex, temporally evolving neural signals spanning hundreds (332) of ROIs across diverse cognitive and perceptual conditions, we required methods capable of capturing both dynamics and representational geometry. For this reason, we selected CEBRA [Schneider et al., 2023] and TAVRNN [Khajehnejad et al., 2024], which are specifically designed to reveal and model structure in high-dimensional neural time series. CEBRA captures temporal structure, while TAVRNN highlights ROI- and condition-specific dynamics—together providing a fuller account of psychedelic effects.

CEBRA is well-suited for fMRI data, summarising the activity of all brain regions (ROIs) at each time point into a compact representation of the overall brain state by embedding these points in a lower-dimensional space. Each point in this space corresponds to a single fMRI volume—that is, a whole-brain snapshot across ROIs. The embeddings themselves are learned directly from the fMRI BOLD signals. Training is guided by a contrastive objective, which also uses temporal adjacency as a simple supervisory signal: volumes that are high in similarity are plotted near one another in the embedding space, while those dissimilar are pushed away. Plotting successive points yields a trajectory that makes shifts between rest, meditation, music, and movie viewing easier to visualise and quantify.

TAVRNN, on the other hand, is a complementary approach that keeps the activity of each individual ROI separate, tracking how their patterns of interaction (i.e. functional connectivity) change over time. This captures detailed, context-dependent communication between regions that is undetectable to static analyses.

These models are valuable because they capture the dynamics and reveal latent (i.e., hidden) structure in the brain's response to psilocybin within a lower-dimensional, more interpretable space—something traditional analyses typically cannot achieve. We have now clarified this in the Methods section of the main manuscript as well:

"CEBRA is well-suited for fMRI data, as it summarises the activity of all brain regions (ROIs) at each moment into a compact representation of the overall brain state and embeds these points in a lower-dimensional space. Each point in this space corresponds to a single fMRI volume—that is, a whole-brain snapshot across ROIs. The embeddings themselves are learned directly from the fMRI BOLD signals. Training is guided by a contrastive objective: volumes that are similar are encouraged to map closer together in the embedding space, while more dissimilar volumes are pushed farther apart. Plotting successive points yields a trajectory that makes shifts between rest, meditation, music, and movie viewing easier to see and quantify."

"TAVRNN allows us to track these ROI-specific dynamics and their changing patterns of interaction (i.e. functional connectivity), revealing finer details. This captures detailed, context-dependent communication between regions that is undetectable to static analyses."

b) How they outperform simpler approaches.

CEBRA, proposed by Schneider et al. [2023], is the most recent deep-learning based innovation and now considered state-of-the-art among widely used dimension reduction methods like PCA [Abdi and Williams, 2010], t-SNE [Maaten and Hinton, 2008], and Isomap [Tenenbaum et al., 2000]. It explicitly preserves temporal continuity and smoothness in the embedding space, unlike traditional dimensionality reduction methods which treat each time point as an independent snapshot and are permutation-invariant to temporal order. By learning a representation that tracks the natural progression of brain activity over time, CEBRA produces trajectories where nearby points correspond to temporally adjacent states. This makes it possible to analyse and visualise how the brain moves

fluidly between states, rather than appearing as isolated clusters, thereby uncovering dynamic patterns that simpler methods cannot capture. Please refer to [Schneider et al., 2023] where its superior performance is compared with established methods in detail.

As requested, we assessed the advantage of CEBRA compared with three widely used dimensionality reduction methods: PCA, t-SNE, and Isomap. To do so, we applied these methods on the same psilocybin fMRI data for participants with high MEQ scores. All methods produced some separation of time-point embeddings between the four conditions (rest, meditation, music, movie) (as seen in Fig. R1), confirming our hypothesis that brain states under different conditions occupy distinct regions of the latent space.

• PCA showed the weakest separation due to its linear nature, capturing global variance without fine-grained contextual distinctions.
• t-SNE and Isomap achieved clearer separation but treated each time point as an independent observation, ignoring temporal relationships and producing fragmented embeddings.
• CEBRA, in contrast, preserved temporal continuity and smoothness in the embedding space, organising nearby points according to their true temporal adjacency. This not only sharpened the separation between conditions but also revealed the transitions between them, producing trajectories that reflect the brain's progression through states. This gives us a view of how these states evolve into organised patterns of brain activity, making it uniquely suited to studying continuous experiential processes like those induced by psychedelics.

Given that each of these methods—to varying extents—recovers similar embedding structures as a function of strong subjective effects, this provides further confirmation that our findings are not the outcome of a single chosen method (CEBRA) over others (PCA, t-SNE, Isomap), but rather generalise across popular dimension reduction methods.

In comparison, TAVRNN operates directly on the full set of ROIs and learns how their functional connectivity patterns evolve over time, unlike simpler low-dimensional representation techniques that reduce the whole brain to a single global trajectory. This allows it to:

• Track dynamic changes in connectivity across consecutive time points, rather than treating each time point as an isolated snapshot.

• Preserve the individuality of each ROI in the learned representation, enabling the model to follow how specific brain regions shift within the network over time.

This combination of temporal tracking and ROI-level resolution allows TAVRNN to capture richer, more fine-grained patterns of brain reorganisation compared to simpler, static embedding methods.

Together, CEBRA uncovers structure in time and TAVRNN reveals differences between individual ROI activity patterns and condition-specific dynamics—offering a complementary, more complete account of psychedelic effects across contexts.

c) What concrete biological inferences should be drawn from their latent spaces.

These machine learning methods do not infer anatomical structure, synaptic mechanisms, or physiological causality. For those types of biological inference, we rely on our conventional analyses (e.g. GFC and DCM), which are more directly tied to underlying physiology. However, the latent spaces provide a useful and complementary biological readout. They summarise how patterns of brain activity evolve over time in relation to context and the strength of the psychedelic experience.

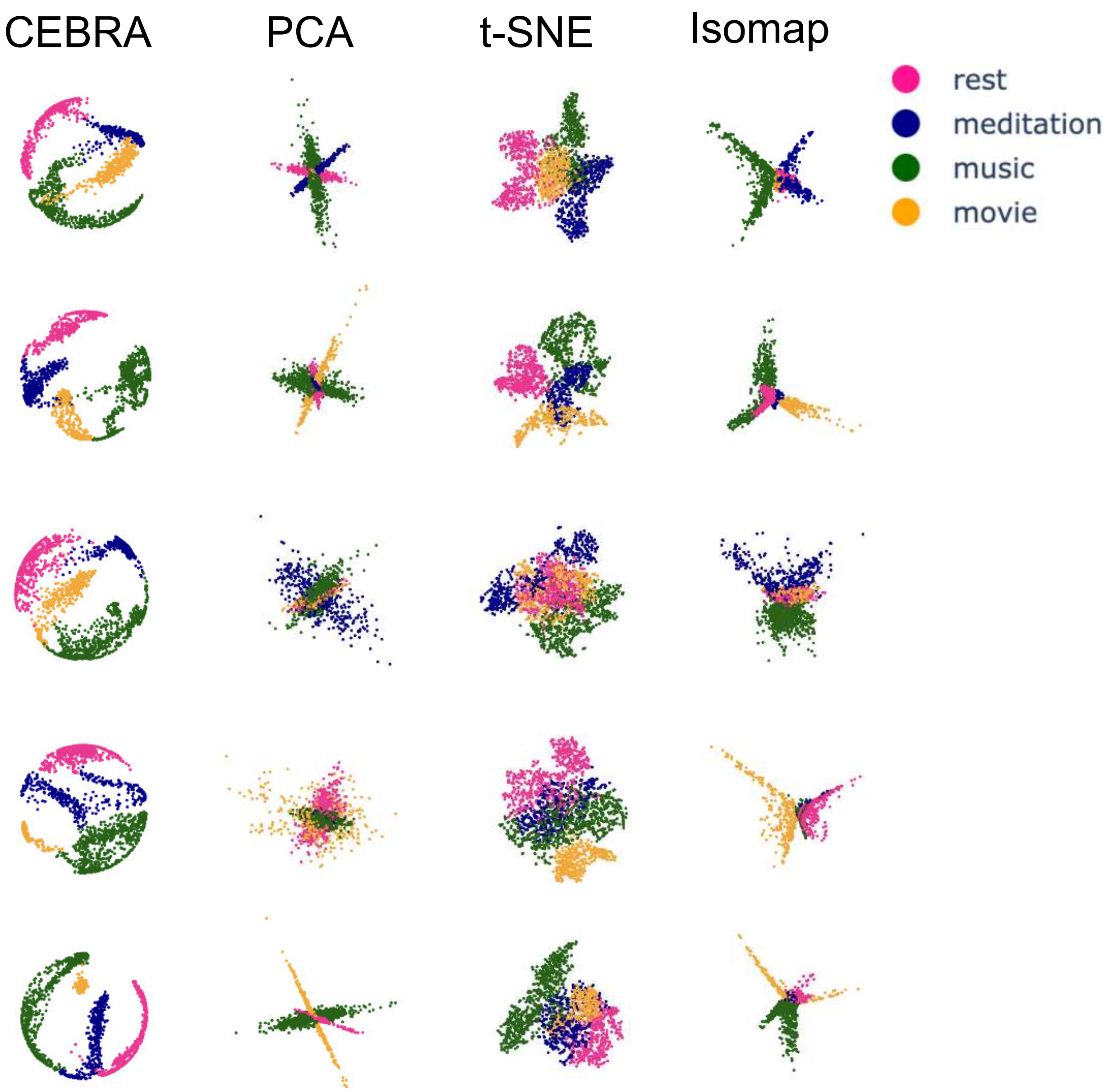

Figure R1: Comparison of low-dimensional embeddings of psilocybin fMRI data from high-MEQ participants using four dimensionality reduction methods: PCA, t-SNE, Isomap, and CEBRA. Each dot corresponds to a time point representing the activity of our 332 ROIs, colored by condition (rest, meditation, music, movie). PCA shows limited separation due to its linear nature, while t-SNE and Isomap achieve clearer separation but ignore temporal continuity, leading to fragmented embeddings. CEBRA preserves temporal adjacency, producing smooth trajectories that reveal both separation between conditions and natural transitions across brain states.

CEBRA embeddings capture temporally structured, condition-aligned patterns of whole-brain activity that are specific to individuals reporting strong self- and boundary-dissolving effects (high MEQ scores). This indicates that, under stronger psychedelic experience, distributed cortical activity is translatable by CEBRA as low-dimensional, context-sensitive temporal patterns. These embeddings provide a principled way to detect when multiregional brain activity becomes more coherent across time and context. The biological inference we draw is organisational: psilocybin is associated with a reconfiguration of brain-wide functional dynamics in a temporally structured manner, and that this organisation—rather than static amplitude—is what carries behaviourally relevant information linked to subjective effects.

TAVRNN models graph topology evolution over time and provides a biologically meaningful systems-level readout of network reorganisation. We observed increased within-network cohesion and enhanced cross-network integration in individuals reporting self–boundary dissolution (high MEQ scores). This pattern is consistent with a more flexible and integrated brain state under psilocybin, and extends prior systems-level characterisations linked to 5-HT_{2A} receptor activation [Daws et al., 2022, Singleton et al., 2022].

Together, the methods provide reproducible, interpretable low-dimensional representations of psychedelic-induced network dynamics that distinguish context and scale with self-boundary dissolution (e.g., MEQ), indicating that behaviourally relevant structure is preserved in the temporal patterning of whole-brain activity. The biological inference is therefore about the organisation of distributed network dynamics under psilocybin linked to subjective experience.

We have added a dedicated passage to the Supplementary Information, appended below, which articulates what the embeddings we present do and do not capture biologically.

Supplementary additions:

Biological interpretability of the latent spaces derived from our machine learning analyses:
"Our machine learning analyses, while agnostic to underlying neurochemistry, allow biologically grounded interpretations of functional brain reorganisation under psilocybin. CEBRA embeddings capture temporally structured, condition-sensitive trajectories of multiregional activity within individuals, revealing neural states that scale with the quality of subjective experience. TAVRNN complements this by modelling time-evolving changes in brain network topology, capturing both increased within-network cohesion and enhanced cross-network integration across conditions. These low-dimensional embeddings do not measure anatomy or causal directionality. Instead, they reflect how cortical activity reorganises over time in response to psilocybin-dependent subjective effects and context, and reveal behaviourally relevant structure in the temporal organisation of whole-brain activity. The biological inference is therefore about the organisation of distributed network dynamics under psilocybin linked to subjective experience."

d) More intuitive schematics and a step-wise rationale (input, loss, validation, interpretation) may help make this accessible for a general neuroscience audience.

In response to this suggestion, we have now included new supplementary schematics (Figs. R2 and R3 in this document) outlining the full process in a step-by-step fashion. These changes make the methods more transparent and accessible to a broad neuroscience audience.

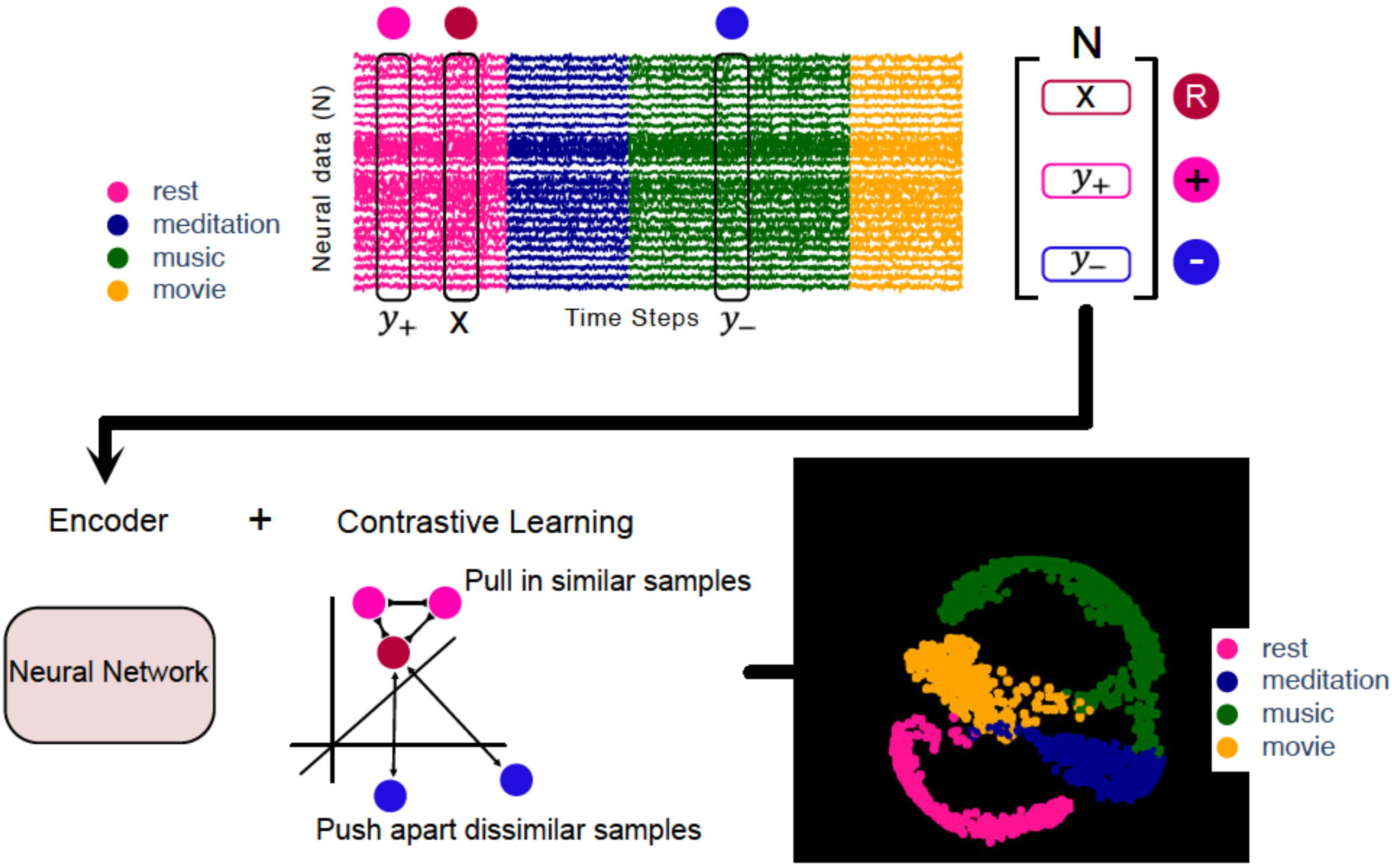

Figure R2: Overview of contrastive learning workflow of CEBRA for neural data analysis. fMRI time series from different experimental conditions (e.g., rest, meditation, music, movie) are paired with corresponding time segments. Here, X denotes the anchor time series window used as the reference time (R) to define the positive (y^+) and negative (y^-) pairs. Positive pairs (y^+) represent similar or temporally aligned samples, while negative pairs (y^-) represent dissimilar samples. An encoder processes the neural data to produce feature representations, which are optimised using contrastive learning to *pull in similar samples* and *push apart dissimilar samples*. The trained model generates a low-dimensional embedding where distinct conditions form separable clusters.

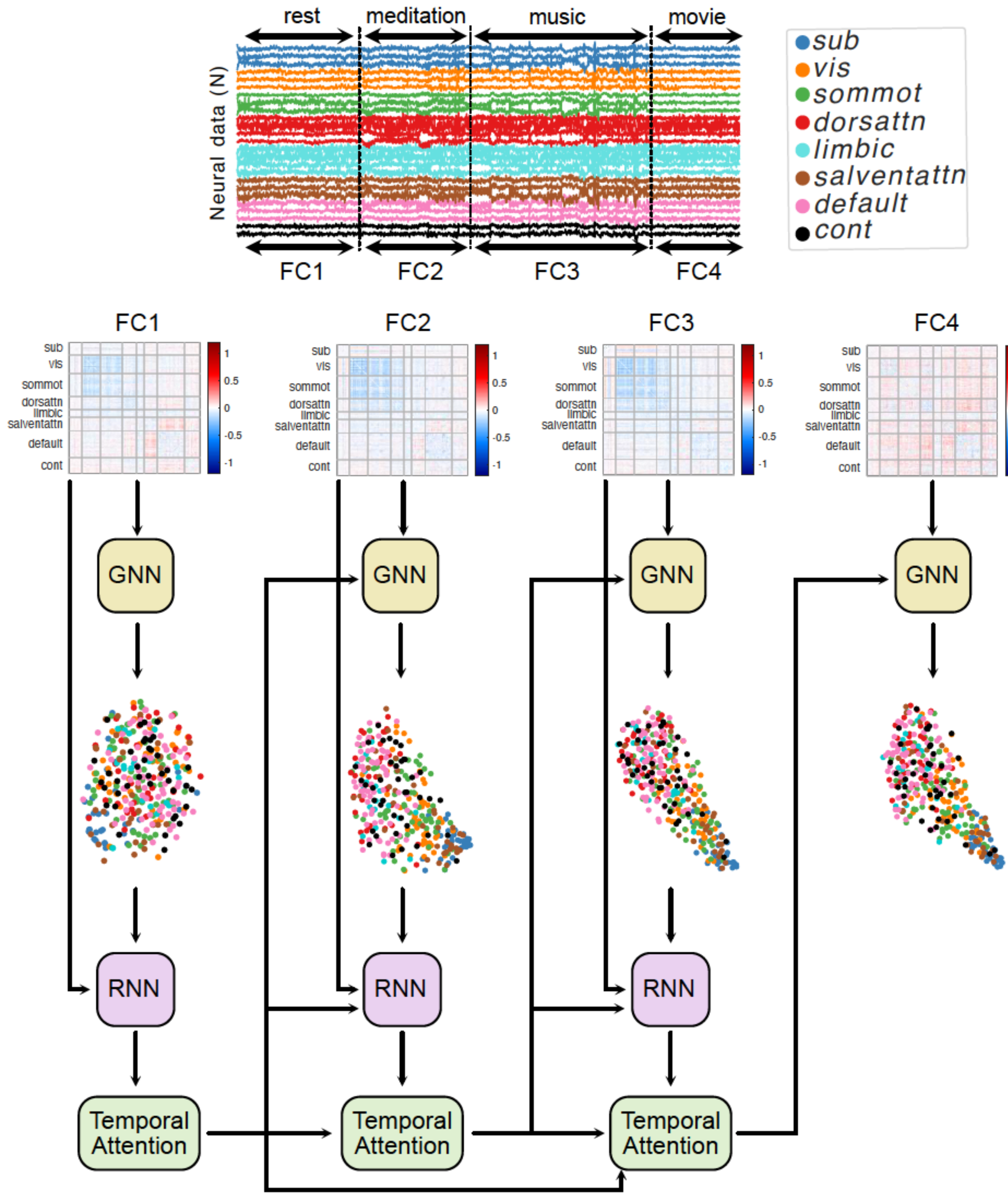

Figure R3: Schematic of the Temporal Attention-augmented Variational Recurrent Neural Network (TAVRNN) framework. Neural activity from four consecutive tasks (rest, meditation, music, movie) is converted into functional connectivity (FC) matrices, each capturing interactions between brain regions during that task segment. Each FC is processed by a Graph Neural Network (GNN) to model spatial relationships between regions, producing a low-dimensional embedding in which each point represents a single ROI (one of 332 ROIs analysed) from the original recording. ROIs with similar interaction profiles lie closer in this space. These embeddings are then passed through a Recurrent Neural Network (RNN) followed by a temporal attention layer that learns a weight for each moment in the sequence and forms a weighted summary of the RNN states. In effect, the model 'focuses' on the most informative time points (e.g., transitions or salient events) when constructing the spatiotemporal representation for each FC, rather than relying solely on the final time step. Geometry in the embedding space reflects similarity or separation of network organisation across tasks

1.2. *CEBRA classification accuracy was correlated with subjective ratings. However I am having trouble understanding how to interpret CEBRA classification accuracy.*

Authors' Response:

In our study, CEBRA classification accuracy reflects how well the learned embedding space differentiates between experimental conditions (e.g., rest, meditation, music, movie) based on time-resolved patterns of brain activity. After CEBRA learns these embeddings, a linear classifier (SVM) is trained within each subject's embedding to decode condition labels from individual time points. In this setup, each time point in the latent space carries its condition label (rest, meditation, music, or movie), so higher SVM accuracy reflects greater separability of condition-specific trajectories in that subject's embedding. A higher classification accuracy indicates that the brain activity during each condition forms a distinct, internally coherent trajectory in latent space, meaning the neural dynamics are structured and condition-specific.

Crucially, we found that this accuracy scales with the intensity of self- and boundary-dissolving subjective effects (e.g., MEQ scores and specific 11D ASC dimensions). This suggests that stronger and more meaningful psychedelic experiences lead to more distinct and coherent neural trajectories, where each context (music, meditation, etc.) evokes a reliably different brain state. In contrast, when the experience was minimal or the subjective effect not well-aligned with the task context (e.g., in participants with late-onset effects), the neural trajectories were less separable, which is reflected in lower classification accuracy.

Therefore, CEBRA classification accuracy is interpretable as a marker of differentiation between brain states across contexts, and shows how this organisation is enhanced when the psychedelic experience is high on positively felt and boundary dissolving dimensions of the MEQ and 11D ASC.

We have now clarified this in the main Results text immediately after detailing how different types of subjective effects relate to the model's performance :

"A higher classification accuracy highlights that psilocybin influences the context-specific coherence of neurodynamics in a manner that scales with the intensity of subjective experience."

1.3. *I take issue with claiming that your metric of CEBRA classification accuracy predicts next-day Mindset change (r=0.4). You should that components of the 11D-ASC predict next day mindset with r=0.6. And that CEBRA classification accuracy predicts components of the 11D-ASC. So I would guess that any relationship between CEBRA classification and next day mindset is simply mediated by both being correlated with 11D-ASC scores.*

Authors' Response:

We appreciate the opportunity to clarify our reasoning. We understand the suggestion that the association between CEBRA classification accuracy and next-day mindset may reflect shared variance with administration-day subjective experience (as assessed using the 11D-ASC). We do not interpret this correlation as evidence of an independent causal pathway. Rather, we consider it to demonstrate that the brain activity embeddings learned by CEBRA account for variation in the after effects of the psychedelic state. We include this analysis to show that the embeddings encode experiential information that relates meaningfully to psychological change. Importantly, the embeddings are derived

directly from brain activity, without reliance on self-report. This suggests brain-derived embeddings can track key experiential components of the psychedelic state that relate to subsequent outcomes.

In response to the reviewer's comment, we have clarified this interpretation in the manuscript's Discussion:

"Although this association is likely mediated by subjective experience, it supports the view that brain embeddings capture meaningful experiential features of the psychedelic state that relate to subsequent psychological change. This suggests that brain-derived embeddings may offer a complementary approach for tracking subject-specific correlates of change where self-report is difficult."

***1.4**. I can't figure out what is being shown in Fig. 3d. The plots do not appear to have axis labels.*

Authors' Response:

Fig. 3d shows two-dimensional embeddings produced by TAVRNN for each condition across participants. While the axes are not labelled with physical quantities—since they represent abstract dimensions learned by the model where only relative distances and neighbourhoods carry meaning—the colour coding clearly distinguishes different brain networks, and each panel is labelled by condition and MEQ grouping. Each point in these plots represents one brain region (ROI), and the model places points closer together when those regions behave similarly over time. This way, the model compresses hundreds of time-varying signals into just two coordinates for each brain region, allowing us to compare overall brain dynamics across different conditions and participant groups. We have added "a.u." (arbitrary units) to the axis labels, with the notation explained in the main-text figure caption.

When we say "abstract dimensions," we mean that the x and y axes in these plots don't have a direct anatomical meaning (like mm or voxels), but instead represent data-driven dimensions that capture how similar or different the activity of each brain region is in the context of the task. This technique helps us visualise large-scale differences in brain dynamics in a way that is compact, intuitive, and easy to compare between people and conditions, especially useful for comparing groups with high vs. low subjective response to psilocybin.

To improve clarity, we have now:

• Added the following note in the caption explaining that the axes represent abstract latent dimensions:

"The axes represent abstract latent dimensions learned by the model and do not correspond to physical spatial coordinates."

• Reaffirmed that each dot represents one ROI, and colours correspond to functional brain networks.

• Highlighted in the main Results text how to interpret these embeddings as spatial layouts of neural activity across conditions:

"These 2D maps represent abstract latent dimensions learned from brain activity patterns, not physical brain coordinates, with each point corresponding to one ROI placed closer to others when their activity evolves similarly over time."

***1.5**. Order effects, lack of counterbalancing of conditions. It appears that the four conditions were always in the same order - eyes-closed rest, guided meditation, music listening, then movie. Thus, condition differences are confounded by time/PK/fatigue differences.*

Authors' Response:

Thank you for raising this important point.

The stimulus sequence (rest $\rightarrow$ meditation $\rightarrow$ music $\rightarrow$ movie) was by design to support the psychological safety and experiential coherence for psychedelic naïve participants in the MRI environment, progressing from low to high intensity context. Our objective was to enhance ecological validity of the recorded brain states by allowing the participant's experience to unfold with minimal disruption from changes in experimental stimuli.

For conventional (non-ML) analyses, our primary inferences concern drug effects within each condition (psilocybin vs no-drug for rest/meditation/music/movie), where order is identical across sessions; these contrasts are therefore not confounded by within-session position. For between-condition comparisons within a session, we assess influence by time-on-scanner, PK, and fatigue, and for completeness, we also assess order impact on our eyes-open vs eyes-closed findings and on the eyes-closed analyses.

For our ML findings, we evaluate sequence effects within this scaffold and demonstrate that no structured embedding emerges at baseline or with low MEQ, indicating that the fixed order alone do not produce the observed phenomenon. Additionally, the structure of embeddings is observed during rest—before order was introduced—suggesting this finding is not a product of order. We also conducted additional analyses to examine monotonic trends of CEBRA embeddings with scan order, which could indicate a generic property of fatigue or the task battery, and found none. Moreover, we provide evidence that inter-individual variability in post-dose pharmacokinetics and pharmacodynamics (PK–PD) undermines a PK-based account.

ML embeddings depend on subjective effects' intensity, not sequence.
We trained CEBRA per participant and per session (baseline and psilocybin) with a contrastive, self-supervised objective that does not use condition labels; labels (rest, meditation, music, movie) were applied only post hoc for visualisation and evaluation. Clear, condition-separable structure emerges only in psilocybin sessions with high MEQ (strong subjective effects); baseline and low-MEQ sessions show weak or no clustering. Crucially, on the drug day this structured geometry is already evident during rest—the first scan—before cumulative sequence effects could accrue, arguing against an order-driven explanation (Fig. R4a–b, pink clusters; Fig. R2 for closeup of MEQ 100 score). TAVRNN reproduces the same macro-pattern (main-text Fig. 3d): when subjective effects are strong, trajectories are condition-specific; when effects are weak, trajectories are diffuse and overlapping.

Quantification using silhouette scores.
To quantify cluster quality and probe for any order-consistent pattern, we additionally computed the *silhouette* scores per participant and per condition (Fig. R4c–d), averaging across 10 resampling iterations. The silhouette score is defined as

$$s = \frac{b - a}{\max(a,\, b)}, \tag{1}$$

where a is the mean within-cluster dissimilarity and b is the mean dissimilarity to the nearest neighbouring cluster. Larger s indicates tighter, better-separated clusters.

At baseline, low- vs high-MEQ groups do not differ across conditions (all $p > 0.19$, Welch's two-sample t-test, Fig. R4c), indicating weak, non-differentiated geometry. Under psilocybin, silhouette scores are significantly higher for high-MEQ across *all four conditions* (Rest $\Delta s = 0.512$, $p = 0.0002$; Meditation $\Delta s = 0.710$, $p = 0.0023$; Music $\Delta s = 0.515$, $p < 0.0001$; Movie $\Delta s = 0.631$,

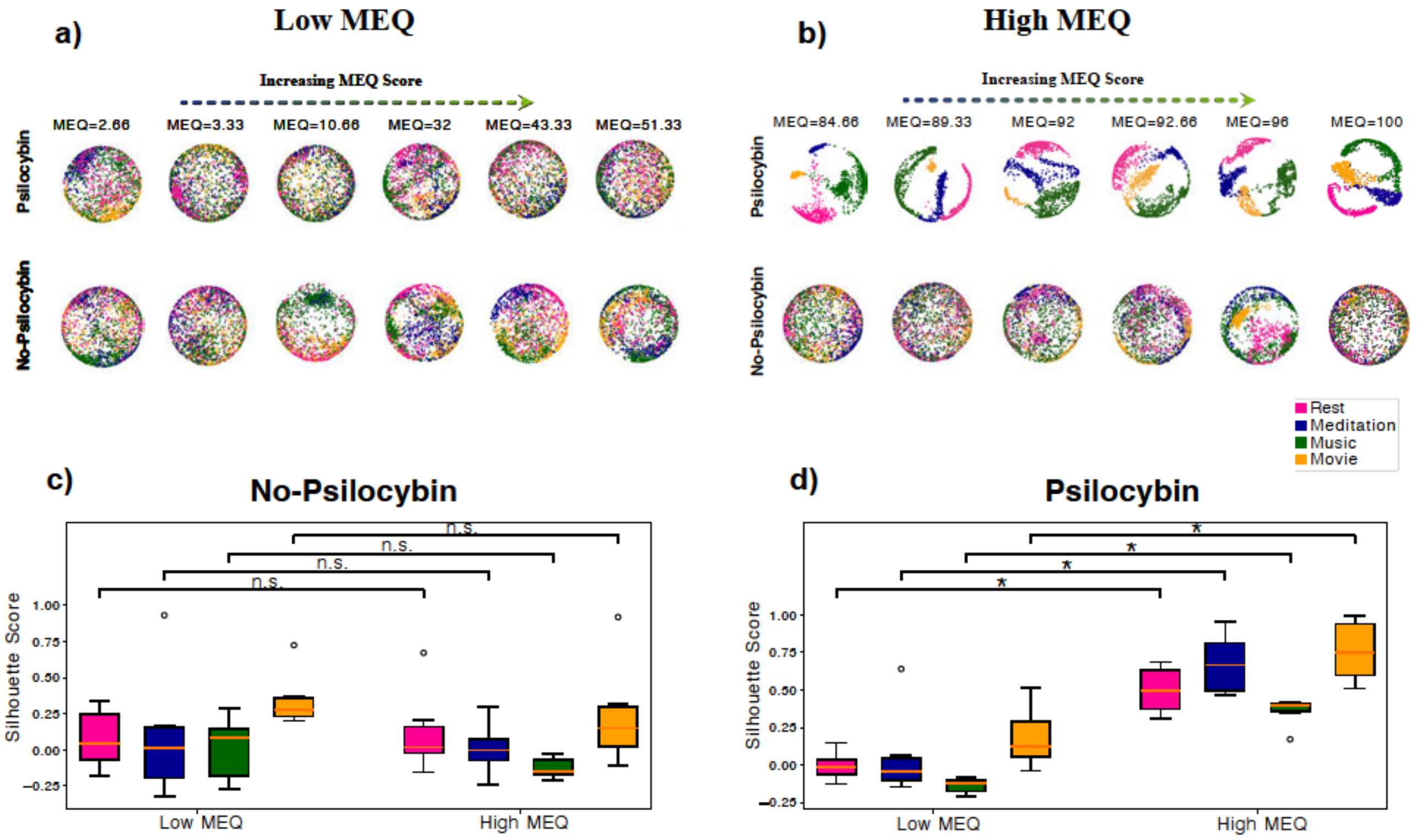

Figure R4: **Comparison of CEBRA embeddings and silhouette scores for subjects with low vs. high MEQ scores.** **(a)** Embeddings of six subjects with *low MEQ sorted by low classification accuracy* under Psilocybin and No-Psilocybin conditions. **(b)** Embeddings of six subjects with *high MEQ sorted by high classification accuracy* under Psilocybin and No-Psilocybin conditions. **(c)** Box plots of silhouette scores during the No-Psilocybin condition, showing no significant (n.s.) differences between low and high MEQ groups. **(d)** Box plots of silhouette scores during the Psilocybin condition, showing significantly higher clustering quality in high MEQ subjects compared to low MEQ subjects. Together, these results indicate that the separation of neural state representations captured by CEBRA is strongly modulated by the intensity of the subjective experience (MEQ), while baseline (No-Psilocybin) states show comparable structure across groups. Psilocybin leads to markedly stronger and more distinct embeddings in high MEQ participants, consistent with enhanced experiential differentiation.

$p = 0.0007$), showing that clustering tracks subjective intensity, not mere drug administration or scan order (Fig. R4c-d).

We have now added the new analyses demonstrated in Fig. R4 as Extended Data Fig. 7 to the revised manuscript.

No monotonic order signature.
If fatigue or generic time-on-scanner effects explained the structure, silhouette values should change monotonically with scan index (1→4). They do not. The typical profile is non-monotonic (Movie ≈ Meditation > Rest > Music; i.e., 4-2-1-3). A mixed-effects model with silhouette $\sim$ *scan_index* + (1 | participant) shows no linear trend with scan order ($\beta_{\text{scan}} = 0.046$, SE $= 0.041$, $p = 0.258$).

Cross-modal replication with order reversal.
The eyes-open (EO) vs eyes-closed (EC) convergence replicates across fMRI and EEG even though the movie order is reversed and separated by a 20–40 min setup break (room change, cap placement, impedance checks): in fMRI, EO movie occurs last; in EEG, EO movie occurs first. We analyse

EO–EC as psilocybin–baseline contrasts, which subtract fixed-order effects shared across sessions. Replication under order reversal and temporal separation makes a simple sequence/fatigue account unlikely and supports a drug- and context-dependent convergence.

For within-session, cross-condition, we provide the following analysis and findings to further rule out the order effects.

Under fMRI, psilocybin decreases the separation in global functional connectivity (GFC) between EO and EC (main-text Fig. 2d and Extended Data Fig. 5). The largest effect was observed in the visual network (-85%, with a robust Cohen's d effect size of -3.23), but the reduction was larger than -66% and highly statistically significant in all Schaefer networks (Mann-Whitney U-test right-tailed p-values $p < 10^{-307}$). Additionally, head-motion indices show no monotonic rise across the session (Fig. R8), weakening and ruling out a fatigue/sequence account.

Under EEG, across the three EC conditions (rest, meditation, music), the group-averaged spectra show similar broadband reductions in theta/alpha/beta power under psilocybin. By contrast, the EO movie spectrum is relatively preserved (i.e., minimal change vs baseline) in the group average (main-text Fig. 6b). Consequently, the EO–EC power difference narrows under psilocybin. This attenuation is also visible in the spatial power-difference maps (psilocybin minus baseline; main-text Fig. 6a), where EO exhibits a reduction that is clearly smaller than the EC reductions. The alpha power difference between eyes-closed and eyes-open conditions decreased by 48% after psilocybin administration (robust Cohen's $d = -0.79$, Mann-Whitney U-test right-tailed p-value $< 10^{-19}$).

Moreover, our eyes-closed EEG findings are consistent with previous literature: Timmermann et al., 2023's simultaneous EEG–fMRI acquisition under the psychedelic DMT found directionally consistent alpha suppression (commonly reported under peak effects [Muthukumaraswamy et al., 2013]) co-occurring with increased fMRI GFC, despite our EEG session beginning approximately 150 minutes post-dose [Timmermann et al., 2023]. This substantiates the authenticity of the change we see from EO-EC, and relatedly, demonstrates the effects we measure at 150 minutes are valid peak effects (noted in Supplementary Information). Time-to-peak (post-dose PK-PD) varies across individuals under psilocybin (subjective peaks 60–90 min [Hasler et al., 2004]; psilocin $T_{\max}$ $\sim$2 h, range $\sim$1–3.5 h) [Brown et al., 2017, Otto et al., 2025], meaning a fixed scan order samples different phases of the PK response across participants, such that PK exposure is not systematically aligned with scan order.

The above noted consistency is now reported in the main text:

"Comparable decreases in alpha power and increases in signal diversity have also been observed under intravenous administration of the serotonergic psychedelic N,N-dimethyltryptamine (DMT) Timmermann et al. [2023] , suggesting these may be conserved markers of serotonergic psychedelic action, despite differences in compound, route of administration, timing, and EEG methodology."

Motion and block-structure controls are inconsistent with order artefacts.
Head-motion indices (e.g., mean FD, DVARS) show no monotonic rise across scans; these covariates are included in group models. Each block is 6–11 min, far longer than canonical haemodynamic carry-over windows (peak ~5 s; return to baseline ~20 s) [Aguirre, 2007, Boynton et al., 1996, Glover, 1999] and fast electrophysiological state changes (sub-second to seconds) [Michel and Koenig, 2018], making immediate cross-block carry-over an implausible driver. Additionally, network modularity follows a non-monotonic context pattern (rest $>$ music $>$ movie $>$ meditation), again inconsistent with progressive order effects.

However, for caution we report the fixed sequence as a design feature in Methods and acknowledge its potential influence transparently in the Supplementary. With respect to within eyes-closed scans,

we make only one descriptive comparison (music ≈ meditation spectra) and use this description to direct readers to Methods for timing and order details.

Methods (Study design):

"In fMRI, conditions began 80 minutes post-dose and followed a fixed order (rest → meditation → music → movie). The sequence progressed from low-stimulus to high-stimulus contexts to prioritise safety and experiential coherence for psychedelic-naïve participants. The EEG session started after a room transfer and 20–40 minutes of setup (cap placement and impedance checks), with the movie presented first, followed by the three eyes-closed conditions (movie → rest → meditation → music)."

We note the following at the end of the study design section:

"Discussion addressing expectancy, task order and EEG timing is detailed in Supplementary Information."

We have also made an adjustment for precision and transparency about order, also indicating its discussion in Supplementary:

"Meditation and music show visually similar group-averaged power spectra at baseline and under psilocybin(Fig. 6b); due to our fixed order design, we report these findings descriptively (see Methods)."

Supplementary Information (Order considerations):
"The fixed within-modality sequence was chosen for safety and experiential coherence. We note that: (i) the movie condition was reversed across modalities (last in fMRI, first in EEG) and separated by a 20–40 minute setup break; (ii) head-motion indices showed no monotonic rise across sessions (Extended Data Fig. 2); (iii) inter-individual post-dose pharmacokinetic-pharmacodynamic timing variability [Hasler et al., 2004, Brown et al., 2017, Otto et al., 2025] means the scan order does not correspond to a common exposure phase across participants; and (iv) each scan lasted 6–11 minutes, limiting carry-over effects. Together with the absence of sequence-aligned signatures in the ML embeddings and (Extended Data Fig. 7) cross-modal replication of reduced differentiation between eyes-open and eyes-closed (main-text Figs. 2 and 6), these factors mitigate the risk that fixed order explains the main results (i.e., the ML findings and the eyes-open vs eyes-closed convergence). Residual order influence cannot be excluded for fine-grained comparisons among the eyes-closed conditions analysed with conventional metrics. Because these scans were presented in a specific order—progressing from low- to high-stimulus (rest → meditation → music), subtle sequence effects (potentially state-dependent under psilocybin, and longer-timescale context drift) could contribute to their relative differences. Accordingly, eyes-closed comparisons are reported descriptively."

1.6*. When was the fMRI started? I can not find it stated anywhere how long after drug ingestion the MRI began.*

Authors' Response:

The fMRI session began approximately 80 minutes after oral administration of psilocybin. We have now added this information to the main-text Fig. 1 caption and clarified it in the Methods section, and added it to the abstract.

Abstract:

”Sixty-two adults were scanned with functional MRI and EEG during rest and naturalistic stimuli (meditation, music, and visual), before and after ingesting 19 mg of psilocybin (functional MRI at 80 min post-dose; EEG at 150 min post-dose).”

Main-text Fig. 1:

”An open label ’baseline’ (no-psilocybin) imaging session was conducted approximately one week before imaging under administration of 19 mg psilocybin. MRI and EEG imaging began ≈80 and ≈150 minutes post-dose, respectively.”

Methods:

“In fMRI, conditions began 80 minutes post-dose and followed a fixed order (rest → meditation → music → movie). The sequence progressed from low-stimulus to high-stimulus contexts to prioritise safety and experiential coherence for psychedelic-naïve participants. The EEG session started after a room transfer and 20–40 minutes of setup (cap placement and impedance checks), with the movie presented first, followed by the three eyes-closed conditions (movie → rest → meditation → music).”

1.7. *Dynamic-causal-model - It is not clear to me how this fits with the other observations made in the paper. There is already a lot of content, perhaps this would be better of in a separate manuscript to allow space for other interesting results in this manuscript to shine (for example I would love to see more interpretation of the collapse of GFC differences between rest vs movie watching).*

Authors’ Response:

We appreciate the concern about fit and length. In revision, we retained a lean DCM result because it provides directional, circuit-level evidence that complements our undirected fMRI/EEG findings while keeping focus on the eyes-open eyes-closed changes. Specifically, we (i) pruned the DCM text to reduce paragraph length, and outline posterior probability ≥ 0.99 thresholds with a simple magnitude summary, and (ii) moved extended interpretation to the Supplementary. We now demonstrate that DCM differentiates eyes-open from eyes-closed contexts via directed aHip–DMN changes. Separately, we expanded the eyes-open eyes-closed convergence interpretation within the Discussion as requested, without over-speculating, and were able to bring the discussion of the DCM findings in line with the theme of eyes-open vs eyes-closed. We hope this addresses fit and provides the requested interpretations, which we believe helps clarify these findings and their impact, while preserving a concise mechanistic complement to our other analytical approaches.

Revised DCM text (removed text is now in Supplementary)

“Dynamic causal modelling (DCM) [Friston et al., 2003, 2014, Razi and Friston, 2016] tested a model of directed coupling between the anterior hippocampus (aHip) and core DMN nodes across rest, meditation, music, and eyes-open movie viewing, comparing baseline (no-psilocybin) and psilocybin conditions (psilocybin minus baseline) (Fig. 5). Group Parametric Empirical Bayes (PEB) contrasts revealed condition-specific changes in effective connectivity. Eyes-closed conditions showed relatively modest changes, while themovie condition exhibited the largest number and magnitude of changes. The total strength of between-region parameter changes (sum of absolute changes with posterior probability (PP) ≥ 0.99; in Hz) was: rest ≈ 0.75; meditation ≈ 0.66; music ≈ 0.73; movie ≈ 1.48 (largest). Overall, eyes-open movie viewing elicited a more complex reconfiguration of connectivity changes relative to the patterns observed in eyes-closed states, providing directional (mechanistic) evidence that complements our undirected fMRI GFC and EEG power analyses. Full details, pa-

rameter estimates and interpretation of directed connectivity changes across contexts are provided in Supplementary Information."

Added more convergence interpretation in the Discussion:

"Therefore, while the spatial organisation of connectivity (fMRI surface maps) and neural dynamics (EEG scalp topographies) remains distinct from baseline (no psilocybin), the overall distributions of connectivity strength (GFC values, (Fig. 2d,e)) and spectral power (EEG power, (Fig. 6b)) converge under psilocybin—indicating that eyes-closed states shift toward a more externally engaged profile while preserving condition-specific topology. We interpret this as increased participation of associative networks in shaping perception during eyes-closed states."

This has also allowed us to align discussion of the DCM results with eyes-open and eyes-closed findings:

"Using DCM, we demonstrated that eyes-open vs eyes-closed context differentially tunes the directed connectivity of these brain circuits during the acute effects, with the eyes-open movie showing the greatest magnitude of directed change, in line with eyes–open eyes-closed distinctions in our fMRI and EEG findings."

1.8. *There does not appear to be any attempt to report or account for differences in head motion between baseline and psilocybin condition. This is almost certainly the reason why there are large increases in SD in the temporal pole and orbital frontal cortex on psilocybin (Fig. 2c) as these are parts of the brain next to sinuses where there is classically the largest motion-correlated confounds. The methods state "7 subjects have one or more condition excluded from the analysis...most corresponded to frame-wise displacement greater than 0.5" This is a fairly loose motion threshold, particularly if there is no censoring of high motion frames prior to analysis.*

Authors' Response:

We thank the reviewer for raising this important point. Motion confounds are a well-recognised challenge in fMRI analysis, and their potential to produce spurious effects particularly in regions near air–tissue interfaces is well established, something we were cautious about in our analyses.

In light of the reviewer's concern that the SD increases observed under psilocybin, particularly in sinus-adjacent regions, might reflect residual motion, and that our original FD threshold (0.5 mm) might be insufficiently strict, we conducted a supplementary robustness analysis using a far more stringent criterion. Specifically, we re-ran the standard deviation analysis from main-text Fig. 2c using a revised FD threshold of 0.25 mm. This threshold is twice as conservative as the original, and is among the most stringent in the fMRI literature [Parkes et al., 2018]. Notably, applying this threshold resulted in approximately six times more data being excluded from the analysis, substantially reducing the risk that head movements could bias the results.

Despite this drastic increase in data exclusion, the spatial pattern of standard deviation differences (psilocybin minus baseline) remained qualitatively unchanged, as shown in Fig. R5. The characteristic increases in standard deviation in orbitofrontal and temporal pole regions were preserved, suggesting that these effects are not artefacts of unmodelled or insufficiently controlled motion. Rather, they appear to reflect genuine signal variance arising under psilocybin, which may plausibly be linked to increased dynamic range of neural activity or altered neurovascular coupling in these regions.

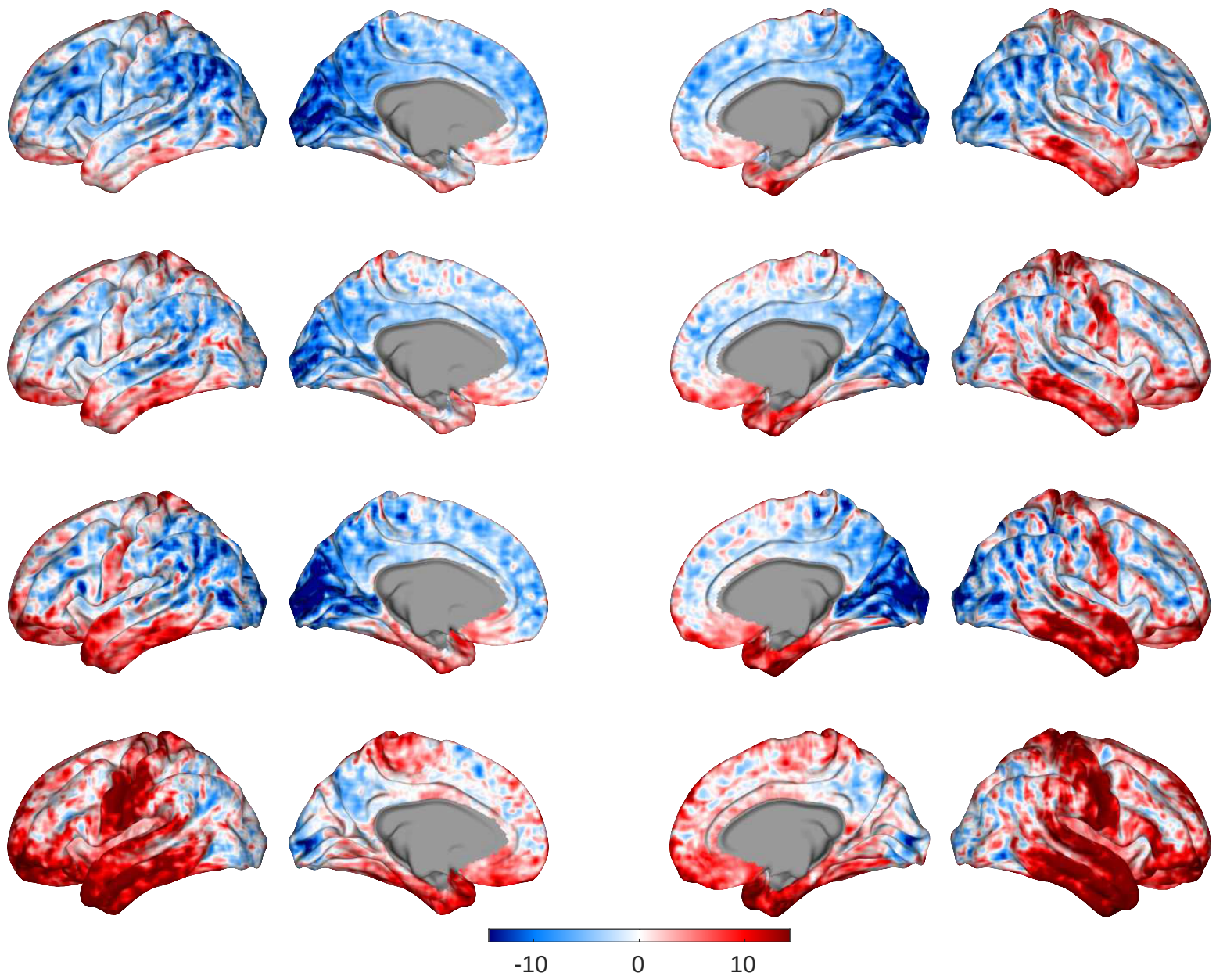

Figure R5: Repeating the standard-deviation (percentage-difference) map with (FD < 0.25, approximately 6 times more data excluded) preserved the spatial pattern shown in main-text Fig. 2c, indicating the effect is not driven by residual motion.

To further quantify and contextualise head motion across sessions, we computed and visualised the mean FD separately for each participant and session (psilocybin versus baseline). As shown in Fig. R6, there was a modest but consistent increase in mean FD during the psilocybin condition. This finding is not surprising, as psychedelic states may induce increased physiological arousal, restlessness, or involuntary facial or body movements, even in the absence of overt participant discomfort. However, the presence of higher FD does not, in itself, imply that our findings are motion-driven—particularly since the key effects remain present even under very strict FD filtering. That is, although head motion was indeed elevated under psilocybin, the fact that we obtained qualitatively identical results after censoring high-motion data (FD $<$ 0.25 mm) indicates that residual motion is not responsible for the observed effects.

Beyond exclusion and regression strategies, our use of multi-echo imaging offers further protection against motion-related artefacts. As an additional validation, we assessed the impact of our denoising pipeline on data quality by calculating temporal signal-to-noise ratio (tSNR) before and after ME-ICA cleaning. We compared single-echo data, optimally combined multi-echo data, and ME-ICA-cleaned data, and quantified tSNR improvements across the whole brain. As shown in Fig. R7, ME-ICA significantly increased tSNR, particularly in inferior frontal and temporal regions near the sinuses, which are most vulnerable to dropout and movement-related distortions. The fact that tSNR improves markedly in these regions following denoising supports the view that ME-ICA is effectively reducing non-BOLD variance in precisely the regions where artefacts are of greatest concern.

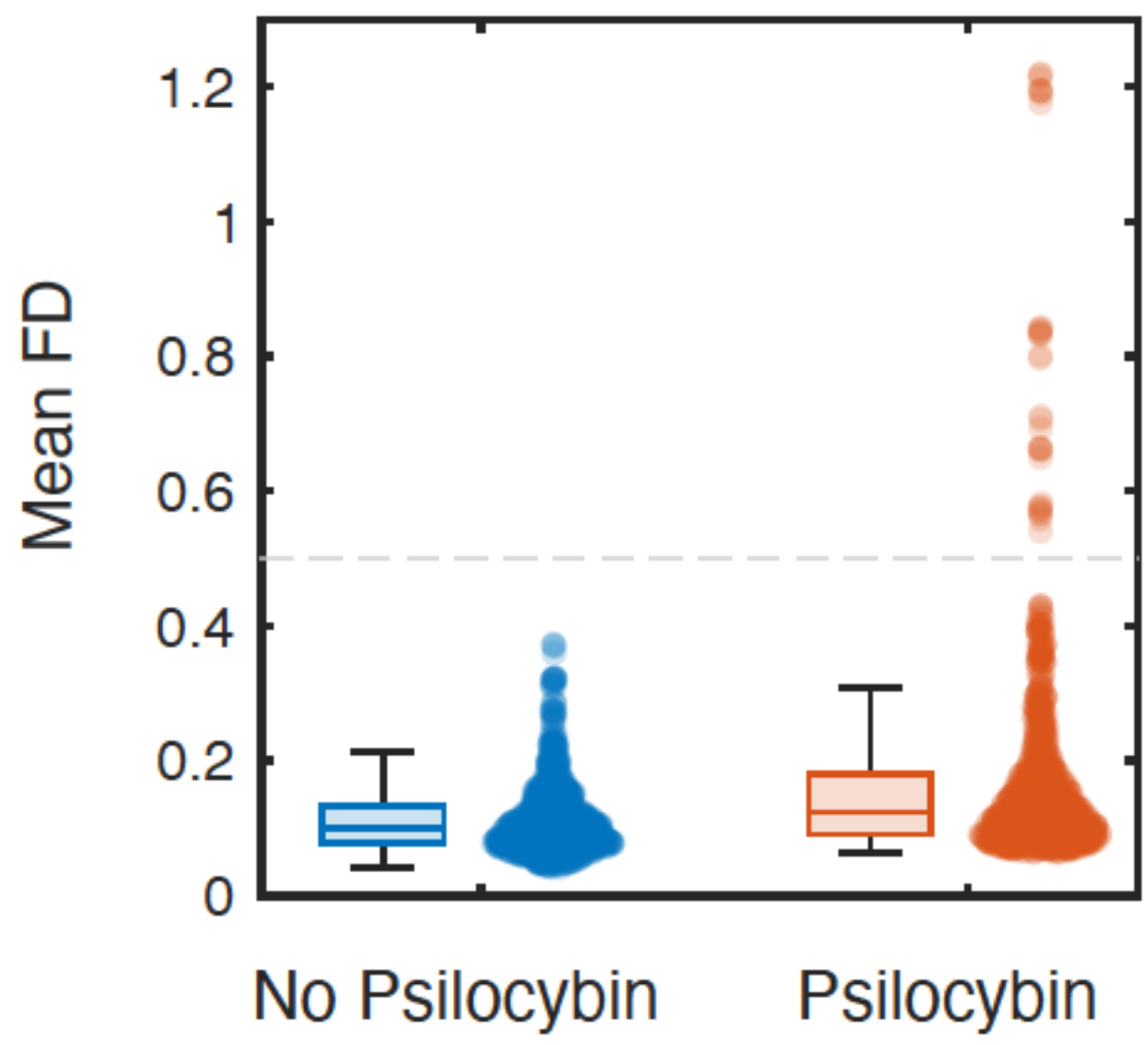

Figure R6: Mean FD (in mm) by session. Dashed line indicates the original exclusion threshold (FD = 0.5 criterion). Participants moved more during psilocybin. In Fig. R5, we show that the main effects are robust to head motion during psilocybin using the much more stringent threshold FD < 0.25.

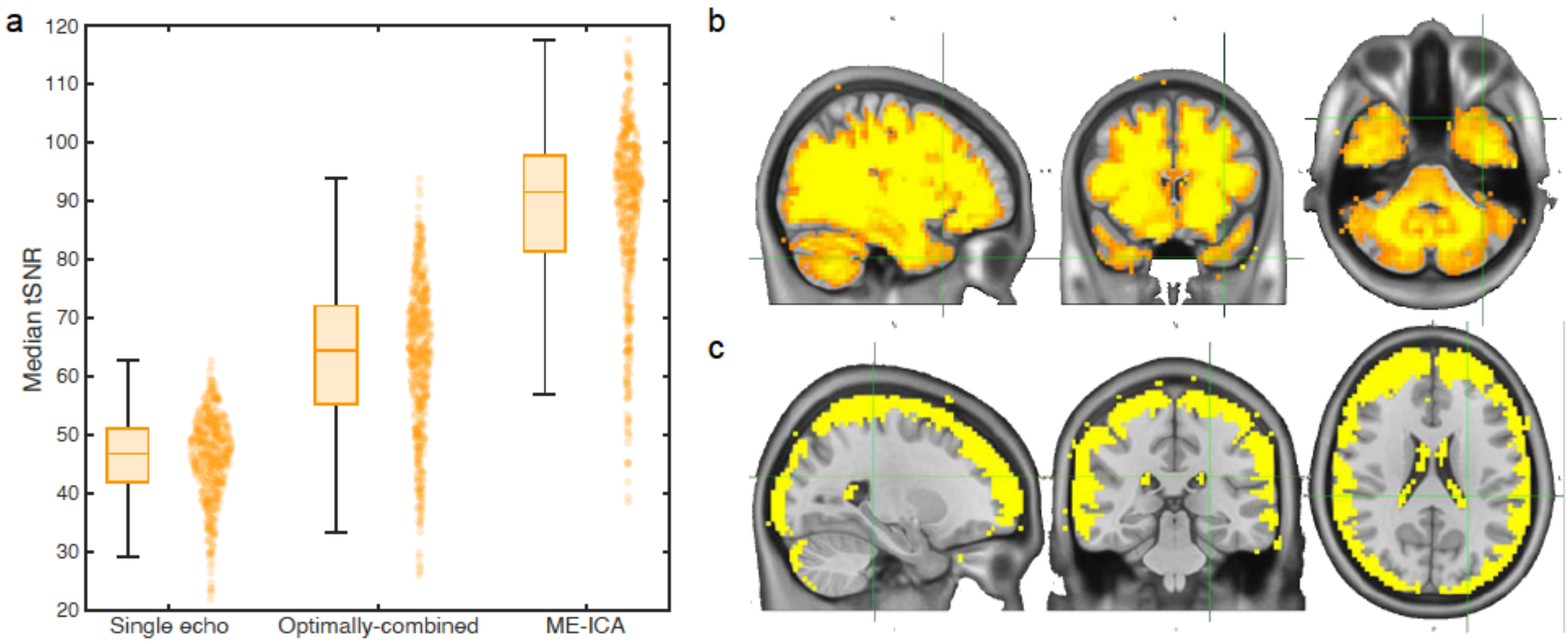

Figure R7: **a)** Comparison of temporal signal-to-noise ratio (tSNR) between single-echo data, optimally combined multi-echo data, and cleaned data via the multi-echo independent component analysis (ME-ICA). **b)** Spatial distribution of temporal signal-to-noise ratio (SNR) differences. Largest tSNR increase in the optimally combined multi-echo data compared to single-echo data. **c)** Further tSNR increase when using cleaned data via ME-ICA compared to the optimally combined data (thresholded at the top quartile to locate the voxels with the largest tSNR increase).

These quality control analyses (between-session FD and tSNR gains with ME-ICA) are fully documented in our dataset descriptor, which we cite in the manuscript for completeness [Novelli et al., 2025]. Taken together, these analyses support the conclusion that the observed spatial patterns of standard deviation under psilocybin are not attributable to motion. Rather, they reflect condition-dependent variance in BOLD signal, consistent with the broader set of findings presented in the manuscript.

1.9. *It is not clear if the authors intend to suggest a link between the ML concept of low-dimensional embeddings and the psychological construct of embeddedness. In the best case, the repeated use of the two terms is confusing.*

Authors' Response:

We appreciate the reviewer's attention to this important point. We agree that "embedding" (in the machine learning sense) and "embeddedness" (as a subjective construct) must be clearly distinguished, especially given their lexical similarity. While the terms are not synonymous, the relationship between embeddings and embeddedness is empirical: the context-organisation of brain embeddings scales with subjective effects. To keep definitions clear and avoid redundancy, we now (i) define ML embeddings once in the Results, and (ii) introduce and delineate embeddedness only at its first appearance in the Discussion. This staging keeps the technical term distinct from the experiential construct and prevents confusion. We demonstrate these changes as follows:

Revised the Results, where we first introduce Embeddings.

"In ML, an *embedding* is a learned mapping from high-dimensional data to a low-dimensional representation, expressed as vector coordinates in an embedding space that preserve latent structure. Applied to fMRI time series of brain activity, these embeddings form representations in a low-dimensional space [Cunningham and Yu, 2014, Pillai and Jirsa, 2017, Perl et al., 2025] that reveal latent organisation in the data."

Revised the Discussion, where the terms are first described together.

"While *embeddedness* refers to an experiential state, CEBRA and TAVRNN are machine learning methods that learn embedding spaces from imaging data. Although the experiential construct and the computational representations are conceptually distinct, the context-organised clustering of low-dimensional coordinate vectors within the learned embedding spaces co-varied with the intensity of subjective effects (Fig. 3b,d; Fig. Extended Data 7a,b), indicating an association with experiential *embeddedness*."

Additionally, we removed instances of Discussion text that risks confounding the definitions and does not explain the terms clearly (e.g. "These findings suggest that embeddedness, as captured by these embeddings, corresponds with psychologically adaptive outcomes.")

Please note, figure links in the quoted text relate to the main manuscript.

1.10.*Lack of a placebo-controlled or double-blind design. The open-label approach heightens expectancy and demand characteristics, which could inflate subjective scores and neural changes.*

Authors' Response:

There is an ongoing debate about how effectively a placebo control can be implemented in psychedelic trials. Below we (i) explain why blinding fails at full psychedelic doses, (ii) summarise evidence that expectancy effects are small compared with pharmacological effects in this setting, and (iii) describe objective drug–brain–behaviour couplings observed in our data and in prior work.

Blinding at macrodose is empirically fragile.
At moderate–high psychedelic doses, effective blinding is difficult to achieve in practice because distinctive subjective effects make allocation easy to infer [Gasser et al., 2014, Studerus et al., 2011, Szigeti and Heifets, 2024], and suitable active placebos are lacking, with participants often discerning allocation when active placebos are used [Bogenschutz et al., 2022, Muthukumaraswamy et al., 2021]. Even under double-blind conditions, trials report very high correct-guess rates. In a double-blind crossover directly comparing lysergic diethylamide acid (LSD), psilocybin, and placebo in healthy adults, placebo was correctly identified in ~96 percent of sessions [Holze et al., 2022]; in a RCT, only one of 20 patients mistook LSD for placebo [Szigeti and Heifets, 2024, Holze et al., 2023]; and a psilocybin RCT for alcohol use disorder reported ~94 percent correct treatment guesses [Bogenschutz et al., 2022, Szigeti et al., 2024]. As Szigeti et al. note, "if a trial is called 'placebo controlled', then it should really control for the placebo effect and not just have a 'placebo group'" [Szigeti et al., 2023]—a standard that current active placebos rarely satisfy. Taken together with pooled macrodose evidence on psychedelic phenomenology and blinding integrity [Studerus et al., 2011, Muthukumaraswamy et al., 2021, Szigeti and Heifets, 2024], these data underscore the absence of viable active placebos that reproduce the perceptual and introspective profile of classic psychedelics—effects which are impractical to achieve through demand and expectancy, motivating our within-subject baseline control.

Expectancy effects appear small in full-dose settings.
Broader discussions [Muthukumaraswamy et al., 2021, Butler et al., 2022] emphasise expectancy in low-dose/microdose designs. In contrast, a head-to-head double-blind RCT re-analysis found that pre-trial expectancy explained only a small, non-significant fraction of outcome variance under psilocybin (HAM-D 1.16 ± 1.05, adj. $p = 1.0$; BDI 1.14 ± 2.40, $p = 1.0$; MADRS 1.69 ± 1.81, $p = 1.0$; QIDS-SR-16 0.56 ± 1.25, $p = 1.0$; STAI-T 0.64 ± 2.66, $p = 1.0$; WEMWBS -2.96 ± 2.44, $p = 1.0$), whereas expectancy *did* significantly predict outcomes in the escitalopram arm (HAM-D -3.91 ± 0.90, adj. $p = 0.001$; BDI -5.47 ± 1.61, $p = 0.013$; MADRS -4.87 ± 1.52, $p = 0.022$; STAI-T -5.20 ± 1.68, $p = 0.028$) [Szigeti et al., 2024]. This pattern suggests that any expectancy-related contribution to macrodose psilocybin outcomes is minor, though further work is needed to refine its precise magnitude.

Placebo-controlled research using 20 mg psilocybin—highly comparable to our 19 mg dose—shows reported subjective effects corresponded with plasma levels and with nominal placebo subjective effect scores Ley et al. [2023, Fig. 1]; and also demonstrated robust separation in subjective effects between active and placebo arms Ley et al. [2023, Fig. 2]. In addition, we point to evidence from Preller et al. [2020, Fig. 1, panel E] (oral psilocybin; body-weight adjusted dose lower than our own equal to ∼14–16 mg across 70–80 kg), psilocybin meta analyses by Hirschfeld and Schmidt [2021, Figs. 1–2]; LSD meta analyses by Hirschfeld et al. [2023, Fig. 1; Fig. 2], and the psilocybin IV trial by Muthukumaraswamy et al. [2013, Fig. 2], illustrating that across designs and compounds, subjective responses under active conditions are substantially high and placebo effects are near floor, at doses lower than or comparable to ours. Our own pre-drug baseline subjective scores similarly show minimal values (11D-ASC; main-text Fig. 4d). We believe this demonstrates the limited magnitude of expectancy on subjective responses, showing that the contribution of expectancy and demand characteristics to subjective reports is minimal when contrasted with the robust effects seen at active doses,

and cannot plausibly explain the effects we report.

Objective correlates of drug action beyond expectancy.
Converging evidence links pharmacology, neural dynamics, and behaviour:
(i) Dose–response: graded increases in subjective effects with escalating dose Griffiths et al. [2011], Studerus et al. [2011], Carbonaro et al. [2020], Hirschfeld et al. [2023].
(ii) Time-dependent neural dynamics: under psilocybin, global functional connectivity changed across ~20, 40 and 70 min post-dose, with subjective effects assessed after each scan; the pattern was time-dependent and consistent with the rise toward peak effects [Preller et al., 2020]. Under LSD, antagonism (ketanserin) abolished both the connectivity changes and subjective effects, evidencing receptor specificity [Preller et al., 2018].
(iii) Behaviour: psilocybin disrupts interval timing and sensorimotor synchrony in a dose-dependent manner [Wittmann et al., 2007].
(iv) Pharmacokinetic–Pharmacodynamic (PK–PD) coupling: plasma psilocin levels correlate with neocortical $5HT_2A$ occupancy and real-time subjective intensity, and time-varying connectivity covaries with psilocin and concurrent subjective intensity ratings [Madsen et al., 2019, Olsen et al., 2022]. Active–placebo separations with plasma measurement further support pharmacological specificity [Ley et al., 2023].

To summarise, it is unfeasible for expectancy or demand characteristics to produce the subjective alteration that arises in macrodose psychedelic experiences. The conjunction of (i) ineffective placebo controls available ii) large active–placebo separations at clinical doses (including ∼20 mg), (iii) dose–response, time-locked neural dynamics and behavioural effects, (iv) consistent PK–PD coupling with subjective effect reports, and (v) subjective-effect-dependent neural embeddings we report, together supports the interpretation that our baseline-controlled findings reflect genuine psilocybin effects.

We acknowledge the reviewer's concern and, in Methods, refer the reader to the Supplementary Information where we provide a note on expectancy:

"Discussion addressing expectancy, task order and EEG timing is detailed in Supplementary Information."

Supplementary Information:

Expectancy considerations
At moderate–high psychedelic doses, effective blinding is difficult to achieve in practice because distinctive subjective effects make allocation easy to infer [Gasser et al., 2014, Studerus et al., 2011, Holze et al., 2022, 2023, Szigeti and Heifets, 2024], and suitable active placebos are lacking, with participants often discerning allocation when active placebos are used [Bogenschutz et al., 2022, Muthukumaraswamy et al., 2021]. Several further features make it doubtful that expectancy accounts for our main findings: (i) large separations in reported subjective effects between active-dose and placebo arms in both oral clinical-dose and intravenous designs [Bogenschutz et al., 2022, Muthukumaraswamy et al., 2013], including 20 mg oral psilocybin [Ley et al., 2023], which is comparable to our 19 mg dose and shows very low placebo subjective scores; dose–response and time-dependent evidence across designs further corroborates pharmacological dominance over expectancy [Hirschfeld and Schmidt, 2021, Hirschfeld et al., 2023, Preller et al., 2020, Muthukumaraswamy et al., 2013]; (ii) pharmacokinetic–pharmacodynamic coupling between psilocin exposure, receptor engagement, and time-varying neural dynamics [Madsen et al., 2019, Preller et al., 2018, 2020, Olsen et al., 2022]; and (iii) condition-separated ML embeddings appear only when subjective effects are strong (high MEQ) and are absent at baseline/low-MEQ. Consistent with this, a published re-analysis of a head-to-head

double-blind RCT found that pre-trial expectancy significantly predicted outcomes in the escitalopram arm but not in the psilocybin arm [Szigeti et al., 2024]. Although expectancy is empirically difficult to eliminate in psychedelic trials, the subjective-response-dependent emergence of structured embeddings, their absence at baseline or with low subjective effects, and the replicated convergence between eyes-open and eyes-closed conditions across modalities despite reversed order collectively make expectancy an implausible primary driver of our main results.

Minor concerns / suggestions

Fig. 6c is labeled as "Lempel-Ziv complexity difference (psilocybin minus baseline (no-psilocybin))" but I think it may be power spectra (the y axis is power).

Response: The plots in main-text Fig. 6c are power spectra, while those in main-text Fig. 6d are Lempel-Ziv complexity, and we have updated the caption accordingly.

Clarify demographic information (average age, gender ratio, exclusion criteria) in the main text.

Response: We have edited the Methods text:

"Sixty-five healthy adults (28 females) aged 18–55 (37.3 ± 10.7) with no psychedelic experience were recruited for the study."

Fig. 4g Caption – "Mutual exclusivity" implies a negative correlation, when in fact no significant correlation was found.

Response: We have removed the ambiguous phrase. We thank the reviewer for pointing this out.

Text/legends in multiple figures is so tiny that it is impossible to read (e.g. network labels Fig. 2b, all labels in Fig. 6). It appears to be 2.5 point font.

Response: We appreciate that the font is small due to space constraints, and have added an enlarged version of main-text Fig. 2b in the Supplementary for convenience. In addition, we ensure all figures are vectorised so they can be enlarged on a screen without resolution loss.

Consider adding citation for Goodwin et al., 2025 "The role of the psychedelic experience. . . " at the end of first paragraph on page 8.

Response: We have added the Goodwin et al., 2025 citation to the end of the paragraph on page 8, ending in "...ongoing debates about the necessity of the psychedelic experience for clinical efficacy (Yaden and Griffiths, 2020; Olson, 2020; Goodwin et al., 2025)".

Please define any abbreviations or nonstandard labels in the figure captions (e.g "salventattn").

Response: Corrected in captions of main-text Fig. 2, 3, and Extended Data Fig. 10, which after examining the manuscript, we determined covered all such instances.

Regarding difference in GFC between eyes open/closed, would cite Wong et al., 2013 Neuroimage.

Response: Thank you for the suggestion. Differences between eyes-open and eyes-closed rest in global measures are established in the literature you have shared (e.g., Wong et al., 2013). In our study, however, the eyes-open condition analysed here is naturalistic movie viewing rather than rest, and our primary endpoint is surface-based GFC, not global signal amplitude. To avoid conflating rest-related vigilance effects with stimulus-driven synchrony, and to keep the Results focused

on psilocybin-induced changes (as well as accommodate formatting requirements that restrict word count), we struggled to find a compatible spot for this information, and decided to not add this citation to the main text.

Fig. 5 The text references panels a-d however there are no panel labels in the figure.

Response: We've added panel labels.

Correct minor typos "anti-depresssant" on page 13

Response: Corrected typos and fixed term "anti-depressant" to "anxiolytic" because the emergency medication was a benzodiazepine. Thank you for pointing this out.

Replace "available upon request" with a DOI-linked repository in the data-availability statement.

Response: We have added the DOI-linked repository in the data-availability statement.

"All data reported has been uploaded to OpenNeuro (https://openneuro.org/datasets/ds006110/versions/1.1.0) and will be made fully open access upon publication."

We're grateful for the reviewer's detailed and thoughtful comments. They helped us shape the manuscript into a more cohesive, conceptually grounded work with clearer logic, stronger methodological justification, and improved interpretability of our core findings.

Referee 2/3

This is an ambitious open-label study in which the effects of psilocybin were investigated in a comparatively large sample of healthy volunteers (n = 60), using four distinct experimental conditions: resting state, meditation, listening to music, and watching a video. The study employed both fMRI and EEG to characterize psilocybin's effects on brain function. The paper presents a complex set of analyses. First, the authors demonstrate changes in GFC, showing that psilocybin reduces differences between eyes-closed and eyes-open conditions. Using machine learning approaches, they further show that psilocybin induces a restructuring of brain dynamics in a context-sensitive manner—i.e., aligning neural activity more closely with the sensory and psychological environment. This work is important in that it offers a shift in framing: from the view that psychedelics merely desynchronize brain activity (e.g., via increased entropy), to a more nuanced interpretation emphasizing alignment of neural dynamics with contextual and experiential factors. This reframing holds potential implications for our understanding of the therapeutic mechanisms of psychedelics

The authors may wish to address the following points, which would improve clarity, enhance interpretability, and increase the manuscript's overall readability. The points are listed in no particular order of importance.

Major concerns

2.1. *The authors should clarify that the EEG session followed the fMRI session, including in the abstract. Additionally, it remains unclear how much time elapsed between drug administration and the start of the fMRI and EEG recordings, respectively. This information could be added to Figure 1 and described in the Methods. Notably, the timing of the EEG session is not discussed anywhere, which is important given that delayed effects might be observed.*

Authors' Response:

We agree that the modality ordering and dose-to-scan intervals should be explicit across the manuscript. The fMRI session began approximately 80 minutes after oral administration of psilocybin, followed by the EEG approximately 150 minutes post-dose. We have now added this information to main-text Fig. 1 caption, clarified it in the Methods section, and added it to the abstract as requested.

Abstract:

"Sixty-two adults were scanned with functional MRI and EEG during rest and naturalistic stimuli (meditation, music, and visual), before and after ingesting 19 mg of psilocybin (functional MRI at 80 min post-dose; EEG at 150 min post-dose)."

Main-text Fig. 1:

"An open label 'baseline' (no-psilocybin) imaging session was conducted approximately one week before imaging under administration of 19 mg psilocybin. MRI and EEG imaging began ≈80 and ≈150 minutes post-dose, respectively."

Methods: "In fMRI, conditions began 80 minutes post-dose and followed a fixed order (rest → meditation → music → movie). The sequence progressed from low-stimulus to high-stimulus contexts to prioritise safety and experiential coherence for psychedelic-naïve participants. The EEG session started after a room transfer and 20–40 minutes of setup (cap placement and impedance checks), with

the movie presented first, followed by the three eyes-closed conditions (movie → rest → meditation → music)."

We also expand on the topic of delayed effects in an edit to the manuscript made in response to comment 2.13, as well as in the Supplementary:

Our EEG session sampled the late-peak/early-offset portion of the acute response. Human PK/PD data indicate that, after oral dosing, plasma psilocin Tmax typically occurs at ∼2–3 h (≈90–150 min) with inter-individual variability, and concentrations remain elevated for several hours; subjective intensity closely tracks psilocin levels and neocortical 5-HT2A engagement across this window [Holze et al., 2022, Madsen et al., 2019, Ley et al., 2023]. Consistent with this coupling, psilocybin-induced changes in large-scale network integrity/segregation have been characterised over multiple hours post-dose, indicating robust central effects throughout our EEG interval [Madsen and Knudsen, 2021].

Evidence that EEG timing is within the acute state is demonstrated between consistency between our findings and canonical electrophysiological signatures are observed in established EEG/MEG psychedelic research—most notably broadband desynchronisation with pronounced parieto-occipital alpha reductions and increased neural signal diversity/entropy [Muthukumaraswamy et al., 2013, Schartner et al., 2017, Timmermann et al., 2023].

2.2. *In the Main section (page 2), the authors write that "recent human research suggests that reductions in DMN and anterior hippocampus connectivity may contribute to the therapeutic effects of psilocybin," but then cite Reinwald (rodent study) and Siegel (healthy participants). These references do not provide direct evidence for the stated claim. Rephrasing would be advisable.*

Authors' Response:

We appreciate the reviewer for highlighting this important point and the needed revision. We agree that our original phrasing overstated the strength of evidence for a therapeutic mechanism and did not clearly distinguish between human and preclinical sources. We have revised the sentence to more accurately reflect the available data and its interpretation:

"Notably, in healthy adults psilocybin produces a persistent reduction in anterior hippocampus–DMN connectivity, and in rodents, preclinical studies report acute DMN hypoconnectivity [Siegel et al., 2024, Reinwald et al., 2023]. Although observed in non-clinical samples, this pattern has been proposed as mechanistically relevant to therapeutic effects."

This revision more precisely attributes the mechanistic proposal to the human imaging findings reported by [Siegel et al., 2024], where the observed circuit-level changes are interpreted in light of prior clinical research. The findings of [Reinwald et al., 2023] are positioned as translational support for DMN-level hypoconnectivity, without implying direct clinical inference.

2.3. *Regarding psilocybin's contribution to therapeutic effects, the observed reductions in modularity are not discussed in relation to prior work, particularly the study by Daws et al. (10.1038/s41591-022-01744-z), which linked decreased modularity with symptom improvements in patients with depression.*

Authors' Response:

This is an important clinical precedent, and we appreciate the reviewer's suggestion. We agree that the modularity finding warrants contextualisation in light of prior work. We have added a sentence to the Results section referencing the [Daws et al., 2022] study, noting that similar reductions in modularity have been reported in clinical samples and associated with longer-term symptom improvement following psilocybin therapy:

"Comparable decreases in modularity have also been reported in clinical samples, where lower post-treatment modularity was associated with longer-term symptom improvement following psilocybin therapy [Daws et al., 2022]."

***2.4**. Participant demographics: Mean age and sex distribution should be reported alongside the age range.*

Authors' Response:

We have updated the Methods section as follows: "Sixty-five healthy adults (28 females) aged 18–55 (37.3 ± 10.7) with no psychedelic experience were recruited for the study."

***2.5**. The rationale for excluding participants who used hallucinogens within the past 6 months is unclear if the sample was already restricted to psychedelic-naïve individuals. Clarification would be helpful.*

Authors' Response:

We appreciate this comment and apologise for any confusion. In the manuscript, the intended phrase "no prior psychedelic experience" was mistakenly written as "no prior psychedelic use." While our screening excluded any hallucinogen use within the past 6 months (to avoid subacute carryover/tolerance and expectancy reinforcement), and acted as an explicit lower bound threshold for exclusion, in practice, we were able to recruit a sample meeting our operational definition of "psychedelic-naïve", meaning no prior psychedelic experience *with subjective effects*, which we define as the subjective sense of altered perception in response to taking a known serotonergic psychedelic substance.

However, 3 out of 62 participants reported nominal or remote lifetime exposures that did not yield subjective psychedelic effects (n=2 self-reported ineffective microdoses; n=1 self-reported remote exposure >20 years ago with minimal recall/no recall of experience). These cases are transparently added in the Supplementary. All other participants reported no lifetime use across three checks (online screening, phone screening and SCID interviewing). We keep the details in the Supplementary table brief, and outline their verbatim reports here. Square brackets have been used in quoted material to clarify, anonymise, or correct text where necessary:

- PC-026. In 2017 "Used psilocybin, twice, microdose, around January 2017. It was summer, and I had some friends who were microdosing, and I just took a pill of some description that was apparently psilocybin mixed with a few other herbs. No effects noticed. Maybe I was paying more attention to things, but as [someone who works in the medical field], I know that this is a mechanism of placebo. I was trying to notice any perceptual changes and I [didn't] notice anything."

- PC-029. In 1998 (>20y ago) "No recollection of my experiences with psilocybin in 1998, did it twice. I remember it being quite nice. I certainly [don't] really remember what to expect from psilocybin. I just remember loving being out in the sun that day. I [don't] remember anything negative, but I do know that it can be negative."
- PC-225. >6 months pre-screen "Did take one very very old one (many years) to try it 6 months ago, but there was no psilocybin present at all or psychedelic experience."

We have corrected the language in the Introduction, changing "use" to "experience":

"We acquired the largest single-site psychedelic neuroimaging dataset to date, integrating multimodal fMRI and EEG with controlled contextual manipulations across eyes-open and eyes-closed states in a cohort with no lifetime psychedelic experience (N=62)."

In the Methods we identify our operational definition and indicate the exceptions, directing readers to the Supplementary table for details of each of these three participants self-report of ingestion and psychedelic naïvety:

"We operationally defined 'no psychedelic experience' as no prior serotonergic psychedelic use with subjective effects. During structured screening, three participants disclosed nominal or remote lifetime exposures without subjective effects: two reported ineffective microdoses, and one reported remote exposure over 20 years ago with minimal or no recall. These cases are detailed in Supplementary Information. All other participants (n=59) reported no lifetime use across online, phone, and SCID checks."

Table content as it appears in Supplementary:

Table R1: Screening disclosures of nominal/remote serotonergic psychedelic exposures, 3 participants ($\approx$ 4.8 percent of N)

Study ID	Timing	Self-Report
(PC-026)	2017	"Psilocybin, twice, microdose. No effects noticed. "
(PC-029)	1998	"Psilocybin, twice, unspecified dose. Minimal/No recollection of subjective effects."
(PC-225)	>6 months pre-screen	"Psilocybin, once, microdose. No effects noticed."

2.6*. In the Participants section (pg 12), the phrase "individual experience" appears, but the section primarily lists adverse effects. It may be clearer to rephrase this to something like: "Psilocybin was generally well tolerated, although some adverse effects were reported, including…". This would align better with the tone typically used in clinical trial reporting.*

Authors' Response:

We thank the reviewer for suggesting clearer phrasing, which we have applied:

"Psilocybin was generally well tolerated, although some adverse effects were reported, including reports of transient headaches during the night (n=3). Three participants required follow-up support

from a clinical psychologist familiar with psychedelic integration."

***2.7**. Extended Data Fig. 2 shows mean FD for the music and movie conditions only. Why are rest and meditation not included? Understanding whether head motion differed across all conditions is essential for interpreting functional connectivity results.*

Authors' Response:

As suggested, we have added the resting state and meditation conditions to the Extended Data Fig. 2 (also shown here as Fig. R8 for convenience). The original goal of this figure was to compare the two conditions with the highest head motion (music and movie), showing that there were no significant FD differences that could provide an alternative explanation to the eyes-open vs eyes-closed difference. This message remains unchanged: the resting-state and meditation conditions have lower median FD values, and these differences are not statistically significant.

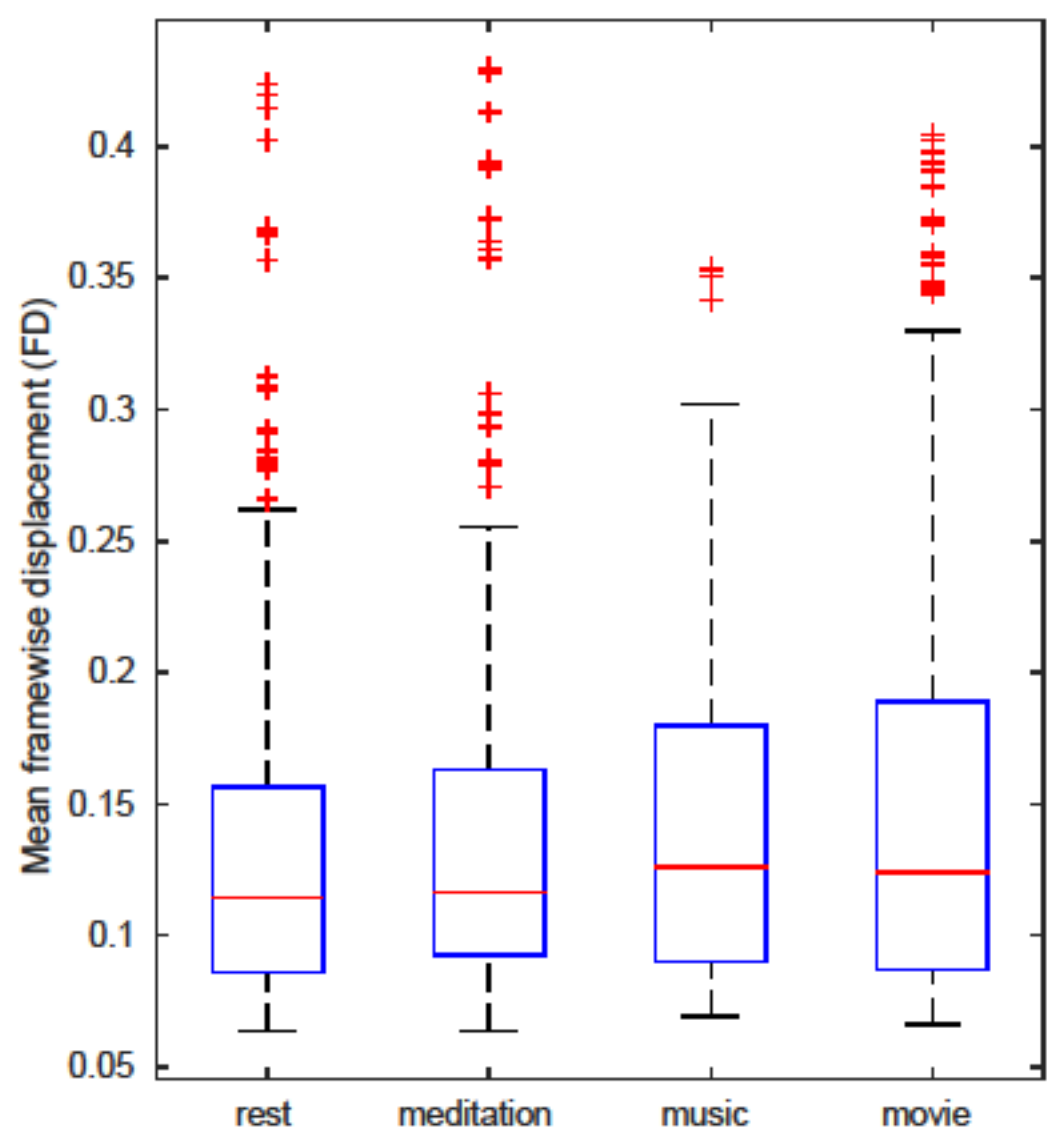

Figure R8: Head motion comparison across conditions, quantified via the mean frame-wise displacement. No statistically significant differences were observed.

***2.8**. The order of conditions differs between fMRI and EEG sessions—ending with movie watching for fMRI and beginning with it for EEG. This difference warrants clarification and possibly justification.*

Authors' Response:

We appreciate the reviewer's request to clarify and justify the difference in task order between the fMRI and EEG sessions. This design choice was deliberate and is now clarified in the Methods.

"In fMRI, conditions began 80 minutes post-dose and followed a fixed order (rest → meditation → music → movie). The sequence progressed from low-stimulus to high-stimulus contexts to prioritise safety and experiential coherence for psychedelic-naïve participants. The EEG session started after a room transfer and 20–40 minutes of setup (cap placement and impedance checks), with the movie presented first, followed by the three eyes-closed conditions (movie → rest → meditation → music)."

This reversal served multiple purposes:

The cross-modality reversal of the movie condition, combined with an inter-modality delay provided us an internal test of sequence sensitivity. The finding that psilocybin reduced differentiation between eyes-open and eyes-closed states in both fMRI and EEG, despite the reversed order, is empirical support that this effect is not attributable to task sequence (see main-text Fig. 2e (GFC histograms) and main-text Fig. 6b (EEG power spectra)).

We grouped the eyes-closed tasks (rest, meditation, music) together (in order of low to high intensity stimuli) to gradually encourage a deeper inward-focus that was both safe for the participants who were undergoing an MRI during the early phase of their first psychedelic experience and optimal for the measuring self- and boundary dissolution during experimental sequences, which can be impeded by attention-focused demands, such as instructions or attention demanding tasks such as movie-watching. As anticipated, EEG power during eyes-open movie-watching appears much closer to baseline than during eyes-closed sequences (main-text Fig. 6, all panels). These results are also consistent with [Siegel et al., 2024]'s description of ”grounding” during attention demanding tasks. Therefore, to maintain stability of the psychedelic experience, pairing eyes-open movie at the end of the MRI with eyes-open at the start of EEG allowed the attention-focus tasks of movie watching to be ”blocked” together. This improved the comparability of the eyes-open effects in later analyses, provided the aforementioned order robustness check, and—after the brief transition back to task engagement with movie watching—enabled the participant to re-experience the eyes-closed states without external cues for their attention risking interruption to the flow of the psychedelic state. Lastly, beginning with the eyes-open condition also allowed for eye movement and blink calibration.

***2.9**. In the Integration section (pg 3), it would improve clarity to specify in brackets which conditions correspond to "eyes-closed states." The finding that occipital areas show the strongest GFC decreases is difficult to interpret without knowing the specific condition(s). Structuring the description of results across eyes-closed conditions (rest, meditation, music) and providing concrete examples for each condition, as is done for EEG, would enhance readability.*

Authors' Response:

We thank the reviewer for this helpful suggestion. We agree that clarity is improved by explicitly identifying the eyes-closed conditions and providing condition-specific examples. We have revised the respective section accordingly to specify the three eyes-closed states (rest, meditation, and music) and to describe the spatial pattern of GFC reductions observed across these. We also clarify that the movie condition was eyes-open and showed a distinct pattern of GFC increases in both sensory and associative regions.

The revised text now reads: "During eyes-closed conditions (rest, meditation, and music), psilocybin reduced the GFC of sensory regions (Fig. 2a), and these reductions formed statistically significant clusters (see Fig. R11). Occipital areas showed the strongest overall reductions (percentage changes up to -39% and Cohen's $d = -0.55$), with midline ventral parietal decreases prominent during rest, meditation and music. In contrast, GFC increased in associative areas during these conditions (percentage changes up to $+72\%$, Cohen's $d = 0.53$). This evidence indicates that, during eyes-closed conditions, psilocybin suppressed the integration of sensory regions with the broader cortical network but enhanced the integration of associative regions. In contrast to the eyes-closed conditions, movie-watching increased GFC in both sensory and associative areas (Fig. 2a), enhancing global synchrony."

Please note, figure links in the quoted text relate to the main manuscript.

2.10. *When discussing GFC findings, the authors may wish to mention other studies using similar metrics such as global correlation (10.1111/acps.12818; 10.1016/j.euroneuro.2021.06.001), degree centrality (10.1038/s41380-024-02734-y, with and without GSR), or functional connectivity density (10.1016/j.cub.2016.02.010). Contextualizing findings is important, especially as the literature is limited.*

Authors' Response:

We thank the reviewer for this thoughtful suggestion and agree that contextualising our GFC results within the broader literature is important. We carefully examined each of the studies cited and now include a concise manuscript addition summarising how their findings converge with our own, while clarifying key methodological differences that limit direct comparability.

All four cited studies employ whole-brain connectivity or connectedness metrics—such as global correlation, functional connectivity density, or degree centrality—that estimate how strongly each brain region is functionally coupled to the rest of the brain. While these metrics vary in mathematical formulation and spatial implementation, they share conceptual overlap with our surface-based GFC measure (defined as the mean BOLD correlation between each cortical vertex and all others). To be thorough, we summarise each study's method and relevance in turn:

[Tagliazucchi et al., 2016] (LSD, anterior increase): This study employed a parcellated global connectivity metric (computed across 401 ROIs), conceptually related to functional connectivity density (FCD). Scans were acquired during eyes-closed rest. LSD was associated with increased global connectivity in associative cortex and thalamus, as well as altered sensory coupling. While the metric differs from our vertex-wise GFC, the spatial pattern—particularly associative increases—is directionally consistent with our findings under psilocybin.

[Müller et al., 2017] (LSD, subcortical): Here, the authors used voxel-wise global correlation, a measure of average correlation between each voxel and the rest of the brain. They reported increased network centrality in the thalamus and dorsal striatum, highlighting cortico–basal ganglia–thalamo–cortical (CSTC) involvement under LSD. Scans were acquired during eyes-closed rest. While our surface-based GFC analysis is limited to the cortex and cannot directly visualise subcortical structures, we performed a qualitative cross-check using our ROI-to-ROI matrix (332 parcels including subcortex). This revealed that the caudate, putamen, and thalamus exhibited the highest average functional connectivity across all parcels—providing complementary support for Müller's findings. We retain this observation in the response letter only, in order to preserve manuscript focus.

[Madsen et al., 2021] (psilocybin, anterior increase): This study related GCOR (global correlation) and LCOR (local correlation) during eyes-closed rest to plasma psilocin levels and subjective drug intensity. Positive GCOR associations were found in frontoparietal regions (DAN/ECN) and thalamus. While the study design focused on within-session pharmacokinetic and experiential correlations (rather than condition-wise contrasts), the implicated networks—particularly associative control systems—are consistent with the anterior increases we observe in surface GFC under psilocybin.

[Avram et al., 2025] (LSD, MDMA, d-amphetamine, posterior decrease): This recent study employed voxel-wise degree centrality (DC) with and without global signal regression (GSR), alongside cluster-level inference. Across all compounds during eyes-closed rest, they observed decreased visual/occipital centrality, and for LSD, increased thalamic and basal ganglia centrality. Their methods—voxel-wise DC with volumetric smoothing, z-standardisation, and GSR variants—differ substantially from our own, which use surface-based vertex-wise GFC and primarily GSR-free maps. These an-

alytic differences likely explain their more conservative spatial patterns. Nevertheless, the posterior sensory reductions are directionally aligned with our findings, strengthening their potential generality.

While these studies span different analytic families—GCOR, FCD/global connectivity, and degree centrality—they share a conceptual emphasis on how strongly a brain region is connected to the rest of the brain. Our surface GFC measure falls within this family, albeit with key distinctions: we restrict to cortical vertices, adopt a surface-based framework, and report primary results without GSR or z-scoring, which we show can distort GFC interpretation (see Methods: A note on global signal regression and z-scoring the global functional connectivity). Given these differences in metric, spatial domain, preprocessing, and experimental design, we acknowledge these studies succinctly, noting their convergence in identifying associative/global increases and sensory/posterior reductions in functional connectivity under serotonergic compounds.

We added the following sentence at the end of the main paragraph describing GFC changes in eyes-closed conditions:

”These eyes-closed shifts in GFC are directionally consistent with prior global connectivity studies under psychedelics during eyes-closed rest, which commonly report increases in associative/frontoparietal systems [Tagliazucchi et al., 2016, Madsen et al., 2021] and, in degree-centrality analyses, decreases in sensory/visual areas [Avram et al., 2025]. However, differences in metrics (such as global correlation, functional connectivity density, and degree centrality), spatial domains (volumetric vs. surface), and subcortical coverage limit direct comparisons.”

We believe this addresses the reviewer’s request with appropriate precision while preserving the flow and specificity of our manuscript.

2.11. *The manuscript highlights reduced within-network connectivity in the DMN and dorsal attention network (pg 4), but visually, the most pronounced effect appears in the VIS (main-text Fig. 2b). This discrepancy should be addressed.*

Authors’ Response:

We thank the reviewer for this helpful observation. The findings referenced are based on our within-network functional connectivity comparison using the Mann–Whitney U test, reported in the Supplementary Information. You are correct that the within-network reduction in the visual network appears most pronounced in main-text Fig. 2b, particularly during eyes-closed conditions. However, our original phrasing described the pattern of statistical significant reductions across all conditions (eyes-closed rest, meditation, music and eyes-open movie) without clarifying this distinction, which may have caused confusion. We have revised the text to reflect this more precisely, specifying that all networks showed reductions during eyes-closed conditions (rest, meditation, and music), while only the default mode network and dorsal attention network showed significant reductions during eyes-open movie viewing. The revised sentence reads:

“Analysis of our larger dataset revealed that psychedelic-induced reorganisation of brain network architecture was primarily attributable to decreased within-network connectivity (Fig. 2b), with significant reductions observed in all the networks across eyes-closed conditions (rest, meditation and music), and only in the DMN and dorsal attention network during eyes-open movie watching (See Supplementary Information).”

We hope this resolves the discrepancy noted and improves the clarity of the manuscript.

2.12. *The lack of differences between the meditation and control group is intriguing, particularly in light of previous work reporting differences (10.1016/j.neuroimage.2019.04.009, 10.1038/s41598-024-55726-x). Even a brief discussion of potential synergistic or non-additive effects would be valuable.*

Authors' Response:

We appreciate this suggestion. Prior studies reporting meditation–psilocybin synergy typically involve experienced meditators engaging in intensive retreat formats where practice is deeply integrated with the dosing context, e.g., [Smigielski et al., 2019, Singer et al., 2024]. In contrast, our participants were meditation-naïve and assigned to a standard MBCT program delivered prior to the session. While we included a guided meditation scan under psilocybin, we found no significant between-group differences. This pattern suggests that synergistic effects may depend less on formal instruction and more on deep, sustained engagement with meditation practice, which was not present in our cohort. We have added text to the manuscript Discussion to elucidate these points.

"Previous studies reporting synergistic effects between meditation and psilocybin have typically involved experienced practitioners engaged in intensive retreat-based practice [Smigielski et al., 2019, Singer et al., 2024]. In contrast, our study participants were assigned to a standardised mindfulness-based cognitive therapy program that was not designed to integrate with the psychedelic session. Despite including a guided meditation scan, we observed no between-group differences under psilocybin—suggesting that synergy may require long-term meditative engagement, self-directed commitment, or a program explicitly tailored to support the psychedelic experience."

Outside of this addition, we made only minor wording edits to the respective meditation text to improve clarity, precision and remove redundancy. This also reduced word count, without altering results or conclusions.

2.13. *How do the current EEG results (power spectrum and LZ complexity) compare to those reported by Timmermann et al. (10.1073/pnas.2218949120) for DMT under resting conditions? A short comparison would help situate the findings.*

Authors' Response:

We thank the reviewer for this helpful suggestion. Both our study and [Timmermann et al., 2023] report decreased alpha power and increased signal diversity (Lempel–Ziv complexity) under psychedelic administration. These effects were observed under distinct conditions (intravenous DMT during the acute peak, and oral psilocybin approximately 2.5 hours post-dose) suggesting that such neurophysiological signatures may be generalisable across serotonergic psychedelics and persist beyond the immediate peak into later phases of the experience. This convergence strengthens the interpretation that reduced alpha rhythmicity and elevated neural complexity reflect sustained properties of the psychedelic state.

[Timmermann et al., 2023] also report increased gamma-band power under IV DMT; in our dataset gamma increases were present but comparatively less prominent and more spatially limited. Differences of this kind may originate from variation in compound (IV DMT vs oral psilocybin), time from peak, and EEG methodology (e.g., short-epoch vs full-trial LZ estimation and associated statistics).

For the main text, we have added the following concise comparison to the Discussion:

"Comparable decreases in alpha power and increases in signal diversity have also been observed under intravenous administration of the serotonergic psychedelic N,N-dimethyltryptamine (DMT) Timmermann et al. [2023] , suggesting these may be conserved markers of serotonergic psychedelic action, despite differences in compound, route of administration, timing, and EEG methodology."

2.14. *The following sentence (pg 10) seems ambiguous and may overreach given the data presented: This pattern supports interpreting embeddedness as a state central to psychedelics' therapeutic relevance by addressing distress arising from separation, which may underlie the therapeutic efficacy of psychedelics in reducing existential distress (Griffiths et al., 2016; Ross et al., 2016). It is unclear what is meant by separation (from what?). The interpretations should be revised.*

Authors' Response:

We thank the reviewer for noting the ambiguity. In response, we now define separation as the pervasive, felt disconnection between self and world—including self–other boundaries and rigid self-referential attachment—and clarify that embeddedness refers to a transient reduction of this separation. We present this as an interpretive framework that is consistent with reported reductions in death-related anxiety during psilocybin-assisted therapy [Griffiths et al., 2016, Ross et al., 2016], and is grounded in the subjective effects observed in our cohort. We have revised the respective text accordingly, as follows:

"This pattern supports interpreting embeddedness as a state central to psychedelics' therapeutic relevance by addressing distress arising from separation . We use *separation* to mean the pervasive, felt disconnection between self and world (including self–other boundaries and rigid self-referential attachment). Interpreted this way, "embeddedness" marks a transient reduction of that separation—an existentially salient state that may help explain reported reductions in death-related anxiety and existential distress following psychedelic treatment [Griffiths et al., 2016, Ross et al., 2016], and, in our non-clinical cohort, is consistent with self- and boundary-dissolving experiences (Fig. 4c–g; Extended Data Fig. 10), increased personal meaning and death acceptance (Extended Data Fig. 11), and greater nature relatedness (Extended Data Fig. 12)."

2.15. *In the Context shaped connectivity section (pg 11), the authors interpret the findings as reflecting reduced sensory and increased associative integration. It would be useful to comment on the specificity of these patterns, especially in light of recent work suggesting that similar effects may not be unique to psychedelics (e.g., 10.1038/s41380-024-02734-y).*

Authors' Response:

We thank the reviewer for raising the issue of specificity. In response, we have added a concise note to the Context-shaped connectivity section, acknowledging that posterior/visual global-connectivity decreases have also been observed under LSD, MDMA, and d-amphetamine using voxel-wise degree centrality [Avram et al., 2025], which indicate that this sensory pattern is not exclusive to psychedelics. We appreciate the suggestion to add this useful context. In examining the cited study, we find it differs from ours in several analytic respects, including volumetric, voxel-based degree centrality, z-standardised DC maps and cluster-level family-wise error correction. In light of these differences and to preserve the flow of the Discussion section, we briefly and directly acknowledge

the overlap:

“Similar posterior/visual degree-centrality reductions have been reported under LSD, MDMA and d-amphetamine Avram et al. [2025], indicating that sensory global-connectivity decreases can occur outside the class of serotonergic psychedelics.”

2.16. *It is notable that the movie condition increased GFC yet showed reduced EEG power effects. This raised the question of “grounding,” as discussed in Siegel et al. (10.1038/s41586-024-07624-5) although this does not align neatly with the fMRI results. The authors might wish to comment on this divergence.*

Authors’ Response:

We appreciate the reviewer’s point. In the movie condition, psilocybin increased global functional connectivity (GFC), indicating greater large-scale synchrony, while EEG spectral changes were attenuated and Lempel–Ziv complexity was reduced relative to eyes-closed conditions. These measures capture different properties—correlation-based synchrony versus oscillatory amplitude and complexity—and are not necessarily expected to covary. Moreover, even if the measures do vary together, they may not imply they reflect the same underlying process, such as attenuation. Therefore, we are cautious in speculating whether the increased brain-wide GFC reflects attenuation, or whether it may reflect a modulation of brain activity unrelated to attenuation. During movie viewing, GFC estimates were also sensitive to methodological choices (e.g., z-scoring and global signal regression; see Methods, “A note on global signal regression and z-scoring of global functional connectivity”), warranting caution in our interpretation. By contrast, the attenuation observed in EEG during movie viewing was consistent with a shift towards baseline activity, suggesting a reduced expression of psilocybin effects during externally oriented attention. Based on these observations, we confine discussion of GFC synchrony and attenuation in the movie condition to the response letter and revise our results to note the parallel between [Siegel et al., 2024]’s findings and the attenuation observed in EEG during movie viewing.

“We found that psilocybin expanded the power spectrum, with decreases in theta, alpha, and, to a lesser extent, beta power, accompanied by modest gamma increases during eyes-closed conditions, concentrated in frontal regions. These became attenuated during movie-watching, with increases localised to early visual areas, indicating condition-dependent topography (Fig. 6a). This attenuation in power and complexity during movie viewing, relative to eyes-closed tasks, is consistent with reduced network-level disruption during externally directed attention, as previously shown using fMRI functional connectivity Siegel et al. [2024].”

2.17. *The authors should consider speculating on how psychedelic-induced therapeutic effects might be interpreted in light of this new framework. So far, psychedelics have been thought to desynchronize brain activity, temporarily loosening canonical networks and allowing for novel configurations to emerge. This paper challenges that view by demonstrating that psilocybin reorganizes neural dynamics in a context-sensitive manner—suggesting a flexible reorientation toward both external stimuli and internal states. This raises the intriguing possibility that therapeutic effects may stem not merely from a dismantling of existing patterns, but from the brain’s enhanced ability to dynamically align with salient contextual or experiential inputs. Such flexible realignment could be particularly relevant in the context of psychedelic-assisted psychotherapy.*

Authors' Response:

We thank the reviewer for prompting this important synthesis and for their clear articulation. Indeed, our findings suggest that the therapeutic potential of psychedelics reflects not only a loosening of entrenched neural patterns, but also an increased capacity for the brain to fluidly align with salient experiential inputs. In response, we have revised the Discussion summary to incorporate this interpretive dimension, clarifying that, beyond the loosening of entrenched patterns, psilocybin produces context-sensitive reorganisation that aligns brain activity with external stimuli and internal states:

"Our findings extend prevailing accounts that emphasise desynchronisation and loosened network organisation by showing that psilocybin reorganises neural dynamics in a context-sensitive manner, enabling flexible alignment of brain activity with internal states and external stimuli during the psychedelic experience. This dynamic alignment provides a systems-level rationale for both how the deliberate structuring of affective, interpersonal, and sensory elements in psychedelic-assisted psychotherapy can shape neurobiological and behavioural outcomes, and for the emergence of self- and boundary-dissolving phenomena."

For flow, and to avoid redundancy, this addition necessitated the adjustment of existing text in the Discussion section: *Context shaped connectivity*. The revised text, which precedes the revised Discussion summary is demonstrated here: "Machine-learning embeddings distinguished patterns of psychedelic brain activity across experimental contexts (rest, meditation, music, movie), with classification accuracy scaling with the intensity of acute subjective experience—particularly positively felt, immersive self- and boundary-dissolving dimensions (e.g., mystical, blissful, unity). Together, increased integration of cortical–subcortical connectivity—including interactions between systems that ordinarily segregate internal (self-referential) and external (environment-focused) processing—modelled with TAVRNN, and the condition separability revealed by CEBRA, converge to support the construct of embeddedness: a state in which individuals feel fundamentally part of their environment, marked by diminished self–world separation. These signatures were detectable at the individual level and associated with next-day mindset improvements, providing a non-verbal, brain-derived marker that links subjective quality of experience to large-scale reorganisation of brain connectivity."

Minor points

1. *On pg 13 under Reported measures, remove "(n=)" from the sentence: "If they responded yes (n=), they were then asked..."*

Response: Completed.

2. *On pg 13 in the MRI acquisition section: Why was the GRAPPA acceleration factor changed from 2 (baseline) to 3 (psilocybin session)? Please clarify.*

Response: The GRAPPA acceleration factor was increased to 3 under psilocybin, compared to the baseline acceleration factor of 2, for faster acquisition of structural T1 scan only (5 mins, 12 sec at the baseline compared to 3 min 52 sec on administration day) to minimise the overall scan duration under the acute effects of psilocybin. All functional scans under psilocybin still used the same acceleration factor as the baseline.

We have updated the respective MRI acquisition Methods text:

"The parallel acquisition technique was GRAPPA. For the structural T1-weighted scan only, the acceleration factor (PE) was 2 at baseline (5:12) and 3 on the psilocybin administration day (3:52) to

minimise time under drug."

3. *For Extended Data Fig. 1: the legend should include the symbols used to denote conditions and L/R for hemispheres.*

Response: Completed.

4. *On pg 16 in the section Tavrrn "FC" is introduced but the abbreviated form has already been used several times before this section – e.g., Parcel-wise FC.*

Response: Completed, and confirmed that the first use of functional connectivity comes with an abbreviation (FC).

5. *Presumably a typo on pg 6: "¿9/10" – please correct.*

Response: Fixed.

We appreciate the clear and practical feedback provided by the reviewers. It helped us tighten the structure, clarify key details, and ensure that our interpretations are better framed and appropriately grounded.

Referee 4

Psychedelics modulate perception and self-awareness by disrupting the brain's integration of internal predictive models with external sensory information, a process thought to be primarily mediated through agonism of the serotonin 5-HT2A receptor. This disruption is claimed to induce functional plasticity and reconfiguration of brain connectivity, particularly within and between associative and sensory networks. These changes (under certain controlled conditions) are often experienced as deeply meaningful and therapeutically beneficial. Consequently, emerging evidence indicates clinically meaningful reductions in symptoms associated with depression, anxiety, and substance use disorders. Leading theoretical frameworks suggest that psychedelics exert their effects by relaxing hierarchical constraints within the heteromodal and highly integrative default mode network (DMN). This disinhibition is thought to facilitate the emergence of novel patterns of functional connectivity, which may underlie the immersive and boundary-dissolving nature of the psychedelic experience.

Psychedelic studies often suffer from small sample sizes due to regulatory and logistical constraints. A dataset with 62 participants presented here, especially with no prior psychedelic use, provides statistical power and generalisability that are uncommon in the field. The authors also incorporated a range of contextual conditions—including rest, meditation, music listening, and movie viewing, which provides valuable opportunities to investigate how different environmental and cognitive contexts modulate the psychedelic experience. Combining fMRI and EEG data allows the authors to present results from slow, large-scale network changes (seen in fMRI) with fast oscillatory dynamics (seen in EEG), which is useful for understanding altered states of consciousness. So overall a unique dataset with huge potential that will surely materialise when the data will be made "fully open access through an OpenNeuro repository upon publication". For this reason alone, the manuscript deserves publication.

Beyond this, the authors also present evidence that under psilocybin, brain dynamics shift toward greater integration of anterior-associative processes, especially during positively felt states. This reorganisation emerges according to the authors, as a context-sensitive, psychologically adaptive neural signature, offering insight into how psychedelics may facilitate therapeutic transformation through altered brain network architecture.

The manuscript is well composed, with a solid narrative and a commendable integration of complex methodologies. However, the interpretation of the findings occasionally drifts into overly ambitious territory, where the prose tends to outpace the empirical findings. While the concept of "embeddedness" is intriguing and the contextual sensitivity of the psychedelic state is well-argued, some of the conclusions are reaching beyond what the data can firmly support. I will try to present some examples of this in my detailed comments below.

The statistical robustness of the results presented in this study is strong, reflecting both methodological rigor and the advantages of a large, well-controlled sample. Across modalities (fMRI, EEG, ML embeddings), the authors report significant effects with solid effect sizes. The use of Bayesian model averaging, non-parametric tests (e.g., Mann-Whitney U), and machine learning classification accuracy further strengthens the inferential validity. Importantly, the authors also address potential confounds (e.g., head motion, global signal regression) and demonstrate replicability across conditions and individuals, enhancing confidence in the generalisability of their findings.

Specific comments:

4.1. *Were the multiple fMRI echos combined into one set of images? How did the authors ensure that the components identified and removed were indeed noise, rather than neuronal signals of in-*

terest? To my knowledge, Kundu et al. recommend the use of ICA for denoising; however, within this experimental framework, there is a risk that meaningful signal—such as a potential subdivision of the default mode network (or any other network) following psilocybin administration—could be misclassified as noise, particularly if one subnetwork appears smaller or less spatially extensive. Was any quality control performed to mitigate this risk, such as visual inspection of removed components or analysis of their spectral characteristics?

Authors' Response:

We used a standard multi-echo preprocessing pipeline with tedana [DuPre et al., 2021], which combines the echoes using T2*-weighted optimisation and classifies components via ME-ICA based on their TE-dependence. Components flagged as non-BOLD were not discarded, but included as nuisance regressors in a GLM applied to the optimally combined data. This conservative strategy reduces artefact without subtracting spatial maps to minimise the potential removal of overlapping neural signal [Kundu et al., 2012].

To mitigate the risk of misclassifying meaningful neural activity, such as altered or spatially constrained sub-networks, we performed visual inspection of the component spatial maps and time series. No components with clear network structure or BOLD-like characteristics were flagged for removal. Moreover, the classifier itself errs on the side of retaining ambiguous components.

These preprocessing steps are documented in our dataset descriptor [Novelli et al., 2025] and are supported by improvements in tSNR (Fig. R7). We believe this pipeline provides a rigorous yet conservative approach appropriate for studying complex brain dynamics under psychedelics.

4.2. *Did the authors use any temporal filtering for the fMRI data? High-pass filtering is commonly used in task-fMRI to remove low frequency scanner drifts for example. Did the denoising process used remove particularly high or particularly low frequencies?*

Authors' Response:

In our preprocessing, low-frequency components, particularly scanner drift and linear trends, were implicitly removed during the denoising stage. Specifically, after optimal echo combination, we regressed out white matter, CSF, head motion parameters, and non-BOLD components identified by ME-ICA (via tedana). This regression step naturally eliminates very low-frequency artefacts without requiring an explicit high-pass filter.

We confirmed this in a related preprint describing the dataset [Novelli et al., 2025], where spectral analysis showed that multi-echo ICA effectively suppresses non-neuronal low-frequency fluctuations while preserving the broad spectral range of BOLD signal. For clarity, we reproduce the relevant figure here (See Fig. R9) and have added the following note to the Methods section:

"Very low-frequency components (especially the linear trend) were implicitly filtered out after cleaning [Novelli et al., 2025], which provides further evidence that multi-echo ICA was able to identify spurious components in the data."

4.3. *Page 2: "Psychedelics such as psilocybin disrupt these interactions by acting as an agonist at the serotonin 5-HT2A receptor, inducing structural and functional plasticity." Please include some details on structural plasticity.*

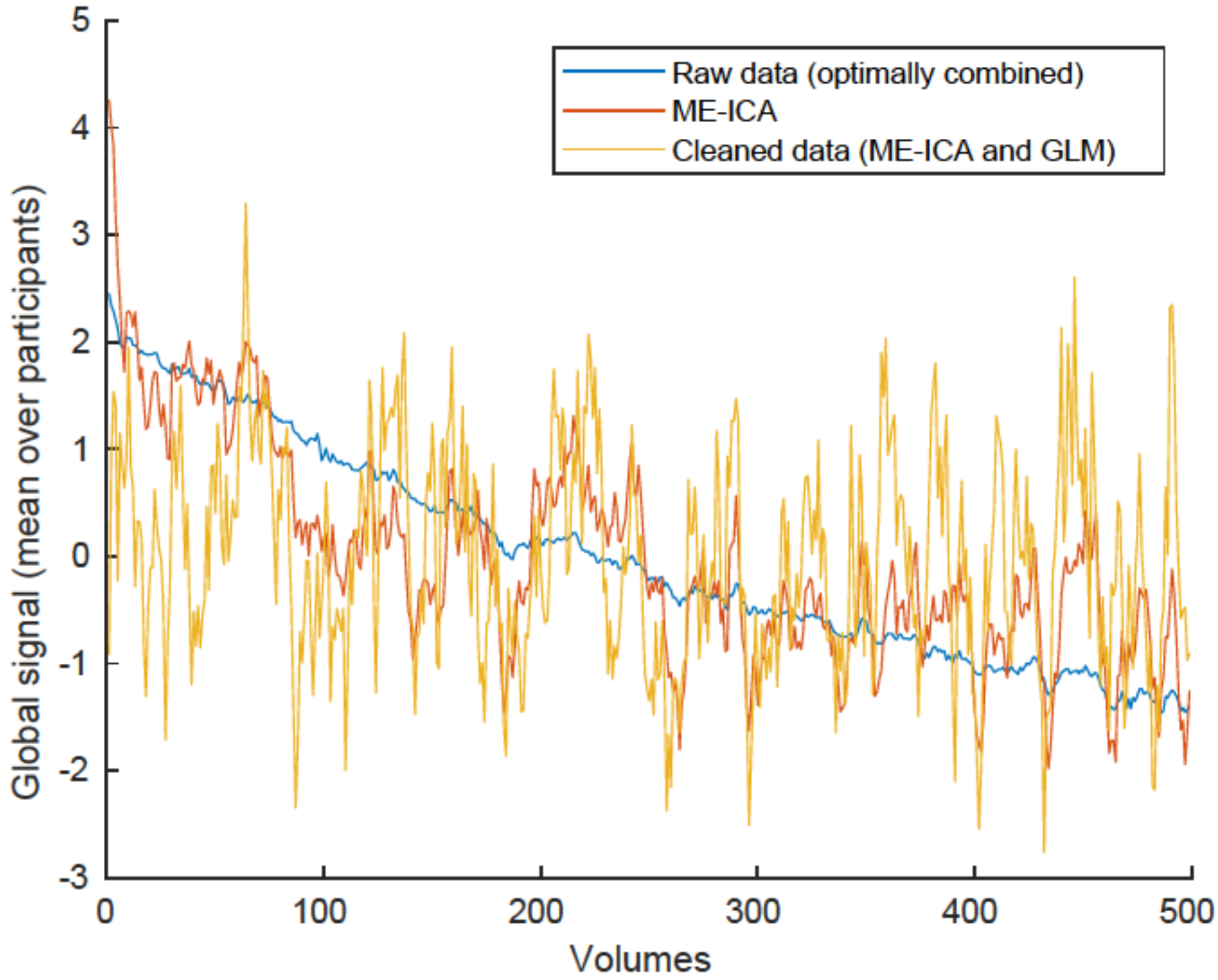

Figure R9: The slow drift in the global signal was removed by cleaning the data via a GLM that included traditional regressors (head motion, white matter and cerebrospinal fluid signals), and the non-BOLD components identified via multi-echo ICA (ME-ICA).

Authors' Response:

We thank the reviewer for raising this important point. We have now added a concise clause to the manuscript that specifies the nature and timescale of psilocybin-induced structural plasticity, while clearly indicating its preclinical basis and mechanistic relevance for lasting effects:

"Psychedelics such as psilocybin disrupt these interactions by acting at the serotonin $5\text{-}HT_{2A}$ receptor, inducing structural and functional plasticity in preclinical models—rapid, 5-HT2A-dependent mPFC spinogenesis that persists for weeks and is mechanistically linked to enduring behavioural effects Shao et al. [2021, 2025b]—that can reshape macro-level connectivity [Ly et al., 2018, Kwan et al., 2022, Burt et al., 2021, Schmidt et al., 2024]."

Supporting this addition, rodent studies show that a single psilocybin dose induces rapid spinogenesis in medial prefrontal layer-5 pyramidal neurons, yielding $\sim$10 percent increases in dendritic-spine density and head size within 24 hours, with a subset of new spines persisting for at least a month [Shao et al., 2021]. Recent cell-type–resolved work further demonstrates that these effects are 5-HT2A-dependent, occur in both pyramidal-tract and intratelencephalic neurons, and that disrupting pyramidal-tract output abolishes the long-term behavioural effects of psilocybin—establishing a mechanistic link between structural plasticity and enduring action [Shao et al., 2025a].

For completeness, we also note convergent large-animal evidence of increased presynaptic density following psilocybin administration, measured via SV2A autoradiography in pigs (hippocampus +4.42 percent at 1 day, +9.24 percent at 7 days; prefrontal cortex +6.10 percent at 7 days) [Raval et al., 2021]. Although direct evidence of dendritic spinogenesis in humans is not yet available, these findings support the interpretation that psilocybin engages plasticity mechanisms with relevance for sustained therapeutic effects. Complementing these animal data, human iPSC-derived cortical neurons exhibit

psilocin-induced neuroplasticity in vitro—including 5-HT2A redistribution, increased BDNF, dendritic complexity, and enhanced synaptic/network activity ([Schmidt et al., 2024]; reviewed preprint).

***4.4**. Page 2: "... research suggests that reductions in DMN and anterior hippocampus connectivity may contribute to the therapeutic effects of psilocybin (Siegel et al., 2024; Reinwald et al., 2023). Are the authors suggesting this to be an overarching mechanism or particular to a specific disorder?*

Authors' Response:

We appreciate this insightful question. We consider reductions in anterior hippocampus–DMN connectivity to reflect a *transdiagnostic circuit-level feature* that may be relevant across internalising disorders, particularly those involving maladaptive self-referential processing.

However, we acknowledge that this interpretation remains provisional, as current evidence derives primarily from non-clinical samples [Siegel et al., 2024, Reinwald et al., 2023].

Accordingly, we revised the quoted sentence to more clearly situate the proposed mechanism within the limits of current evidence:

"Notably, in healthy adults psilocybin produces a persistent reduction in anterior hippocampus–DMN connectivity, and in rodents, preclinical studies report acute DMN hypoconnectivity [Siegel et al., 2024, Reinwald et al., 2023]. Although observed in non-clinical samples, this pattern has been proposed as mechanistically relevant to therapeutic effects."

This revision avoids overstating the generalisability of current findings while still noting their potential mechanistic significance.

***4.5**. Page 3: Section on "Spatially and context-dependent increase of BOLD signal variance". Why do they authors compute standard deviation but really discuss entropy? I am not sure that the references in this paragraph associate variability with entropy "Increased BOLD signal variability under psychedelics has been commonly associated with increased entropy (Carhart-Harris et al., 2014; Herzog et al., 2023; Viol et al., 2019)".*

Authors' Response:

We agree that standard deviation and Shannon entropy are formally different measures. However, under the assumptions relevant for BOLD time series—namely continuous, approximately unimodal distributions with finite variance—there is a monotonic relationship between the two [Chung et al., 2017]. This means that increases in standard deviation can be interpreted as a proxy indicator of entropy under the stated distributional assumptions.

We also clarify that the prior studies we cited did not compute Shannon entropy directly on BOLD signals. Instead, they applied entropy measures to derived signals, such as intra-network synchrony, power spectra, or model-derived neuronal firing rates. In light of this, we decided to refine the citations supporting our discussion of entropy. We have now revised both the citations and the framing of the text for precision. The updated references draw from studies that measured signal diversity and entropy across various modalities, including EEG/MEG signal diversity [Schartner et al., 2017, Timmermann et al., 2019], fMRI sample entropy [Lebedev et al., 2016], and model-derived firing-rate entropy [Herzog et al., 2023]. Each of these studies reports increased entropy or complexity under

psychedelics, supporting the interpretation that greater signal variability reflects increased neural uncertainty or diversity, consistent with an entropy increase.

To avoid confusion, we revised the manuscript text accordingly. The updated text reads:

"Increased BOLD signal variability under psychedelics is consistent with increased Shannon entropy, as the BOLD-signal distribution is unimodal with finite variance (see Methods). This finding complements and aligns with previous studies that report increased entropy across modalities, including EEG/MEG signal diversity, fMRI sample entropy, and model-derived neuronal firing-rate entropy [Schartner et al., 2017, Lebedev et al., 2016, Timmermann et al., 2019, Herzog et al., 2023]. "

We also updated Methods section to reflect this clarification:

"While standard deviation and Shannon entropy are distinct, there is a monotonic relationship between the two when the data distribution is Gaussian. More generally, these two quantities are tightly related when the data distribution is continuous, unimodal, and has finite variance, such as in fMRI data. Variance is bounded above and below by scaled entropy powers, so that as one increases, the other is constrained to increase within bounded limits [Chung et al., 2017]. Accordingly, under these conditions, greater standard deviation corresponds to greater Shannon entropy."

4.6*. Page 6: "These findings suggest embeddings are indicative of the profound transformative qualities of the psychedelic experience unique to serotonergic psychedelics." I find this statement challenging to reconcile with the presented evidence. There wasn't any profoundness statistically tested here and neither were non-serotonergic psychedelics. Furthermore, that particular paragraph repeats the idea that classification accuracy tracks subjective experience multiple times, without adding new information.*

Authors' Response:

We thank the reviewer for this helpful comment. While profoundness of subjective effects can be inferred from participant ratings and is cited in a qualitative report, we agree that the term "profound" and the "unique(ness) to serotonergic psychedelics" were not directly tested by the presented analyses, and that the paragraph in question repeated points already made regarding the association between classification accuracy and subjective experience.

We have therefore removed the identified statement and find that no replacement text was required to align with the reviewer's accurate observation that this section contained redundancy. Specifically, the following sentence has been *deleted* to improve clarity:

"These findings suggest embeddings are indicative of the profound transformative qualities of the psychedelic experience unique to serotonergic psychedelics (i.e., self- and boundary-dissolving effects), as well as positive psychological adjustment, which were correlated, suggesting they may predict the translation of 'embeddedness' (see section on embeddedness in brain and perception) experienced during the acute psychedelic state to the period after psilocybin."

The section reads more clearly as a result, and the interpretation remains appropriately confined to the quantitative associations between embedding separability and the reported self-/boundary-dissolving subjective effects.

Additionally, for tighter alignment between the findings and Discussion statements, we have removed

the term “profound” from the Discussion excerpts ”profound self- and boundary-dissolving” and “reflecting profound interconnectedness”. We demonstrate the revised text below:

“CEBRA embeddings showed that the highest classification accuracy covaried with the occurrence of intense, positively felt, self- and boundary-dissolving subjective effects”

These embeddings distinguished patterns of psychedelic brain activity across experimental contexts (rest, meditation, music, movie), with classification accuracy scaling with the intensity of acute subjective experience—particularly positively felt immersive self- and boundary-dissolving dimensions (e.g., mystical, blissful, unity).

***4.7**. Page 9. The Precuneus is considered part of the DMN and it’s highly relevant in this study (e.g. Lyu D,. . . Parvizi J Neuron 2023) why omit it from the DCM model? Also not clear whether the authors modelled many to many interactions in their model or a predetermined set of hypothesis driven interactions.*

Authors’ Response:

We agree that the precuneus is a relevant and well-supported node within the DMN, particularly in relation to bodily-self processing, as demonstrated by [Lyu et al., 2023]. More broadly, DMN nodes interact differentially with other systems [Uddin et al., 2009], and widely used parcellations support multiple valid operationalisations—PCC-centric, precuneus-centric, or combined posterior-midline [Thomas Yeo et al., 2011, Andrews-Hanna et al., 2010, Utevsky et al., 2014].

In this study, we selected the posterior cingulate cortex (PCC) as the posterior-midline DMN node to align with our focus on changes to self-related cognition across sensory and cognitive contexts. The PCC is well established in cognitive neuroscience as a core hub of internally directed and self-referential processes [Leech and Sharp, 2014], and has been prominently implicated in psychedelic research [Carhart-Harris et al., 2012, Muthukumaraswamy et al., 2013, Smigielski et al., 2019]. This choice is also consistent with our prior modelling work, which likewise adopted a PCC-based posterior DMN representation [Preller et al., 2019, Zhou et al., 2018, Stoliker et al., 2023, 2024b].

Additionally, we designed the model with DCM-specific considerations in mind. The PCC and precuneus are anatomically adjacent and partially overlapping, and extracting timeseries from both regions can introduce collinearity, leading to less stable posterior estimates in fully connected DCMs [Zeidman et al., 2019]. Including both would also have increased the number of regions from 6 to 7, expanding the number of directed connections from 30 to 42 (49 including self-connections) and increasing the risk of over-parameterisation in our multi-condition PEB analysis [Seghier and Friston, 2013, Friston et al., 2016].

To clarify our model architecture, as suggested above, the DCM was a fully connected model (all interactions, including self-connections). This has been added to the Methods: ”Subject-level fully connected DCMs were combined at the group level with Parametric Empirical Bayes...” and to the caption of the DCM figure: “The model was specified as fully connected, allowing all possible directed interactions between regions.”

Finally, we acknowledge the importance of the anterior precuneus in bodily-self representations and its potential relevance for future psychedelic studies. We have added the following note to the extended DCM section of Supplementary Information:

Multiple valid operationalisations of the DMN exist (e.g., PCC-centric, precuneus-centric, or combined posterior-midline schemes) [Thomas Yeo et al., 2011, Andrews-Hanna et al., 2010, Utevsky et al., 2014]. We selected the posterior cingulate cortex (PCC) as the posterior-midline DMN node to align with our focus on changes to self-related cognition across sensory and cognitive contexts. The PCC is implicated in psychedelic neuroimaging [Carhart-Harris et al., 2012, Muthukumaraswamy et al., 2013, Smigielski et al., 2019] and this choice is in line with our prior work, including psychedelic and DCM/DMN studies [Preller et al., 2019, Stoliker et al., 2023, 2024b, Zhou et al., 2018]. Given causal evidence implicating the anterior precuneus in bodily-self processing [Lyu et al., 2023], we flag an expanded model variant including an explicit precuneus node as a future, hypothesis-driven extension for studies of the bodily sense of self under psychedelics.

4.8. *Why was a 3D latent space chosen for CEBRA embeddings? Was this empirically validated (e.g., via explained variance or classification performance)?*

Authors' Response:

CEBRA uses a distance metric—in our case, cosine distance—to learn a low-dimensional space in which patterns of brain activity that are similar over time are placed closer together. Cosine distance specifically captures the angle between two signals, reflecting how similar their shapes are, regardless of their overall magnitude.

For cosine distance to effectively capture meaningful relationships, the embedding space must provide sufficiently rich angular structure. Cosine distance compares how similar the directions of vectors are, not their absolute values. For this to work meaningfully:

• In 1D space, cosine distance becomes degenerate: there are only two directions (left or right), so all distinctions collapse to just "same or opposite direction."

• In 2D space, vectors lie on a circle, but the angular resolution is limited, and it's difficult to distinguish multiple complex patterns.

• 3D is the minimum meaningful space where vectors can spread out over a sphere, allowing richer angular relationships to emerge. This makes the embedding space both functional and interpretable.

Increasing the embedding dimensionality beyond 3 does not improve classification accuracy or visual interpretability in our downstream analyses. Thus, 3D offered the best balance between expressiveness and clarity, especially for visualization purposes (as higher dimensions cannot be directly visualized).

4.9. *Were multiple runs of CEBRA performed to assess the stability of embeddings?*

Authors' Response:

We thank the reviewer for this important point.

CEBRA is an established method published in *Nature*, where one of its core characteristics—*stability and robustness across repeated runs*—is explicitly demonstrated ([Schneider et al., 2023]; see Fig. 1b,e and Extended Data Figs. 2 and 4).

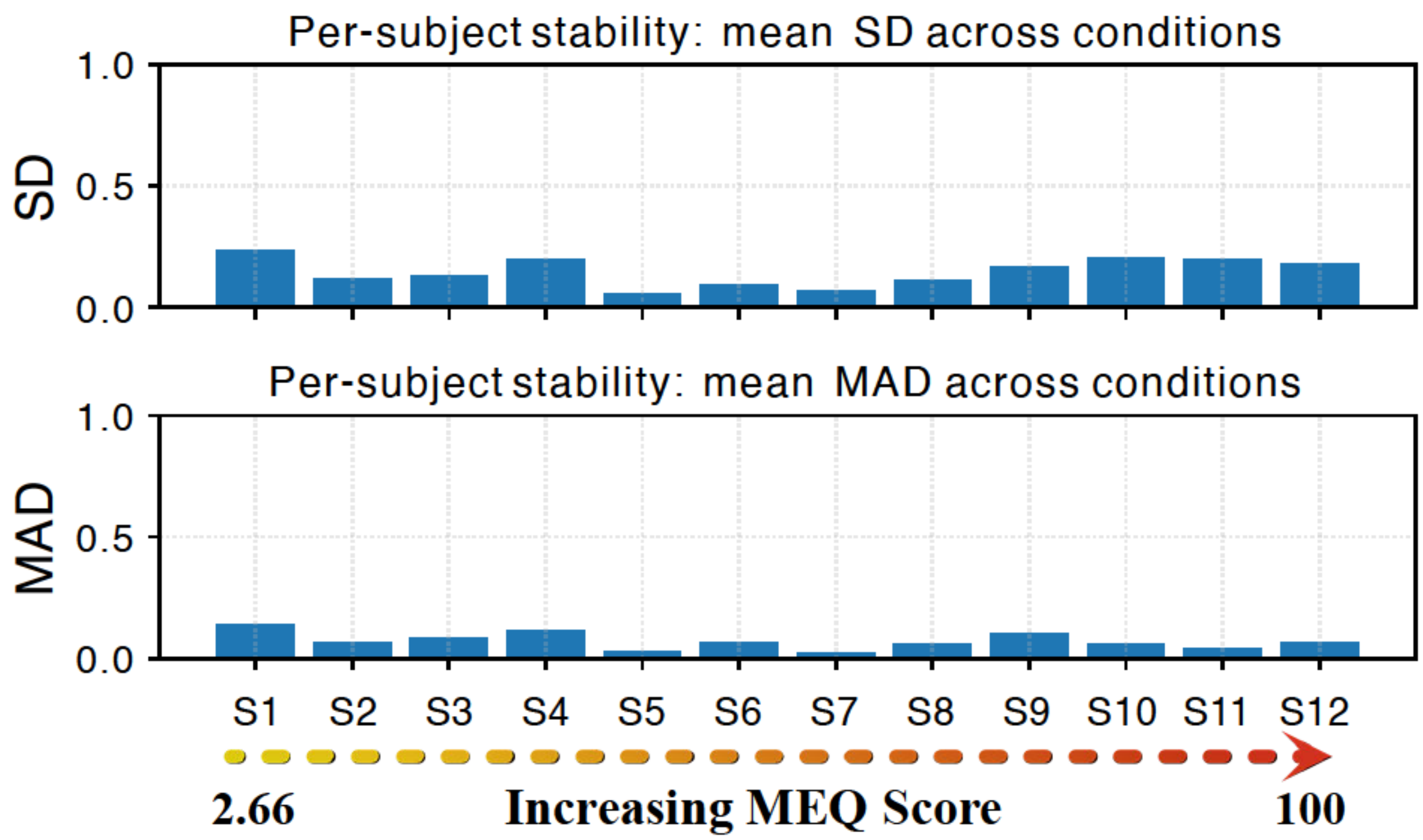

Figure R10: **Per-subject stability of CEBRA embeddings across repeated runs.** For each of the 12 subjects included in Fig. R4, CEBRA training was repeated 10 times per subject. Run-to-run variability of silhouette scores was quantified using (top) the **mean standard deviation (SD)** and (bottom) the **mean median absolute deviation (MAD)** across the four conditions. Both SD and MAD values were consistently small (SD: ∼0.05–0.25; MAD: ∼0.03–0.14), indicating that repeated runs of CEBRA yield nearly identical clustering quality. These results confirm that the observed group-level differences (e.g., between low and high MEQ scores in Fig. R4d) are far larger than run-to-run variability, demonstrating the robustness of our conclusions.

In addition to that and in line with this published guidance, we explicitly evaluated stability in our own dataset. For each of the 12 subjects shown in Fig. R4, we repeated CEBRA training 10 times per subject with different random seeds. We then quantified run-to-run variability of silhouette scores using two complementary measures: *Standard Deviation (SD)* and *Median Absolute Deviation (MAD)*.

As shown in Fig. R10, both SD and MAD values were consistently small across subjects. Mean SD values ranged from ∼0.05 to 0.25, and mean MAD values from ∼0.03 to 0.14, well within the $[-1, 1]$ range of silhouette scores. This demonstrates that repeated runs of CEBRA yield nearly identical clustering quality for each subject and condition.

Importantly, these small run-to-run variations are an order of magnitude smaller than the group-level effects reported in our silhouette score analysis (e.g., Low vs High MEQ differences under Psilocybin of ∼0.4–0.6, see Fig. R4d).

Together, these results confirm that our conclusions are robust to initialisation and fully consistent with the stability characteristics reported in the original *Nature* CEBRA paper. We have now added these new findings as Extended Data Fig. 7 to the revised manuscript.

4.10. *In the part of the manuscript the authors explain TAVRNN they state: "In this work, each scan under a specific condition (rest, meditation, music, or movie) is treated as a temporal snapshot. Therefore, the attention mechanism enables the lower-dimensional representation of one scan to inform the representation of the subsequent scans." Was there any segmentation of the timeseries for TAVRNN input e.g. overlapping windows, or condition-averaged matrices? I am not sure how the 2D embeddings of individual brain regions were interpreted? clustering to relate them to subjective or behavioural data?*

Authors' Response:

To clarify, for each subject, we computed one functional connectivity (FC) matrix per condition using the entire time series for that condition. This gave us a sequence of four FC matrices—one for each condition—which served as input to TAVRNN. There was no segmentation or overlapping windowing applied; we used full-condition data to capture stable, condition-specific brain network states.

TAVRNN learns 2D representations of the individual brain regions (ROIs) during each condition. In main-text Fig. 3d, each point represents one ROI positioned in the learned 2D space. These embeddings reveal how the spatial organisation of brain activity varies across conditions and between high- and low-MEQ groups.

The 2D points are ROI embeddings whose distances reflect similarity of connectivity fingerprints within a condition; they are intermediate features for the temporal model, not independent observations to be clustered. For this reason we did not perform ROI-level clustering or direct behavioral correlations on the 2-D points, which would conflate within-subject dependence and discard temporal structure. Instead, we assess condition separability and group differences (e.g., high- vs low-MEQ) at the representation level best aligned with the model's design. We observed that clear spatial patterns emerged: the high-MEQ group showed more structured and distinct patterns compared to the low-MEQ group, suggesting psilocybin enhances the brain's ability to reorganise network-level activity in response to experiential context. We have clarified these points in the revised manuscript to avoid any confusion:

"These 2D maps represent abstract latent dimensions learned from brain activity patterns, not physical brain coordinates, with each point corresponding to one ROI placed closer to others when their activity evolves similarly over time."

and

"No segmentation or overlapping windows were applied; each FC was computed using the full time series for that condition to capture stable, condition-specific network states."

4.11. *Am I mistaken in thinking that the TAVRNN framework is best implemented with EEG data because of its superior temporal resolution?*

Authors' Response:

Thank you for raising this point. EEG's high temporal resolution is valuable, but in our implementation TAVRNN does not operate at millisecond scales. We compute one functional connectivity (FC) matrix per condition block and input the time-ordered sequence of these FCs to TAVRNN, which learns transitions between condition-level network states rather than rapid moment-to-moment fluc-

tuations. This design suits fMRI because it provides whole-brain (including subcortical) coverage and stable block-level FC estimates with reliable spatial mapping of macroscale networks. By contrast, sensor-space EEG connectivity is confounded by electrical activity from a single source spreading to multiple electrodes and source ambiguity. Even after source reconstruction, spatial precision and depth sensitivity are typically lower, making whole-brain block-level FC estimates from fMRI more robust in this context.

4.12. *Page 12: "Our decision to administer a standardised dose of 19 mg psilocybin, rather than a body-weight-adjusted dose was based on previous research showing limited benefits of body-weight-adjusted dosing (Garcia-Romeu et al., 2021). " What does limited benefits mean? Was it in terms of efficacy, safety, variability, or practicality?*

Authors' Response:

We appreciate the reviewer's request for clarification. The phrase "limited benefits" refers to findings from Garcia-Romeu et al. (2021), based on pooled data from 288 adults across ten Johns Hopkins studies (2001–2018), which included both weight-adjusted dosing (20 or 30 mg/70 kg) and a fixed-dose subgroup. Regression analyses found no evidence that weight-adjusted dosing improved efficacy, as measured by subjective effect intensity. Although the study did not directly assess inter-individual variability or safety by dose strategy, no consistent patterns of divergence were noted across a body weight range of 49–113 kg. In contrast, fixed dosing yielded comparable outcomes and offers greater practical and logistical simplicity. On this basis, we considered a fixed 19 mg dose to be both scientifically justified and operationally efficient.

We have updated the text as follows:

"Our decision to administer a standardised dose of 19 mg psilocybin, rather than a body-weight-adjusted dose was based on previous research showing no clear advantage of weight-adjusted dosing in terms of subjective effect intensity or predictable differences in response across individuals of varying body weight [Garcia-Romeu et al., 2021]. "

4.12. *Fig. Extended Data 1 shows increases and decreases but not clear under what conditions.*

Authors' Response:

The four rows of the figure correspond to the four conditions (rest, meditation, music, movie), and we have added the same icons as in the other figures in the main manuscript to make this more intuitive. The updated figure is also presented here for convenience (Fig. R11).

Concluding remarks Thank you for your constructive suggestions. They helped improve the transparency, stability, and biological interpretability of our machine learning methods, and strengthened both the clarity and robustness of the overall analytic framework.

References

H. Abdi and L. J. Williams. Principal component analysis. *Wiley interdisciplinary reviews: computational statistics*, 2(4):433–459, 2010.

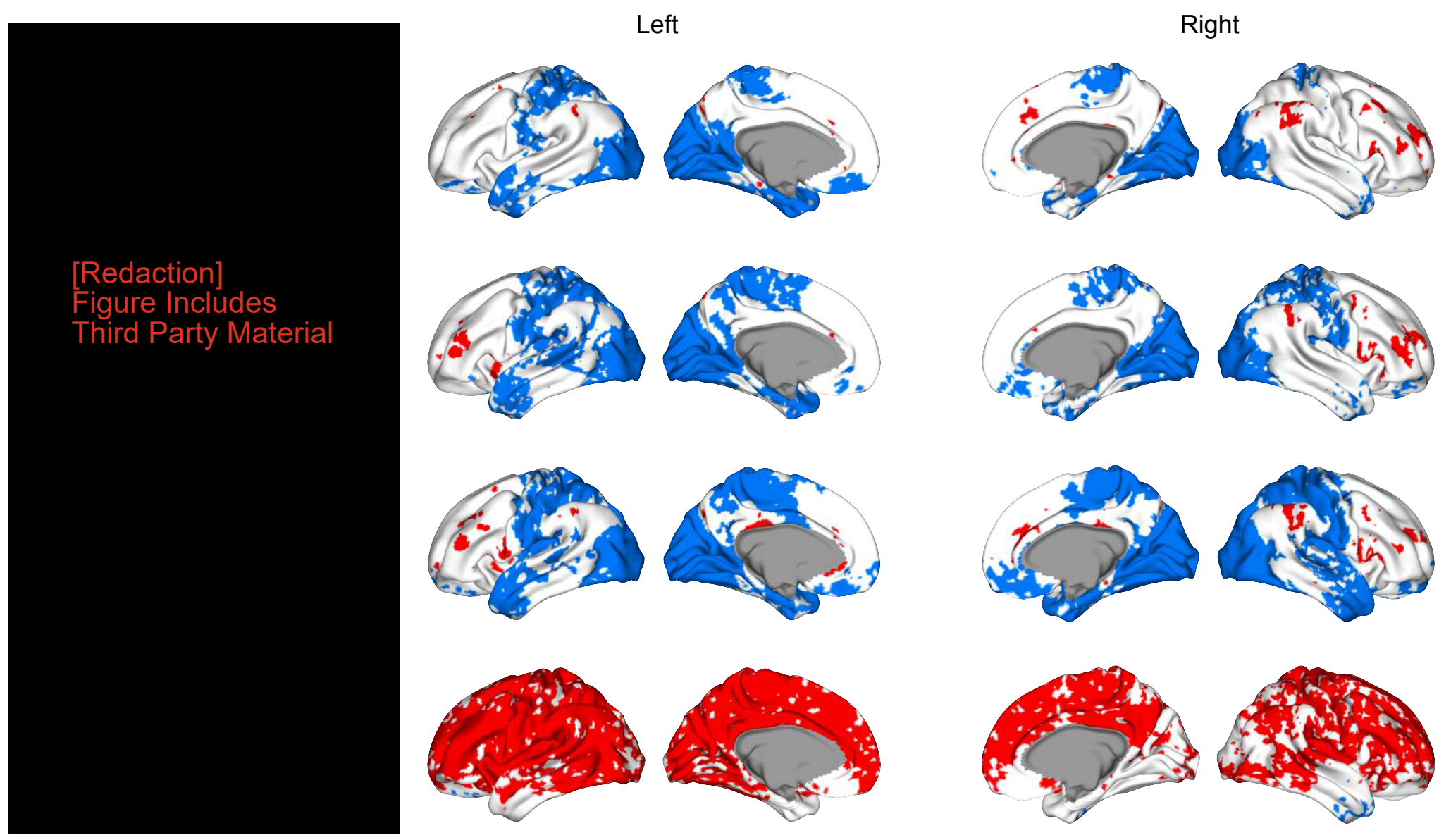

Figure R11: Clusters of statistically significant global functional connectivity increase (red) or decrease (blue) at $p < 0.05$ after threshold-free cluster enhancement.

G. K. Aguirre. Continuous carry-over designs for fmri. *NeuroImage*, 35(4):1480–1494, 2007. doi: 10.1016/j.neuroimage.2007.02.005.

J. R. Andrews-Hanna, J. S. Reidler, J. Sepulcre, R. Poulin, and R. L. Buckner. Functional-anatomic fractionation of the brain's default network. *Neuron*, 65(4):550–562, 2010. ISSN 1097-4199 0896-6273. doi: 10.1016/j.neuron.2010.02.005.

P. Avram et al. Large-scale brain connectivity changes following the administration of lysergic acid diethylamide, d-amphetamine and 3,4-methylenedioxyamphetamine. *Molecular Psychiatry*, 30: 1297–1307, 2025. doi: 10.1038/s41380-024-02734-y.

M. P. Bogenschutz, S. Ross, S. Bhatt, T. Baron, A. A. Forcehimes, E. Laska, S. E. Mennenga, K. O'Donnell, L. T. Owens, S. Podrebarac, J. Rotrosen, J. S. Tonigan, and L. Worth. Percentage of heavy drinking days following psilocybin-assisted psychotherapy vs placebo in the treatment of adult patients with alcohol use disorder: A randomized clinical trial. *JAMA Psychiatry*, 79(10): 953–962, 2022. doi: 10.1001/jamapsychiatry.2022.2096.

G. M. Boynton, S. A. Engel, G. H. Glover, and D. J. Heeger. Linear systems analysis of functional magnetic resonance imaging in human v1. *Journal of Neuroscience*, 16(13):4207–4221, 1996.

R. T. Brown, C. R. Nicholas, N. V. Cozzi, M. C. Gassman, K. M. Cooper, D. Muller, C. D. Thomas, S. Hetzel, K. M. Henriquez, A. S. Ribaudo, and P. R. Hutson. Pharmacokinetics of escalating doses of oral psilocybin in healthy adults. *Clinical Pharmacokinetics*, 56(12):1543–1554, 2017. doi: 10.1007/s40262-017-0540-6.

J. B. Burt, K. H. Preller, M. Demirtas, J. L. Ji, J. H. Krystal, F. X. Vollenweider, A. Anticevic, and J. D. Murray. Transcriptomics-informed large-scale cortical model captures topography of pharmacological neuroimaging effects of LSD. *eLife*, 10:e69320, 2021. ISSN 2050-084X. doi: 10.7554/eLife.69320.

M. Butler, L. A. Jelen, and J. J. Rucker. Expectancy in placebo-controlled trials of psychedelics: if so, so what? *Psychopharmacology (Berl.)*, 239:3047–3055, 2022. doi: 10.1007/s00213-022-06221-6.

T. M. Carbonaro, M. W. Johnson, and R. R. Griffiths. Subjective features of the psilocybin experience that may account for its self-administration by humans: A double-blind comparison of psilocybin and dextromethorphan. *Psychopharmacology (Berl)*, 237(8):2293–2304, 2020. ISSN 0033-3158. doi: 10.1007/s00213-020-05533-9.

R. L. Carhart-Harris, D. Erritzoe, T. Williams, J. M. Stone, L. J. Reed, A. Colasanti, R. J. Tyacke, R. Leech, A. L. Malizia, K. Murphy, P. Hobden, J. Evans, A. Feilding, R. G. Wise, and D. J. Nutt. Neural correlates of the psychedelic state as determined by fMRI studies with psilocybin. *Proceedings of the National Academy of Sciences*, 109(6):2138–2143, Feb. 2012. doi: 10.1073/pnas.1119598109.

H. W. Chung, B. M. Sadler, and A. O. Hero. Bounds on Variance for Unimodal Distributions. *IEEE Transactions on Information Theory*, 63(11):6936–6949, 2017. ISSN 1557-9654. doi: 10.1109/TIT.2017.2749310.

J. P. Cunningham and B. M. Yu. Dimensionality reduction for large-scale neural recordings. *Nat Neurosci*, 17(11):1500–9, Nov. 2014. ISSN 1097-6256 (Print) 1097-6256. doi: 10.1038/nn.3776.

R. E. Daws, C. Timmermann, B. Giribaldi, J. D. Sexton, M. B. Wall, D. Erritzoe, L. Roseman, D. Nutt, and R. L. Carhart-Harris. Increased global integration in the brain after psilocybin therapy for depression. *Nature Medicine*, 28(4):844–851, 2022. doi: 10.1038/s41591-022-01744-z. URL https://www.nature.com/articles/s41591-022-01744-z.

E. DuPre, T. Salo, Z. Ahmed, P. A. Bandettini, K. L. Bottenhorn, C. Caballero-Gaudes, L. T. Dowdle, J. Gonzalez-Castillo, S. Heunis, P. Kundu, A. R. Laird, R. Markello, C. J. Markiewicz, S. Moia, I. Staden, J. B. Teves, E. Uruñuela, M. Vaziri-Pashkam, K. Whitaker, and D. A. Handwerker. TE-dependent analysis of multi-echo fMRI with tedana. *Journal of Open Source Software*, 6(66):3669, Oct. 2021. ISSN 2475-9066. doi: 10.21105/joss.03669.

K. Friston, L. Harrison, and W. Penny. Dynamic causal modelling. *NeuroImage*, 19(4):1273–1302, Aug. 2003. ISSN 10538119. doi: 10.1016/S1053-8119(03)00202-7.

K. J. Friston, J. Kahan, B. Biswal, and A. Razi. A DCM for resting state fMRI. *NeuroImage*, 94: 396–407, July 2014. ISSN 10538119. doi: 10.1016/j.neuroimage.2013.12.009.

K. J. Friston, V. Litvak, A. Oswal, A. Razi, K. E. Stephan, B. C. M. van Wijk, G. Ziegler, and P. Zeidman. Bayesian model reduction and empirical Bayes for group (DCM) studies. *NeuroImage*, 128:413–431, Mar. 2016. ISSN 1053-8119. doi: 10.1016/j.neuroimage.2015.11.015.

A. Garcia-Romeu, F. S. Barrett, T. M. Carbonaro, M. W. Johnson, and R. R. Griffiths. Optimal dosing for psilocybin pharmacotherapy: Considering weight-adjusted and fixed dosing approaches. *Journal of Psychopharmacology*, 35(4):353–361, 2021. doi: 10.1177/0269881121991822.

P. Gasser, D. Holstein, Y. Michel, R. Doblin, B. Yazar-Klosinski, T. Passie, and R. Brenneisen. Safety and efficacy of lysergic acid diethylamide-assisted psychotherapy for anxiety associated with life-threatening diseases. *The Journal of Nervous and Mental Disease*, 202(7):513–520, 2014. doi: 10.1097/NMD.0000000000000113.

G. H. Glover. Deconvolution of impulse response in event-related bold fmri. *NeuroImage*, 9(4): 416–429, 1999. doi: 10.1006/nimg.1998.0419.

R. R. Griffiths, M. W. Johnson, W. A. Richards, B. D. Richards, U. McCann, and R. Jesse. Psilocybin occasioned mystical-type experiences: Immediate and persisting dose-related effects. *Psychopharmacology*, 218(4):649–665, 2011. ISSN 0033-3158. doi: 10.1007/s00213-011-2358-5.

R. R. Griffiths, M. W. Johnson, M. A. Carducci, A. Umbricht, W. A. Richards, B. D. Richards, M. P. Cosimano, and M. A. Klinedinst. Psilocybin produces substantial and sustained decreases in depression and anxiety in patients with life-threatening cancer: A randomized double-blind trial. *Journal of Psychopharmacology*, 30(12):1181–1197, 2016. ISSN 0269-8811. doi: 10.1177/0269881116675513.

F. Hasler, U. Grimberg, M. A. Benz, T. Huber, and F. X. Vollenweider. Acute psychological and physiological effects of psilocybin in healthy humans: a double-blind, placebo-controlled dose–effect study. *Psychopharmacology*, 172(2):145–156, 2004. doi: 10.1007/s00213-003-1640-6.

R. Herzog, P. A. M. Mediano, F. E. Rosas, P. Lodder, R. Carhart-Harris, Y. S. Perl, E. Tagliazucchi, and R. Cofre. A whole-brain model of the neural entropy increase elicited by psychedelic drugs. *Sci Rep*, 13(1):6244, Apr. 2023. ISSN 2045-2322. doi: 10.1038/s41598-023-32649-7.

T. Hirschfeld and T. T. Schmidt. Dose–response relationships of psilocybin-induced subjective experiences in humans. *Journal of Psychopharmacology*, 35(4):384–397, 2021. ISSN 0269-8811. doi: 10.1177/0269881121992676.

T. Hirschfeld, J. Prugger, T. Majić, and T. T. Schmidt. Dose-response relationships of LSD-induced subjective experiences in humans. *Neuropsychopharmacology*, 48(11):1602–1611, Oct. 2023. ISSN 0893-133X (Print) 0893-133x. doi: 10.1038/s41386-023-01588-2.

F. Holze, A. M. Becker, K. E. Kolaczynska, U. Duthaler, and M. E. Liechti. Pharmacokinetics and pharmacodynamics of oral psilocybin administration in healthy participants. *Clin Pharmacol Ther*, 2022. ISSN 0009-9236. doi: 10.1002/cpt.2821.

F. Holze, P. Gasser, F. Müller, P. C. Dolder, and M. E. Liechti. Lysergic acid diethylamide–assisted therapy in patients with anxiety with and without a life-threatening illness: A randomized, double-blind, placebo-controlled phase ii study. *Biological Psychiatry*, 93(3):215–223, 2023. doi: 10.1016/j.biopsych.2022.08.025.

M. Khajehnejad, F. Habibollahi, A. Khajehnejad, B. J. Kagan, and A. Razi. TAVRNN: Temporal Attention-enhanced Variational Graph RNN Captures Neural Dynamics and Behavior, Oct. 2024.

P. Kundu, S. J. Inati, J. W. Evans, W.-M. Luh, and P. A. Bandettini. Differentiating BOLD and non-BOLD signals in fMRI time series using multi-echo EPI. *NeuroImage*, 60(3):1759–1770, Apr. 2012. ISSN 1053-8119. doi: 10.1016/j.neuroimage.2011.12.028.

A. C. Kwan, D. E. Olson, K. H. Preller, and B. L. Roth. The neural basis of psychedelic action. *Nat Neurosci*, 25(11):1407–1419, Nov. 2022. ISSN 1546-1726. doi: 10.1038/s41593-022-01177-4.

A. Lebedev, M. Kaelen, M. Lövdén, J. Nilsson, A. Feilding, D. Nutt, and R. Carhart-Harris. LSD-induced entropic brain activity predicts subsequent personality change. *Human Brain Mapping*, 37(9):3203–3213, 2016. ISSN 1097-0193. doi: 10.1002/hbm.23234.

R. Leech and D. J. Sharp. The role of the posterior cingulate cortex in cognition and disease. *Brain*, 137(Pt 1):12–32, 2014. ISSN 1460-2156 0006-8950. doi: 10.1093/brain/awt162.

L. Ley, F. Holze, D. Arikci, A. M. Becker, I. Straumann, A. Klaiber, F. Coviello, S. Dierbach, J. Thomann, U. Duthaler, D. Luethi, N. Varghese, A. Eckert, and M. E. Liechti. Comparative acute effects of mescaline, lysergic acid diethylamide, and psilocybin in a randomized, double-blind, placebo-controlled cross-over study in healthy participants. *Neuropsychopharmacology*, 48(11):1659–1667, Oct. 2023. doi: 10.1038/s41386-023-01607-2.

C. Ly, A. C. Greb, L. P. Cameron, J. M. Wong, E. V. Barragan, P. C. Wilson, K. F. Burbach, S. Soltanzadeh Zarandi, A. Sood, M. R. Paddy, W. C. Duim, M. Y. Dennis, A. K. McAllister, K. M. Ori-McKenney, J. A. Gray, and D. E. Olson. Psychedelics Promote Structural and Functional Neural Plasticity. *Cell Reports*, 23(11):3170–3182, June 2018. ISSN 2211-1247. doi: 10.1016/j.celrep.2018.05.022.

D. Lyu, J. R. Stieger, C. Xin, E. Ma, Z. Lusk, M. K. Aparicio, K. Werbaneth, C. M. Perry, K. Deisseroth, V. Buch, and J. Parvizi. Causal evidence for the processing of bodily self in the anterior precuneus. *Neuron*, 111(1):76–89, 2023. doi: 10.1016/j.neuron.2023.05.013.

L. v. d. Maaten and G. Hinton. Visualizing data using t-sne. *Journal of machine learning research*, 9 (Nov):2579–2605, 2008.

M. K. Madsen and G. M. Knudsen. Plasma psilocin critically determines behavioral and neurobiological effects of psilocybin. *Neuropsychopharmacology*, 46(1):257–258, 2021. ISSN 1740-634X 0893-133X. doi: 10.1038/s41386-020-00823-4.

M. K. Madsen, D. Burmester, D. S. Stenbaek, S. Kristiansen, A. Dyssegaard, S. Lehel, K. Linnet, S. S. Johansen, C. Svarer, B. Ozenne, D. Erritzoe, P. M. Fisher, and G. M. Knudsen. Psilocybin occupancy of brain serotonin 2A receptors correlates with psilocin levels and subjective experience: A 11C Cimbi-36 PET study in humans. *European Neuropsychopharmacology*, 29:S304–S305, 2019. ISSN 0924-977X. doi: 10.1016/j.euroneuro.2018.11.474.

M. K. Madsen, D. S. Stenbæk, A. Arvidsson, S. Armand, M. R. Marstrand-Joergensen, S. S. Johansen, K. Linnet, B. Ozenne, G. M. Knudsen, and P. M. Fisher. Psilocybin-induced changes in brain network integrity and segregation correlate with plasma psilocin level and psychedelic experience. *European Neuropsychopharmacology*, 50:121–132, 2021. ISSN 0924-977X. doi: 10.1016/j.euroneuro.2021.06.001.

C. M. Michel and T. Koenig. Eeg microstates as a tool for exploring the temporal dynamics of whole-brain neuronal networks: A review. *NeuroImage*, 180:577–593, 2018. doi: 10.1016/j.neuroimage.2017.11.062. Sub-second quasi-stable EEG states, rapid transitions.

F. Müller, C. Lenz, P. Dolder, U. Lang, A. Schmidt, M. Liechti, and S. Borgwardt. Increased thalamic resting-state connectivity as a core driver of LSD-induced hallucinations. *Acta Psychiatrica Scandinavica*, 2017.

S. D. Muthukumaraswamy, R. L. Carhart-Harris, R. J. Moran, M. J. Brookes, T. M. Williams, D. Errtizoe, B. Sessa, A. Papadopoulos, M. Bolstridge, K. D. Singh, A. Feilding, K. J. Friston, and D. J. Nutt. Broadband Cortical Desynchronization Underlies the Human Psychedelic State. *The Journal of Neuroscience*, 33(38):15171–15183, 2013. doi: 10.1523/jneurosci.2063-13.2013.

S. D. Muthukumaraswamy, A. Forsyth, and T. Lumley. Blinding and expectancy confounds in psychedelic randomized controlled trials. *Expert Review of Clinical Pharmacology*, 14(9):1133–1152, 2021. ISSN 1751-2433. doi: 10.1080/17512433.2021.1933434.

L. Novelli, D. Stoliker, T. Barta, M. D. Greaves, S. Chopra, J. Jackson, J. Kwee, M. L. Williams, and A. Razi. PsiConnect: A Multimodal Neuroimaging Study of Psilocybin-Induced Changes in Brain and Behaviour. page 2025.04.11.643415, May 2025. doi: 10.1101/2025.04.11.643415.

A. S. Olsen, A. Lykkebo-Valløe, B. Ozenne, M. K. Madsen, D. S. Stenbæk, S. Armand, M. Mørup, M. Ganz, G. M. Knudsen, and P. M. Fisher. Psilocybin modulation of time-varying functional connectivity is associated with plasma psilocin and subjective effects. *NeuroImage*, 264:119716, 2022. doi: 10.1016/j.neuroimage.2022.119716.

M. E. Otto, R. A. Slivicki, E. A. da Silva, N. Topping, A. Peace, R. Cairns, L. Colon-Perez, B. Setlow, R. Vohra, A. Bruijnzeel, and A. W. Bruijnzeel. Clinical pharmacokinetics of psilocin after psilocybin administration: A systematic review and post-hoc analysis. *Clinical Pharmacokinetics*, 2025. doi: 10.1007/s40262-024-01412-6. Epub ahead of print.

L. Parkes, B. Fulcher, M. Yücel, and A. Fornito. An evaluation of the efficacy, reliability, and sensitivity of motion correction strategies for resting-state functional MRI. *NeuroImage*, 171:415–436, May 2018. ISSN 10959572. doi: 10.1016/j.neuroimage.2017.12.073.

Y. S. Perl, S. Geli, E. Pérez-Ordoyo, L. Zonca, S. Idesis, J. Vohryzek, V. K. Jirsa, M. L. Kringelbach, E. Tagliazucchi, and G. Deco. Modelling low-dimensional interacting brain networks reveals organising principle in human cognition. *Network Neuroscience*, pages 1–21, Jan. 2025. ISSN 2472-1751. doi: 10.1162/netn_a_00434.

A. S. Pillai and V. K. Jirsa. Symmetry Breaking in Space-Time Hierarchies Shapes Brain Dynamics and Behavior. *Neuron*, 94(5):1010–1026, June 2017. ISSN 0896-6273. doi: 10.1016/j.neuron.2017.05.013.

K. H. Preller, J. B. Burt, J. L. Ji, C. H. Schleifer, B. D. Adkinson, P. Stämpfli, E. Seifritz, G. Repovs, J. H. Krystal, J. D. Murray, F. X. Vollenweider, and A. Anticevic. Changes in global and thalamic brain connectivity in LSD-induced altered states of consciousness are attributable to the 5-HT2A receptor. *eLife*, 7:e35082, Oct. 2018. ISSN 2050-084X. doi: 10.7554/eLife.35082.

K. H. Preller, A. Razi, P. Zeidman, P. Stämpfli, K. J. Friston, and F. X. Vollenweider. Effective connectivity changes in LSD-induced altered states of consciousness in humans. *Proceedings of the National Academy of Sciences of the United States of America*, 116(7):2743–2748, Feb. 2019. ISSN 10916490. doi: 10.1073/pnas.1815129116.

K. H. Preller, P. Duerler, J. B. Burt, J. L. Ji, B. Adkinson, P. Stämpfli, E. Seifritz, G. Repovš, J. H. Krystal, J. D. Murray, A. Anticevic, and F. X. Vollenweider. Psilocybin Induces Time-Dependent Changes in Global Functional Connectivity. *Biological Psychiatry*, 88(2):197–207, July 2020. ISSN 0006-3223. doi: 10.1016/j.biopsych.2019.12.027.

N. R. Raval, A. Johansen, L. L. Donovan, N. F. Ros, B. Ozenne, H. D. Hansen, and G. M. Knudsen. A Single Dose of Psilocybin Increases Synaptic Density and Decreases 5-HT(2A) Receptor Density in the Pig Brain. *International journal of molecular sciences*, 22(2), 2021. ISSN 1422-0067. doi: 10.3390/ijms22020835.

A. Razi and K. J. Friston. The Connected Brain: Causality, models, and intrinsic dynamics. *IEEE Signal Processing Magazine*, 33(3):14–35, May 2016. ISSN 1053-5888. doi: 10.1109/MSP.2015.2482121.

J. R. Reinwald, C. N. Schmitz, I. Skorodumov, M. Kuchar, W. Weber-Fahr, R. Spanagel, and M. W. Meinhardt. Psilocybin-induced default mode network hypoconnectivity is blunted in alcohol-dependent rats. *Translational psychiatry*, 13(1):392, Dec. 2023. ISSN 2158-3188. doi: 10.1038/s41398-023-02690-1.

S. Ross, A. Bossis, J. Guss, G. Agin-Liebes, T. Malone, B. Cohen, S. E. Mennenga, A. Belser, K. Kalliontzi, J. Babb, Z. Su, P. Corby, and B. L. Schmidt. Rapid and sustained symptom reduction following psilocybin treatment for anxiety and depression in patients with life-threatening cancer: A randomized controlled trial. *Journal of Psychopharmacology*, 30(12):1165–1180, 2016. ISSN 0269-8811. doi: 10.1177/0269881116675512.

M. M. Schartner, R. L. Carhart-Harris, A. B. Barrett, A. K. Seth, and S. D. Muthukumaraswamy. Increased spontaneous MEG signal diversity for psychoactive doses of ketamine, LSD and psilocybin. *Sci Rep*, 7(1):46421, Apr. 2017. ISSN 2045-2322. doi: 10.1038/srep46421.

M. Schmidt, A. Hoffrichter, M. Davoudi, S. Horschitz, T. Lau, M. Meinhardt, R. Spanagel, J. Ladewig, G. Köhr, and P. Koch. *Psilocin Fosters Neuroplasticity in iPSC-derived Human Cortical Neurons*. 2024.

S. Schneider, J. H. Lee, and M. W. Mathis. Learnable latent embeddings for joint behavioural and neural analysis. *Nature*, 617(7960):360–368, May 2023. ISSN 1476-4687. doi: 10.1038/s41586-023-06031-6.

M. L. Seghier and K. J. Friston. Network discovery with large DCMs. *NeuroImage*, 68:181–191, Mar. 2013. ISSN 10538119. doi: 10.1016/j.neuroimage.2012.12.005.

L. Shao, C. Liao, P. A. Davoudian, N. K. Savalia, Q. Jiang, C. Wojtasiewicz, D. Tan, J. D. Nothnagel, R. Liu, S. C. Woodburn, O. M. Bilash, H. B. Kim, A. Che, and A. C. Kwan. Psilocybin's lasting action requires pyramidal cell types and 5-HT$_{2A}$ receptors. *Nature*, 2025a. doi: 10.1038/s41586-025-08813-6.

L. X. Shao, C. Liao, I. Gregg, P. A. Davoudian, N. K. Savalia, K. Delagarza, and A. C. Kwan. Psilocybin induces rapid and persistent growth of dendritic spines in frontal cortex in vivo. *Neuron*, 109(16):2535–2544.e4, 2021. ISSN 0896-6273 (Print) 0896-6273. doi: 10.1016/j.neuron.2021.06.008.

L.-X. Shao, C. Liao, P. A. Davoudian, N. K. Savalia, Q. Jiang, C. Wojtasiewicz, D. Tan, J. D. Nothnagel, R.-J. Liu, S. C. Woodburn, O. M. Bilash, H. Kim, A. Che, and A. C. Kwan. Psilocybin's lasting action requires pyramidal cell types and 5-ht2a receptors. *Nature*, 642:411–420, Apr. 2025b. doi: 10.1038/s41586-025-08813-6. Published online 2025-04-02.

J. S. Siegel, S. Subramanian, D. Perry, B. P. Kay, E. M. Gordon, T. O. Laumann, T. R. Reneau, N. V. Metcalf, R. V. Chacko, C. Gratton, C. Horan, S. R. Krimmel, J. S. Shimony, J. A. Schweiger, D. F. Wong, D. A. Bender, K. M. Scheidter, F. I. Whiting, J. A. Padawer-Curry, R. T. Shinohara, Y. Chen, J. Moser, E. Yacoub, S. M. Nelson, L. Vizioli, D. A. Fair, E. J. Lenze, R. Carhart-Harris, C. L. Raison, M. E. Raichle, A. Z. Snyder, G. E. Nicol, and N. U. F. Dosenbach. Psilocybin desynchronizes the human brain. *Nature*, 632(8023):131–138, Aug. 2024. ISSN 1476-4687. doi: 10.1038/s41586-024-07624-5.

B. Singer, D. Meling, M. Hirsch-Hoffmann, L. Michels, M. Kometer, L. Smigielski, D. Dornbierer, E. Seifritz, F. Vollenweider, and M. Scheidegger. Psilocybin enhances insightfulness in meditation: A perspective on the global topology of brain imaging during meditation. *Scientific Reports*, 14, 2024. doi: 10.1038/s41598-024-55726-x.

S. P. Singleton, A. I. Luppi, R. L. Carhart-Harris, J. Cruzat, L. Roseman, D. J. Nutt, G. Deco, M. L. Kringelbach, E. A. Stamatakis, and A. Kuceyeski. Receptor-informed network control theory links LSD and psilocybin to a flattening of the brain's control energy landscape. *Nat Commun*, 13(1): 5812, Oct. 2022. ISSN 2041-1723. doi: 10.1038/s41467-022-33578-1.

L. Smigielski, M. Scheidegger, M. Kometer, and F. X. Vollenweider. Psilocybin-assisted mindfulness training modulates self-consciousness and brain default mode network connectivity with lasting effects. *NeuroImage*, 196:207–215, 2019. ISSN 1095-9572. doi: 10.1016/j.neuroimage.2019.04.009.

D. Stoliker, L. Novelli, F. X. Vollenweider, G. F. Egan, K. H. Preller, and A. Razi. Effective Connectivity of Functionally Anticorrelated Networks Under Lysergic Acid Diethylamide. *Biological Psychiatry*, 93:224–232, 2023. ISSN 00063223. doi: 10.1016/j.biopsych.2022.07.013.

D. Stoliker, L. Novelli, F. X. Vollenweider, G. F. Egan, K. H. Preller, and A. Razi. Neural Mechanisms of Resting-State Networks and the Amygdala Underlying the Cognitive and Emotional Effects of Psilocybin. *Biological Psychiatry*, 96(1):57–66, 2024b. ISSN 0006-3223. doi: 10.1016/j.biopsych.2024.01.002.

E. Studerus, M. Kometer, F. Hasler, and F. X. Vollenweider. Acute, subacute and long-term subjective effects of psilocybin in healthy humans: A pooled analysis of experimental studies. *Journal of Psychopharmacology*, 25(11):1434–1452, 2011. ISSN 0269-8811. doi: 10.1177/0269881110382466.

B. Szigeti and B. D. Heifets. Expectancy effects in psychedelic trials. *Biological Psychiatry: Cognitive Neuroscience and Neuroimaging*, 9(5):512–521, May 2024. doi: 10.1016/j.bpsc.2024.02.004. Review article.

B. Szigeti, D. Carboni, A. K. Rengaswamy, M. I. Ettlinger, D. Erritzoe, R. L. Carhart-Harris, and B. D. Heifets. The difference between 'placebo group' and 'placebo control': a case study in psychedelic microdosing. *Scientific Reports*, 13(1):12107, 2023. doi: 10.1038/s41598-023-34938-7.

B. Szigeti et al. Placebo effects in psychedelic clinical trials: Evidence from a post hoc reanalysis of expectancy effects in a randomized controlled trial. *Psychological Medicine*, 2024. doi: 10.1017/S0033291724000214. In press.

E. Tagliazucchi, L. Roseman, M. Kaelen, C. Orban, S. D. Muthukumaraswamy, K. Murphy, H. Laufs, R. Leech, J. McGonigle, N. Crossley, E. Bullmore, T. Williams, M. Bolstridge, A. Feilding, D. J. Nutt, and R. Carhart-Harris. Increased Global Functional Connectivity Correlates with LSD-Induced Ego Dissolution. *Current Biology*, 26(8):1043–1050, Apr. 2016. ISSN 0960-9822. doi: 10.1016/j.cub.2016.02.010.

J. B. Tenenbaum, V. d. Silva, and J. C. Langford. A global geometric framework for nonlinear dimensionality reduction. *science*, 290(5500):2319–2323, 2000.

B. T. Thomas Yeo, F. M. Krienen, J. Sepulcre, M. R. Sabuncu, D. Lashkari, M. Hollinshead, J. L. Roffman, J. W. Smoller, L. Zöllei, J. R. Polimeni, B. Fisch, H. Liu, and R. L. Buckner. The organization of the human cerebral cortex estimated by intrinsic functional connectivity. *Journal of Neurophysiology*, 106(3):1125–1165, Sept. 2011. ISSN 00223077. doi: 10.1152/jn.00338.2011.

C. Timmermann, L. Roseman, M. Schartner, R. Milliere, L. T. J. Williams, D. Erritzoe, S. Muthukumaraswamy, M. Ashton, A. Bendrioua, O. Kaur, S. Turton, M. M. Nour, C. M. Day, R. Leech, D. J. Nutt, and R. L. Carhart-Harris. Neural correlates of the DMT experience assessed with multivariate EEG. *Sci Rep*, 9(1):16324, Nov. 2019. ISSN 2045-2322. doi: 10.1038/s41598-019-51974-4.

C. Timmermann, L. Roseman, S. Haridas, F. E. Rosas, L. Luan, H. Kettner, J. Martell, D. Erritzoe, E. Tagliazucchi, C. Pallavicini, M. Girn, A. Alamia, R. Leech, D. J. Nutt, and R. L. Carhart-Harris. Human brain effects of DMT assessed via EEG-fMRI. *Proc. Natl. Acad. Sci. U.S.A.*, 120(13): e2218949120, Mar. 2023. ISSN 0027-8424, 1091-6490. doi: 10.1073/pnas.2218949120.

L. Q. Uddin, A. M. Kelly, B. B. Biswal, F. X. Castellanos, and M. P. Milham. Functional connectivity of default mode network components: Correlation, anticorrelation, and causality. *Hum Brain Mapp*, 30(2):625–37, 2009. ISSN 1065-9471 (Print) 1065-9471. doi: 10.1002/hbm.20531.

A. V. Utevsky, D. V. Smith, and S. A. Huettel. Precuneus is a functional core of the default-mode network. *Journal of Neuroscience*, 34(3):932–940, 2014. doi: 10.1523/JNEUROSCI.4227-13.2014.

M. Wittmann, O. Carter, F. Hasler, B. R. Cahn, U. Grimberg, P. Spring, D. Hell, H. Flohr, and F. X. Vollenweider. Effects of psilocybin on time perception and temporal control of behaviour in humans. *Journal of Psychopharmacology*, 21(1):50–64, 2007. ISSN 0269-8811. doi: 10.1177/0269881106065859.

P. Zeidman, A. Jafarian, N. Corbin, M. L. Seghier, A. Razi, C. J. Price, and K. J. Friston. A guide to group effective connectivity analysis, part 1: First level analysis with DCM for fMRI. *NeuroImage*, 200:174–190, Oct. 2019. ISSN 10959572. doi: 10.1016/j.neuroimage.2019.06.031.

Y. Zhou, K. J. Friston, P. Zeidman, J. Chen, S. Li, and A. Razi. The Hierarchical Organization of the Default, Dorsal Attention and Salience Networks in Adolescents and Young Adults. *Cerebral Cortex*, 28(2):726–737, Feb. 2018. ISSN 14602199. doi: 10.1093/cercor/bhx307.

Psychedelics Align Brain Activity with Context

Response to Reviewers for MS-ID: 2025-04-08374

Referee 1

I think the finding that psilocybin greatly reduces the usual separation between eyes-open and eyes-closed fMRI and EEG measures is quite interesting.

Overall, I find myself unsatisfied with the other main finding - a correlation between A) run separability by an SVM and B) intensity of subjective experience. I'm not sure if that correlation constitutes a substantive advance, much less "a new framework for understanding how psychedelics shape neurobiology." Perhaps further and better analysis could be done to understand what ARE the differences between conditions (rest, meditation, music, movie) that cause them to separate with CEBRA.

I understand that stronger and more meaningful psychedelic experiences correlate with a larger difference between runs (and perhaps condition, although I'm not sure this is clearly shown). But what is that difference?

As far as I can tell from Fig 1, average FC difference look pretty similar across rest/meditation/music. The only manipulation that seems to have appreciable differences is eyes closed vs open (i.e. movie watching). Although that seems to be primarily because it is different at baseline, which becomes less prominent on psilocybin. But I think this is the opposite of the main claim the authors are making that "CEBRA classification accuracy is interpretable as a marker of differentiation between brain states across contexts."

Even if we accepted that psychedelics increase differences in FC between contexts, I'm not sure this would be interpretable as a neurobiologically meaningful insightful (without some idea of what of where those differences are). I believe the CEBRA models were trained separately for each participant so in fact there is no way to ascertain meaningful insights about whether psilocybin has any context/condition-specific effects (as the authors seem to claim) much less what those effects might be. They have simply shown that participants who had high MEQ scores showed more 'change' in BOLD signals across four scans during their psilocybin visit. This is not a novel insight and it does not justify the claims about 'embeddedness.'

The authors state in R2R "we rely on our conventional analyses (e.g. GFC and DCM), which are more directly tied to underlying physiology." But I am not aware of any evidence tying GFC to any physiological process.

Response to Reviewer 1

We thank the reviewer for their sustained engagement with this work. The concerns raised have sharpened both our framing of the results and the evidence underlying our central claims. Our response is presented in six sections. The main points are as follows: (i) to answer the reviewer's question about what drives the cross-context separation, we introduce a Network Perturbation Analysis that identi-

fies which functional systems' dynamics underlie the context-aligned organisation that emerges under psilocybin. This analysis reveals that the effect depends most strongly on Default Mode and Visual networks—systems which anchor opposite ends of the principal cortical gradient from internal to external processing—with smaller contributions from other functional networks, providing anatomical grounding that goes beyond statistical separability (Section 3; Fig. R1); (ii) we clarify that CEBRA operates on frame-by-frame BOLD time series—the context-aligned trajectories it reveals are the finding, with SVM accuracy serving as a descriptive readout of their separability. When temporal ordering is preserved, context-dependent separation emerges in the dynamic trajectories even where static FC summaries appear similar, resolving an apparent contradiction between static FC convergence and dynamic trajectory differentiation (Section 2); (iii) we rule out the possibility that the effect simply reflects more change in BOLD signals under psilocybin through convergent evidence including previously supplied silhouette scores across all four conditions and replication across independent dimensionality reduction methods (both provided in Revision 1), and new motion controls (response to minor comment 1.8); (iv) we clarify that individual model training was a design choice that does not preclude cross-subject inference (Section 4); and (v) we have corrected the characterisation of GFC as tied to physiology and refined the framing of embeddedness to ground it phenomenologically in the observed association between context-aligned neural dynamics and subjective experience (Sections 5–6).

Section 1: Context-aligned organisation of brain dynamics under psilocybin represents a substantive advance in understanding psychedelic action

"Overall, I find myself unsatisfied with the other main finding - a correlation between A) run separability by an SVM and B) intensity of subjective experience. I'm not sure if that correlation constitutes a substantive advance, much less 'a new framework for understanding how psychedelics shape neurobiology.' [Intervening comments are addressed in Sections 2–4.] *[. . .] They have simply shown that participants who had high MEQ scores showed more 'change' in BOLD signals across four scans during their psilocybin visit. This is not a novel insight* [addressed here] *and it does not justify the claims about 'embeddedness'* [addressed in Section 5]."

The reviewer questions whether the correlation between SVM classification accuracy and subjective experience constitutes a substantive advance, or whether this simply reflects increased signal variance under psilocybin. Before addressing both points, we clarify a distinction that bears on what follows. CEBRA reveals that under psilocybin, whole-brain activity becomes organised into trajectories that are structured by experiential context. CEBRA was trained only on temporal adjacency, without condition labels, so this structure is discovered from the data, not imposed. This context-aligned organisation, identified by CEBRA, is the central finding. Downstream, the SVM serves as an index of this organisation—high classification accuracy indicates that these trajectories have become distinct and reproducible—and the correlation with MEQ scores indicates that this organisation scales with the depth of the psychedelic state.

Our finding is that psilocybin reorganises the moment-to-moment evolution of whole-brain activity into trajectories that are structured by experiential context—and that the strength of this context-aligned organisation scales with the depth of subjective experience (Fig. 3a,c). This goes beyond showing that psilocybin changes brain activity; it demonstrates that the form of that change is context-dependent and experientially graded. The evidence for this claim is convergent (provided across multiple Extended Data Figures): CEBRA-derived embeddings show context-dependent clustering that is absent at baseline and in low-MEQ participants (Fig. R2a–d, Fig. Extended Data 7); this context-dependent clustering is evident across leading dimensionality reduction methods (PCA, t-

SNE, Isomap, Fig. Extended Data 9); and the structural organisation of these trajectories is significantly associated with next-day mindset change (Fig. Extended Data 10). Together, these findings represent the first evidence in human neuroimaging that psilocybin-induced whole-brain dynamics are organised into context-aligned trajectories graded by subjective experience and linked to next-day psychological change.

The current understanding of psilocybin's effects on brain dynamics—measured in humans, *in vivo*—rests largely on two classes of finding: changes in time-averaged functional connectivity, and increases in signal entropy or complexity, both typically quantified as context-independent summary measures that collapse the temporal information. These approaches have established that psilocybin alters large-scale brain organisation, but they have not addressed whether psilocybin reshapes the temporal structure of brain activity in relation to what a person is doing and experiencing at a given moment.

The reviewer notes uncertainty about whether between-condition differentiation is present, writing: "I understand that stronger and more meaningful psychedelic experiences correlate with a larger difference between runs (and perhaps condition, although I'm not sure this is clearly shown)." The silhouette scores reported below address this concern. They are computed within the psilocybin session across all four conditions: each score quantifies how distinctly a given condition's trajectories separate from the trajectories of the other three conditions.

The reviewer also suggests that the context-aligned organisation may simply reflect more change in BOLD signals among participants with stronger subjective effects. We examined this account and found it inconsistent with the data. Unstructured variance is an adversary to classification accuracy, not an aid—if the effect simply reflected more change in BOLD signals, trajectories would become diffuse and overlapping in the embedding, and classification accuracy would degrade. The opposite was observed. Participants with the strongest subjective effects showed the tightest clustering of context-specific trajectories, as quantified by silhouette scores—which index both between-context separation and within-context coherence—that increased significantly in high-MEQ relative to low-MEQ subjects across all four conditions (Rest: $\Delta s = 0.512$, $p = 0.0002$; Meditation: $\Delta s = 0.710$, $p = 0.0023$; Music: $\Delta s = 0.515$, $p < 0.0001$; Movie: $\Delta s = 0.631$, $p = 0.0007$; Fig. R2). This pattern—trajectories that are more distinct and reproducible, not more diffuse—is incompatible with a 'more change' (increased variance) account. This effect is evident across alternative dimensionality reduction approaches reported in our previous response (PCA, t-SNE, Isomap; Fig. Extended Data 9), and the new FD-covariate analysis confirms it is not explained by head motion (see response to comment 1.8 and corresponding updated Fig. 2c).

Taken together, the substantive advance is this: prevailing accounts characterise the psychedelic state as desynchronised or entropically disordered; our data reveal a latent organisation in the low-dimensional trajectories of brain activity—context-aligned trajectories whose structure emerges in proportion to the depth of subjective experience. The reviewer suggested that further analysis could help identify what drives the cross-context separation. We agree with this view, and the new Network Perturbation Analysis described in Section 3 was developed in response. The following sections address the reviewer's specific methodological and interpretive concerns regarding what the neurodynamic reorganisation consists of, which neural systems underpin it, and what inferences the analytical approach does and does not support.

Manuscript Revision (Results, "Context-aligned neural trajectories emerge with psychedelic experience")

The following manuscript revisions implement the key arguments developed in this section.

Clarifying the distinction between CEBRA and SVM:

> Here, we used CEBRA to generate low-dimensional embeddings of the preprocessed, frame-by-frame regional BOLD time series and used support vector machine (SVM) classification as a descriptive readout of how well session-specific embeddings distinguished between rest, meditation, music, and movie conditions [Schneider et al., 2023] (see Methods).

Introducing context-alignment and its scaling with subjective experience:

> Participants with higher MEQ30 mean scores generally exhibited higher classification accuracy, indicating that the context-specific organisation of neural dynamics scales with the intensity of subjective experience (Fig. 3c, right panel). We refer to this structured alignment of neural dynamics with experiential context as *context-alignment*.

Addressing the possibility that the effect reflects increased variance rather than structured organisation:

> Crucially, if the observed effect merely increased random noise, trajectories would become diffuse and overlapping, degrading classification accuracy. The opposite was observed. Participants reporting stronger effects showed tighter clustering (higher silhouette scores; Fig. Extended Data 7d) and higher accuracy, indicating that the change is structured rather than random.

Section 2: Static functional connectivity and dynamic trajectory analysis address different levels of neural organisation

"As far as I can tell from Fig 1, average FC difference look pretty similar across rest/meditation/music. The only manipulation that seems to have appreciable differences is eyes closed vs open (i.e. movie watching). Although that seems to be primarily because it is different at baseline, which becomes less prominent on psilocybin. But I think this is the opposite of the main claim the authors are making that 'CEBRA classification accuracy is interpretable as a marker of differentiation between brain states across contexts."'

Average FC differences across rest, meditation, and music do appear similar, and the most appreciable FC contrast—between eyes-open and eyes-closed conditions—does *decrease* under psilocybin. This observation is accurate at the level of static FC: the difference maps do appear broadly similar across eyes-closed conditions. This limitation of static FC—its inability to differentiate context-specific neural signatures despite detecting broad changes—is why the manuscript frames the ML analyses as uncovering organisation that time-averaged connectivity measures miss.

This apparent contradiction resolves because FC collapses the temporal dimension while CEBRA preserves it—these methods address fundamentally different levels of neural organisation. FC computes time-averaged pairwise correlations, collapsing the temporal dimension into a single summary matrix per condition. CEBRA, by contrast, operates on the continuous, frame-by-frame BOLD time series

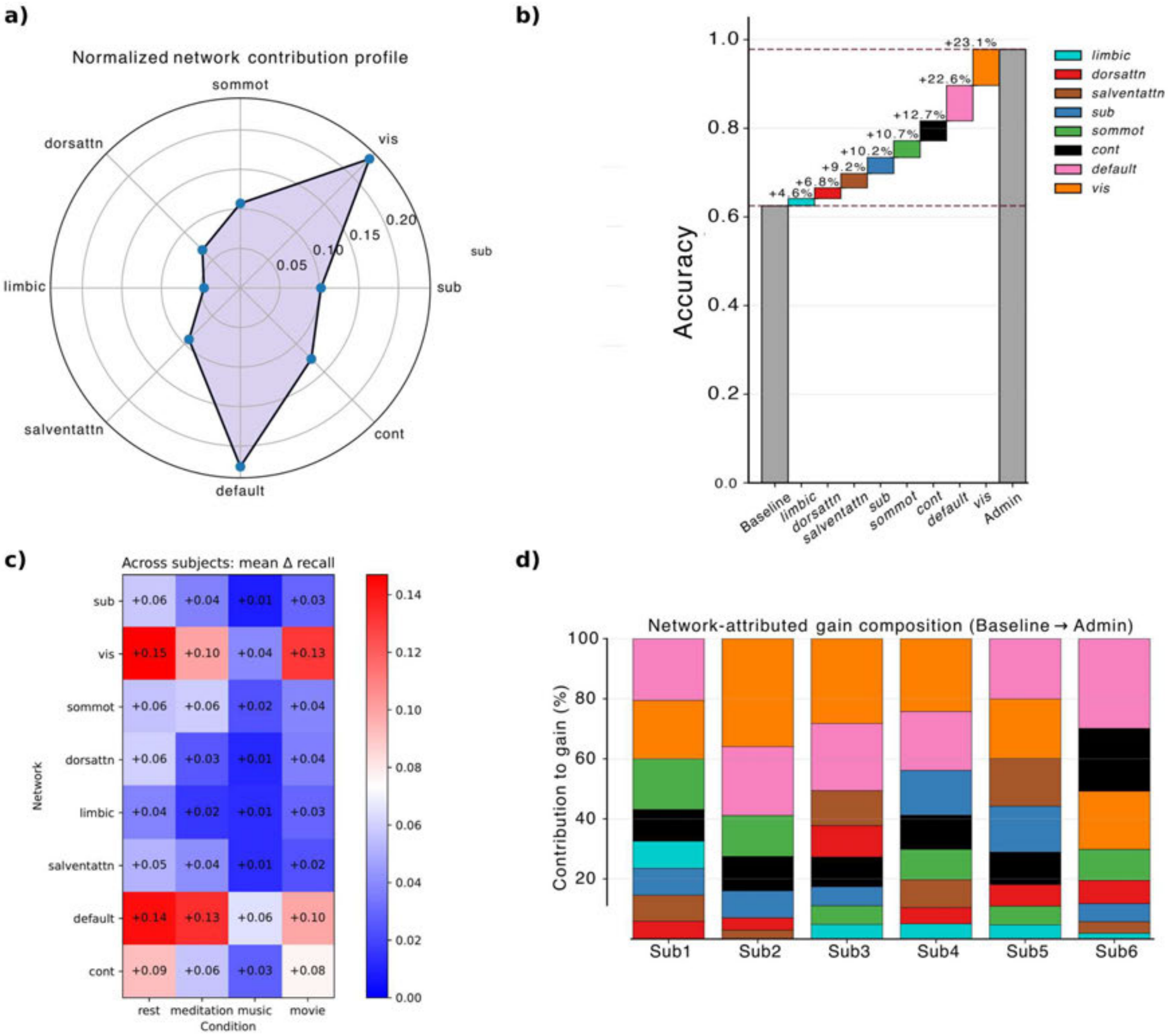

Figure R1: **Network-attributed decomposition of context-aligned trajectory organisation under psilocybin, for the six high-MEQ participants shown in Fig. R2 b,d.** For each participant, the psilocybin time series of a given functional network was replaced with that participant's corresponding baseline (no-psilocybin) dynamics, one network at a time. The CEBRA embedding and SVM classification were re-run on the resulting hybrid time series, and the drop in performance was measured: a network whose psilocybin dynamics are critical for context separation produces a large accuracy drop; one that contributes little produces a smaller drop (see Methods: Network perturbation analysis; Supplementary Information for the full attribution formula). **(a)** Normalised network contribution profile averaged across participants. Each axis represents one functional network; distance from centre indicates the relative contribution to the psilocybin-induced accuracy gain. Default Mode and Visual networks show the largest contributions. **(b)** Waterfall plot of the average additive decomposition across participants. Starting from baseline classification accuracy, each coloured segment shows one network's attributed contribution, ordered by magnitude. The final bar corresponds to the observed psilocybin accuracy. Dashed lines mark baseline and psilocybin performance levels. Contributions are normalised to sum to the total accuracy gain (see Supplementary Information). **(c)** Per-condition decomposition of network contributions. Heatmap shows the across-subject mean change in per-condition recall (Δ recall $=$ $\text{recall}_{\text{psilocybin}} - \text{recall}_{\text{substituted}}$) for each network (rows) and condition (columns), where recall is the diagonal of the row-normalised confusion matrix. Positive values indicate that replacing that network's psilocybin activity with baseline activity reduces correct classification of that condition, consistent with that network carrying condition-discriminative information under psilocybin. Default Mode and Visual networks show the largest effects across all four conditions, with graded modulation across contexts rather than condition-specific dissociation (see Supplementary Information for condition-level interpretation). Values are annotated within each cell. **(d)** Per-subject stacked bars showing the relative contribution (%) of each network to the total psilocybin-induced accuracy gain for each participant individually. Contributions are normalised so that the sum across networks equals 100% of each participant's observed gain. The consistency of Default Mode and Visual dominance across all six individually trained models (control marginally exceeded visual in one participant) indicates that the anatomical anchoring is a stable property of the data. Network abbreviations: sub, subcortical; vis, visual; sommot, somatomotor; dorsattn, dorsal attention; limbic; salventattn, salience/ventral attention; default, default mode; cont, frontoparietal control.

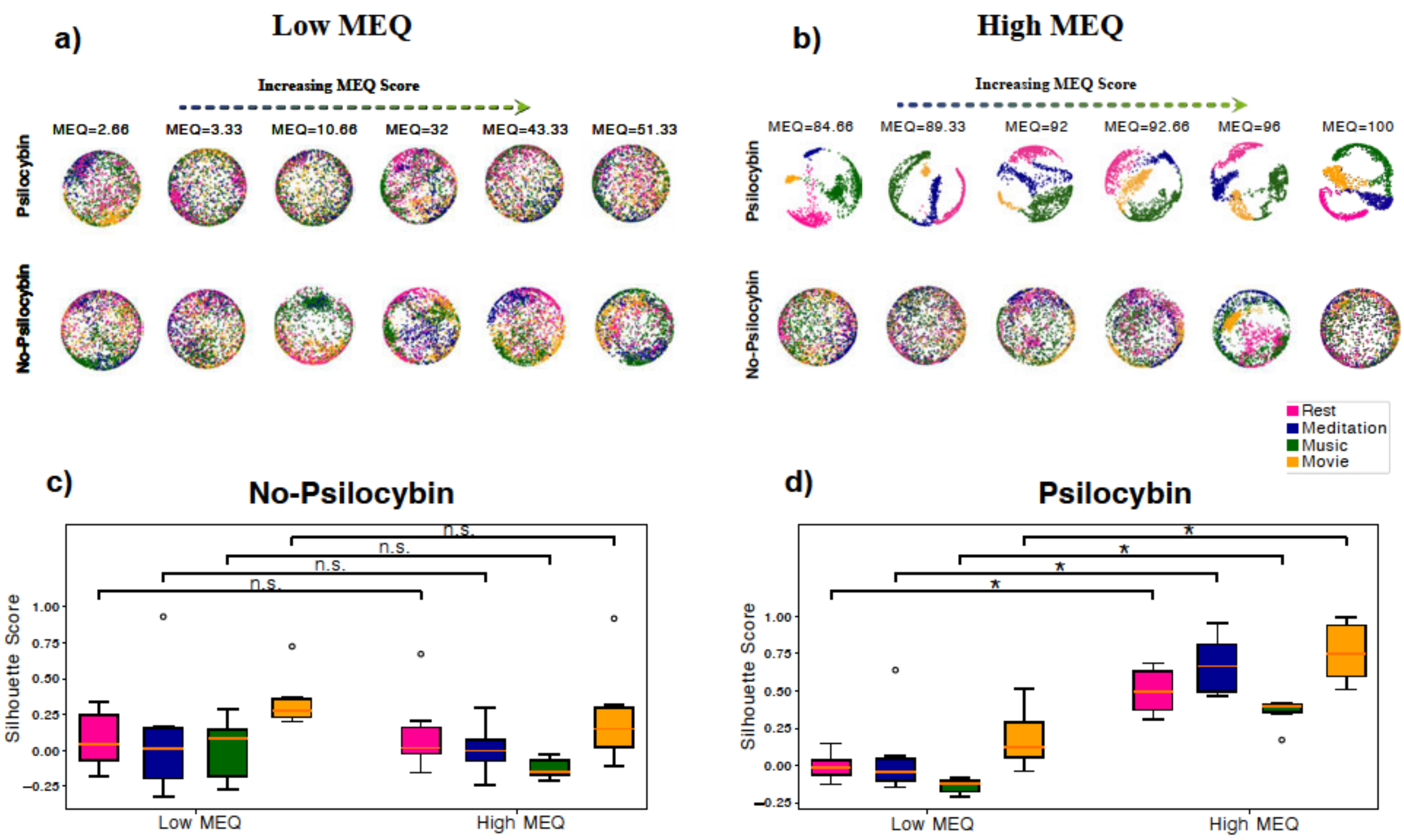

Figure R2: **Comparison of CEBRA embeddings and silhouette scores for subjects with low vs. high MEQ scores.** (**a**) Embeddings of six subjects with *low MEQ sorted by low classification accuracy* under Psilocybin and No-Psilocybin conditions. (**b**) Embeddings of six subjects with *high MEQ sorted by high classification accuracy* under Psilocybin and No-Psilocybin conditions. (**c**) Box plots of silhouette scores during the No-Psilocybin condition, showing no significant (n.s.) differences between low and high MEQ groups. (**d**) Box plots of silhouette scores during the Psilocybin condition, showing significantly higher clustering quality in high MEQ subjects compared to low MEQ subjects. Together, these results indicate that the separation of neural state representations captured by CEBRA is strongly modulated by the intensity of the subjective experience (MEQ), while baseline (No-Psilocybin) states show comparable structure across groups. Psilocybin leads to markedly stronger and more distinct embeddings in high MEQ participants, consistent with enhanced experiential differentiation.

from 332 brain regions, preserving the full temporal resolution of the data. The analytic pipeline is: preprocessed regional BOLD time series → CEBRA embedding → SVM readout. No FC matrices were used as inputs at any stage. Because these methods operate on different aspects of the signal, similar-looking FC maps across conditions do not preclude—and would not be expected to preclude—distinct temporal dynamics.

We interpret the reduction in FC contrast between eyes-open and eyes-closed states not as a loss of structure but as a relaxation of rigid sensory-state boundaries in the time-averaged signal. CEBRA reveals that as these static boundaries loosen, the moment-to-moment evolution of brain activity within each condition becomes more temporally distinctive and reproducible—more tightly locked to context. This coexistence of static convergence and dynamic differentiation shows that temporal ordering carries information that time-averaging discards.

Manuscript Revision (Discussion, “Context-aligned reorganisation of brain dynamics”):

> This pattern reflects a redistribution of integration in which associative networks participate more broadly during eyes-closed states while sensory networks become less con-

strained by baseline organisation. Importantly, the convergence of sensory-state boundaries in time-averaged FC (Fig. 2b,d) is not a loss of structure but a reorganisation compatible with the context-aligned organisation captured by low-dimensional trajectory analysis (Fig. 3a). As rigid distinctions between eyes-open and eyes-closed states relax, temporally ordered dynamics become more distinctly locked to each context.

This dissociation between static and dynamic measures extends from FC organisation to brain–behaviour association. Per-subject functional modularity under psilocybin—which decreased robustly at the group level (Fig. 2f)—was not associated with MEQ30 mean or next-day mindset change in any condition, whereas CEBRA classification accuracy, derived from the same participants, was associated with both (Fig. R3). The experientially meaningful signal resides in temporal dynamics that time-averaged summaries discard.

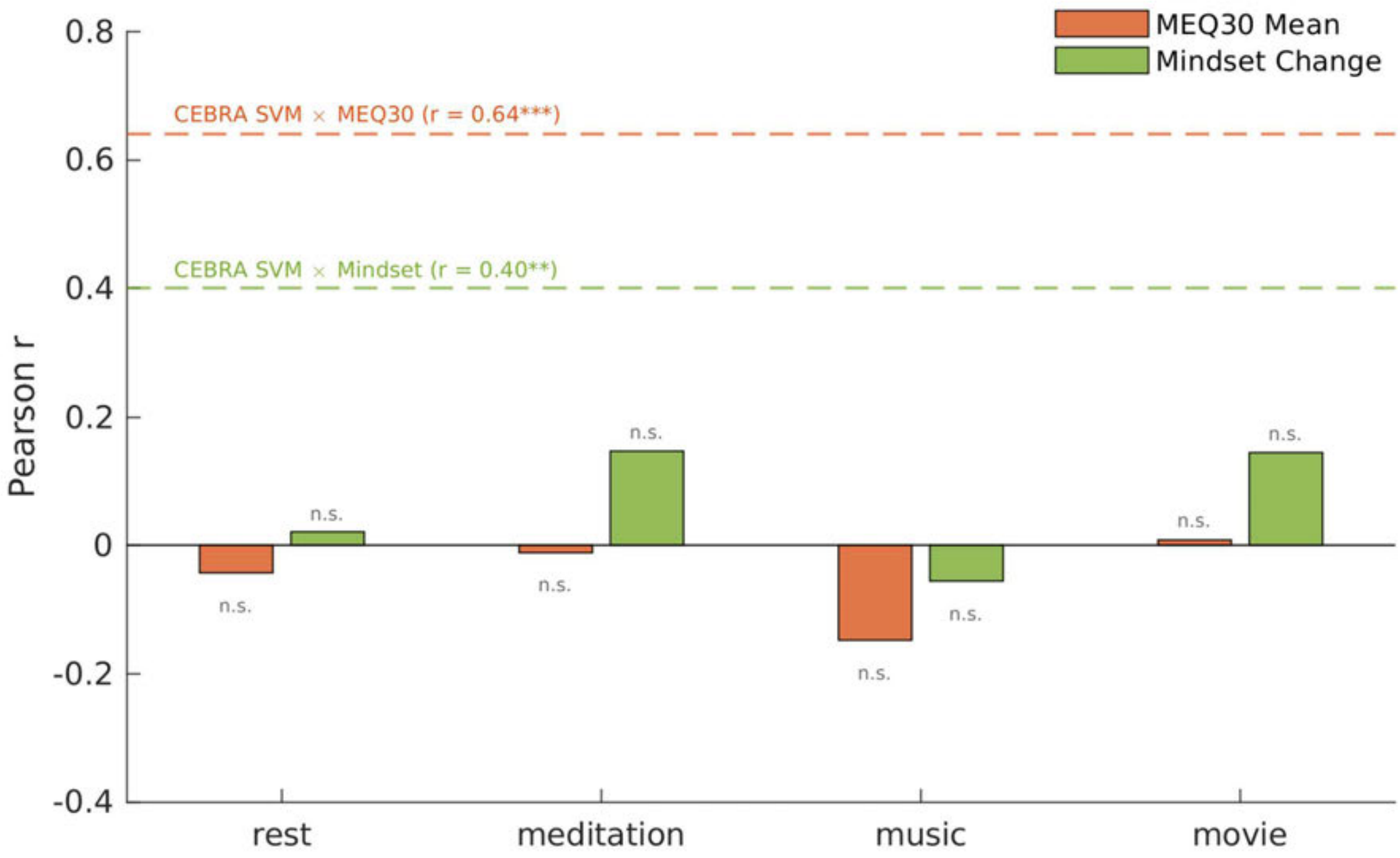

Figure R3: Pearson correlation coefficients between per-subject functional modularity (psilocybin session) and two behavioural outcomes—MEQ30 mean ($n = 54$) and next-day mindset change ($n = 53$)—across four conditions. Despite robust group-level reductions under psilocybin (Cohen's $d = -0.60$ to -0.91; Fig. 2f), modularity did not predict individual differences in either outcome (all $|r| < 0.15$, all $p > 0.28$ uncorrected; Bonferroni-corrected $\alpha = 0.0063$; confirmed with Spearman rank correlations). Dashed lines show CEBRA SVM classification accuracy correlations with the same outcomes ($r = 0.64$, $p < 10^{-6}$; $r = 0.40$, $p < 0.01$).

Section 3: The Network Perturbation Analysis provides neurobiological grounding at the level of large-scale functional systems

"Perhaps further and better analysis could be done to understand what ARE the differences between conditions (rest, meditation, music, movie) that cause them to separate with CEBRA. [Intervening comments are addressed in Sections 1–2.] *[. . .] I'm not sure this would be interpretable as a neurobiologically meaningful insight (without some idea of what or where those differences are)."*

The reviewer's central concern is that cross-context separability, on its own, is not interpretable as a neurobiologically meaningful finding without some account of what and where the differences are. This is a fair point. Latent-space separability is not, on its own, a neurobiological explanation. To address this, we introduced a 'Network Perturbation Analysis' that provides anatomical anchoring at the level of large-scale functional systems.

The logic of the analysis is as follows. For each participant, we systematically replaced the psilocybin-state time series for a given functional network with that same participant's corresponding baseline (no-psilocybin) dynamics, one network at a time. The CEBRA embedding and SVM classification were then re-run on the resulting hybrid time series, and the drop in classification performance was measured. This procedure tests how much the learned separation depends on preserving each network's psilocybin-state dynamics, measured by the drop in decoding performance under substitution, while preserving the natural statistical structure of the individual's biological signal (the mathematical details of this analysis are in the Appendix at the end of this letter and in Supplementary Information).

This analysis revealed that the learned cross-context separation depends disproportionately on preserving psilocybin-state dynamics in the Default Mode and Visual networks (∼6–8 percentage-point accuracy drop each under substitution), with smaller drops for all other networks (Fig. R1a,b). The organisation is globally distributed—consistent with a system-wide reconfiguration—but these two systems carry the greatest share of context-discriminative information.

DMN and Visual networks occupy opposite poles of the principal gradient of macroscale cortical organisation described by Margulies et al. [2016]—from internally generated, self-referential processing (DMN) to externally driven sensory processing (Visual). Yet both systems contribute uniformly across all four conditions (Fig. R1c): DMN does not dominate for internally directed contexts (rest, meditation), nor Visual for externally directed ones (movie). Rather, these are the systems that ordinarily maintain the functional separation between internal and external processing, and both are altered specifically under self- and boundary-dissolving subjective effects (embeddedness; Fig. R2d). This alteration sets the conditions under which brain activity aligns differentially with context, constituting a specific, non-trivial answer to the reviewer's question about what drives the cross-context separation.

The per-condition recall analysis provides numerical detail on this pattern (Fig. R1c). Default Mode and Visual networks exhibited the largest recall decrements across all four conditions, with graded modulation: rest showed the strongest dependence on individual network substitutions (Visual: Δ recall = +0.15; Default Mode: +0.14) and music the weakest (Default Mode: Δ recall = +0.06). Although music exhibited robust experience-dependent clustering (silhouette $\Delta s = 0.515$, $p < 0.0001$), absolute silhouette scores under psilocybin were lower for music than for other conditions (Fig. R2d), indicating weaker context-aligned clustering—consistent with its smaller per-network contributions in the perturbation analysis. This pattern may reflect two features of the experimental design. First, the substantially longer and continuously evolving auditory stimulus (11:24 min vs. 6–8 min for other conditions), which increases temporal heterogeneity in BOLD trajectories; and second, the absence of a dedicated auditory network in the Schaefer cortical parcellation, which distributes auditory-related contributions across multiple network labels. Taken together, the per-condition decomposition confirms that the DMN and Visual anchoring generalises across all four contexts with graded variation, rather than being driven by any single condition.

Per-subject analysis confirmed that individually trained models converge on the same anatomical attribution (Fig. R1d), indicating that the dominance of DMN and Visual systems is a stable property of the data, not an artefact of group averaging. The analysis was performed on the six participants with the strongest context-aligned organisation—the same participants whose silhouette scores con-

firmed significant clustering under psilocybin (Fig. R2d)—because the perturbation question (which networks' dynamics are necessary for this organisation) is only meaningful where the organisation is present.

The "difference" that CEBRA picks up is not a single static FC contrast, but a distributed, multivariate difference in the temporal organisation of brain activity across contexts. The classifier's discrimination depends more strongly on altered dynamics in Default Mode and Visual systems than on other networks, providing network-level neurobiological grounding for the effect.

We should be explicit about the scope of this analysis. It provides anatomical anchoring at the level of large-scale functional systems, not a direct account of lower-level neurophysiology; it does not establish a one-to-one mapping between specific contexts and specific networks. The network level is the appropriate grain for the phenomenon we describe: context-aligned organisation is a distributed, temporal property of whole-brain dynamics, and the perturbation analysis is designed to identify which systems' dynamics are necessary for that organisation to emerge. Within these constraints, the perturbation analysis provides a neurobiological account of context-alignment at the level that whole-brain fMRI can rigorously address.

The anatomical anchoring in DMN and Visual systems provides brain network-level grounding for the context-aligned organisation and a concrete entry point for the finer-grained mechanistic investigations that will follow in future.

Manuscript Revision (Results, "Context-aligned neural trajectories emerge with psychedelic experience"):

> A network perturbation analysis (see Methods) revealed that this context-aligned organisation is globally distributed but depends most strongly on altered dynamics in Default Mode and Visual networks ($\sim$6–8 percentage-point accuracy reduction each when their psilocybin-induced dynamics are replaced with baseline; Fig. Extended Data 8a,b)—the two systems that anchor opposite poles of the principal cortical gradient from internal to external processing Margulies et al. [2016]. This dependence was uniform across all four conditions rather than condition-specific (Fig. Extended Data 8c), indicating that the joint alteration of both gradient-endpoint systems sets the conditions for brain activity to align with context. This convergence held across individually trained models (Fig. Extended Data 8d), providing anatomical grounding for how psilocybin reorganises neural trajectories into structured patterns that reflect context.

Section 4: Individual model training does not preclude meaningful cross-subject inference

"I believe the CEBRA models were trained separately for each participant so in fact there is no way to ascertain meaningful insights about whether psilocybin has any context/condition-specific effects (as the authors seem to claim) much less what those effects might be."

The reviewer is correct that individual training generates unique latent coordinate systems. This was a deliberate methodological choice. Inter-subject variability in neural reorganisation is the phenomenon of interest. Subject-specific models capture the idiosyncratic reorganisation that would be obscured by group-level training, and for the reasons we demonstrate below, individually trained

models nonetheless converge on consistent external and anatomical benchmarks—indicating that this approach captures meaningful structure within that variability.

This concern would apply if our cross-subject inferences depended on comparing the latent coordinates themselves, which they do not. Although models were trained individually, they were all trained against the same externally imposed temporal structure: every participant underwent an identical sequence of conditions (Rest → Meditation → Music → Movie) with the same number of frames per condition. The relevant comparison is therefore between how each individual's brain activity aligns to and differentiates the same sequence of contexts. Because each model maps its participant's frame-by-frame BOLD data onto this shared timeline, the degree of alignment—quantified by classification accuracy and silhouette scores—is commensurable across subjects even though the latent coordinates are not. The resulting context-dependent organisation is visually apparent in individual embeddings (Fig. 3a,b) and quantifiable via silhouette scores (Fig. Extended Data 7c,d).

This approach supports subject-specific measures of context-sensitive brain-state organisation that can then be related across subjects to shared external variables. Three lines of evidence, each derived from individually trained models, demonstrate that context- and condition-specific insights are ascertainable from this approach:

1. **Subjective validation:** Individual model accuracy correlates significantly with a standardised measure of subjective experience (MEQ scores) during psilocybin administration, but not at baseline (Fig. 3c), indicating that the effect is state-dependent rather than reflecting stable individual differences in signal quality.

2. **Behavioural relevance:** The structural organisation of individual trajectories is significantly associated with next-day mindset change (Fig. Extended Data 10), establishing a link between context-separated brain dynamics during administration and behavioural outcomes measured the following day.

3. **Anatomical convergence:** As detailed in Section 3, the six individually trained models converge on the same network attribution, with DMN and Visual networks the dominant contributors (Fig. R1d), despite having no shared parameters or training signal.

We acknowledge that individual training constrains the level of inference. The analysis supports subject-specific measures of context-sensitive organisation that can be related across subjects via shared external variables (MEQ and mindset change), rather than a single shared population-level representation. The manuscript has been revised to ensure that claims are calibrated to this level of inference.

Section 5: Embeddedness is defined as the phenomenological correlate of context-alignment

"This is not a novel insight and it does not justify the claims about 'embeddedness."'

The reviewer challenges whether the evidence justifies the claims made about embeddedness. We have taken this critique seriously and revised the manuscript to address this concern in several specific ways. First, the definition has been grounded phenomenologically, in the revised manuscript, as the subjective experience of being continuous with, rather than separate from, the environment—an

experiential description tied to what participants report, rather than a claim about mechanism. By referring to embeddedness as "the phenomenological correlate of context-alignment," the revised text separates the experiential construct from the computational embedding spaces learned by CEBRA and TAVRNN; the co-variation between context-organised clustering and subjective effects indicates an association. Second, we refined earlier claims and calibrated them to the evidence: the revised Discussion uses more cautious language indicating that embeddedness may inform clinical approaches, and retains the latter within an explicitly speculative framing ('may reflect') supported by existing literature—positioning both as motivated hypotheses rather than conclusions of the present study.

The evidential basis for the construct is the context-alignment finding itself: context-aligned trajectories scale with MEQ scores, covary with next-day mindset change, and are anatomically anchored in Default Mode and Visual systems. Embeddedness names what participants report when this neural organisation is present—continuity with the environment—rather than an additional claim beyond the data. The revisions described here ensure that the claims about the construct are calibrated to that evidence.

Manuscript Revision (Discussion, "Psilocybin transforms embeddedness in brain activity and perception"):

> We refer to this state as embeddedness, the phenomenological correlate of context-alignment. Unlike connectedness, which implies links between separate entities, embeddedness is the subjective experience of being continuous with, rather than separate from, the environment, which emerges when brain dynamics become aligned with context in proportion to the depth of self- and boundary-dissolving effects.

Section 6: Global functional connectivity is not directly tied to physiology

"The authors state in R2R 'we rely on our conventional analyses (e.g. GFC and DCM), which are more directly tied to underlying physiology.' But I am not aware of any evidence tying GFC to any physiological process."

In our previous response letter, we described GFC as 'more directly tied to underlying physiology.' We agree this was imprecise. GFC is an undirected statistical summary of global synchronisation, not a physiological measure. The revised manuscript does not characterise GFC as tied to physiology.

Summary

The six sections above address each of the reviewer's concerns with new analyses, revised manuscript text, and refined claims calibrated to the evidence. Under psilocybin, low-dimensional trajectories of whole-brain activity became organised by experiential context, with the strength of this context-aligned organisation scaling with the intensity of self- and boundary-dissolving subjective effects and associating with next-day mindset change. This organisation coexisted with—but was not captured by—reductions in time-averaged functional modularity, which decreased robustly at the group level yet did not predict individual differences in subjective experience or next-day mindset change. A network perturbation analysis revealed that context-aligned organisation depended most strongly on altered dynamics in Default Mode and Visual networks—the systems that anchor opposite ends of the principal cortical gradient from internally generated to externally driven processing [Margulies et al.,

2016]—with this dependence holding consistently across all four conditions and across individually trained models. This pattern of results indicates that psilocybin reorganises the temporal dynamics of gradient-endpoint systems in a manner that is structured by context rather than reflecting undifferentiated increases in signal variability. Together with the motion controls detailed in the minor responses below, these analyses establish that the context-aligned organisation of neural dynamics under psilocybin is anatomically anchored in identifiable functional systems, experientially graded, behaviourally relevant, and robust to the analytical alternatives examined.

Beyond these targeted additions, the manuscript has been substantially revised throughout for precision and clarity of claims. The reviewer's sustained questioning of what drives the cross-context separation led directly to the network perturbation analysis, sharpened the distinction between static and dynamic levels of neural organisation, and prompted a more disciplined alignment between evidence and interpretation across the manuscript. The paper is measurably stronger for this engagement, and we are grateful for it.

Minor

1.1. *Were SVM classification scores higher overall during psilocybin than no drug?*

Authors' Response:

Yes. Overall, SVM classification accuracy was higher during psilocybin compared to baseline. For the subset of participants included in the analyses of this response letter (the top and bottom six subjects by MEQ scores), mean decoding accuracy during psilocybin was 0.8024, compared to 0.5217 during baseline. This pattern is consistent with the embedding structure shown in Fig. R2: clustering quality does not significantly differ between low and high MEQ groups at baseline (panel c), whereas under psilocybin (panel d) the separation between neural states is markedly stronger, particularly in high-MEQ participants.

1.4. *TAVRNN – "Simultaneously, embeddings across all networks showed tighter clustering" where is this shown?*

Authors' Response:

The tighter cross-network clustering referenced in this sentence is visible in the TAVRNN embeddings shown in Fig. 3d, where high-MEQ participants (top row) exhibit visibly more compact, network-segregated embedding configurations compared to low-MEQ participants (bottom row) across all four conditions.

Fig. Extended Data 11 provides quantification of this pattern. For each condition, mean pairwise distances between all ROIs within the same functional network were computed in the TAVRNN embedding space and averaged across participants with the highest and lowest MEQ scores, respectively. Across all four conditions, within-network distances are lower in high-MEQ participants (top row) than in low-MEQ participants (bottom row), as reflected by the heatmap diagonals. This indicates that ROIs within the same network cluster more compactly in the learned embedding space under stronger subjective effects. This pattern is observed across all conditions, confirming that psilocybin-enhanced

within-network cohesion in the TAVRNN embedding is not condition-specific but reflects a broad reorganisation that scales with subjective intensity.

1.8. *It is nice to see that the authors reran processing with more stringent criteria, however the appropriate test of whether increases in SD in the temporal pole and orbital frontal cortex is associated with motion would be the correlation between framewise displacement and SD in those areas (as has been published before and is generally attributed to the interaction of susceptibility artifact with motion), not whether the spatial pattern disappears with more stringent exclusion. A model in which FD is used as a covariate is preferred.*

Authors' Response:

We agree that explicitly modelling FD as a covariate provides a more direct test of whether the observed changes in BOLD standard deviation (SD) reflect residual motion.

To address this, we implemented a surface-based general linear model using standard tools for cortical surface inference (FSL PALM), in which vertex-wise SD change between baseline and psilocybin was regressed on subject-level FD. For each task and hemisphere, at every cortical vertex v we modelled the percentage change in SD as

$$\Delta \mathrm{SD}_v = \beta_{0,v} + \beta_{1,v}\,\Delta\mathrm{FD} + \beta_{2,v}\,\overline{\mathrm{FD}} + \varepsilon_v,$$

where $\Delta\mathrm{FD}$ is the within-subject change in mean FD (psilocybin minus baseline) and $\overline{\mathrm{FD}}$ is the subject's mean FD across the two sessions. Both FD regressors were mean-centred so that $\beta_{1,v}$ isolates the association between SD change and motion change, over and above stable between-subject differences in head motion.

Statistical inference on $\beta_{1,v}$ was performed using nonparametric permutation testing in PALM with two directed contrasts (positive and negative associations) and family-wise error correction. This nonparametric approach does not rely on Gaussian assumptions about the residuals.

The results (in Fig. R4) show that only a subset of the SD changes observed in Fig. 2c can be attributed to head motion. As expected, we identify significant positive associations between SD change and FD change mainly in regions close to air–tissue interfaces (including parts of the orbitofrontal cortex and anterior temporal lobe). This confirms that signal in these regions is difficult to disentangle from artifacts, consistent with known susceptibility-by-motion effects. However, many vertices in these regions with robust SD increases under psilocybin do not show a significant relationship with FD, and large portions of the wider cortical pattern of SD increases remain unrelated to FD. Conversely, all regions showing SD decreases under psilocybin are not associated with FD change across all conditions.

Only a small fraction of cortical vertices showed a significant association between SD change and FD change ($< 10\%$ during resting-state and $< 1\%$ in the other conditions), with effects concentrated in canonical susceptibility-by-motion regions (orbitofrontal cortex and anterior temporal lobe). When we mask the SD maps by the FD–SD regression results (removing vertices where SD change was significantly related to FD), the overall spatial organisation of SD changes is preserved. In particular, the decreases in SD in early visual cortex retain their correspondence to the decreases in global functional connectivity (GFC) in the same regions. We have updated Fig. 2c to show these FD-masked SD maps. For convenience, we also show the original and masked plots here, Fig. R4.

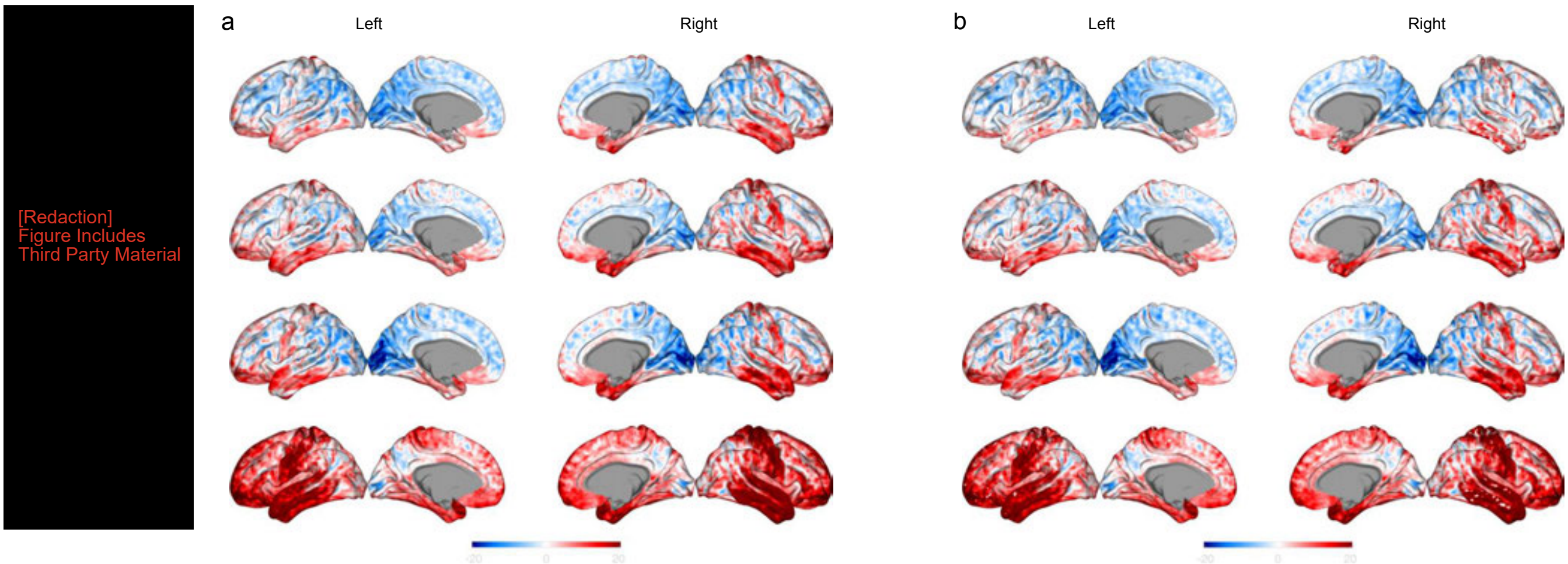

Figure R4: **(a)** Original BOLD standard deviation (SD) percentage change between baseline and psilocybin. **(b)** The same maps after masking vertices that were associated to mean framewise displacement. The largest difference is in the resting-state condition (top row), while the other three conditions show only minimal differences in regions prone to motion artifacts such as the temporal pole (less than 1% of vertices).

For completeness, we also applied the same FD-covariate model to the GFC data used in Fig. 2a. Only a very small fraction of cortical vertices showed a significant association between GFC change and FD ($< 0.1\%$ across all four conditions), and the FD-masked GFC maps are visually indistinguishable from the original GFC difference maps. This indicates that our main GFC findings are not driven by residual motion.

Together, these analyses confirm the reviewer's point that motion contributes locally to SD changes in canonical motion-sensitive regions, while the broader pattern of psilocybin-related changes in SD and GFC cannot be explained by head motion alone.

1.9. *I'm unclear as to whether Extended Data 2 shows FD during psilocybin or baseline. Please show both if possible.*

Authors' Response:

We have updated Fig. Extended Data 2 to show both baseline and psilocybin, and also present it here for convenience (Fig. R5). At baseline, a slight but statistically significant increase in mean FD was observed between the guided meditation and music listening conditions (Mann-Whitney U-test, two-sided, $p = 0.003$), and a slight decrease between music listening and movie watching ($p = 0.004$). No statistically significant differences were observed between conditions in the psilocybin session (all $p > 0.18$).

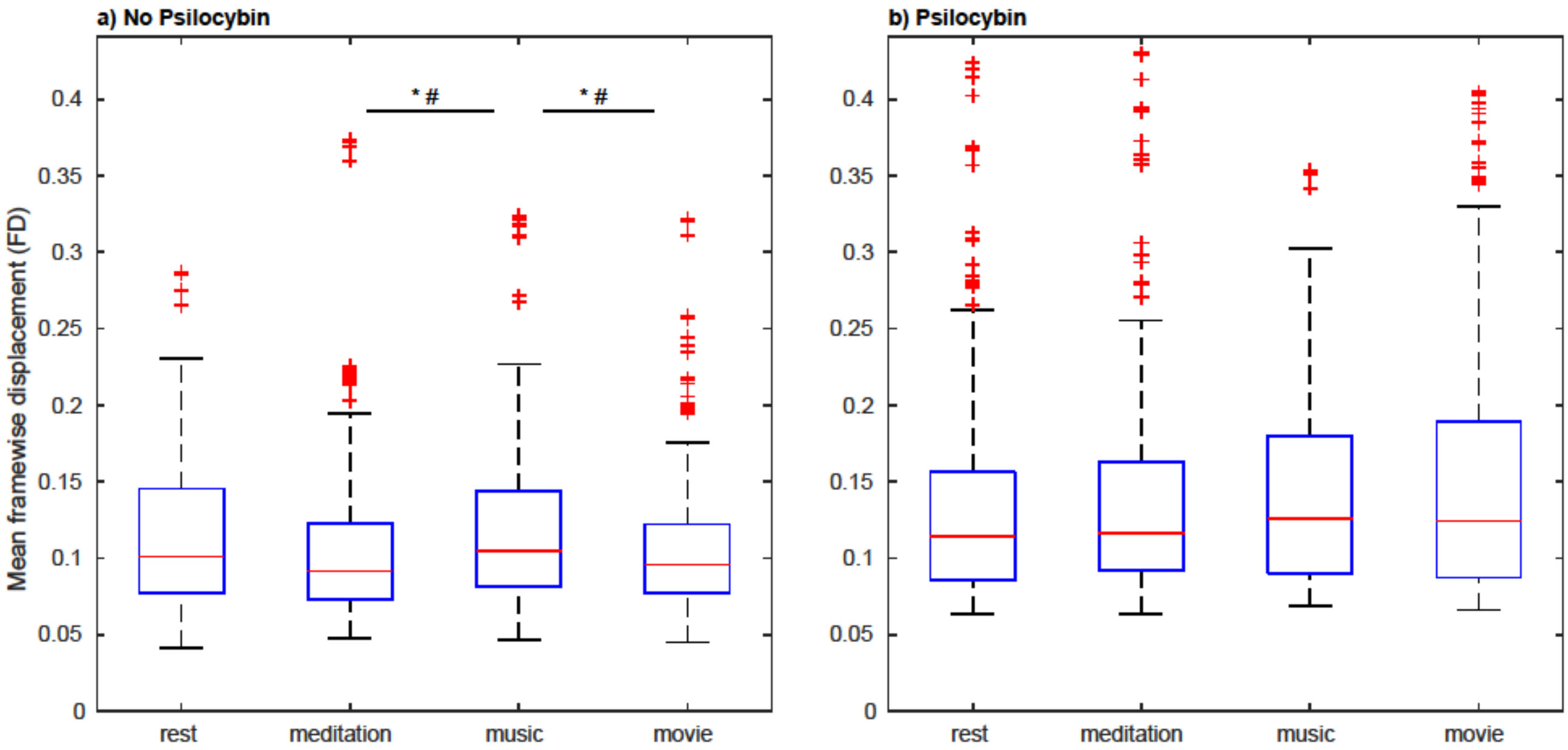

Figure R5: Head motion comparison across conditions within session, quantified via the mean frame-wise displacement (FD). **a)** Baseline session. A slight but statistically significant increase in mean FD was observed between the guided meditation and music listening conditions (Mann-Whitney U-test, two-sided, $p = 0.003$), and a slight decrease between music listening and movie watching ($p = 0.004$). **b)** Psilocybin session. No statistically significant differences were observed between conditions (all $p > 0.18$).

Referee 4

The authors did an excellent job addressing most of my questions thoroughly and thoughtfully. Their explanations were clear and well-supported. However, there is one point that would benefit from further clarification to avoid ambiguity.

***4.1**. In their response to my question about temporal filtering of the fMRI data (4.2) the authors state that "This regression step naturally eliminates very low-frequency artefacts without requiring an explicit high-pass filter." And "ICA effectively suppresses non-neuronal low-frequency fluctuations while preserving the broad spectral range of BOLD signal. For clarity, we reproduce the relevant figure here (see Fig. R9)". However, plot R9 is not a frequency plot. It is a plot of global signal change over a 500-volume acquisition. Can the authors please provide a frequency plot to support their argument?*

Authors' Response:

We agree that the argument is better supported by a frequency plot and have provided it in Fig. R6.

The reviewer's original question was *"Did the authors use any temporal filtering for the fMRI data? High-pass filtering is commonly used in task-fMRI to remove low frequency scanner drifts for example. Did the denoising process used remove particularly high or particularly low frequencies?"*.

We show that low-frequency components, particularly scanner drift and linear trends, were implicitly removed during the cleaning stage. Specifically, after optimal echo combination, we regressed out white matter, CSF, head motion parameters, and non-BOLD components identified by ME-ICA (via

tedana). This regression step naturally eliminated very low-frequency artefacts without requiring an explicit high-pass filter, but with comparable results (see Fig. R6).

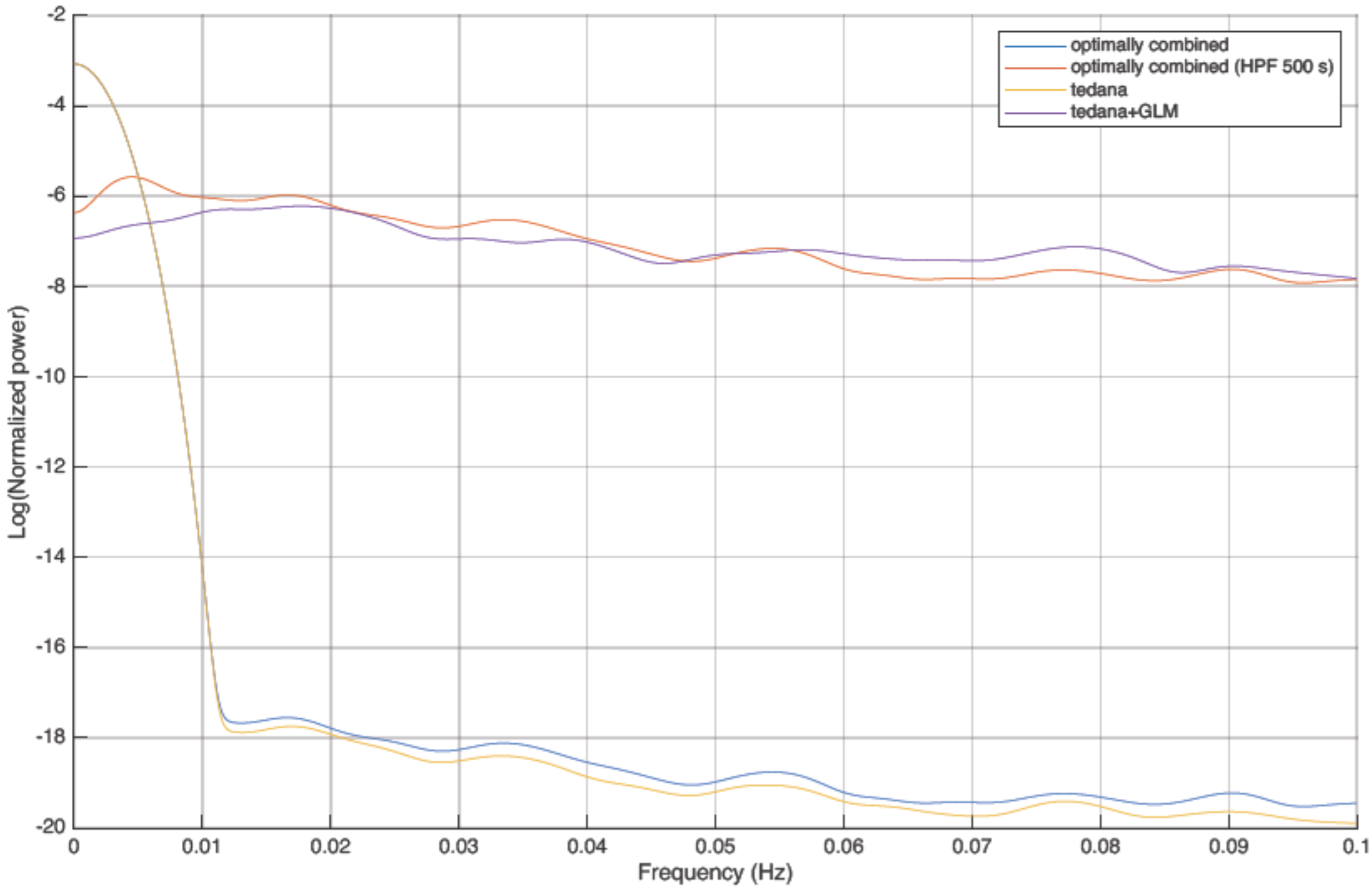

Figure R6: Impact of preprocessing and cleaning on the frequency spectrum of the global signal. The (log) power spectrum of the cleaned data (purple line) closely resembles the effect of a high-pass filter applied on the optimally-combined data (red). Both curves are higher than the unfiltered ones (blue and yellow) because the power is normalised across frequency, not because high-pass filtering increased the overall amplitude or power of the signal. This supports the conclusion that low frequencies were attenuated by cleaning the data via a GLM that included traditional regressors (head motion, white matter and cerebrospinal fluid signals), and the non-BOLD components identified via multi-echo ICA.

4.2. Additionally, in response to my question about the precuneus (4.5), the authors note that "the PCC and precuneus are anatomically adjacent and partially overlapping." This statement does not appear in the main text, so it is not a major concern, however, for the sake of precision, it is worth clarifying that, in terms of cytoarchitecture, the PCC and precuneus are distinct regions.

Authors' Response:

We have clarified that the PCC and precuneus are cytoarchitecturally distinct regions where we note about these regions in the supplementary.

Manuscript Revision (Supplementary Information):

> Multiple valid operationalisations of the DMN exist (e.g., PCC-centric, precuneus-centric, or combined posterior-midline schemes) (Andrews-Hanna et al., 2010; Thomas Yeo et al., 2011; Utevsky et al., 2014). We selected the posterior cingulate cortex (PCC) as the posterior-midline DMN node to align with our focus on changes to self-related cognition across sensory and cognitive contexts. Although the PCC and precuneus are cytoarchitecturally distinct regions, our DCM analysis models the PCC and precuneus as separate

nodes defined by stereotactic coordinates (see Methods); our use of "PCC" therefore refers specifically to the posterior cingulate node centred at the coordinates specified, not to the broader posterior midline cortex. The PCC is implicated in psychedelic neuroimaging (Carhart-Harris et al., 2012; Muthukumaraswamy et al., 2013; Smigielski et al., 2019) and this choice is in line with our prior work, including psychedelic and DCM/DMN studies (Stoliker et al., 2023, 2024) (Preller et al., 2019; Zhou et al., 2018). Given causal evidence implicating the anterior precuneus in bodily-self processing (Lyu et al., 2023), we flag an expanded model variant including an explicit precuneus node as a future, hypothesis-driven extension for studies of the bodily sense of self under psychedelics.

Appendix: Network Perturbation Attribution

Let $s \in \{1, \dots, S\}$ index subjects and $k \in \{1, \dots, K\}$ index functional networks. For each subject, the total improvement in decoding accuracy from baseline to psilocybin is defined as

$$G^{(s)} = \mathrm{Acc}^{(s)}_{\text{admin}} - \mathrm{Acc}^{(s)}_{\text{baseline}}. \tag{1}$$

To quantify the contribution of each network to this gain, we measure the drop in accuracy obtained by selectively replacing the psilocybin activity of network k with its baseline activity:

$$\Delta\mathrm{Acc}^{(s)}_{k} = \mathrm{Acc}^{(s)}_{\text{admin}} - \mathrm{Acc}^{(s)}_{\text{admin with } k \to \text{baseline}}. \tag{2}$$

Only positive contributions are considered when constructing an additive attribution. Normalised weights are therefore defined as

$$w^{(s)}_{k} = \frac{\max\big(\Delta\mathrm{Acc}^{(s)}_{k},\, 0\big)}{\sum_{j=1}^{K} \max\big(\Delta\mathrm{Acc}^{(s)}_{j},\, 0\big)}. \tag{3}$$

Each network's attributed contribution to the psilocybin-induced improvement is then

$$\mathrm{Contrib}^{(s)}_{k} = w^{(s)}_{k}\, G^{(s)}, \tag{4}$$

which guarantees that

$$\sum_{k=1}^{K} \mathrm{Contrib}^{(s)}_{k} = G^{(s)}. \tag{5}$$

For visualisation in Fig. R1a, contributions are further expressed as percentages of the total gain:

$$\mathrm{Contribution\%}^{(s)}_{k} = \frac{\mathrm{Contrib}^{(s)}_{k}}{G^{(s)}} \times 100. \tag{6}$$

References

D. S. Margulies, S. S. Ghosh, A. Goulas, M. Falkiewicz, J. M. Huntenburg, G. Langs, G. Bezgin, S. B. Eickhoff, F. X. Castellanos, M. Petrides, E. Jefferies, and J. Smallwood. Situating the default-mode network along a principal gradient of macroscale cortical organization. *Proceedings of the National Academy of Sciences*, 113(44):12574–12579, Nov. 2016. doi: 10.1073/pnas.1608282113.

S. Schneider, J. H. Lee, and M. W. Mathis. Learnable latent embeddings for joint behavioural and neural analysis. *Nature*, 617(7960):360–368, May 2023. ISSN 1476-4687. doi: 10.1038/s41586-023-06031-6.

Psychedelics Align Brain Activity with Context

Response to Reviewer 1 for MS-ID: 2025-04-08374

Referee 1

The added analyses (particularly the network-attributed decomposition) strengthen the manuscript and improve the interpretability of the CEBRA findings. At the same time, I still think the central result remains difficult to interpret neurobiologically. As currently presented, a highly complex set of time series data from multiple runs is entered into a machine-learning pipeline, which yields a separability metric that then correlates with MEQ scores. This is interesting and may indeed capture meaningful structure in the data, but it does not yet make clear which specific temporal, spatial, or network-level features of the BOLD signal are driving the effect, nor what neurobiological process should be inferred from it. For that reason, I would encourage the authors to soften some of the stronger interpretive language. In particular, I do not think the present data clearly establish that psychedelics 'align brain activity with context' (title) or that the identified patterns 'reflect the quality of subjective experience' (abstract) in a mechanistic sense. A more cautious framing would better match the evidence provided.

Two related concerns illustrate the limitations to interpretation. First, as noted in the prior reviews, the interpretation of the CEBRA/SVM findings as context-specific reorganization still seems difficult to disentangle from time-on-drug effects. In the fMRI session, conditions were acquired in a fixed order (rest, meditation, music, movie) beginning approximately 80 minutes post-dose, and the model was trained on concatenated time series from these consecutive runs. As a result, condition is confounded with time within session, and with changing drug level or effect near peak. The authors show greater clustering/separability under psilocybin, which is intriguing, but I do not think they have ruled out the possibility that this effect reflects temporal nonstationarity in drug effects rather than condition-specific reorganization per se. If additional analyses cannot address this directly, this should be acknowledged explicitly as a limitation.

Second, the reduction in separation between eyes-closed and eyes-open states may be vulnerable to a vigilance-state confound. The key comparison contrasts three eyes-closed conditions (rest, meditation, music) with an eyes-open movie condition, but I do not find a description of direct vigilance or sleep monitoring during the fMRI scans. Given that drowsiness and sleep occurs during more than 40 percent of eyes-closed resting scans, it seems important to clarify whether the authors can exclude the possibility that sleepiness or sleep contributed to the observed convergence. If this cannot be ruled out, it should be acknowledged as a limitation in the manuscript.

Overall, I think the manuscript contains an interesting and valuable dataset, and the additional analyses have improved it. My suggestion is not necessarily that the authors must fully resolve each of these issues analytically, but rather that they should adopt a somewhat more cautious interpretation and acknowledge limitations.

Response to Reviewer 1

Reviewer 1 raised three points in the final assessment: the strength of the interpretive language, a possible time-on-drug confound arising from the fixed acquisition order, and a possible vigilance confound in the eyes-closed versus eyes-open comparison. We address each below and indicate the corresponding manuscript revision and its location. In line with the Reviewer's guidance, we have moderated the interpretive language and added explicit acknowledgements of the relevant limitations, each supported by evidence set out in the Supplementary Information.

Section 1: The interpretive language has been moderated in the abstract, introduction, and discussion

"I would encourage the authors to soften some of the stronger interpretive language. In particular, I do not think the present data clearly establish that psychedelics 'align brain activity with context' (title) or that the identified patterns 'reflect the quality of subjective experience' (abstract) in a mechanistic sense."

We have moderated the interpretive language to ensure consistency with the evidence.

Manuscript Revision (Abstract):

In the abstract, the verb describing the relationship between the neural patterns and subjective experience was changed from "reflect" to "covary with":

> ...structured, context-sensitive patterns that covary with the quality of subjective experience...

We made parallel edits in the introduction and discussion. Representative examples are shown below, with the revised wording in blue:

Manuscript Revision (Discussion, "Embeddedness as brain-mind continuity"):

> ...modularity did not predict the individual differences in subjective experience or mindset change that CEBRA classification accuracy predicted, indicating that experientially meaningful organisation resides within the temporal dynamics that time-averaged summaries do not preserve.

(The verb "confirming" was replaced by "indicating".)

Manuscript Revision (Discussion, "Brain activity aligns with context"):

> Our larger sample helps clarify discrepancies in earlier reports of GFC patterns from serotonergic psychedelic studies, where spatial patterns varied across datasets and GSR.

(Previously: "Our large-sample size advanced this area of research by clarifying controversial findings from earlier small-sample serotonergic psychedelic studies".)

Manuscript Revision (Introduction):

> The finding that neural organisation tracks the transformative quality of subjective experience helps to elucidate how the psychedelic state translates into psychological change.

(The verb "elucidates" was softened to "helps to elucidate".)

We have retained the title, which describes alignment as an observed property of brain dynamics under psilocybin, consistent with the empirical findings reported, while explicitly bounding the interpretation in the discussion. The Reviewer also asked which features of the signal carry the effect; the network perturbation analysis we added addresses this, identifying the systems whose psilocybin-specific dynamics are necessary for the context-aligned organisation, while making clear that this is a systems-level account rather than a lower-level neurophysiological mechanism:

Manuscript Revision (Discussion, "Embeddedness as brain-mind continuity"):

> This anatomical anchoring is at the level of large-scale functional systems, not lower-level neurophysiology, and does not assign unique network generators to individual contexts. Context-alignment is a distributed, temporal property of whole-brain dynamics, and the perturbation analysis identifies which systems' dynamics are necessary for that organisation to emerge, providing a neurobiological account at the grain that whole-brain fMRI can rigorously address, and a concrete entry point for finer-grained mechanistic investigations to follow.

Section 2: The context-aligned organisation is not explained by fixed-order acquisition or time-on-drug

"...as noted in the prior reviews, the interpretation of the CEBRA/SVM findings as context-specific reorganization still seems difficult to disentangle from time-on-drug effects. In the fMRI session, conditions were acquired in a fixed order (rest, meditation, music, movie) beginning approximately 80 minutes post-dose, and the model was trained on concatenated time series from these consecutive runs. As a result, condition is confounded with time within session, and with changing drug level or effect near peak. The authors show greater clustering/separability under psilocybin, which is intriguing, but I do not think they have ruled out the possibility that this effect reflects temporal nonstationarity in drug effects rather than condition-specific reorganization per se. If additional analyses cannot address this directly, this should be acknowledged explicitly as a limitation."

We have added an explicit statement to the main text and expanded the corresponding Supplementary Information section. Two observations already in the main text bear directly on this concern. First, the late-onset participant PC201, who reported no subjective effect onset during the MRI session despite having received the dose, showed CEBRA embeddings under psilocybin that resembled their baseline scan; this dissociates the embedding change from time or exposure and ties it to the subjective state. Second, the network attribution in the perturbation analysis was uniform across the four conditions rather than condition-specific, which is inconsistent with a time-on-drug driver that would be expected to produce graded differences across the fixed sequence.

The expanded "Condition-order considerations" section of the Supplementary Information sets out the converging evidence: (i) the movie condition was reversed across modalities (last in fMRI, first in EEG) and separated by a 20 to 40 minute setup break, so a sequence-driven effect would not reproduce across modalities; (ii) head-motion indices showed no monotonic rise across the session; (iii) inter-individual pharmacokinetic and pharmacodynamic timing variability means the scan order does not correspond to a common exposure phase across participants; (iv) each scan lasted only 6 to 11 minutes, limiting carry-over; (v) structured CEBRA clustering was already evident during rest, the first scan, before cumulative sequence effects could accrue; and (vi) a mixed-effects model (silhouette $\sim$ scan index + (1 | participant)) showed no linear trend of clustering quality with scan order ($\beta = 0.046$, SE $= 0.041$, $p = 0.258$), while the silhouette profile across conditions (Movie $\approx$ Meditation $>$ Rest $>$ Music) and the network modularity profile (Rest $>$ Music $>$ Movie $>$ Meditation) were both non-monotonic, neither consistent with progressive order effects.

The new main-text statement, concluding the network perturbation paragraph, reads:

Manuscript Revision (Results, "Context-aligned trajectories emerge"):

> ...indicating that this reorganisation was structured rather than random. No structured embedding emerged at baseline or with low MEQ, and clustering was already strong during rest (the first condition acquired), before any sequence could accrue, indicating the low-dimensional organisation is unlikely to be driven by the fixed order of conditions (see Supplementary Information for condition-order considerations).

As requested, the limitation is also stated explicitly. The closing sentence of the Supplementary Information section reads:

Manuscript Revision (Supplementary Information, "Condition-order considerations"):

> While these analyses argue against fixed-order or pharmacokinetic effects as drivers of the CEBRA clustering, complete exclusion of subtle temporal contributions would require a counterbalanced design.

Section 3: The eyes-closed versus eyes-open convergence is not explained by reduced vigilance

"...the reduction in separation between eyes-closed and eyes-open states may be vulnerable to a vigilance-state confound. The key comparison contrasts three eyes-closed conditions (rest, meditation, music) with an eyes-open movie condition, but I do not find a description of direct vigilance or sleep monitoring during the fMRI scans. Given that drowsiness and sleep occurs during more than 40 percent of eyes-closed resting scans it seems important to clarify whether the authors can exclude the possibility that sleepiness or sleep contributed to the observed convergence. If this cannot be ruled out, it should be acknowledged as a limitation in the manuscript."

We have added a main-text statement and a new "Vigilance considerations" section to the Supplementary Information. Two electrophysiological signatures argue against a drowsiness-driven account. First, baseline eyes-closed alpha was robust: a large baseline eyes-closed versus eyes-open alpha difference can only arise when eyes-closed alpha is strong, the signature of alert relaxed wakefulness,

whereas drowsiness would attenuate this rhythm and narrow, rather than widen, the baseline gap. The premise that the baseline eyes-closed scans were drowsy is therefore not supported by the data. Second, signal complexity was highest during eyes-closed conditions under psilocybin, the reverse of the reduction that sleep and drowsiness reliably produce. These signatures derive from the separately acquired EEG session, recorded later in the day when fatigue would be more, not less, likely, which strengthens the inference.

The new main-text statement, in the EEG results subsection, reads:

Manuscript Revision (Results, "Context modulates power and complexity"):

> This convergence, observed in both fMRI and EEG, is unlikely to reflect lower vigilance during the eyes-closed conditions, given robust baseline eyes-closed alpha and elevated eyes-closed signal complexity under psilocybin (see Supplementary Information for vigilance considerations).

As requested, the limitation is stated explicitly. The closing sentence of the Supplementary Information section reads:

Manuscript Revision (Supplementary Information, "Vigilance considerations"):

> However, without explicit vigilance monitoring, a partial arousal contribution cannot be formally excluded.

No peer-reviewed data were removed in making these changes.
